# Comparison of Methods for Assessing *Chlamydia trachomatis* Transmission Intensity: A Systematic Review

**DOI:** 10.4269/ajtmh.25-0541

**Published:** 2026-09-22

**Authors:** Kristen K. Renneker, Che-Chi Lin, Jennifer L. Hsieh, Pamela J. Hooper, Robert Butcher, T. Déirdre Hollingsworth, Diana L. Martin, Anthony W. Solomon, Emma M. Harding-Esch

**Affiliations:** ^1^ International Trachoma Initiative, Task Force for Global Health, Decatur, Georgia, USA;; ^2^ Clinical Research Department, London School of Hygiene and Tropical Medicine, London, United Kingdom;; ^3^ Division of Parasitic Diseases and Malaria, Centers for Disease Control and Prevention, Atlanta, Georgia, USA;; ^4^ Big Data Institute, La Ka Shing Center for Health Informatics, University of Oxford, Oxford, United Kingdom;; ^5^ Global Neglected Tropical Diseases Programme, World Health Organization, Geneva, Switzerland

## Abstract

Field-graded trachomatous inflammation–follicular (TF) has limitations as the sole indicator of *Chlamydia trachomatis* (*Ct*) transmission for trachoma programmatic decision-making. To assess the relationship between field-graded TF and other methodologies for evaluating *Ct* transmission intensity, a systematic review was conducted. A total of 35,764 studies were screened, yielding 235 included studies from 49 countries spanning the years of data collection from 1991 to 2021 and the years of publication from 1991 to 2022. The relationship between TF and other indicators was stronger before mass drug administration (MDA) versus after MDA initiation: TF versus infection (*R*^2^ = 0.43, *P* = 0.004 versus *R*^2^ = 0.05, *P* = 0.140, respectively), TF versus seroprevalence (*R*^2^ = 0.55, *P* <0.001 versus *R*^2^ = 0.09, *P* = 0.084, respectively), and TF versus seroconversion rate (SCR; *R*^2^ = 0.52, *P* = 0.012 versus *R*^2^ = 0.26, *P* = 0.061, respectively). Post-MDA initiation, infection and SCR were highly correlated (*R*^2^ = 0.71, *P* = 0.001). These results are strongly suggestive of the poor performance of TF prevalence for guiding programmatic decision-making post-MDA initiation. Infection and/or serology should be considered in these settings.

## INTRODUCTION

Trachoma is a neglected tropical disease that can cause blindness. In the infectious phase of the disease, repeated conjunctival infection with ocular strains of the bacterium *Chlamydia trachomatis* (*Ct*) results in inflammation of the conjunctiva, which is known as active trachoma.^1^ Trachoma has a number of clinical features, including signs, such as Herbert’s pits and pannus at the corneal limbus, which were captured in the 22-sign 1966 WHO grading scheme.^2^ However, the complexity of that system precluded its reliable use at scale. As such, the WHO condensed this into the follicles, papillae, cicatrices (FPC) system in 1981^3^ and then, further condensed it into the simplified trachoma grading scheme in 1987. The simplified system was designed to be simple enough to be reliably graded by nonspecialists as part of community-level field surveys.^4^ It defines active trachoma as the presence in either eye of trachomatous inflammation–follicular (TF) and/or trachomatous inflammation–intense (TI) (Table 1).^4^^–^^15^

**Table 1:** Summary of indicators of ocular *Chlamydia trachomatis* transmission included in this review

Indicator	Description	Collection Method	Strengths and Weaknesses as a Programmatic Marker
TF	The presence of five or more follicles of at least 0.5 mm in diameter in the central upper tarsal conjunctiva^4^	Clinical examination measured as part of standard trachoma prevalence surveys^5^^,^^6^	Strengths: Easy to measure reproducibly,^4^ high correlation with *Ct* infection pre-MDA,^7^^,^^8^ and currently recommended for programmatic decision-making by the WHO. Weaknesses: Poor correlation with *Ct* infection after MDA^7^ and feasibility of standardizing measurement^5^^,^^6^ in low-prevalence settings.
TI	Pronounced inflammatory thickening of the upper tarsal conjunctiva that obscures more than half of the deep tarsal vessels^4^	Clinical examination measured as part of standard trachoma prevalence surveys^5^^,^^6^	Strength: Evidence that TI is highly correlated with infection,^9^ even in areas with a history of MDA (at implementation-unit levels).^10^ Weaknesses: Lack of evidence of a postinitiation of MDA correlation between TI and infection at the community level,^7^ harder to standardize grading of TI at scale compared with TF, and population-level prevalence of TI not currently recommended by the WHO for programmatic decision-making.
Conjunctival photography	Photograph of the tarsal conjunctiva	Photographs of the conjunctiva are taken and then, later graded for signs of trachoma	Strengths: May mitigate the challenge of training field graders, provide an opportunity for validation of field grades, and reduce biases compared with field grading.^11^ Weaknesses: Lack of standards in the literature on camera type and quality control methods^11^; high incidence of ungradable images; and implementation considerations, such as ethics, case management, and data systems.^12^
*Ct* infection	Identification of the presence of *Ct*, most commonly through a nucleic acid amplification test	Swab of the upper tarsal conjunctiva	Strength: Direct measurement of the target of antibiotics. Weakness: Concerns around the potential for increased cost and infrastructure to measure (compared with clinical signs).^13^
Serology	Identification of the presence of antibodies that indicate prior exposure to *Ct*	Blood collection by finger prick	Strengths: Direct measurement of exposure, capacity for integration with other serosurveillance programs,^14^ and ability to measure cumulative exposure to *Ct* infection over time.^15^ Weaknesses: Antigens in conjunctival *Ct* strains are shared by urogenital *Ct* strains.^15^

*Ct* = *Chlamydia trachomatis*; MDA = mass drug administration; TF = trachomatous inflammation–follicular; TI = trachomatous inflammation–intense.

The WHO recommends the surgery, antibiotics, facial cleanliness, and environmental improvement (SAFE) strategy for the elimination of trachoma as a public health problem.^16^ The purpose of the antibiotics, facial cleanliness, and environmental improvement components of SAFE is to reduce the prevalence and transmission of ocular *Ct*. However, at the time that the simplified grading system was developed, measuring ocular *Ct* infection directly (for example, with molecular analysis of conjunctival swabs) at scale was not feasible in most trachoma-endemic settings. As a consequence, the district-level prevalence of trachomatous inflammation–follicular in children ages 1 to 9 years old (TF_1–9_) as measured by trained field graders is used to guide programmatic decision-making. Currently, the WHO recommends a certain number of rounds of antibiotic mass drug administration (MDA) based on the TF_1–9_ prevalence category: one round for TF_1–9_ ≥5% and <10%,^17^ three rounds for TF_1–9_ ≥10% and <30%,^18^ and at least five rounds for TF_1–9_ ≥30%.^18^ One of the three criteria for elimination of trachoma as a public health problem is that TF_1–9_ is <5% in all formerly endemic districts.^19^

As the global prevalence of trachoma has declined, several limitations of field-graded TF have emerged (Table 1).^4^^–^^15^ These limitations are likely to be exacerbated as we approach and achieve elimination of trachoma, with TF also being unreliable as a tool for postelimination surveillance. There are a number of indicators for *Ct* transmission that have been described as “alternative” (i.e., collected in place of) or “complementary” to (i.e., collected alongside) field-graded TF (Table 1).^4^^–^^15^ There is currently no WHO guidance for the routine use of serological or infection testing for population-based prevalence surveys; therefore, data on TF will continue to be collected as routine for the foreseeable future, with the other indicators complementing the TF data and decisions being made on the whole body of evidence. Therefore, for the purposes of this review, these other indicators will be referred to as complementary. Two complementary indicators of particular interest have emerged as having the potential to help guide programs in decision-making: prevalence of *Ct* infection as measured by polymerase chain reaction and seroconversion rate (SCR); the latter is preferable to seroprevalence because of the implicit adjustment for age.^20^

Separate reviews looking at the association between field-graded TF and *Ct* infection,^7^ photograph-graded TF,^11^ and serology^15^ have been published in the past decade; however, these results have yet to be collated or assessed as a single body of evidence. In addition, since the search dates of those previous reviews, considerable amounts of new data have been published. The research question for this review was as follows: “What is the relationship between field-graded TF and other methodologies for evaluating ocular *Ct* transmission intensity?” Our objectives were to identify studies collecting data on trachoma assessed with field clinical grading of TF and at least one complementary indictor to explore the implications of the use of complementary indicators on programmatic decision-making in populations with measures of TF, infection prevalence, and SCR and to identify gaps in understanding of complementary indicators, including their use for decision-making, especially in settings postinitiation of MDA. A full population, intervention, comparators, outcomes, situation, and study type framework was developed^21^ (Supplemental File 1).

## MATERIALS AND METHODS

### Protocol.

This review protocol was prospectively registered on PROSPERO (CRD42022356013) in March 2022. Before commencing the search, the search strategy was reviewed using Peer Review of Electronic Search Strategies guidelines^22^ by a reference librarian at the London School of Hygiene and Tropical Medicine (LSHTM). As all data in the review were already in the public domain, no formal ethical review was required.

### Eligibility criteria.

Studies that met the following inclusion criteria were followed: 1) on trachoma; 2) publication from 1987 (the publication year of the WHO simplified grading system^4^ for trachoma programmatic decision-making); 3) publication in English, French, Spanish, or Portuguese; 4) reporting measurement of TF or F2/F3 from the FPC system by clinical field grading and at least one other indicator; 5) reporting primary data collection results; and 6) on humans. (TF and F2/F3 are not completely synonymous. The F2 category denotes more than five follicles, whereas TF denotes five or more follicles^13^; however, for the purposes of this review, TF and its precursor F2 were considered interchangeable, and this marker will be referred to as “TF” throughout.) The clinical signs pannus and Herbert’s pits were considered for inclusion as potential “other indicators”; however, they were ultimately excluded based on the fact that they are not markers of current active infection.

### Search methods.

This systematic search was conducted in six databases: Medline (US Medical Library/Ovid), Embase (Elsevier/Ovid), Global Health (Centre for Agriculture and Bioscience International/Ovid), Global Index Medicus (WHO), Scopus (Elsevier), and e-Theses Online Service (EThoS; University of London). The search terms were split into two main components joined with an AND term.

• Component 1: Trachoma
• Component 2: Complementary indicator terms joined with an OR term:
  ▪ Non-TF (or non-F) clinical field-graded measures of current infection (e.g., TI)
  ▪ Infection
  ▪ Serology
  ▪ Conjunctival photography

EThoS did not allow Boolean searches; therefore, the term “trachoma” was searched, and the result set was checked manually for inclusion. The full search term lists for each database are included as Supplemental File 2. All databases except for EThoS were searched on October 19, 2022. EThoS was searched on April 12, 2023.

### Study selection.

Two-stage screening was conducted in Covidence.^23^ Two authors independently reviewed titles and abstracts of search results against the inclusion criteria for potentially eligible publications. Once a consensus was reached on which titles/abstracts to include, the same two authors independently reviewed the full text of references that passed the title and abstract screen. Where the reviewers agreed, the search result was included or excluded, as appropriate; where they did not, search results were discussed, and a consensus was reached. Where full texts were excluded, the reason for exclusion was recorded. Searches for full texts were completed through the online libraries of Emory University and LSHTM using the available search tools provided by relevant journal websites and by contacting corresponding authors where contact information could be found. The following exclusion criteria were used: 1) studies with missing abstract, full text, or other key information that could not be found; 2) studies reporting only on genomic data, cellular data, or nonclinical science; 3) opinion pieces; 4) studies only reporting on conditions not related to ocular *Ct* (including sexually transmitted infections, genital chlamydia, or other infections); 5) studies only reporting trachoma measures that are not active trachoma (e.g., trachomatous trichiasis [TT]); 6) case studies; 7) systematic reviews, modeling, economic evaluations, or other secondary analyses; and 8) studies duplicating results in a less-comprehensive way (e.g., conference proceedings that were later published as full peer-reviewed articles).

### Data extraction.

One author extracted the primary data from each included study. The extracted variables included information on year of publication, year of data collection, country of data collection, MDA setting (preinitiation of MDA or postinitiation of MDA), and type of study (cross-sectional, case–control, etc.). In addition, for each indicator (field-graded TF and at least one of another clinical indicator, photography, infection, or serology), population tested size, population tested age range, population tested gender, outcome type, and outcome (prevalence by indicator, including 95% CIs where reported) were extracted. Any reported measures of association between indicators were also extracted. No assumptions about missing data were made; if information was not reported, this was recorded as “unknown” and excluded from relevant analyses. A random 20% sample^24^ of included studies was independently extracted by a second reviewer, and the results of these double-extracted studies were compared with checks for accuracy and completeness of the primary extraction. When information about history of MDA was not included in a publication, this information was abstracted from the Trachoma Atlas,^25^ which includes global binary (treated/not treated) treatment history information by year. Regions were defined according to WHO classifications.^26^ Conference abstracts where no subsequent journal article could be found were treated in the same manner as journal articles during the data extraction and quality and risk of bias assessment.

### Risk of bias assessment.

Individual studies were assessed for quality and risk of bias using the Joanna Briggs Institute (JBI) critical appraisal Checklist for Prevalence Studies or Checklist for Diagnostic Test Accuracy Studies^27^^–^^30^ based on study type. Prevalence studies that included measures of association between indicators were assessed using both checklists. The assessment was completed by a single author. A per-study score was calculated by considering a response of “yes” to each checklist item as a marker of high methodological quality, and these scores were averaged to produce an overall measure of reported methodological quality; “good” quality was defined as a score of 70% or higher. (Note that this measures quality pertaining to this review rather than the general study quality.) The body of evidence for each outcome was assessed according to Grading of Recommendations Assessment, Development and Evaluation guidelines.^31^

### Data synthesis and analysis.

During analysis, comparisons not measuring TF_1–9_ were excluded as comparing the performance of other indicators with this commonly measured indicator of programmatic importance was the primary objective. Studies not reporting a point prevalence from each studied indicator were excluded. If studies reported results at multiple geographic levels, results were deduplicated by prioritizing data reported at the evaluation unit level. Studies reporting more than one comparison by each method were deduplicated in the following manner: If serological results using different antigens were reported, results using the Pgp3 antigen (only) were kept, and others (i.e., CT-694 or a combination of CT-694 and Pgp3) were discarded. If multiple serological assay types were reported, results using the ELISA type were kept, and others (e.g., lateral flow assay results) were discarded as in papers reporting multiple assay types, the ELISA was considered the reference standard. If SCRs were estimated at multiple time points, those estimated for time periods aligning with measurements of TF_1–9_ were kept, whereas others were discarded. Measurements of complementary indicators tested only in clinically positive children or those outside of the 1- to 9-year-old age group were excluded. Studies not reporting the number of participants tested were removed from correlation calculations as sample sizes were used as weights. Although the outcomes of interest (TF_1–9_, other clinical indicator prevalence, infection prevalence, seroprevalence, and SCR) are each continuous measures, it can be useful to divide such measures into categories, which are either currently used (in the case of TF_1–9_) or could potentially be used in the future to determine programmatic action. To explore the relationships of TF_1–9_ with complementary indicators, analyses were performed comparing continuous measures against each other, by comparing categorical TF_1–9_ versus continuous measures of complementary indicators, and by comparing categorical TF_1–9_ versus potential categories of complementary indicators. Similar methods were also used to compare complementary indicators against each other.

### Field-graded versus photograph-graded TF_1–9_.

The agreement between field-graded TF and photograph-graded TF was assessed through Bland–Altman analysis.^32^ The difference between the two measures from the same population was calculated, and the mean was taken to find the mean difference. The CIs for the mean difference were calculated. Mean differences and CIs were calculated based on camera type (digital single-lens reflex [DSLR] or unaided smartphone, which was defined as the use of a smartphone camera without any additional devices, such as the head-mounted Image Capture and Processing System^33^ or the Corneal CellScope attachment^34^). Data were visualized with a Bland–Altman plot.

### TF_1–9_ versus complementary indicators preinitiation and postinitiation of MDA.

The relationships between TF_1–9_ and the prevalence estimates or SCR estimates obtained in the same populations with different indicators in preinitiation and postinitiation of MDA settings were visualized with scatterplots. Coefficients of determination (*R*^2^) between indicators were obtained by calculating and then squaring the Pearson correlation coefficients. Standard errors, *t*-values, and *P*-values were calculated. For significance testing, an alpha of 0.05 was used. Correlation coefficients were weighted based on sample size. Comparisons between TI prevalence and infection prevalence, seroprevalence, and SCR were analyzed in the same manner.

### Binary TF_1–9_ category versus continuous measures of complementary indicators.

Distributions of continuous complementary indicator results within binary categorical TF_1–9_ results (TF_1–9_ <5% versus TF_1–9_ ≥5%) were visualized using box plots, including medians with interquartile ranges (IQRs) and histograms. The lower and upper whiskers of the box plots extend to 1.5 times the lower IQR and upper IQR, respectively; points outside of this range were plotted individually as outliers.^35^

### Categorical TF_1–9_ category versus complementary indicator category in surveys used for programmatic decision-making.

To compare measurements of TF_1–9_, infection prevalence, and SCR in studies reporting results of population-based prevalence surveys used for programmatic decision-making, additional data on the purpose of the study (programmatic decision-making versus other) were extracted from studies reporting infection and/or SCR. From the subset of studies used for programmatic decision-making, survey level (EU versus a combination of several EUs) and study context (including whether the area was preinitiation or postinitiation of MDA and whether the area was considered to have unusual epidemiology [i.e., demonstrated persistent or recrudescent active trachoma or high reported TF_1–9_ but no or low reported TT]) were extracted. Outcome measure estimates were categorized into low, medium, and high categories using the following thresholds: for TF_1–9_, low: <5%,^19^ medium: 5% to 10%, and high: ≥10%^18^ and for *Ct* infection, low: <1%, medium: ≥1% to 2%, and high: ≥2%.^36^ For SCR, the thresholds used depended on the context of study; in areas that either had no history of MDA or had unusual epidemiology, the thresholds used were as follows: low: ≤1.6 seroconversions per 100 person-years, medium: 1.6 to 3.8 seroconversions per 100 person-years, and high: ≥3.8 seroconversions per 100 person-years. In areas postinitiation of MDA (including postvalidation) but with no reported unusual epidemiology, the thresholds used were as follows: low: ≤2.2 seroconversions per 100 person-years, medium: 2.2 to 4.5 seroconversions per 100 person-years, and high: ≥4.5 seroconversions per 100 person-years. Seroconversion rate thresholds were based on a recent global analysis.^20^ Two indicators were “concordant” if they fell into the same category in the same population using the categories defined above.

### Populations with three indicators (TF_1–9_, infection prevalence, and SCR): Continuous and categorical comparisons.

To explore implications of decision-making for populations with all three metrics (TF_1–9_, infection prevalence, and SCR) reported, the two-way relationships between the continuous indicator values (TF_1–9_ versus infection prevalence, TF_1–9_ versus SCR, and infection prevalence versus SCR) were visualized using scatterplots; coefficients of determination between indicators were obtained as above. Indicators were categorized using the thresholds defined above, and categorical measures were compared; two indicators were “concordant” if they fell into the same category in the same population.

Data cleaning and analyses were conducted in R (R Foundation, Vienna, Austria)^37^ using the tidyverse,^38^ cowplot,^39^ and weights^40^ packages. Because of the heterogeneous nature of the included studies and caution against comparing indicators that measure linked biological phenomena but cannot be considered directly comparable, a formal meta-analysis was not conducted. This manuscript was written in accordance with the Preferred Reporting Items for Systematic Reviews and Meta-Analysis (PRISMA) 2020 statement guidelines and checklist (Supplemental File 3).^41^

## RESULTS

### Characteristics of included studies.

A total of 35,764 studies were imported for screening. Of those, 21,150 records were unique, and of those, 883 were assessed for eligibility at the full-text screening stage. Two hundred thirty-eight papers met the inclusion criteria and were included in the review.^8^^,^^10^^,^^33^^,^^34^^,^^42^^–^^275^ Comparison between the full extraction and a random 20% of papers that were extracted by a second reviewer yielded an initial match rate of 94%. After reviewing the discrepancies, in all cases, the original extraction was accurate. Complete results of the screening process are included in the PRISMA diagram (Supplemental File 4). Of the 129 studies with insufficient information in the abstract and no available full text, 36 were conference abstracts that did not meet the inclusion criteria, and 93 were journal articles where the full text could not be found, representing 10.5% of the 883 studies assessed at the full-text stage. A majority of these missing full-text papers predated online archives of their respective journals, with 54% of studies published in 1997 or earlier (Supplemental File 5). A table of included studies is included as Supplemental File 6. Studies were included from 49 countries, spanning the years of data collection from 1991 to 2021. Of the 238 included papers, 225 were in peer-reviewed journals, 3 were PhD dissertations,^77^^,^^106^^,^^243^ and 10 were conference abstracts for which a subsequent peer-reviewed article could not be found.^69^^,^^72^^,^^86^^,^^137^^,^^142^^,^^157^^,^^160^^,^^163^^,^^201^^,^^205^ Three pairs of the 238 papers reported on different indicators as part of the same data collection activity, yielding a total of 235 unique studies. The range of publication years was from 1991 to 2022, with just over half (51%) of included studies published in 2014 or later (Supplemental File 7). The proportions of studies by reported region of data collection were 75% in the African Region, 8% in the Western Pacific Region, 6% in each of the Americas Region and the Eastern Mediterranean Region, 3% in the Southeast Asia Region, and 1% did not report the country of data collection (Supplemental File 7). A total of 183 studies reported a clinical indicator other than TF, 9 reported photograph-graded TF, 106 reported *Ct* infection, and 27 reported using serology, of which 13 reported at least one SCR analysis (Supplemental File 6). Three studies measured field-graded F2/F3,^8^^,^^171^^,^^202^ with one of these^8^ reporting results as TF.

### Overview of included comparisons.

For the purposes of this review, a “comparison” is defined as two indicators measured in the same population. Unless otherwise noted, one of these measurements is TF_1–9_. Note that studies sometimes reported either multiple non-TF indicators in the same population and/or multiple populations in the same study; hence, there is not a one-to-one relationship between study and comparison. Table 2 includes the number of studies and comparisons of field-graded TF versus another indicator. A total of 1,767 comparisons were reported, including comparisons between field-graded TF and other clinical indicators (*n* = 999), photograph-graded TF (*n* = 10), *Ct* infection (*n* = 344), and serology (*n* = 414).

**Table 2:** Number of studies and comparisons between field-graded clinical signs of trachomatous inflammation–follicular and other indicators

Complementary Indicator Type	No. of Studies	No. of Comparisons with TF
Clinical indicators		999
Follicular conjunctivitis		
<5 follicles	3	13
Papillary conjunctivitis		
TI (WHO simplified grading system)	164	701
P2/P3 (FPC)	1	4
P3 (FPC)	4	6
Follicular and/or papillary conjunctivitis		
TF and TI	13	29
TF and/or TI	53	246
Photography	9	10
Infection	106	344
Serology	27	414
Total	235*	1,767

In the FPC grading system, P3 is equivalent to TI. FPC = follicles, papillae, cicatrices; TF = trachomatous inflammation–follicular; TI = trachomatous inflammation–intense.

* This column does not sum to the total number of studies as each study may report one or more indicator types.

### Outcomes.

#### Comparison between field-graded TF and photograph-graded TF in the same population.

From the 10 included comparisons, the mean difference in prevalence measured by field-graded versus photograph-graded TF was 0.7 percentage points, with a 95% CI of −15.2 to 16.7. The range of the absolute value of differences was 0 to 17.6. Only one included study reported a population prevalence of TF by both field grading and photographs taken with an unaided smartphone,^68^ and this study reported a mean difference between the two methods of 14.8 percentage points. Six studies^8^^,^^68^^,^^196^^,^^206^^,^^216^^,^^217^ reported a population prevalence of TF by both field grading and photographs taken with a DSLR camera, and the mean difference in these comparisons was 3.9 percentage points (95% CI: −8.1 to 15.9). The range of the absolute value of differences for the studies using a DSLR was 0.2 to 17.6. The Bland−Altman plot visualizes these results (Supplemental File 8).

#### Comparison between TF and TI, infection, and serology preinitiation versus postinitiation of MDA.

**Figure 1.**
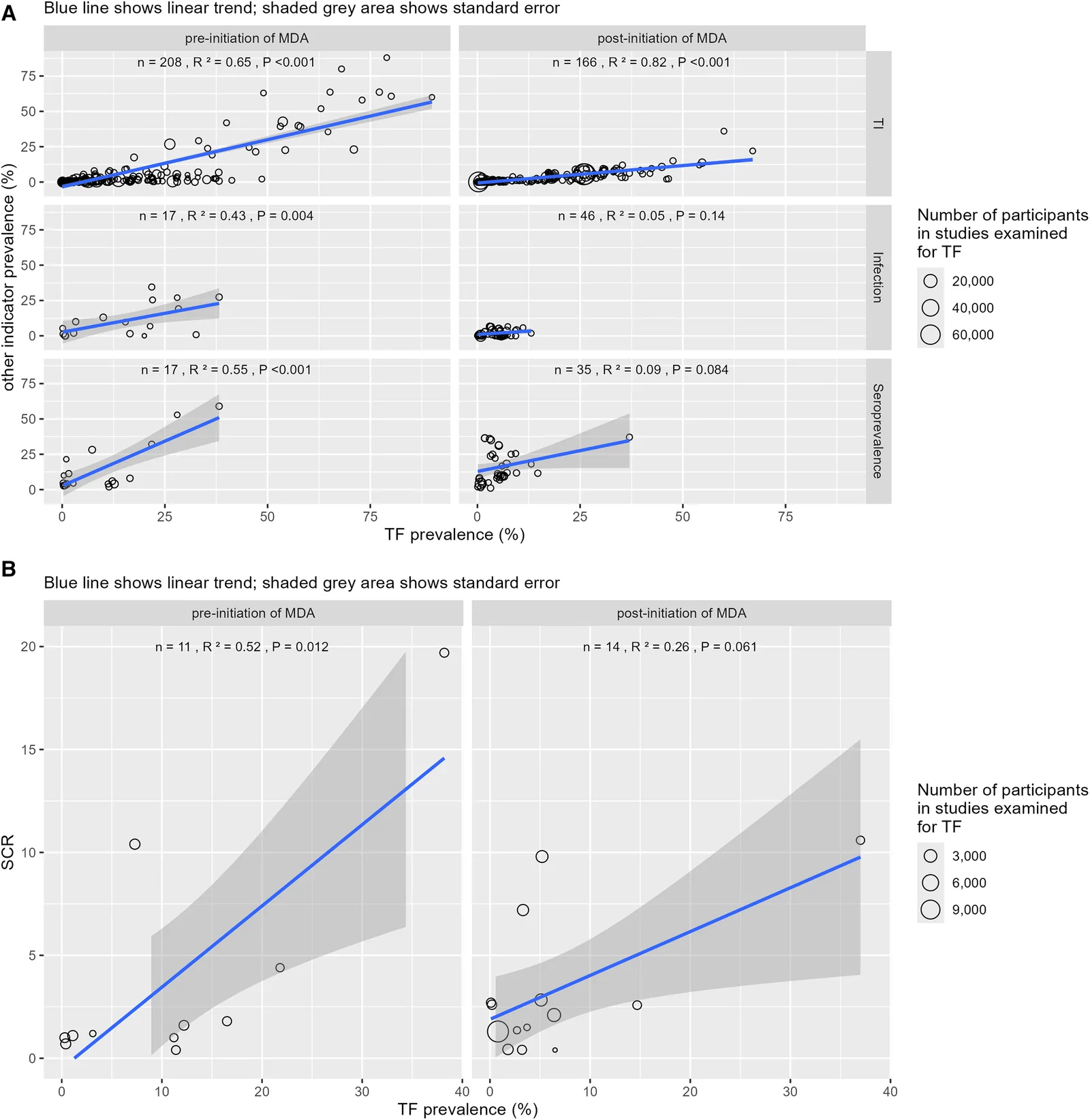
(**A**) Trachomatous inflammation–follicular (TF) in 1- to 9-year-old children vs. other indicators preinitiation vs. postinitiation of mass drug administration (MDA). Scatterplots of population-level prevalence estimates of TF vs. trachomatous inflammation–intense (TI) in 1- to 9-year-old children, infection, and seroprevalence with weighted correlations. The blue lines show the linear trend; the shaded gray areas show standard errors. (**B**) Trachomatous inflammation–follicular in 1- to 9-year-old children vs. the seroconversion rate (SCR) per 100 person-years preinitiation vs. postinitiation of MDA. Scatterplots of population-level prevalence estimates of TF vs. SCR with weighted correlations. The blue lines show the linear trend; the shaded gray areas shows standard errors.

The relationships between TF_1–9_ and TI prevalence, infection prevalence, seroprevalence, and SCR preinitiation versus postinitiation of MDA are shown in Figure 1. The comparison of TF_1–9_ and TI prevalence shows that the correlation increased postinitiation of MDA from a weighted correlation of *R*^2^ = 0.65 (*n* = 208) to *R*^2^ = 0.82 (*n* = 166) and was statistically significant both preinitiation and postinitiation of MDA (*P* <0.001 in both settings). Trachomatous inflammation–follicular in children ages 1 to 9 years old and infection were more strongly correlated preinitiation of MDA (*n* = 17, weighted *R*^2^ = 0.43, *P* = 0.004) compared with postinitiation of MDA (*n* = 46, weighted *R*^2^ = 0.05, *P* = 0.140). Trachomatous inflammation–follicular in children ages 1 to 9 years old and seroprevalence were more strongly correlated preinitiation of MDA (*n* = 17, weighted *R*^2^ = 0.55, *P* <0.001) compared with postinitiation of MDA (*n* = 35, weighted *R*^2^ = 0.09, *P* = 0.084). Trachomatous inflammation–follicular in children ages 1 to 9 years old and SCR were more strongly correlated preinitiation of MDA (*n* = 11, weighted *R*^2^ = 0.52, *P* = 0.012) compared with postinitiation of MDA (*n* = 14, weighted *R*^2^ = 0.26, *P* = 0.061).

#### Comparison between TI infection and serology preinitiation versus postinitiation of MDA.

The relationships between TI in 1- to 9-year-old children and infection prevalence, seroprevalence, and SCR preinitiation versus postinitiation of MDA are shown in Supplemental File 9. The correlation of TI prevalence versus infection prevalence decreased postinitiation of MDA, with a weighted *R*^2^ = 0.46 and *P* = 0.063 preinitiation of MDA (*n* = 8) and a weighted *R*^2^ = 0.37 and *P* = 0.273 postinitiation of MDA (*n* = 5). In both MDA settings, the weighted correlation between TI and infection was higher than that between TF_1–9_ and infection. Likewise, the correlation of TI prevalence and seroprevalence decreased postinitiation of MDA, with a weighted *R*^2^ = 0.37 and *P* = 0.145 preinitiation of MDA (*n* = 7) and a weighted *R*^2^ = 0.28 and *P* = 0.176 postinitiation of MDA (*n* = 8). The correlation between TI prevalence and SCR was relatively high both preinitiation and postinitiation of MDA, although only statistically significant postinitiation of MDA: preinitiation of MDA: weighted *R*^2^ = 0.82, *P* = 0.278 (*n* = 3) and postinitiation of MDA: weighted *R*^2^ = 0.85, *P* = 0.001 (*n* = 8).

#### Comparison between categorical TF_1–9_ and continuous measures of other indicators.

**Figure 2.**
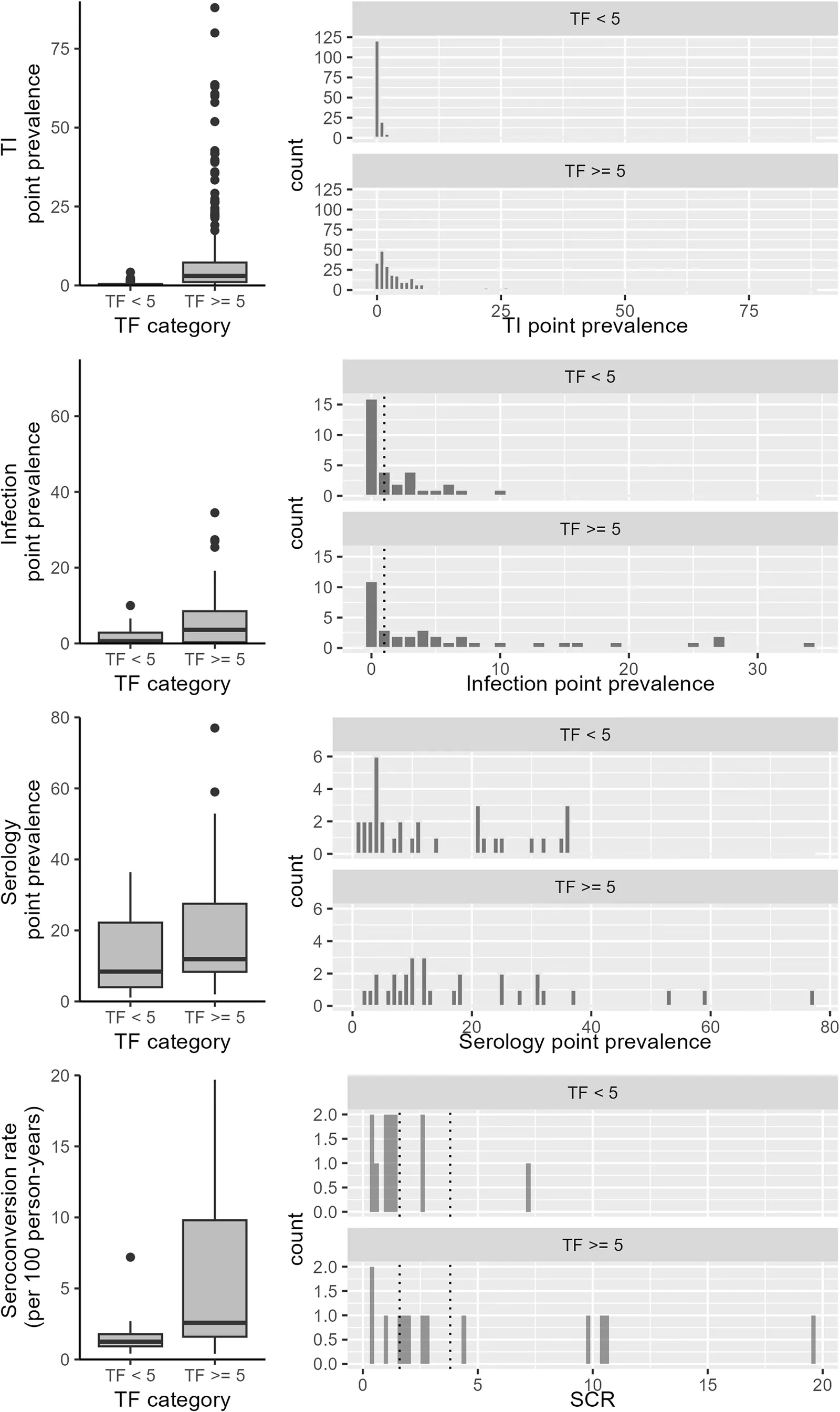
Indicator results by trachomatous inflammation–follicular (TF) in the 1- to 9-year-old children categories. The left panels display box plots by TF category (TF <5% vs. TF ≥5%), and the right panels display histograms by TF category. Dotted lines represent threshold values of interest. Infection indicates the potential threshold of 1%. Seroconversion rate (SCR) indicates proposed thresholds of 1.6 and 3.8 per 100 person-years.^23^ TI, trachomatous inflammation–intense (in 1- to 9-year-old children).

Figure 2 shows the results of each continuous non-TF indicator (TI, infection, seroprevalence, and SCR) versus categorical TF_1–9_ using the 5% programmatic threshold to categorize TF_1–9_. Within each pair of results in the rows in Figure 2, the left panels present box plots, and the right panels present histograms, where the *y* axis shows the frequency. Table 3 presents the median, IQR, minimum, and maximum of each comparison group for each non-TF indicator.

**Table 3:** Summary statistics for continuous measures of complementary indicators with trachomatous inflammation—follicular (TF) in 1–9-year-olds above and below 5% threshold

	TF category	minimum	Q1	median	Q3	maximum
TI prevalence (%)	TF <5%	0.0	0.0	0.1	0.4	4.2
	TF ≥5%	0.0	1.1	3.0	7.3	88.0
*Ct* infection prevalence (%)	TF <5%	0.0	0.03	0.6	2.9	10.0
	TF ≥5%	0.0	0.3	3.6	8.5	34.5
Seroprevalence (%)	TF <5%	1.1	3.9	8.4	22.2	36.4
	TF ≥5%	2.0	8.4	11.9	27.5	77.0
SCR (per 100 person-years)	TF <5%	0.4	0.9	1.3	1.8	7.2
	TF ≥5%	0.4	1.6	2.6	9.8	19.7

*Ct* = *Chlamydia trachomatis*; Q1 = first quartile; Q3 = third quartile; SCR = seroconversion rate; TI = trachomatous inflammation—intense in 1–9-year-olds.

### Trachomatous inflammation–follicular in children ages 1 to 9 years old category versus complementary indicator category in surveys used for programmatic decision-making.

A total of 45 discrete areas (either evaluation units or combinations of evaluation units) in 17 studies were identified as reporting infection and/or SCR data collected as part of surveys used for programmatic decision-making (Supplemental File 10). Twenty-five areas had a TF_1–9_ result warranting continued MDA (i.e., TF_1–9_ ≥5%). Of these, seven had at least one other complementary indicator that was “low,” and another five areas had at least one other indicator that was “medium.” If the typical programmatic action (based solely upon the TF_1–9_ category) was adhered to, 10 areas may have had 3 unnecessary rounds of MDA, and 2 areas may have had 1 additional round of MDA, resulting in a total of 32 unnecessary MDA rounds.

Table 4 presents (for the subset of surveys identified as being used for programmatic decision-making) the results of pair-wise categorical comparisons between TF_1–9_, infection prevalence, and SCR in three different contexts: no history of MDA, unusual epidemiology, and postinitiation of MDA (including postvalidation). In areas with no history of MDA, 100% of the TF_1–9_ versus infection prevalence comparisons (*n* = 5/5) and infection prevalence versus SCR comparisons (*n* = 2/2) were concordant, and 86% of the TF_1–9_ versus SCR comparisons (*n* = 6/7) were concordant. In the areas with unusual epidemiology, 56% of the TF_1–9_ versus infection prevalence comparisons (*n* = 9/16), 38% of the TF_1–9_ versus SCR comparisons (*n* = 3/8), and 80% of the infection prevalence versus SCR comparisons (*n* = 4/5) were concordant. In the areas that were postinitiation of MDA, 67% of the TF_1–9_ versus infection prevalence comparisons (*n* = 8/12), 50% of the TF_1–9_ versus SCR comparisons (*n* = 3/6), and 67% of the infection prevalence versus SCR comparisons (*n* = 2/3) were concordant.

**Table 4:** Concordance of indicators in surveys used for programmatic decision-making in different contexts

Preinitiation of MDA					Unusual Epidemiology					Postinitiation of MDA (including postvalidation)				
Infection Prevalence	TF Prevalence			No.	Infection Prevalence	TF Prevalence			No.	Infection Prevalence	TF Prevalence			No.
	Low	Medium	High			Low	Medium	High			Low	Medium	High	
Low	2	0	0		Low	4	0	2		Low	5	2	0	
Medium	0	0	0		Medium	2	0	3		Medium	0	0	2	
High	0	0	3		High	0	1	5		High	0	0	3	
Total concordant comparisons				5	Total concordant comparisons				9	Total concordant comparisons				8
Total comparisons				5	Total comparisons				16	Total comparisons				12

SCR	TF Prevalence			No.	SCR	TF Prevalence			No.	SCR	TF Prevalence			No.
	Low	Medium	High			Low	Medium	High			Low	Medium	High	
Low	4	0	0		Low	2	0	3		Low	2	1	0	
Medium	0	0	0		Medium	0	0	2		Medium	2	1	0	
High		1	2		High	0	0	1		High	0	0	0	
Total concordant comparisons				6	Total concordant comparisons				3	Total concordant comparisons				3
Total comparisons				7	Total comparisons				8	Total comparisons				6

Infection Prevalence	SCR			No.	Infection Prevalence	SCR			No.	Infection Prevalence	SCR			No.
	Low	Medium	High			Low	Medium	High			Low	Medium	High	
Low	0	0	0		Low	2	1	0		Low	2	1	0	
Medium	0	0	0		Medium	0	1	0		Medium	0	0	0	
High	0	0	2		High	0	0	1		High	0	0	0	
Total concordant comparisons				2	Total concordant comparisons				4	Total concordant comparisons				2
Total comparisons				2	Total comparisons				5	Total comparisons				3

MDA = mass drug administration; SCR = seroconversion rate; TF = trachomatous inflammation–follicular (in 1- to 9-year-old children).

### Comparison between infection and SCR preinitiation versus postinitiation of MDA.

**Figure 3.**
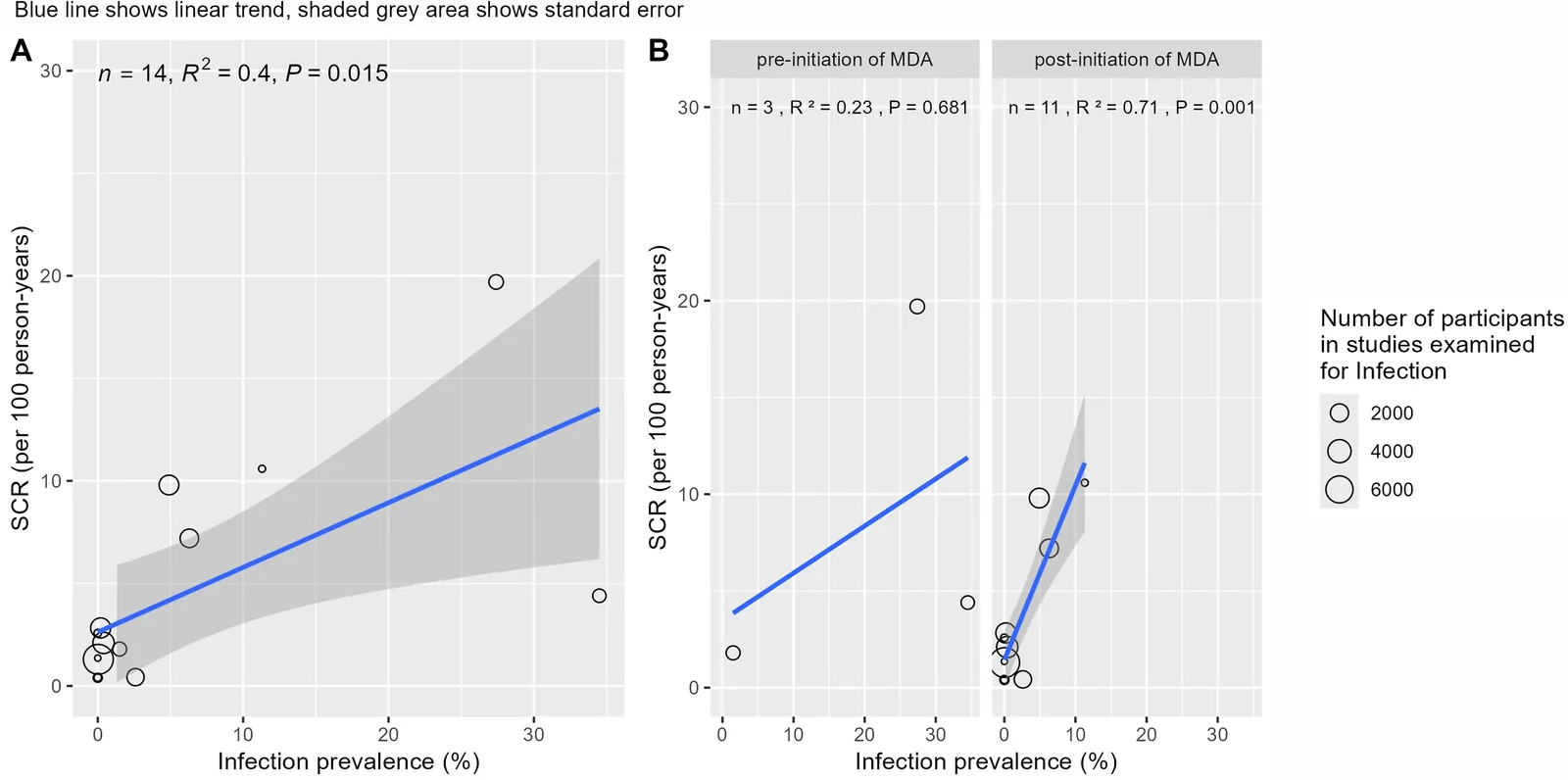
Comparisons of continuous measures of infection and seroconversion rate (SCR) for studies where both measures are reported in the same population. Scatterplots of population-level infection prevalence estimates vs. SCR. The blue lines show the linear trend; the shaded gray areas show standard errors. (**A**) All observations. (**B**) Splits the observations according to mass drug administration (MDA) setting.

Figure 3 presents comparisons of continuous measures of infection prevalence and SCR for the studies where all three measures were reported in the same populations (*n* = 14 populations reported in nine studies). Figure 3A presents the relationship between infection and SCR in all observations regardless of MDA history; in Figure 3B, the observations are split by preinitiation versus postinitiation of MDA. Figure 3A shows that the correlation between infection and SCR was significant (weighted *R*^2^ = 0.4, *P* = 0.015); however, when split by MDA history, there was no correlation in pre-MDA settings (weighted *R*^2^ = 0.23, *P* = 0.681), whereas the correlation in postinitiation of MDA settings was strong (weighted *R*^2^ = 0.71) and statistically significant (*P* = 0.001). There were only three observations pre-MDA. All pre-MDA observations were from the Pacific Islands, whereas all postinitiation of MDA observations were from Africa.

Comparisons of TF_1–9_ and infection prevalence as well as TF_1–9_ and SCR separately for the populations with all three indicators reported are presented in Supplemental File 11. For each of the TF_1–9_ versus infection prevalence and TF_1–9_ versus SCR comparisons, the weighted correlations were moderate and significant (weighted *R*^2^ = 0.49, *P* = 0.006 and weighted *R*^2^ = 0.54, *P* = 0.003, respectively), although the sample size was small (*n* = 14).

Table 5 presents categorical comparisons of measures of TF_1–9_, infection prevalence, and SCR for the studies in which all three measures were reported in the same populations (regardless of the context of the study or whether it was used for programmatic decision-making). Of 14 total comparisons with all three measures, 43% of comparisons of TF_1–9_ versus infection prevalence (*n* = 6), 57% of comparisons of TF_1–9_ and SCR (*n* = 8), and 79% of comparisons of infection prevalence versus SCR (*n* = 11) were concordant.

**Table 5:** Categorical comparisons of TF_1–9_, infection prevalence, and SCR

Infection Prevalence	TF Prevalence		
	Low	Medium	High
Low	3	3	1
Medium	0	0	1
High	2	1	3

SCR	TF Prevalence		
	Low	Medium	High
Low	4	2	0
Medium	0	1	2
High	1	1	3

Infection Prevalence	SCR		
	Low	Medium	High
Low	5	2	0
Medium	0	1	0
High	1	0	5

SCR = seroconversion rate; TF = trachomatous inflammation–follicular (in 1- to 9-year-old children).

### Risk of bias and study quality assessment.

The mean quality score for studies assessed using the JBI Checklist for Prevalence Studies was 74%, and the mean quality score for studies assessed using the JBI Checklist for Diagnostic Test Accuracy was 83% (Supplemental File 6). Of the 220 studies assessed using the JBI Checklist for Prevalence Studies, 57% (*n* = 125) achieved a score of 70% or higher; of the 42 studies assessed using the JBI Checklist for Diagnostic Test Accuracy, 79% (*n* = 33) achieved a score of 70% or higher. When categorized by indicator, the mean quality scores were 72% for studies reporting clinical indicators other than TF, 41% for studies reporting photograph-graded TF, 62% for studies reporting infection, 79% for studies reporting seroprevalence, and 83% for studies reporting SCR. Because of the majority of the contributing studies being observational, a “low” GRADE rating for each outcome was given.

## DISCUSSION

This review explored the relationship between TF and other indicators reported in 235 different studies over a 30-year period, including data from 49 countries and representing all trachoma-endemic WHO regions.

The comparisons of TF_1–9_ with *Ct* infection, seroprevalence, and SCR in preinitiation versus postinitiation of MDA settings showed a similar pattern for each indicator; in each analysis, the weighted correlation between TF and the other indicator was stronger preinitiation versus postinitiation of MDA. A previous review comparing TF and infection found similar results.^7^ Given that the relationship deteriorates for each of the three pair-wise comparisons with TF (TF versus infection, TF versus seroprevalence, and TF versus SCR), it is likely that the indicator that has changed its performance postinitiation of MDA is TF. Thus, these results are strongly suggestive of poor performance of TF prevalence for guiding programs on decision-making after MDA has commenced. Conversely, infection and SCR were strongly and statistically significantly correlated postinitiation of MDA, although there were few studies containing data on TF_1–9_, infection, and SCR in the same population.

With small sample sizes (*n* = 13 for TI versus infection and *n* = 11 for TI versus SCR), evidence suggests stronger correlations between TI and infection preinitiation of MDA and between TI and SCR postinitiation of MDA compared with TF and infection or TF and SCR at these time points. Although TI is often considered more closely associated with current conjunctival *Ct* infection than TF, the evidence base is limited. Data from a previous systematic review^7^ indicate that TF prevalence is more strongly correlated than TI prevalence with population-level infection prevalence, whereas a latent class analysis,^235^ which estimated diagnostic test characteristics at the individual level, reported that TI had a much higher specificity for *Ct* compared with TF and *Ct*. However, given that 1) intergrader agreement on TI has been shown to be lower than TF^4^ and in our experience, varies considerably between different groups of graders (which may depend on the degree of emphasis given in training to the “pronounced inflammatory *thickening*” part of TI’s definition) and because of 2) the reduction of the correlation between TI and infection evident in postinitiation of MDA settings, challenges would remain with using TI as an indicator of *Ct* transmission intensity.

When comparing results of TF_1–9_, infection prevalence, and SCR in the subset of studies reporting data used for programmatic decision-making, almost half (48%) of the areas where TF_1–9_ was greater than the threshold for MDA (TF_1–9_ ≥5%) had at least one other indicator with a result that was low or medium. Considering these results, populations for which programmatic decisions were based solely on TF_1–9_ may have proceeded with additional rounds of MDA (and later, trachoma impact surveys) that may not have been warranted based on infection and/or serology data. The additional cost of adding complementary indicators to standard trachoma surveys must be weighed against the potential additional costs of unnecessary rounds of MDA and subsequent surveys. Similar justification has been made on the cost savings of trachoma impact surveys; it is a relatively costly activity, but if it demonstrates that MDA can be stopped, it ultimately saves programs money.^276^ This study has provided a comprehensive review of data available, which can allow stakeholders to make future recommendations.

When comparing continuous measures of TI, infection, and serology with categorical TF_1–9_, in each comparison, the median complementary indicator measure was higher for the higher TF_1–9_ category. Generally, when TF_1–9_ was under the 5% threshold, measures of infection and SCR tended to also be low; when TF_1–9_ was over the 5% threshold, measures of infection and SCR had a wider range of values (i.e., could be high or low). This suggests that making programmatic decisions based solely on TF_1–9_ has a propensity for “overcalling” (i.e., continuing MDA unnecessarily) rather than “undercalling” (i.e., stopping MDA prematurely) treatment decisions, resulting in unnecessary MDA rounds, unnecessary expense, and delayed validation of elimination of trachoma as a public health problem.

In the subset of populations that included TF, *Ct* infection, and SCR, the infection versus SCR comparisons had a higher rate of concordant results compared with either of the comparisons with TF, which suggests that SCR is a better marker for true new infections and a good estimate of the force of infection compared with TF. In the comparison of continuous infection and SCR data, the weighted correlation between these measures was strong and significant in postinitiation of MDA settings. Additionally, in studies reporting data used for programmatic decision-making, the concordance of infection and SCR was as high or higher than either the TF_1–9_ versus infection or TF_1–9_ versus SCR comparisons in all three settings. However, the sample size for these comparisons was small (*n* = 14).

Recent attempts to produce a serological cutoff based on SCR acknowledge the existence of a gray area between proposed thresholds indicating high probability of elimination (or not) where additional measurements or context-specific consideration may be needed to determine future programmatic activity.^20^ Relatively few included studies that reported the use of serological indicators included an estimation of SCR, and even fewer included both SCR and infection data. Given the growing evidence that SCR has a potential use for programmatic decision-making,^20^^,^^277^^,^^278^ more collection and analysis of serological and infection data in a variety of trachoma settings would be beneficial. The WHO has recommended that complementary indicator data be collected and used for programmatic decision-making in areas where trachoma is persistent or recrudescent.^279^ The WHO also recently published a Target Product Profile for trachoma surveillance that identified additional epidemiological contexts in which complementary data could be beneficial, including in newly suspected trachoma-endemic areas, after discontinuation of MDA, and in areas with high TF_1–9_ but little evidence of its sequela (TT) in adults.^280^ Work by Tropical Data to standardize the collection and analysis of infection and serology data as part of routine trachoma surveys is ongoing.^5^

Conjunctival photography is a potential solution to several problems with field grading of signs of trachoma, including allowing review of grading decisions by outside experts, the adjudication of border cases by multiple graders,^281^ and the potential for machine learning technologies to remove human subjectivity and reduce overall error.^282^ Comparing field-graded TF with photograph-graded TF, we found that the mean prevalence by each method was very close, but the 95% CIs were wide and crossed zero (meaning that one type of grading did not consistently overestimate or underestimate the other). Despite different inclusion criteria and key outcome measures, these results are consistent with a systematic review published in 2021 showing high agreement between the two methods.^11^ However, only one included study in either review compared photographs taken with an unaided smartphone against field grading. A recently published study showed very poor sensitivity of smartphones versus field grading for TF and a high proportion (20%) of photographs being ungradable because of poor photograph quality.^283^ Thus, further work is needed to ensure accurate and reproducible smartphone photographs in the field and their grading. Key challenges remain in deploying the use of photography at scale, including needing to develop minimum criteria for photographers, camera equipment, and image quality, as well as reducing the proportion of images that are ungradable and understanding the risk of bias posed by ungradable images.^12^ Although routine use of the International Coalition for Trachoma Control’s manual for taking high-quality field photographs^284^ will help overcome some of these issues (such as decreasing the proportion of ungradable photographs), the trachoma community has agreed that further work is needed before recommending the routine use of photographs to replace field grading.^12^^,^^282^

This review had several limitations. Although mean quality scores of included studies were high, these varied by indicator, and the observational nature of the included studies conferred a “low” GRADE rating for each outcome, indicating that our confidence in the summary statistics output is limited. The wide scope yielded many studies to be screened for eligibility from a time period spanning over 30 years, and there were studies where the full text was unable to be located, in part because of publication dates before widespread digitization; all care was taken to locate records wherever possible. A majority of these studies predate the use of serological detection platforms for trachoma^285^ and did not reference data collection beyond clinical indicator types in their abstracts; therefore, the effect on the results for the nonclinical indicator types is likely minimal. The wide scope also led to a heterogeneous final dataset with different study types, purposes, and methodologies (including different grader training systems, different nucleic acid amplification tests, different serological assays, etc.), which led, in part, to the decision to not pursue a meta-analysis. In addition, we were unable to complete a full double data extraction or double quality and risk of bias assessment; however, comparing full data extraction with a random sample of 20% of papers that were double extracted yielded a high degree of agreement, lending confidence to the accuracy of the full data extraction. Nevertheless, because of the scope of the review, there remains a risk of inaccuracy owing to human error. The majority of the included studies in this review came from published literature, which introduces the risk of publication bias. In our case, there may be less of a risk of prevalence studies with results under programmatic thresholds remaining unpublished as country programs must provide evidence that thresholds have been achieved to be validated by the WHO as having achieved the elimination of trachoma as a public health problem.^19^ However, there is undoubtedly a risk of bias attributed to the nonstandard application of complementary indicators as it is probable that additional indicators (especially infection and serology) are more likely to be collected in areas with “unusual” epidemiology. Furthermore, for infection data, in the absence of publications demonstrating a more rigorous analytical approach to determine proposed thresholds (as there is for SCR),^20^^,^^278^ thresholds for this analysis were based on values proposed less formally (i.e., at an international meeting of stakeholders); the use of different thresholds could alter the results of the categorical analyses. In addition, although we extracted (and abstracted) data on whether settings had ever conducted MDA, we did not extract data on the timing of data collection postinitiation of MDA; as there is evidence that both infection and TF prevalence change over time after the cessation of MDA,^286^ the timing of the postinitiation of MDA data collection could influence the results of these indicators. Lastly, a number of comparisons had relatively small sample sizes; of note are those populations reporting both infection and SCR (*n* = 14). There is a chance that some studies with these data did not meet the inclusion criteria if they did not also report field-graded TF. Because of the small number of studies with these data and lack of diversity in settings in the studies that were included, it is likely that outliers disproportionately affected these results. In addition, it is likely that there are a number of studies including infection and serology data that have been published since the cut point of this review (October 2022), which could be included in future systematic reviews.

Because categorical thresholds are particularly useful for programmatic decision-making, further work to propose meaningful thresholds for infection would be beneficial. Further studies should also look to better understand how each of the included indicators relates to longer-term sequelae of the disease: trachomatous scarring, TT, and eventual vision impairment and blindness.

## CONCLUSION

Trachomatous inflammation–follicular is not strongly correlated with other indicators of transmission of ocular *Ct* (i.e., infection prevalence and SCR) in postinitiation of MDA settings. With the global target of elimination of trachoma as a public health problem by 2030^287^ and evidence that TF has a tendency to overcall transmission intensity, resulting in unnecessary, costly, and time-consuming additional MDA and survey rounds, the evidence supports inclusion of these indicators as part of the global strategy to achieving a world free from trachoma.

## Supplemental Materials

10.4269/ajtmh.25-0541 Supplemental Materials
