# Supplementary material for: Comparison of Methods for Assessing Chlamydia trachomatis Transmission Intensity: A Systematic Review
Source: Am J Trop Med Hyg. 2026 Sep 22;115(5):903–22. doi: 10.4269/ajtmh.25-0541 (PMC13615270; doi:10.4269/ajtmh.25-0541)
Supplement: Supplementary file 1 [file tpmd250541.SD1.pdf]

S1. Picost framework

Table – PICOST Framework

	Term	Question	Response
**P**	Population or Problem	What are the characteristics of the Population or patient? What is the Problem, condition, or disease you are interested in?	Populations where TF has been diagnosed clinically and at least 1 alternative and/or complementary indicator
**I**	Intervention or Exposure	How do you wish to Intervene - what do you want to do with this patient - treat, diagnose, observe, etc.?	Diagnosis of trachoma via non-field-graded clinical TF or FPC (the precursor to TF)
**C**	Comparison	What is the Comparison or alternative to the intervention - placebo, different drug or therapy, surgery, etc.?	Diagnosis of trachoma via field-graded clinical TF or FPC (the precursor to TF)
**O**	Outcome	What are the possible Outcomes - morbidity, death, complications, etc.?	Prevalence of trachoma as measured by different indicators and correlations between different measures
**S**	Situation	Context or location	Populations in trachoma-endemic (or formerly-endemic) countries
**T**	Type of Study	Study type(s), such as systematic reviews, randomised control trials (RCTs), observational studies, economic evaluations, or others.	Randomized control trials, observational studies, longitudinal studies, cross-sectional studies
