# Supplementary material for: Comparison of Methods for Assessing Chlamydia trachomatis Transmission Intensity: A Systematic Review
Source: Am J Trop Med Hyg. 2026 Sep 22;115(5):903–22. doi: 10.4269/ajtmh.25-0541 (PMC13615270; doi:10.4269/ajtmh.25-0541)
Supplement: Supplementary file 2 [file tpmd250541.SD10.pdf]

S10. Table of studies used for programmatic decision-making

| Study ID (First author, year) | Country | Survey level | Area Surveyed | Year* | MDA status | Population tested (for TF) | TF prevalence (%)** | *Chlamydia trachomatis* infection prevalence (%)*** | SCR (per 100 person-years)^ | context | Typical programmatic action based on TF prevalence | Notes |
|---|---|---|---|---|---|---|---|---|---|---|---|---|
| Senyonjo 2018 | Ghana | combination of several implementation units | Overall | 2015 | history of MDA | 11,730 | 0.8 (0.6 - 1) | 0.04 (0 - 0.1) | 1.3 | post-MDA (no unusual epi) | no MDA warranted | infection age range: 1-9 years, SCR context: post-MDA |
| Senyonjo 2018 | Ghana | implementation unit | Wa | 2015 | history of MDA | 1,357 | 1.1 (0.5 - 1.9) | 0 | | post-MDA (no unusual epi) | no MDA warranted | infection age range: 1-9 years |
| Senyonjo 2018 | Ghana | implementation unit | West Gonja | 2015 | history of MDA | 1,148 | 1.1 (0.4 - 1.2) | 0 | | post-MDA (no unusual epi) | no MDA warranted | infection age range: 1-9 years |
| Senyonjo 2018 | Ghana | implementation unit | Bole/Sawla-Tuna-Kalpa | 2015 | history of MDA | 1,307 | 0.5 (0.2 - 1) | 0.07 (0 - 0.12) | | post-MDA (no unusual epi) | no MDA warranted | infection age range: 1-9 years |
| Senyonjo 2018 | Ghana | implementation unit | Zabzugu-Tatale | 2015 | history of MDA | 1,345 | 1 (0.5 - 1.5) | 0.2 (0 - 0.6) | | post-MDA (no unusual epi) | no MDA warranted | infection age range: 1-9 years |
| Migchelsen 2017 | Gambia | implementation unit | Lower River Region | 2014 | history of MDA | 383 | 3.7 | | 1.5 | post-MDA (no unusual epi) | no MDA warranted | SCR context: post-MDA |
| Hammou 2022 | Morocco | implementation unit | Agadez | 2019 | history of MDA | 1,246 | 0.2 (0 - 0.5) | | 2.6 | post-MDA (no unusual epi) | no MDA warranted | SCR context: post-validation surveillance |
| Hammou 2022 | Morocco | implementation unit | Boumalene Dades | 2019 | history of MDA | 1,121 | 0.1 (0 - 0.2) | | 2.7 | post-MDA (no unusual epi) | no MDA warranted | SCR context: post-validation surveillance |
| Burr 2019 | Malawi | implementation unit | Mchinji | 2014 | history of MDA | 3,355 | 6.4 (5.6 - 7.3) | 0.4 (0.2 - 0.7) | 2.1 | post-MDA (no unusual epi) | 1 round MDA | infection age range: 1-9 years, SCR context: post-MDA |
| Burr 2019 | Malawi | implementation unit | Chikwawa | 2014 | history of MDA | 2,720 | 5.1 (4.3 - 6) | 0.2 (0.1 - 0.4) | 2.8 | post-MDA (no unusual epi) | 1 round MDA | infection age range: 1-9 years, SCR context: post-MDA |
| Ngondi 2009 | Ethiopia | implementation unit | Dera | 2007 | history of MDA | 398 | 15 (10 - 20) | 1.1 (0.1 - 4) | | post-MDA (no unusual epi) | 3 rounds MDA | infection age range: 1-6 years |
| Ngondi 2009 | Ethiopia | implementation unit | Estie | 2007 | history of MDA | 382 | 46 (36 - 53) | 1.7 (0.3 - 4.8) | | post-MDA (no unusual epi) | 3 rounds MDA | infection age range: 1-6 years |
| Ngondi 2009 | Ethiopia | implementation unit | Huleteju Enese | 2007 | history of MDA | 409 | 36 (29 - 43) | 3.9 (1.6 - 7.8) | | post-MDA (no unusual epi) | 3 rounds MDA | infection age range: 1-6 years |
| Ngondi 2009 | Ethiopia | implementation unit | Enebsie Sarmedir | 2007 | history of MDA | 443 | 11 (8 - 15) | 4.4 (1.9 - 8.6) | | post-MDA (no unusual epi) | 3 rounds MDA | infection age range: 1-6 years |
| Ngondi 2009 | Ethiopia | implementation unit | Ebinat | 2007 | history of MDA | 416 | 54 (48 - 61) | 4.4 (1.9 - 8.6) | | post-MDA (no unusual epi) | 3 rounds MDA | infection age range: 1-6 years |
| Senyonjo 2018 | Ghana | implementation unit | Gushiegu | 2015 | no history of MDA | 1,367 | 0.7 (0.3 - 1.1) | 0 | | pre-MDA | no MDA warranted | infection age range: 1-9 years |
| Senyonjo 2018 | Ghana | implementation unit | Nadowli | 2015 | no history of MDA | 1,363 | 0.8 (0.3 - 1.5) | 0 | | pre-MDA | no MDA warranted | infection age range: 1-9 years |
| Gwyn 2021 | Togo | implementation unit | Keran | 2017 | no history of MDA | 1,511 | 0.4 (0.1 - 0.6) | | 0.7 | pre-MDA | no MDA warranted | SCR context: no history of MDA |
| Gwyn 2021 | Togo | implementation unit | Anie | 2017 | no history of MDA | 1,414 | 0.3 | | 1.0 | pre-MDA | no MDA warranted | SCR context: no history of MDA |
| Gwyn 2021 | Democratic Republic of the Congo | implementation unit | Nyemba | 2018 | no history of MDA | 1,578 | 1.1 (0.5 - 1.8) | | 1.1 | pre-MDA | no MDA warranted | SCR context: no history of MDA |
| Migchelsen 2017 | Gambia | implementation unit | Upper River Region | 2014 | no history of MDA | 359 | 3.1 | | 1.2 | pre-MDA | no MDA warranted | SCR context: no history of MDA |
| Gwyn 2021 | DRC | implementation unit | Manono | 2018 | no history of MDA | 1,591 | 7.3 (4.2 - 11.6) | | 10.4 | pre-MDA | 1 round MDA | SCR context: no history of MDA |
| Butcher 2020 | Kiribati | implementation unit | Tarawa | 2016 | no history of MDA | 1,059 | 38.2 (35.7 - 41.5) | 27.4 (24.7 - 30.1) | 19.7 | pre-MDA | 3 rounds MDA | infection age range: 1-9 years, SCR context: no history of MDA |
| Lynch 2022 2 | Nauru | implementation unit | whole country | 2019 | no history of MDA | 818 | 21.8 (15.2 - 26.2) | 34.5 (30.6 - 38.9) | 4.4 | pre-MDA | 3 rounds MDA | infection age range: 1-9 years, SCR context: no history of MDA |
| Cama 2017 | Kiribati | implementation unit | Kiritimati | 2015 | no history of MDA | 406 | 28 (24 - 35) | 27 | | pre-MDA | 3 rounds MDA | infection age range: 1-9 years |
| Gwyn 2021 | Ethiopia | implementation unit | Alefa | 2017 | history of MDA | 1,106 | 3.2 (1.4 - 5.7) | 0 | 0.4 | unusual epidemiology | no MDA warranted | infection age range: 1-5 years, SCR context: unusual epidemiology |
| Gwyn 2021 | Ethiopia | implementation unit | Woreta Town | 2017 | history of MDA | 532 | 2.7 (1.5 - 4.5) | 0 | 1.4 | unusual epidemiology | no MDA warranted | infection age range: 1-5 years, SCR context: unusual epidemiology |
| Nash 2018 1 | Ethiopia | implementation unit | Injibara | 2015 | history of MDA | 1,019 | 1.5 (0.4 - 4.9) | 0 | | unusual epidemiology | no MDA warranted | infection age range: 1-5 years |
| Nash 2022 | Ethiopia | implementation unit | Sedie | 2018 | history of MDA | 863 | 4.1 (2 - 6.8) | 0 | | unusual epidemiology | no MDA warranted | infection age range: 1-5 years |
| Butcher 2016 | Solomon Islands | implementation unit | Temotu, Rennell, Bellona | 2013 | no history of MDA | 1,135 | 0.26 | 1.3 | | unusual epidemiology | no MDA warranted | infection age range: 1-9 years |
| Cocks 2016/ Macleod 2016 | Fiji | implementation unit | Western Division | 2013 | no history of MDA | 1,009 | 2.8 (1.4 - 4.3) | 1.9 | | unusual epidemiology | no MDA warranted | infection age range: 1-9 years |
| Kasubi 2020 | Tanzania | implementation unit | Mpwapwa | 2017 | history of MDA | 1,000 | 9.5 (9.6 - 12.2) | 4.2 | | unusual epidemiology | 1 round MDA | infection age range: 1-9 years |
| Gwyn 2021 | Ethiopia | implementation unit | Dera | 2017 | history of MDA | 967 | 14.7 (10 - 20.5) | 0 | 2.6 | unusual epidemiology | 3 rounds MDA | infection age range: 1-5 years, SCR context: unusual epidemiology |
| Nash 2022 | Ethiopia | implementation unit | Enebessie Sar Midir | 2018 | history of MDA | 808 | 16.2 (11.7 - 22) | 0.3 (0 - 1) | | unusual epidemiology | 3 rounds MDA | infection age range: 1-5 years |
| Butcher 2020 | Vanuatu | combination of several implementation units | whole country | 2016 | no history of MDA | 1,112 | 16.5 (14.3 - 18.7) | 1.5 (0.8 - 2.2) | 1.8 | unusual epidemiology | 3 rounds MDA | infection age range: 1-9 years, SCR context: no history of MDA |
| Nash 2022 | Ethiopia | implementation unit | Enarge | 2018 | history of MDA | 589 | 16.9 (12.5 - 23.3) | 1.1 (0 - 2.5) | | unusual epidemiology | 3 rounds MDA | infection age range: 1-5 years |

S10. Table of studies used for programmatic decision-making

| Study ID (First author, year) | Country | Survey level | Area Surveyed | Year* | MDA status | Population tested (for TF) | TF prevalence (%)** | *Chlamydia trachomatis* infection prevalence (%)*** | SCR (per 100 person-years)^ | context | Typical programmatic action based on TF prevalence | Notes |
|---|---|---|---|---|---|---|---|---|---|---|---|---|
| Nash 2022 | Ethiopia | implementation unit | Huletu Eju Enessie | 2018 | history of MDA | 958 | 13.4 (10 - 17.7) | 1.3 (0 - 3.2) | | unusual epidemiology | 3 rounds MDA | infection age range: 1-5 years |
| Gwyn 2021 | Ethiopia | implementation unit | Andabet | 2017 | history of MDA | 824 | 37 (31.1 - 43.3) | 11.3 | 10.6 | unusual epidemiology | 3 rounds MDA | infection age range: 1-5 years, SCR context: unusual epidemiology |
| Nash 2017 | Ethiopia | combination of several implementation units | 16 districts in Amhara | 2012 | history of MDA | 17,124 | 18.1 (15.8 - 20.7) | 2.5 (0 - 5) | | unusual epidemiology | 3 rounds MDA | infection age range: 1-5 years |
| Nash 2018 2 | Ethiopia | combination of several implementation units | 10 zones in Amhara | 2011 | history of MDA | 68,959 | 26.2 (25.2 - 27.2) | 5.7 (4.2 - 7.3) | | unusual epidemiology | 3 rounds MDA | infection age range: 1-5 years |
| Nash 2022 | Ethiopia | implementation unit | Enemay | 2018 | history of MDA | 647 | 23.4 (17.8 - 29.7) | 3.8 (1.3 - 6.9) | | unusual epidemiology | 3 rounds MDA | infection age range: 1-5 years |
| Nash 2022 | Ethiopia | implementation unit | Shebel | 2018 | history of MDA | 743 | 22.9 (16.8 - 27.7) | 4 (1.4 - 7.3) | | unusual epidemiology | 3 rounds MDA | infection age range: 1-5 years |
| Macleod 2020 | Papua New Guinea | implementation unit | West New Britain | 2015 | no history of MDA | 1,000 | 11.4 | | 0.4 | unusual epidemiology | 3 rounds MDA | SCR context: unusual epidemiology |
| Macleod 2020 | Papua New Guinea | implementation unit | Western | 2015 | no history of MDA | 790 | 11.2 | | 1 | unusual epidemiology | 3 rounds MDA | SCR context: unusual epidemiology |
| Macleod 2020 | Papua New Guinea | implementation unit | Southern Highlands | 2015 | no history of MDA | 1,391 | 12.2 | | 1.6 | unusual epidemiology | 3 rounds MDA | SCR context: unusual epidemiology |

***Thresholds for infection: low: <1%, medium: 1-2%, high: ≥2%

**Thresholds for TF: low: <5%, medium: 5-9.9%, high: ≥10%

*Year of data collection, if known; otherwise, year of publication

^Thresholds for SCR depend on context: for areas that have no MDA history or have unusual epidemology, low: <=1.6, medium: 1.6-3.8, high: >=3.8; for areas post-MDA (including post-validation), low: <=2.2, medium: 2-2-4.5, high: >=4.5 seroconversions per 100 person-years

Cells appear as red, yellow, and green associated with thresholds pertaining to high, medium, and low categories (respectively), according to thresholds given above.

TF: trachomatous inflammation--follicuar, SCR: Seroconversion Rate, MDA: Mass Drug Administration
