# Supplementary material for: Comparison of Methods for Assessing Chlamydia trachomatis Transmission Intensity: A Systematic Review
Source: Am J Trop Med Hyg. 2026 Sep 22;115(5):903–22. doi: 10.4269/ajtmh.25-0541 (PMC13615270; doi:10.4269/ajtmh.25-0541)
Supplement: Supplementary file 3 [file tpmd250541.SD11.pdf]

S11. TF vs. Infection, TF vs. SCR in subset of populations with all three indicators

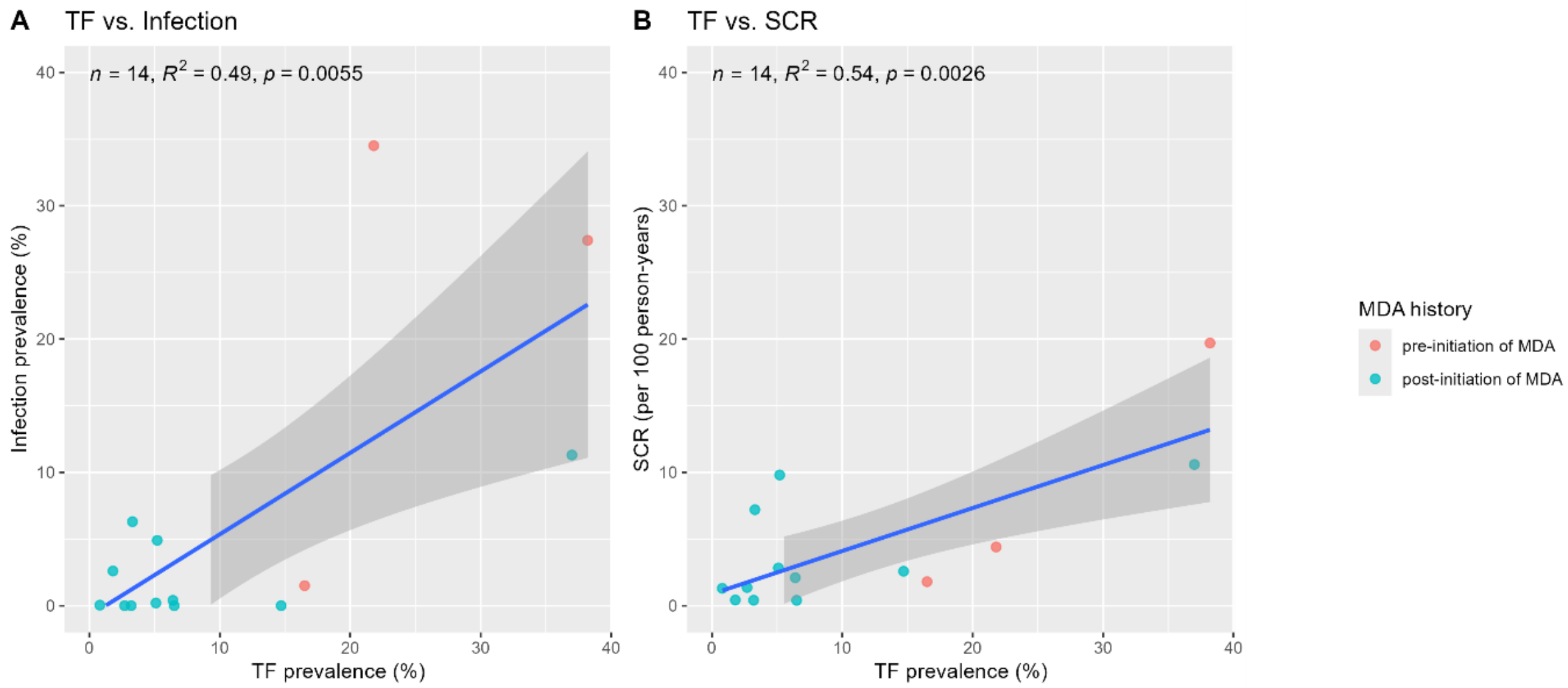

TF: trachomatous inflammation—follicular in children 1–9, SCR: seroconversion rate, MDA: mass drug administration
