# Supplementary material for: Comparison of Methods for Assessing Chlamydia trachomatis Transmission Intensity: A Systematic Review
Source: Am J Trop Med Hyg. 2026 Sep 22;115(5):903–22. doi: 10.4269/ajtmh.25-0541 (PMC13615270; doi:10.4269/ajtmh.25-0541)
Supplement: Supplementary file 7 [file tpmd250541.SD5.pdf]

S5. Publication year for missing full texts

Analysis of publication year for full text articles that could not be found (n=93)

Year of publication	Count of Published Year	running total	running percent
**1987**	1	1	1%
**1988**	3	4	4%
**1989**	6	10	11%
**1990**	6	16	17%
**1991**	5	21	23%
**1992**	12	33	35%
**1993**	2	35	38%
**1994**	3	38	41%
**1995**	4	42	45%
**1996**	3	45	48%
**1997**	5	50	54%
**1999**	3	53	57%
**2000**	2	55	59%
**2001**	4	59	63%
**2003**	1	60	65%
**2004**	2	62	67%
**2005**	1	63	68%
**2007**	4	67	72%
**2009**	3	70	75%
**2010**	1	71	76%
**2011**	2	73	78%
**2012**	1	74	80%
**2013**	4	78	84%
**2014**	3	81	87%
**2015**	1	82	88%
**2016**	4	86	92%
**2017**	3	89	96%
**2018**	1	90	97%
**2019**	1	91	98%
**2020**	2	93	100%
