# Supplementary material for: Comparison of Methods for Assessing Chlamydia trachomatis Transmission Intensity: A Systematic Review
Source: Am J Trop Med Hyg. 2026 Sep 22;115(5):903–22. doi: 10.4269/ajtmh.25-0541 (PMC13615270; doi:10.4269/ajtmh.25-0541)
Supplement: Supplementary file 8 [file tpmd250541.SD6.pdf]

S6. Table of included studies

| | | | | | | | | | | Risk of Bias checklist items** | | | | | | | | | | | | | | | | | | | | | | |
|---|---|---|---|---|---|---|---|---|---|---|---|---|---|---|---|---|---|---|---|---|---|---|---|---|---|---|---|---|---|---|---|---|
| reference | Country or countries of data collection | Type of study | TF or FPC | other clinical indicators? | photo-graded TF or FPC? | infection? | serology? | SCR? | Critical Appraisal Tool used** | Prevalence study: Was the sample frame appropriate to address the target pop? | Prevalence study: Were study participants sampled in an appropriate way? | Prevalence study: Was the sample size adequate? | Prevalence study: Were the study subjects and setting described in detail? | Prevalence study: Was the data analysis conducted with sufficient coverage of the identified sample? | Prevalence study: Were valid methods used for the identification of the condition(s)? | Prevalence study: Was the condition measured in a standard, reliable way for all participants? | Prevalence study: Was there appropriate statistical analysis? | Prevalence study: Was the response rate adequate, and if not, was the low response rate managed appropriately? | DTA study: Was a consecutive or random sample of patients enrolled? | DTA study: Was a case-control design avoided? | DTA study: Did the study avoid inappropriate exclusions? | DTA: Were the index test results interpreted without knowledge of the results of the reference standard? | DTA: If a threshold was used, was it pre-specified? | DTA: Is the reference standard likely to correctly classify the target condition? | DTA: Were the reference standard results interpreted without knowledge of the results of the index test? | DTA: Was there an appropriate interval between index test and reference standard? | DTA: Did all patients receive the same reference standard? | DTA: Were all patients included in the analysis? | Prevalence study score [meets criteria/total] (%) | DTA study score [meets criteria/total] (%) | Max score (if both checklists used, higher of 2 scores) |
| Abdilwohab, M. G.; Abebo, Z. H.High prevalence of clinically active trachoma and its associated risk factors among preschool-aged children in arba minch health and demographic surveillance site, southern ethiopia. 2020. Clinical Ophthalmology. , volume: 14, issue: , pages: 3709-3718. Doi: https://dx.doi.org/10.2147/OPTH.S282567. | Ethiopia | Cross-sectional | TF | TI | no | no | no | no | Checklist for Prevalence Studies | Yes | Yes | Yes | Yes | Yes | Yes | Yes | Yes | Yes | | | | | | | | | | | [9/9] (100%) | NA | 100% |
| Abdou, A.; Nassirou, B.; Kadri, B.; Moussa, F.; Munoz, B. E.; Opong, E.; West, S. K.Prevalence and risk factors for trachoma and ocular Chlamydia trachomatis infection in Niger. 2007. British Journal of Ophthalmology. , volume: 91, issue: 1, pages: 13-17. Doi: http://dx.doi.org/10.1136/bjo.2006.099507. | Niger | Cross-sectional | TF | TI | no | yes | no | no | Checklist for Prevalence Studies | Yes | Yes | Yes | No | Yes | Yes | Yes | Yes | Yes | | | | | | | | | | | [8/9] (89%) | NA | 89% |
| Abebo, T. A.; Tesfaye, D. J.Prevalence and distribution of active trachoma among children 1-9 years old at Leku town, Southern Ethiopia. 2017. Current Pediatric Research. , volume: 21(3), issue: , pages: 507-513. Doi: . | Ethiopia | Cross-sectional | TF | TI | no | no | no | no | Checklist for Prevalence Studies | Yes | Yes | Yes | Yes | Yes | Yes | Yes | Yes | Yes | | | | | | | | | | | [9/9] (100%) | NA | 100% |
| Yilikal, Adamu; Semira, FerejiPrevalence of active trachoma among children between 1-9 years, in Woliso town, central Ethiopia. 2018. Ethiopian Journal of Health Development. , volume: 32, issue: 2, pages: unpaginated. Doi: . | Ethiopia | Cross-sectional | TF | TI, TF and/or TI | no | no | no | no | Checklist for Prevalence Studies | Yes | Yes | Yes | Yes | Unclear | Yes | Yes | Yes | Unclear | | | | | | | | | | | [7/9] (78%) | NA | 78% |
| Tesfay, Haileselassie; Samson, BayuAltitude - a risk factor for active trachoma in southern Ethiopia. 2007. Ethiopian Medical Journal. , volume: 45, issue: 2, pages: 181-186. Doi: . | Ethiopia | Cross-sectional | TF | TI | no | no | no | no | Checklist for Prevalence Studies | Yes | Yes | No | Yes | Yes | Yes | Yes | Yes | Yes | | | | | | | | | | | [8/9] (89%) | NA | 89% |
| Al-Arab, G. E.; Tawfik, N.; El-Gendy, R.; Anwar, W.; Courtright, P.The burden of trachoma in the rural Nile Delta of Egypt: a survey of Menofiya governorate. 2001. British Journal of Ophthalmology. , volume: 85, issue: 12, pages: 1406-1410. Doi: http://dx.doi.org/10.1136/bjo.85.12.1406. | Egypt | Cross-sectional | TF | TI | no | no | no | no | Checklist for Prevalence Studies | Yes | Yes | Yes | No | Yes | Yes | Yes | Yes | Yes | | | | | | | | | | | [8/9] (89%) | NA | 89% |
| Wondu, Alemayehu; Muluken, Melese; Fredlander, E.; Alemayehu, Worku; Courtright, P.Active trachoma in children in central Ethiopia: association with altitude. 2005. Transactions of the Royal Society of Tropical Medicine and Hygiene. , volume: 99, issue: 11, pages: 840-843. Doi: http://dx.doi.org/10.1016/j.trstmh.2005.06.013. | Ethiopia | Cross-sectional | TF | TI, TF and TI, TF and/or TI | no | no | no | no | Checklist for Prevalence Studies | Yes | Yes | Yes | Yes | Yes | Yes | Yes | Yes | Yes | | | | | | | | | | | [9/9] (100%) | NA | 100% |
| Amer, K.; Muller, A.; Abdelhafiz, H. M.; Al-Khatib, T.; Bakhtiari, A.; Boisson, S.; El-Arab, G. E.; Gad, H.; Gordon, B. A.; Madian, A.; Mahanna, A. T.; Mokhtar, S.; Safa, O. H.; Samy, M.; Shalaby, M.; Taha, Z. A.; Willis, R.; Yacoub, A.; Mamdouh, A. R.; Younis, A. K.; Zoheir, M. B. E.; Courtright, P.; Solomon, A. W.Prevalence of trachoma in four marakez of Elmenia and Bani Suef Governorates, Egypt. 2018. Special Issue: 2017 Global Trachoma Mapping Project.. , volume: 25, issue: Suppl. 1, pages: 70-78. Doi: http://dx.doi.org/10.1080/09286586.2018.1446536. | Egypt | Cross-sectional | TF | TI | no | no | no | no | Checklist for Prevalence Studies | Yes | Yes | Yes | No | Yes | Yes | Yes | Yes | Yes | | | | | | | | | | | [8/9] (89%) | NA | 89% |
| Amza, A.; Goldschmidt, P.; Einterz, E.; Huguet, P.; Olmiere, C.; Bensaid, P.; Bella-Assumpta, L.Elimination of active trachoma after two topical mass treatments with azithromycin 1.5% eye drops. 2010. PLoS Neglected Tropical Diseases. , volume: 4, issue: 11, pages: e895. Doi: http://dx.doi.org/10.1371/journal.pntd.0000895. | Cameroon | Cross-sectional | TF | TF and/or TI | no | no | no | no | Checklist for Prevalence Studies | Yes | Yes | Yes | Yes | Yes | Yes | Yes | Yes | Yes | | | | | | | | | | | [9/9] (100%) | NA | 100% |
| Amza, A.; Kadri, B.; Nassirou, B.; Stoller, N. E.; Yu, S. N.; Zhou, Z.; Chin, S.; West, S. K.; Bailey, R. L.; Mabey, D. C. W.; Keenan, J. D.; Porco, T. C.; Lietman, T. M.; Gaynor, B. D.Community risk factors for ocular chlamydia infection in Niger: Pre-treatment results from a cluster-randomized trachoma trial. 2012. PLoS Neglected Tropical Diseases. , volume: 6(4) (no pagination), issue: , pages: . Doi: https://dx.doi.org/10.1371/journal.pntd.0001586. | Niger | Cross-sectional | TF | TF and/or TI | no | yes | no | no | Checklist for Prevalence Studies | Yes | Yes | Yes | Yes | Yes | Yes | Yes | Yes | Yes | | | | | | | | | | | [9/9] (100%) | NA | 100% |
| Amza, A.; Kadri, B.; Nassirou, B.; Yu, S. N.; Stoller, N. E.; Bhosai, S. J.; Zhou, Z.; McCulloch, C. E.; West, S. K.; Bailey, R. L.; Keenan, J. D.; Lietman, T. M.; Gaynor, B. D.The Easiest Children to Reach Are Most Likely to Be Infected with Ocular Chlamydia trachomatis in Trachoma Endemic Areas of Niger. 2013. PLoS Neglected Tropical Diseases. , volume: 7(1) (no pagination), issue: , pages: . Doi: https://dx.doi.org/10.1371/journal.pntd.0001983. | Niger | Cross-sectional | TF | no | no | yes | no | no | Checklist for Prevalence Studies | Yes | Yes | Unclear | No | Unclear | Yes | Yes | Yes | Unclear | | | | | | | | | | | [5/9] (56%) | NA | 56% |
| Amza, A.; Kadri, B.; Nassirou, B.; Cotter, S. Y.; Stoller, N. E.; Zhou, ZhaoXia; Bailey, R. L.; Mabey, D. C.; Porco, T. C.; Keenan, J. D.; Gaynor, B. D.; West, S. K.; Lietman, T. M.A cluster-randomized trial to assess the efficacy of targeting trachoma treatment to children. 2017. Clinical Infectious Diseases. , volume: 64, issue: 6, pages: 743-750. Doi: . | Niger | Cross-sectional within RCT | TF | TI | no | yes | no | no | Checklist for Prevalence Studies | Yes | Yes | Unclear | Yes | Unclear | Yes | Yes | Yes | Unclear | | | | | | | | | | | [6/9] (67%) | NA | 67% |
| Amza, A.; Kadri, B.; Nassirou, B.; Cotter, S. Y.; Stoller, N. E.; West, S. K.; Bailey, R. L.; Porco, T. C.; Gaynor, B. D.; Keenan, J. D.; Lietman, T. M.; Oldenburg, C. E.Effectiveness of expanding annual mass azithromycin distribution treatment coverage for trachoma in Niger: a cluster randomised trial. 2018. British Journal of Ophthalmology. , volume: 102, issue: 5, pages: 680-686. Doi: http://dx.doi.org/10.1136/bjophthalmol-2017-310916. | Niger | Cross-sectional within RCT | TF | no | no | yes | no | no | Checklist for Prevalence Studies | Yes | Yes | Unclear | Yes | Unclear | Yes | Yes | Yes | Unclear | | | | | | | | | | | [6/9] (67%) | NA | 67% |
| Amza, A.; Kadri, B.; Nassirou, B.; Cotter, S. Y.; Stoller, N. E.; West, S. K.; Bailey, R. L.; Porco, T. C.; Keenan, J. D.; Lietman, T. M.; Oldenburg, C. E.Community-level association between clinical trachoma and ocular chlamydia infection after mass azithromycin distribution in a mesoendemic region of Niger. 2019. Ophthalmic Epidemiology. , volume: 26, issue: 4, pages: 231-237. Doi: http://dx.doi.org/10.1080/09286586.2019.1597129. | Niger | Cross-sectional within RCT | TF | TI | no | yes | no | no | Checklist for Prevalence Studies | Yes | Yes | Unclear | Yes | Unclear | Yes | Yes | Yes | Unclear | | | | | | | | | | | [6/9] (67%) | NA | 67% |
| Astle, W. F.; Wiafe, B.; Ingram, A. D.; Mwanga, M.; Glassco, C. B.Trachoma control in Southern Zambia - an international team project employing the SAFE strategy. 2006. Ophthalmic Epidemiology. , volume: 13, issue: 4, pages: 227-236. Doi: http://dx.doi.org/10.1080/09286580600718974. | Zambia | Cross-sectional | TF | TI | no | no | no | no | Checklist for Prevalence Studies | Yes | Unclear, Not sure how villages were chosen for inclusion | Unclear | Yes | Unclear | Yes | Yes | Yes | Unclear | | | | | | | | | | | [5/9] (56%) | NA | 56% |
| Ayele, B.; Gebre, T.; Moncada, J.; House, J. I.; Stoller, N. E.; Zhou, Z.; Porco, T. C.; Gaynor, B. D.; Emerson, P. M.; Schachter, J.; Keenan, J. D.Risk factors for ocular chlamydia after three mass azithromycin distributions. 2011. PLoS Neglected Tropical Diseases. , volume: 5(12) (no pagination), issue: , pages: . Doi: https://dx.doi.org/10.1371/journal.pntd.0001441. | Ethiopia | Cross-sectional | TF | TI, TF and/or TI | no | yes | no | no | Checklist for Prevalence Studies | Yes | Yes | Yes | Yes | Yes | Yes | Yes | Yes | Yes | | | | | | | | | | | [9/9] (100%) | NA | 100% |
| Ayelgn, K.; Guadu, T.; Getachew, A.Low prevalence of active trachoma and associated factors among children aged 1-9 years in rural communities of Metema District, Northwest Ethiopia: a community based cross-sectional study. 2021. Italian Journal of Pediatrics. , volume: 47(1) (no pagination), issue: , pages: . Doi: https://dx.doi.org/10.1186/s13052-021-01064-x. | Ethiopia | Cross-sectional | TF | TI | no | no | no | no | Checklist for Prevalence Studies | Yes | Yes | Yes | No | Yes | Yes | Yes | Yes | Yes | | | | | | | | | | | [8/9] (89%) | NA | 89% |
| Ayena, K. D.; Amza, A.; Agbo, Y. M.; Dorkenoo, M.; Azoumah, K. D.; Dogbe, K. S.; Sognikin, K. S.; Komlagan, S. A.; Mathieu, E.; Bronzan, R.; Anthony, K. K.; Sodahlon, Y. K.; Banla, M. J.; Balo, K. P.[What do we know about trachoma in the economically weakest infantile population of Kara in Togo?]. [French]. 2010. Bulletin de la Societe belge d'ophtalmologie. , volume: , issue: 316, pages: 37-42. Doi: . | Togo | Cross-sectional | TF | TI | no | no | no | no | Checklist for Prevalence Studies | Yes | Unclear how villages/children chosen and if this was random. | Unclear | Yes | Unclear | Yes | Yes | Yes | Unclear | | | | | | | | | | | [5/9] (56%) | NA | 56% |
| Ayena, K. D.; Dzidzinyo, K.; Koffi, K. S.; Salou, M.; Amza, A.; Amedome, K. M.; Balo, K. P.[Trachoma rapid assessment in the infantile population of Togo]. [French]. 2011. Medecine tropicale : revue du Corps de sante colonial. , volume: 71(5), issue: , pages: 515-516. Doi: . | Togo | Cross-sectional | TF | TI | no | no | no | no | Checklist for Prevalence Studies | Yes | Unclear how villages/children chosen and if this was random. | Unclear | No | Unclear | Yes | Yes | Yes | Unclear | | | | | | | | | | | [4/9] (44%) | NA | 44% |
| Babalola, O. E.; Abiose, A.; Barrie, J. R.The problem of trachoma within an onchocerciasis-endemic zone in Kaduna: need for a multilateral approach?. 2005. Ophthalmic Epidemiology. , volume: 12, issue: 5, pages: 311-9. Doi: . | Nigeria | Cross-sectional within RCT | TF | TI | no | no | no | no | Checklist for Prevalence Studies | Yes | Unclear, villages chosen on basis of proximity to black fly breeding sites. | Unclear | No | Yes | Yes | Yes | Yes | Yes | | | | | | | | | | | [6/9] (67%) | NA | 67% |
| Bailey, R. L.; Hampton, T. J.; Hayes, L. J.; Ward, M. E.; Whittle, H. C.; Mabey, D. C. W.Polymerase chain reaction for the detection of ocular chlamydial infection in trachoma-endemic communities. 1994. Journal of Infectious Diseases. , volume: 170, issue: 3, pages: 709-712. Doi: http://dx.doi.org/10.1093/infdis/170.3.709. | Gambia | Cross-sectional | any follicles | no | no | yes | no | no | Checklist for Prevalence Studies | Yes | Yes | Yes | No | Yes | Yes | Yes | Yes | Yes | | | | | | | | | | | [8/9] (89%) | NA | 89% |
| Bamani, S.; Dembele, M.; Sankara, D.; Coulibaly, F.; Kamissoko, Y.; Ting, J.; Rotondo, L. A.; Emerson, P. M.; King, J. D.Evaluation of the prevalence of trachoma 12 years after baseline surveys in Kidal Region, Mali. 2010. Tropical Medicine and International Health. , volume: 15, issue: 3, pages: 306-311. Doi: http://dx.doi.org/10.1111/j.1365-3156.2009.02459.x. | Mali | Cross-sectional | TF | TI | no | no | no | no | Checklist for Prevalence Studies | Yes | Yes | Yes | Yes | Yes | Yes | Yes | Yes | Yes | | | | | | | | | | | [9/9] (100%) | NA | 100% |
| Bamani, S.; King, J. D.; Dembele, M.; Coulibaly, F.; Sankara, D.; Kamissoko, Y.; Ting, J.; Rotondo, L. A.; Emerson, P. M.Where do we go from here? Prevalence of trachoma three years after stopping mass distribution of antibiotics in the regions of Kayes and Koulikoro, Mali. 2010. PLoS Neglected Tropical Diseases. , volume: 4, issue: 7, pages: e734. Doi: http://dx.doi.org/10.1371/journal.pntd.0000734. | Mali | Cross-sectional | TF | TI | no | no | no | no | Checklist for Prevalence Studies | Yes | Yes | Yes | Yes | Unclear | Yes | Yes | Yes | Unclear | | | | | | | | | | | [7/9] (78%) | NA | 78% |
| Bejiga, A.; Alemayehu, W.Prevalence of trachoma and its determinants in Dalocha District, Central Ethiopia. 2001. Ophthalmic Epidemiology. , volume: 8, issue: 44960, pages: 119-25. Doi: . | Ethiopia | Cross-sectional | TF | TI | no | no | no | no | Checklist for Prevalence Studies | Yes | Yes | Yes | Yes | Yes | Yes | Yes | Yes | Yes | | | | | | | | | | | [9/9] (100%) | NA | 100% |
| Bella, A. L.; Einterz, E.; Huguet, P.; Bensaid, P.; Amza, A.; Renault, D.Effectiveness and safety of azithromycin 1.5% eye drops for mass treatment of active trachoma in a highly endemic district in Cameroon. 2020. BMJ Open Ophthalmology. , volume: 5, issue: 1, pages: . Doi: 10.1136/bmjophth-2020-000531. | Cameroon | Cross-sectional | TF | TI | no | no | no | no | Checklist for Prevalence Studies | Yes | Yes | Yes | Yes | Yes | Yes | Yes | Yes | Yes | | | | | | | | | | | [9/9] (100%) | NA | 100% |

S6. Table of included studies

| | | | | | | | | | | Risk of Bias checklist items** | | | | | | | | | | | | | | | | | | | | | |
|---|---|---|---|---|---|---|---|---|---|---|---|---|---|---|---|---|---|---|---|---|---|---|---|---|---|---|---|---|---|---|---|
| reference | Country or countries of data collection | Type of study | TF or FPC | other clinical indicators? | photo-graded TF or FPC? | infection? | serology? | SCR? | Critical Appraisal Tool used** | Prevalence study: Was the sample frame appropriate to address the target pop? | Prevalence study: Were study participants sampled in an appropriate way? | Prevalence study: Was the sample size adequate? | Prevalence study: Were the study subjects and setting described in detail? | Prevalence study: Was the data analysis conducted with sufficient coverage of the identified sample? | Prevalence study: Were valid methods used for the identification of the condition(s)? | Prevalence study: Was the condition measured in a standard, reliable way for all participants? | Prevalence study: Was there appropriate statistical analysis? | Prevalence study: Was the response rate adequate, and if not, was the low response rate managed appropriately? | DTA study: Was a consecutive or random sample of patients enrolled? | DTA study: Was a case-control design avoided? | DTA study: Did the study avoid inappropriate exclusions? | DTA: Were the index test results interpreted without knowledge of the results of the reference standard? | DTA: If a threshold was used, was it pre-specified? | DTA: Is the reference standard likely to correctly classify the target condition? | DTA: Were the reference standard results interpreted without knowledge of the results of the index test? | DTA: Was there an appropriate interval between index test and reference standard? | DTA: Did all patients receive the same reference standard? | DTA: Were all patients included in the analysis? | Prevalence study score [meets criteria/total] (%) | DTA study score [meets criteria/total] (%) | Max score (if both checklists used, higher of 2 scores) |
| Yemane, Berhane; Alemayehu, Worku; Abebe, Bejiga; Liknaw, Adamu; Wondu, Alemayehu; Amir, Bedri; Zegeye, Haile; Allehone, Ayalew; Yilkal, Adamu; Teshome, Gebre; Kebede, T. D.; West, E.; West, S.Prevalence of trachoma in Ethiopia. 2007. Ethiopian Journal of Health Development. , volume: 21, issue: 3, pages: 211-215. Doi: . | Ethiopia | Cross-sectional | TF | TI, TF and/or TI | no | no | no | no | Checklist for Prevalence Studies | Yes | Yes | Unclear | No | Unclear | Yes | Yes | Yes | Unclear | | | | | | | | | | | [5/9] (56%) | NA | 56% |
| Bhosai, S. J.; Amza, A.; Beido, N.; Bailey, R. L.; Keenan, J. D.; Gaynor, B. D.; Lietman, T. M.Application of smartphone cameras for detecting clinically active trachoma. 2012. British Journal of Ophthalmology. , volume: 96, issue: 10, pages: 1350-1351. Doi: 10.1136/bjophthalmol-2012-302050. | Niger | Cross-sectional | TF | TI | yes | no | no | no | Both | Yes | Yes | Yes | Yes | Yes | Yes | Yes | Unclear | Unclear | Random | Yes | Yes | Yes | Yes | Unclear - LCA used | Yes | Yes | Yes | Yes | [7/9] (78%) | [8/10] (80%) | 80% |
| Bid, R.; Sandi, F.; Goodhew, B.; Massae, P.; Alexander, N.; Laswai, A.; Makupa, W.; Molina, S.; Holland, M.; Mabey, D.; Lammie, P. J.; Solomon, A.; Martin, D.Piloting serological tools for trachoma postelimination surveillance. 2013. American Journal of Tropical Medicine and Hygiene. , volume: 1), issue: , pages: 12. Doi: . | Tanzania | Cross-sectional | TF | TI | no | no | no | no | Checklist for Prevalence Studies | Unclear | Unclear | Unclear | No | Unclear | Unclear | Unclear | Unclear | Unclear | | | | | | | | | | | [0/9] (0%) | NA | 0% |
| Bio, A. A.; Boko, P. M.; Dossou, Y. A.; Tougoue, J. J.; Kabore, A.; Sounouvou, I.; Biaou, J. E.; Nizigama, L.; Courtright, P.; Solomon, A. W.; Rotondo, L.; Batcho, W.; Kinde-Gazard, D.Prevalence of trachoma in northern Benin: results from 11 population-based prevalence surveys covering 26 districts. 2017. Ophthalmic Epidemiology. , volume: 24, issue: 4, pages: 265-273. Doi: http://dx.doi.org/10.1080/09286586.2017.1279337. | Benin | Cross-sectional | TF | TI | no | no | no | no | Checklist for Prevalence Studies | Yes | Yes | Yes | No | Yes | Yes | Yes | Yes | Yes | | | | | | | | | | | [8/9] (89%) | NA | 89% |
| Bobo, L.; Munoz, B.; Viscidi, R.; Quinn, T.; Mkocha, H.; West, S.Diagnosis of Chlamydia trachomatis eye infection in Tanzania by polymerase chain reaction/enzyme immunoassay. 1991. Lancet. , volume: 338, issue: Oct. 5, pages: 847-850. Doi: . | Tanzania | Cross-sectional | TF | TI | no | yes | no | no | Checklist for Prevalence Studies | Yes | Yes | Unclear | No | Unclear | Yes | Yes | Yes | Unclear | | | | | | | | | | | [5/9] (56%) | NA | 56% |
| Brady, C. J.; Wolle, M. A.; Naufal, F.; Mkocha, H.; West, S. K.Crowdsourcing can match field grading validity for follicular trachoma. 2021. Investigative Ophthalmology and Visual Science. Conference: Annual Meeting Association for Research in Vision and Ophthalmology, ARVO. , volume: 62, issue: 8, pages: . Doi: . | Tanzania | Cross-sectional | TF | no | yes | no | no | no | Both | Unclear | Unclear | Unclear | Unclear | Unclear | Unclear | Unclear | Unclear | Unclear | Unclear | Unclear | Unclear | Unclear | Unclear | Unclear | Unclear | Unclear | Unclear | Unclear | [0/9] (0%) | [0/10] (0%) | 0% |
| Burr, S. E.; Hart, J. D.; Edwards, T.; Baldeh, I.; Bojang, E.; Harding-Esch, E. M.; Holland, M. J.; Lietman, T. M.; West, S. K.; Mabey, D. C. W.; Sillah, A.; Bailey, R. L.Association between ocular bacterial carriage and follicular trachoma following mass azithromycin distribution in The Gambia. 2013. PLoS Neglected Tropical Diseases. , volume: 7, issue: 7, pages: e2347. Doi: http://dx.doi.org/10.1371/journal.pntd.0002347. | Gambia | Cross-sectional | TF | no | no | yes | no | no | Both | Yes | Yes | Yes | Yes | Yes | Yes | Yes | Yes | Yes | Random | Yes | Yes | Yes | Yes | Yes | Yes | Yes | Yes | Yes | [9/9] (100%) | [9/10] (90%) | 100% |
| Burr, S. E.; Hart, J.; Samikwa, L.; Chaima, D.; Cooley, G.; Martin, D.; Masika, M.; Solomon, A. W.; Bailey, R. L.; Kalua, K.Pgp3 seroprevalence and associations with active trachoma and ocular Chlamydia trachomatis infection in Malawi: cross-sectional surveys in six evaluation units. 2019. PLoS Neglected Tropical Diseases. , volume: 13, issue: 10, pages: e0007749. Doi: http://dx.doi.org/10.1371/journal.pntd.0007749. | Malawi | Cross-sectional | TF | no | no | yes | yes | yes | Both | Yes | Yes | Yes | No | Unclear | Yes | Yes | Yes | Unclear | Random | Yes | Yes | Yes | Yes | Yes | Yes | Yes | Yes | Yes | [6/9] (67%) | [9/10] (90%) | 90% |
| Burton, M. J.; Holland, M. J.; Makalo, P.; Aryee, E. A. N.; Sillah, A.; Cohuet, S.; Natividad, A.; Alexander, N. D. E.; Mabey, D. C. W.; Bailey, R. L.Profound and sustained reduction in Chlamydia trachomatis in the Gambia: a five-year longitudinal study of trachoma endemic communities. 2010. PLoS Neglected Tropical Diseases. , volume: 4, issue: 10, pages: e835. Doi: http://dx.doi.org/10.1371/journal.pntd.0000835. | Gambia | Cross-sectional within longitudinal | TF | no | no | yes | no | no | Checklist for Prevalence Studies | Yes | Yes | Unclear | No | Yes | Yes | Yes | Yes | Yes | | | | | | | | | | | [7/9] (78%) | NA | 78% |
| Burton, M. J.; Ramadhani, A.; Weiss, H. A.; Hu, V.; Massae, P.; Burr, S. E.; Shangali, W.; Holland, M. J.; Mabey, D. C. W.; Bailey, R. L.Active trachoma is associated with increased conjunctival expression of IL17A and profibrotic cytokines. 2011. Infection and Immunity. , volume: 79, issue: 12, pages: 4977-4983. Doi: http://dx.doi.org/10.1128/IAI.05718-11. | Tanzania | Cross-sectional | TF | TI | no | yes | no | no | Both | Yes | Yes | Unclear | No | Yes | Yes | Yes | Yes | Yes | Unclear how village chosen | Yes | Yes | Unclear | Yes | Yes | Yes | Yes | Yes | Yes | [7/9] (78%) | [8/10] (80%) | 80% |
| Butcher, R. M. R.; Sokana, O.; Jack, K.; Macleod, C. K.; Marks, M. E.; Kalae, E.; Sui, L.; Russell, C.; Tutill, H. J.; Williams, R. J.; Breuer, J.; Willis, R.; Mesurier, R. T. le; Mabey, D. C. W.; Solomon, A. W.; Roberts, C. H.Low prevalence of conjunctival infection with Chlamydia trachomatis in a treatment-naive trachoma-endemic region of the Solomon Islands. 2016. PLoS Neglected Tropical Diseases. , volume: 10, issue: 9, pages: e0004863. Doi: . | Solomon Islands | Cross-sectional | TF | TI | no | yes | no | no | Checklist for Prevalence Studies | Yes | Yes | Yes | Yes | Yes | Yes | Yes | Yes | Yes | | | | | | | | | | | [9/9] (100%) | NA | 100% |
| Butcher, R.Using alternate indicators to define need for public health intervention for trachoma: Evidence from the Pacific Islands. 2017. , volume: , issue: , pages: . Doi: . | Fiji | Cross-sectional | TF | TI | no | no | no | no | Checklist for Prevalence Studies | Yes | Yes | Yes | Yes | Unclear | Yes | Yes | Yes | Unclear | | | | | | | | | | | [7/9] (78%) | NA | 78% |
| Butcher, R.; Sokana, O.; Jack, K.; Sui, L.; Russell, C.; Last, A.; Martin, D. L.; Burton, M. J.; Solomon, A. W.; Mabey, D. C. W.; Roberts, C. H.Clinical signs of trachoma are prevalent among Solomon Islanders who have no persistent markers of prior infection with Chlamydia trachomatis [version 1; referees: 2 approved]. 2018. Wellcome Open Research. , volume: 3, issue: , pages: . Doi: 10.12688/wellcomeopenres.13423.1. | Solomon Islands | Cross-sectional | TF | TI | no | yes | yes | no | Both | Unclear; only villages with previous TF rates >10% were chosen | Yes | Yes | Yes | Yes | Yes | Yes | Yes | Yes | Random | Yes | Yes | Yes | Yes | Yes | Yes | Yes | Yes | Yes | [8/9] (89%) | [9/10] (90%) | 90% |
| Butcher, R.; Handley, B.; Garae, M.; Taoaba, R.; Pickering, H.; Bong, A.; Sokana, O.; Burton, M. J.; Sepulveda, N.; Cama, A.; Mesurier, R. le; Solomon, A. W.; Mabey, D.; Taleo, F.; Tekeraoi, R.; Roberts, C. H.Ocular Chlamydia trachomatis infection, anti-Pgp3 antibodies and conjunctival scarring in Vanuatu and Tarawa, Kiribati before antibiotic treatment for trachoma. 2020. Journal of Infection. , volume: 80, issue: 4, pages: 454-461. Doi: http://dx.doi.org/10.1016/j.jinf.2020.01.015. | Kiribati, Vanuatu | Cross-sectional | TF | TI | no | yes | yes | yes | Both | Yes | Yes | Yes | Yes | Yes | Yes | Yes | Yes | Yes | Random | Yes | Yes | Yes | Yes | Yes | Yes | Yes | Yes | Yes | [9/9] (100%) | [9/10] (90%) | 100% |
| Abreu Caligaris, L. S.; Miyake Morimoto, W. T.; Medina, N. H.; Waldman, E. A.Trachoma prevalence and risk factors among preschool children in a central area of the city of Sao Paulo, Brazil. 2006. Ophthalmic Epidemiology. , volume: 13(6), issue: , pages: 365-370. Doi: https://dx.doi.org/10.1080/09286580601013078. | Brazil | Cross-sectional | TF | TI | no | no | no | no | Checklist for Prevalence Studies | Yes | Yes | Yes | No | Yes | Yes | Yes | Yes | Yes | | | | | | | | | | | [8/9] (89%) | NA | 89% |
| Cama, A.; Muller, A.; Taoaba, R.; Butcher, R. M. R.; Itibita, I.; Migchelsen, S. J.; Kiauea, T.; Pickering, H.; Willis, R.; Roberts, C. H.; Bakhtiari, A.; Mesurier, R. T. le; Alexander, N. D. E.; Martin, D. L.; Tekeraoi, R.; Solomon, A. W.Prevalence of signs of trachoma, ocular Chlamydia trachomatis infection and antibodies to Pgp3 in residents of Kiritimati Island, Kiribati. 2017. PLoS Neglected Tropical Diseases. , volume: 11, issue: 9, pages: e0005863. Doi: http://dx.doi.org/10.1371/journal.pntd.0005863. | Kiribati | Cross-sectional | TF | TI | no | yes | yes | no | Checklist for Prevalence Studies | Yes | Yes | Yes | No | Yes | Yes | Yes | Yes | Yes | | | | | | | | | | | [8/9] (89%) | NA | 89% |
| Chen, X.; Munoz, B.; Mkocha, H.; Gaydos, C. A.; Dize, L.; Quinn, T. C.; West, S. K.Risk of seroconversion and seroreversion of antibodies to Chlamydia trachomatis pgp3 in a longitudinal cohort of children in a low trachoma prevalence district in Tanzania. 2022. PLoS Neglected Tropical Diseases. , volume: 16(7) (no pagination), issue: , pages: . Doi: https://dx.doi.org/10.1371/journal.pntd.0010629. | Tanzania | Cross-sectional within longitudinal | TF | TI | no | yes | yes | yes | Checklist for Prevalence Studies | Yes | Yes | Unclear | Yes | Yes | Yes | Yes | Yes | Yes | | | | | | | | | | | [8/9] (89%) | NA | 89% |
| Cochereau, I.; Goldschmidt, P.; Goepogui, A.; Afghani, T.; Delval, L.; Pouliquen, P.; Bourcier, T.; Robert, P. Y.Efficacy and safety of short duration azithromycin eye drops versus azithromycin single oral dose for the treatment of trachoma in children: a randomised, controlled, double-masked clinical trial. 2007. British Journal of Ophthalmology. , volume: 91, issue: 5, pages: 667-672. Doi: http://dx.doi.org/10.1136/bjo.2006.099275. | Guinea and Pakistan | Cross-sectional within RCT | TF | TF and/or TI | no | no | no | no | Checklist for Prevalence Studies | No | Unclear | Unclear | Yes | Unclear | Yes | Yes | Yes | Unclear | | | | | | | | | | | [4/9] (44%) | NA | 44% |
| Cocks, N.; Rainima-Qaniuci, M.; Yalen, C.; Macleod, C.; Nakolinivalu, A.; Migchelsen, S.; Roberts, C. H.; Butcher, R.; Kama, M.; Mabey, D.; Marks, M.Community seroprevalence survey for yaws and trachoma in the Western Division of Fiji. 2016. Transactions of the Royal Society of Tropical Medicine and Hygiene. , volume: 110, issue: 10, pages: 582-587. Doi: http://dx.doi.org/10.1093/trstmh/trw069. | Fiji | Cross-sectional | TF | TI | no | yes | yes | no | Checklist for Prevalence Studies | Yes | Yes | Yes | Yes | Yes | Yes | Yes | Yes | Yes | | | | | | | | | | | [9/9] (100%) | NA | 100% |
| Cox, J. T.; Mkocha, H.; Munoz, B.; West, S.Prevalence of trachomatous scarring in children in a formerly endemic district of Tanzania. 2017. Investigative Ophthalmology and Visual Science. Conference. , volume: 58, issue: 8, pages: . Doi: . | Tanzania | Cross-sectional | TF | no | no | yes | no | no | Checklist for Prevalence Studies | Yes | Unclear | Unclear | No | Unclear | Unclear | Unclear | Unclear | Unclear | | | | | | | | | | | [1/9] (11%) | NA | 11% |
| Cromwell, E. A.; Amza, A.; Kadri, B.; Beidou, N.; King, J. D.; Sankara, D.; Mosher, A. W.; Hassan, S.; Kane, S.; Emerson, P. M.Trachoma prevalence in Niger: results of 31 district-level surveys. 2014. Transactions of the Royal Society of Tropical Medicine and Hygiene. , volume: 108, issue: 1, pages: 42-48. Doi: http://dx.doi.org/10.1093/trstmh/trt101. | Niger | Cross-sectional | TF | TI | no | no | no | no | Checklist for Prevalence Studies | Yes | Yes | Yes | No | Yes | Yes | Yes | Yes | Yes | | | | | | | | | | | [8/9] (89%) | NA | 89% |
| Cumberland, P.; Girum, Hailu; Todd, J.Active trachoma in children aged three to nine years in rural communities in Ethiopia: prevalence, indicators and risk factors. 2005. Transactions of the Royal Society of Tropical Medicine and Hygiene. , volume: 99, issue: 2, pages: 120-127. Doi: http://dx.doi.org/10.1016/j.trstmh.2004.03.011. | Ethiopia | Cross-sectional | TF | TI | no | no | no | no | Checklist for Prevalence Studies | Yes | Yes | Unclear | No | Unclear | Yes | Yes | Yes | Unclear | | | | | | | | | | | [5/9] (56%) | NA | 56% |
| Cumberland, P.; Edwards, T.; Hailu, G.; Harding-Esch, E.; Andreasen, A.; Mabey, D.; Todd, J.The impact of community level treatment and preventative interventions on trachoma prevalence in rural Ethiopia. 2008. International Journal of Epidemiology. , volume: 37, issue: 3, pages: 549-558. Doi: http://dx.doi.org/10.1093/ije/dyn045. | Ethiopia | Cross-sectional | TF | no | no | yes | no | no | Checklist for Prevalence Studies | Yes | Yes | Unclear | Yes | Unclear | Yes | Yes | Yes | Unclear | | | | | | | | | | | [6/9] (67%) | NA | 67% |

S6. Table of included studies

| | | | | | | | | | | | | Risk of Bias checklist items** | | | | | | | | | | | | | | | | | | | | | |
|---|---|---|---|---|---|---|---|---|---|---|---|---|---|---|---|---|---|---|---|---|---|---|---|---|---|---|---|---|---|---|---|---|---|
| reference | Country or countries of data collection | Type of study | TF or FPC | other clinical indicators? | photo-graded TF or FPC? | infection? | serology? | SCR? | Critical Appraisal Tool used** | Prevalence study: Was the sample frame appropriate to address the target pop? | Prevalence study: Were study participants sampled in an appropriate way? | Prevalence study: Was the sample size adequate? | Prevalence study: Were the study subjects and setting described in detail? | Prevalence study: Was the data analysis conducted with sufficient coverage of the identified sample? | Prevalence study: Were valid methods used for the identification of the condition(s)? | Prevalence study: Was the condition measured in a standard, reliable way for all participants? | Prevalence study: Was there appropriate statistical analysis? | Prevalence study: Was the response rate adequate, and if not, was the low response rate managed appropriately? | DTA study: Was a consecutive or random sample of patients enrolled? | DTA study: Was a case-control design avoided? | DTA study: Did the study avoid inappropriate exclusions? | DTA: Were the index test results interpreted without knowledge of the results of the reference standard? | DTA: If a threshold was used, was it pre-specified? | DTA: Is the reference standard likely to correctly classify the target condition? | DTA: Were the reference standard results interpreted without knowledge of the results of the index test? | DTA: Was there an appropriate interval between index test and reference standard? | DTA: Did all patients receive the same reference standard? | DTA: Were all patients included in the analysis? | Prevalence study score [meets criteria/total] (%) | DTA study score [meets criteria/total] (%) | Max score (if both checklists used, higher of 2 scores) |
| Damasceno, R. W. F.; Santos, R. R.; Cavalcanti, T. R. T.; Hida, R. Y.; Santos, M. J.; Santos, A. M. C.; Dantas, P. E. C.Trachoma: epidemiologic study of scholars from Alagoas state - Brazil. 2009. Arquivos Brasileiros de Oftalmologia. , volume: 72, issue: 3, pages: 355-359. Doi: http://dx.doi.org/10.1590/S0004-27492009000300014. | Brazil | Cross-sectional | TF | TI | no | no | no | no | Checklist for Prevalence Studies | Yes | Yes | Yes | Yes | Unclear | Yes | Yes | Yes | Unclear | | | | | | | | | | | [7/9] (78%) | NA | 78% |
| Datta, P.; Frost, E.; Peeling, R.; Masinde, S.; Deslandes, S.; Echelu, C.; Wamola, I.; Brunham, R. C.Ophthalmia neonatorum in a trachoma endemic area. 1994. Sexually Transmitted Diseases. , volume: 21, issue: 1, pages: 44930. Doi: . | Kenya | Cross-sectional | TF | TI | no | yes | no | no | Checklist for DTA studies | | | | | | | | | | Consecutive | Yes | No | Unclear | NA | Yes | Yes | Yes | Yes | No | NA | [5/10] (50%) | 50% |
| Debrah, O.; Mensah, E. O.; Senyonjo, L.; Souza, D. K. de; Hervie, T. E.; Agyemang, D.; Bakajika, D.; Marfo, B.; Ahorsu, F.; Wanye, S.; Bailey, R.; Koroma, J. B.; Aboe, A.; Biritwum, N. K.Elimination of trachoma as a public health problem in Ghana: providing evidence through a pre-validation survey. 2017. PLoS Neglected Tropical Diseases. , volume: 11, issue: 12, pages: e0006099. Doi: http://dx.doi.org/10.1371/journal.pntd.0006099. | Ghana | Cross-sectional | TF | TI | no | no | no | no | Checklist for Prevalence Studies | Yes | Yes | Yes | Yes | Yes | Yes | Yes | Yes | Yes | | | | | | | | | | | [9/9] (100%) | NA | 100% |
| Derrick, T.; Holland, M. J.; Cassama, E.; Markham-David, R.; Nabicassa, M.; Marks, M.; Bailey, R. L.; Last, A. R.Can corneal pannus with trachomatous inflammation - follicular be used in combination as an improved specific clinical sign for current ocular Chlamydia trachomatis infection?. 2016. Parasites and Vectors. , volume: 9, issue: 30, pages: . Doi: . | Guinea Bissau | Case-control | TF | no | no | yes | no | no | Checklist for DTA studies | | | | | | | | | | Unclear | No | Yes | Unclear | Yes | Yes | Unclear | Yes | Yes | Yes | NA | [6/10] (60%) | 60% |
| Derrick, T.; Last, A. R.; Burr, S. E.; Roberts, C. H.; Nabicassa, M.; Cassama, E.; Bailey, R. L.; Mabey, D. C. W.; Burton, M. J.; Holland, M. J.Inverse relationship between microRNA-155 and -184 expression with increasing conjunctival inflammation during ocular Chlamydia trachomatis infection. 2016. BMC Infectious Diseases. , volume: 16, issue: 60, pages: . Doi: . | Guinea Bissau | Case-control | TF | P3 (FPC) | no | yes | no | no | Checklist for DTA studies | | | | | | | | | | Unclear | No | Yes | Yes | Yes | Yes | Unclear | Yes | Yes | Yes | NA | [7/10] (70%) | 70% |
| Derrick, T. R.; Sandetskaya, N.; Pickering, H.; Kolsch, A.; Ramadhani, A.; Mafuru, E.; Massae, P.; Malisa, A.; Mtuy, T.; Burton, M. J.; Holland, M. J.; Kuhlmeier, D.DjinniChip: evaluation of a novel molecular rapid diagnostic device for the detection of Chlamydia trachomatis in trachoma-endemic areas. 2020. Parasites and Vectors. , volume: 13, issue: 533, pages: . Doi: http://dx.doi.org/10.1186/s13071-020-04414-6. | Tanzania | Cross-sectional | TF | P3 (FPC) | no | yes | no | no | Checklist for DTA studies | | | | | | | | | | All children from selected villages | Yes | Yes | Yes | Yes | Yes | Unclear | Yes | Yes | Yes | NA | [8/10] (80%) | 80% |
| de Sousa Meneghim, R. L. F.; Viveiros, M. M. H.; Galindo-Ferreiro, A.; Padovani, C. R.; Schellini, S. A.Sequelae from Epidemic Viral Conjunctivitis Can Be Associated with Inflammatory Trachoma in Schoolchildren?. 2018. Seminars in Ophthalmology. , volume: 33, issue: 2, pages: 219-222. Doi: 10.1080/08820538.2016.1208762. | Brazil | Cross-sectional | TF | TI | no | no | no | no | Checklist for Prevalence Studies | No (what about private schools?) | Yes | Yes | No | Yes | Yes | Yes | Yes | Yes | | | | | | | | | | | [7/9] (78%) | NA | 78% |
| Dorkenoo, A. M.; Bronzan, R. N.; Ayena, K. D.; Anthony, G.; Agbo, Y. M.; Sognikin, K. S. E.; Dogbe, K. S.; Amza, A.; Sodahlon, Y.; Mathieu, E.Nationwide integrated mapping of three neglected tropical diseases in Togo: countrywide implementation of a novel approach. 2012. Tropical Medicine and International Health. , volume: 17, issue: 7, pages: 896-903. Doi: http://dx.doi.org/10.1111/j.1365-3156.2012.03004.x. | Togo | Cross-sectional | TF | TI | no | no | no | no | Checklist for Prevalence Studies | Yes | Unclear | Unclear | No | Unclear | Yes | Yes | Yes | Unclear | | | | | | | | | | | [4/9] (44%) | NA | 44% |
| Ahmed Badei, Duale; Nebiyu Negussu, Ayele; Macleod, C. K.; Amir Bedri, Kello; Zelalem Eshetu, Gezachew; Amsalu, Binegdie; Michael, Dejene; Wondu, Alemayehu; Flueckiger, R. M.; Massae, P. A.; Willis, R.; Biruck Kebede, Negash; Solomon, A. W.Epidemiology of trachoma and its implications for implementing the "SAFE" strategy in Somali Region, Ethiopia: results of 14 population-based prevalence surveys. 2018. Special Issue: 2017 Global Trachoma Mapping Project.. , volume: 25, issue: Suppl. 1, pages: 25-32. Doi: http://dx.doi.org/10.1080/09286586.2017.1409358. | Ethiopia | Cross-sectional | TF | TI | no | no | no | no | Checklist for Prevalence Studies | Yes | Yes | Unclear | Yes | Yes | Yes | Yes | Yes | Yes | | | | | | | | | | | [8/9] (89%) | NA | 89% |
| Edwards, T.; Smith, J.; Sturrock, H. J. W.; Kur, L. W.; Sabasio, A.; Finn, T. P.; Lado, M.; Haddad, D.; Kolaczinski, J. H.Prevalence of trachoma in Unity State, South Sudan: results from a large-scale population-based survey and potential implications for further surveys. 2012. PLoS Neglected Tropical Diseases. , volume: 6, issue: 4, pages: e1585. Doi: http://dx.doi.org/10.1371/journal.pntd.0001585. | South Sudan | Cross-sectional | TF | TI | no | no | no | no | Checklist for Prevalence Studies | Yes | Yes | Unclear | Yes | Yes | Yes | Yes | Yes | Yes | | | | | | | | | | | [8/9] (89%) | NA | 89% |
| Ejigu, M.; Kariuki, M. M.; Ilako, D. R.; Gelaw, Y.Rapid trachoma assessment in Kersa District, Southwest Ethiopia. 2013. Ethiopian Journal of Health Sciences. , volume: 23, issue: 1, pages: 44935. Doi: . | Ethiopia | Cross-sectional | TF | TI | no | no | no | no | Checklist for Prevalence Studies | Yes | Unclear - convenience sampling of sub-districts as per TRA methodology | Yes | Yes | Unclear | Yes | Yes | Yes | Unclear | | | | | | | | | | | [6/9] (67%) | NA | 67% |
| Ervin, A. M.; Mkocha, H.; Munoz, B.; Dreger, K.; Dize, L.; Gaydos, C.; Quinn, T. C.; West, S. K.Surveillance and azithromycin treatment for newcomers and travelers evaluation (ASANTE) trial design and baseline characteristics. 2016. Ophthalmic Epidemiology. , volume: 23, issue: 6, pages: 347-353. Doi: http://dx.doi.org/10.1080/09286586.2016.1238947. | Tanzania | Cross-sectional | TF | TI, TF and TI | no | yes | no | no | Checklist for Prevalence Studies | Yes | Yes | Unclear | Yes | Unclear | Yes | Yes | Yes | Unclear | | | | | | | | | | | [6/9] (67%) | NA | 67% |
| Faal, N.; Bailey, R. L.; Sarr, I.; Joof, H.; Mabey, D. C. W.; Holland, M. J.Temporal cytokine gene expression patterns in subjects with trachoma identify distinct conjunctival responses associated with infection. 2005. Clinical and Experimental Immunology. , volume: 142, issue: 2, pages: 347-353. Doi: http://dx.doi.org/10.1111/j.1365-2249.2005.02917.x. | Gambia | Cross-sectional | TF | TF and TI | no | yes | no | no | Checklist for DTA studies | | | | | | | | | | Unclear | Yes | Yes | Unclear | Yes | Yes | Yes | Yes | Yes | Yes | NA | [8/10] (80%) | 80% |
| Faal, N.; Bailey, R. L.; Jeffries, D.; Joof, H.; Sarr, I.; Laye, M.; Mabey, D. C.; Holland, M. J.Conjunctival FOXP3 expression in trachoma: do regulatory T cells have a role in human ocular Chlamydia trachomatis infection?. 2006. PLoS Medicine / Public Library of Science. , volume: 3, issue: 8, pages: e266. Doi: . | Gambia | Cross-sectional | TF | TI | no | yes | no | no | Checklist for DTA studies | | | | | | | | | | Unclear | Yes | Yes | Unclear | Yes | Yes | Yes | Yes | Yes | Unclear | NA | [7/10] (70%) | 70% |
| Favacho, J.; Alves da Cunha, A. J. L.; Gomes, S. T. M.; Freitas, F. B.; Queiroz, M. A. F.; Vallinoto, A. C. R.; Ishak, R.; Ishak, M. D. O. G.Prevalence of trachoma in school children in the Marajo Archipelago, Brazilian Amazon, and the impact of the introduction of educational and preventive measures on the disease over eight years. 2018. PLoS Neglected Tropical Diseases. , volume: 12(2) (no pagination), issue: , pages: . Doi: https://dx.doi.org/10.1371/journal.pntd.0006282. | Brazil | Cross-sectional | TF | TI | no | no | no | no | Checklist for Prevalence Studies | Yes | Yes | Unclear | Yes | Unclear | Yes | Yes | Yes | Unclear | | | | | | | | | | | [6/9] (67%) | NA | 67% |
| Faye, M.; Kuper, H.; Dineen, B.; Bailey, R.Rapid assessment for prioritisation of trachoma control at community level in one district of the Kaolack Region, Senegal. 2006. Transactions of the Royal Society of Tropical Medicine and Hygiene. , volume: 100, issue: 2, pages: 149-157. Doi: http://dx.doi.org/10.1016/j.trstmh.2005.06.029. | Senegal | Cross-sectional | TF | TI, TF and TI | no | no | no | no | Checklist for Prevalence Studies | Yes | Yes | Yes | Unclear | Unclear | Yes | Yes | Yes | Unclear | | | | | | | | | | | [6/9] (67%) | NA | 67% |
| Felton, JMStudies of human immune responses to various antigenic proteins of Chlamydia trachomatis.. 2003. , volume: , issue: , pages: . Doi: . | Gambia | Case-control | TF | TI | no | no | no | no | Checklist for DTA studies | | | | | | | | | | Unclear | No | Yes | No | Yes | Yes | No | Yes | Yes | Yes | NA | [6/10] (60%) | 60% |
| Feng, L.; Lu, X.; Yu, Y.; Wang, T.; Luo, S.; Sun, Z.; Duan, Q.; Wang, N.; Song, L.Survey, culture, and genome analysis of ocular Chlamydia trachomatis in Tibetan Boarding Primary Schools in Qinghai Province, China. 2017. Frontiers in Cellular and Infection Microbiology. , volume: 6, issue: JAN, pages: . Doi: 10.3389/fcimb.2016.00207. | China | Cross-sectional | TF | TI, <5 follicles | no | no | no | no | Checklist for Prevalence Studies | Yes | Unclear | Unclear | No | Unclear | Yes | Yes | Yes | Unclear | | | | | | | | | | | [4/9] (44%) | NA | 44% |
| Ferede, A. T.; Dadi, A. F.; Tariku, A.; Adane, A. A.Prevalence and determinants of active trachoma among preschool-aged children in Dembia District, Northwest Ethiopia. 2017. Infectious Diseases of Poverty. , volume: 6(1) (no pagination), issue: , pages: . Doi: https://dx.doi.org/10.1186/s40249-017-0345-8. | Ethiopia | Cross-sectional | TF | TI | no | no | no | no | Checklist for Prevalence Studies | Yes | Yes | Yes | No | Yes | Yes | Yes | Yes | Yes | | | | | | | | | | | [8/9] (89%) | NA | 89% |
| Freitas, H. S. A.; Medina, N. H.; Lopes, M. F. C.; Soares, O. E.; Teodoro, M. T. C.; Ramalho, K. R. B.; Caligaris, L. S. A.; Morschbacher, R.; de Menezes, M. N. C.; Luna, E. J. A.Trachoma in Indigenous Settlements in Brazil, 2000-2008. 2016. Ophthalmic Epidemiology. , volume: 23(6), issue: , pages: 354-359. Doi: https://dx.doi.org/10.3109/09286586.2015.1131305. | Brazil | Cross-sectional | TF | TI | no | no | no | no | Checklist for Prevalence Studies | Yes | Unclear | Unclear | Yes | Unclear | Yes | Unclear | Unclear | Unclear | | | | | | | | | | | [3/9] (33%) | NA | 33% |
| Gelaye, B.; Kumie, A.; Aboset, N.; Berhane, Y.; Williams, M. A.School-based intervention: evaluating the role of water, latrines and hygiene education on trachoma and intestinal parasitic infections in Ethiopia. 2014. Journal of Water, Sanitation and Hygiene for Development. , volume: 4, issue: 1, pages: 120-130. Doi: http://dx.doi.org/10.2166/washdev.2013.060. | Ethiopia | Cross-sectional within longitudinal | TF | TI | no | no | no | no | Checklist for Prevalence Studies | Yes | Yes | Unclear | Yes | Unclear | Yes | Yes | Yes | Unclear | | | | | | | | | | | [6/9] (67%) | NA | 67% |
| Genet, A.; Dagnew, Z.; Melkie, G.; Keleb, A.; Motbainor, A.; Mebrat, A.; Leshargie, C. T.Prevalence of active trachoma and its associated factors among 1-9 years of age children from model and non-model kebeles in Dangila district, northwest Ethiopia. 2022. PLoS ONE [Electronic Resource]. , volume: 17(6 June) (no pagination), issue: , pages: . Doi: https://dx.doi.org/10.1371/journal.pone.0268441. | Ethiopia | Cross-sectional | TF | TI | no | no | no | no | Checklist for Prevalence Studies | Yes | Yes | Yes | Yes | Yes | Yes | Yes | Yes | Yes | | | | | | | | | | | [9/9] (100%) | NA | 100% |
| Ghasemian, E.; Inic-Kanada, A.; Collingro, A.; Tagini, F.; Stein, E.; Alchalabi, H.; Schuerer, N.; Keše, D.; Babiker, B. E.; Borel, N.; Greub, G.; Barisani-Asenbauer, T.Detection of Chlamydiaceae and Chlamydia-like organisms on the ocular surface of children and adults from a trachoma-endemic region. 2018. Scientific Reports. , volume: 8, issue: 1, pages: 7432. Doi: http://dx.doi.org/10.1038/s41598-018-23887-1. | Sudan | Case-control | TF | no | no | yes | no | no | Checklist for DTA studies | | | | | | | | | | Unclear | No | Yes | Unclear | Yes | Yes | Yes | Unclear | Yes | Yes | NA | [6/10] (60%) | 60% |

S6. Table of included studies

| | | | | | | | | | | Risk of Bias checklist items** | | | | | | | | | | | | | | | | | | | | | |
|---|---|---|---|---|---|---|---|---|---|---|---|---|---|---|---|---|---|---|---|---|---|---|---|---|---|---|---|---|---|---|---|
| reference | Country or countries of data collection | Type of study | TF or FPC | other clinical indicators? | photo-graded TF or FPC? | infection? | serology? | SCR? | Critical Appraisal Tool used** | Prevalence study: Was the sample frame appropriate to address the target pop? | Prevalence study: Were study participants sampled in an appropriate way? | Prevalence study: Was the sample size adequate? | Prevalence study: Were the study subjects and setting described in detail? | Prevalence study: Was the data analysis conducted with sufficient coverage of the identified sample? | Prevalence study: Were valid methods used for the identification of the condition(s)? | Prevalence study: Was the condition measured in a standard, reliable way for all participants? | Prevalence study: Was there appropriate statistical analysis? | Prevalence study: Was the response rate adequate, and if not, was the low response rate managed appropriately? | DTA study: Was a consecutive or random sample of patients enrolled? | DTA study: Was a case-control design avoided? | DTA study: Did the study avoid inappropriate exclusions? | DTA: Were the index test results interpreted without knowledge of the results of the reference standard? | DTA: If a threshold was used, was it pre-specified? | DTA: Is the reference standard likely to correctly classify the target condition? | DTA: Were the reference standard results interpreted without knowledge of the results of the index test? | DTA: Was there an appropriate interval between index test and reference standard? | DTA: Did all patients receive the same reference standard? | DTA: Were all patients included in the analysis? | Prevalence study score [meets criteria/total] (%) | DTA study score [meets criteria/total] (%) | Max score (if both checklists used, higher of 2 scores) |
| Ghasemian, E.; Inic-Kanada, A.; Collingro, A.; Mejdoubi, L.; Alchalabi, H.; Kese, D.; Elshafie, B. E.; Hammou, J.; Barisani-Asenbauer, T.Comparison of genovars and Chlamydia trachomatis infection loads in ocular samples from children in two distinct cohorts in Sudan and Morocco. 2021. PLoS Neglected Tropical Diseases. , volume: 15, issue: 8, pages: . Doi: http://dx.doi.org/10.1371/journal.pntd.0009655. | Morocco | Case-control | TF | no | no | yes | no | no | Checklist for DTA studies | | | | | | | | | | Unclear | No | Yes | Unclear | Yes | Yes | Yes | Unclear | Yes | Yes | NA | [6/10] (60%) | 60% |
| Goldschmidt, P.; Afghani, T.; Nadeem, M.; Ali-Khan, W.; Chaumeil, C.; De Barbeyrac, B.Clinical and microbiological diagnosis of trachoma in children living in rural areas in the District of Attock, Punjab, Pakistan. 2006. Ophthalmic Epidemiology. , volume: 13(5), issue: , pages: 335-342. Doi: https://dx.doi.org/10.1080/09286580600943796. | Pakistan | Cross-sectional | TF | TI | no | yes | no | no | Checklist for Prevalence Studies | Yes | Unclear | Unclear | Yes | Unclear | Yes | Yes | Yes | Unclear | | | | | | | | | | | [5/9] (56%) | NA | 56% |
| Goldschmidt, P.; Benallaoua, D.; Amza, A.; Einterz, E.; Huguet, P.; Poisson, F.; Bilinkai, A. B.; Ismaila, M.; Bensaid, P.; Bella, L.; Chaumeil, C.Clinical and microbiological assessment of trachoma in the Kolofata Health District, Far North Region, Cameroon. 2012. Tropical Medicine and Health. , volume: 40, issue: 1, pages: 45121. Doi: http://dx.doi.org/10.2149/tmh.2011-26. | Cameroon | Cross-sectional | TF | TI | no | yes | no | no | Checklist for Prevalence Studies | Yes | Yes | Yes | Yes | Yes | Yes | Yes | Yes | Yes | | | | | | | | | | | [9/9] (100%) | NA | 100% |
| Goldschmidt, P.; Einterz, E.The limits of medical interventions for the elimination of preventable blindness. 2014. Tropical Medicine and Health. , volume: 42, issue: 1, pages: 43-52. Doi: http://dx.doi.org/10.2149/tmh.2013-26. | Cameroon | Cross-sectional | TF | TF and TI | no | no | no | no | Checklist for Prevalence Studies | Yes | Yes | Yes | Yes | Unclear | Yes | Yes | Yes | Unclear | | | | | | | | | | | [7/9] (78%) | NA | 78% |
| Golovaty, I.; Jones, L.; Gelaye, B.; Melkie, Tilahun; Habtamu, Belete; Abera, Kumie; Yemane, Berhane; Williams, M. A.Access to water source, latrine facilities and other risk factors of active trachoma in Ankober, Ethiopia. 2009. PLoS ONE [Electronic Resource]. , volume: , issue: August, pages: e6702. Doi: http://dx.doi.org/10.1371/journal.pone.0006702. | Ethiopia | Cross-sectional | TF | TI, TF and/or TI | no | no | no | no | Checklist for Prevalence Studies | Yes | Yes | Yes | Yes | Unclear | Yes | Yes | Yes | Unclear | | | | | | | | | | | [7/9] (78%) | NA | 78% |
| Gower, E. W.; Solomon, A. W.; Burton, M. J.; Aguirre, A.; Munoz, B.; Bailey, R.; Holland, M.; Makalo, P.; Massae, P.; Mkocha, H.; Mabey, D. C.; West, S. K.Chlamydial positivity of nasal discharge at baseline is associated with ocular chlamydial positivity 2 months following azithromycin treatment. 2006. Investigative Ophthalmology & Visual Science. , volume: 47, issue: 11, pages: 4767-4771. Doi: . | Gambia, Tanzania | Cross-sectional | TF | TI | no | yes | no | no | Both | Yes | Yes | Yes | No | Yes | Yes | Yes | Yes | Yes | Unclear | Yes | Yes | Unclear | Yes | Yes | Yes | Yes | Yes | Yes | [8/9] (89%) | [8/10] (80%) | 89% |
| Gwyn, S. E.; Xiang, L.; Kandel, R. P.; Dean, D.; Gambhir, M.; Martin, D. L.Prevalence of Chlamydia trachomatis-Specific Antibodies before and after Mass Drug Administration for Trachoma in Community-Wide Surveys of Four Communities in Nepal. 2018. American Journal of Tropical Medicine & Hygiene. , volume: 98, issue: 1, pages: 216-220. Doi: https://dx.doi.org/10.4269/ajtmh.17-0102. | Nepal | Cross-sectional | TF | no | no | no | yes | no | Checklist for Prevalence Studies | Yes | Yes | Yes | Yes | Yes | Yes | Yes | Yes | Unclear | | | | | | | | | | | [8/9] (89%) | NA | 89% |
| Gwyn, S.; Mkocha, H.; Randall, J. M.; Kasubi, M.; Martin, D. L.Optimization of a rapid test for antibodies to the Chlamydia trachomatis antigen Pgp3. 2019. Diagnostic Microbiology and Infectious Disease. , volume: 93, issue: 4, pages: 293-298. Doi: http://dx.doi.org/10.1016/j.diagmicrobio.2018.11.001. | Tanzania | Cross-sectional | TF | no | no | yes | yes | no | Checklist for Prevalence Studies | Yes | Yes | Unclear | Yes | Unclear | Yes | Yes | Yes | Unclear | | | | | | | | | | | [6/9] (67%) | NA | 67% |
| Gwyn, S.; Awoussi, M. S.; Bakhtiari, A.; Bronzan, R. N.; Crowley, K.; Harding-Esch, E. M.; Kassankogno, Y.; Kilangalanga, J. N.; Makangila, F.; Mupoyi, S.; Ngondi, J.; Ngoyi, B.; Palmer, S.; Randall, J. M.; Seim, A.; Solomon, A. W.; Stewart, R.; Togbey, K.; Uvon, P. A.; Martin, D. L.Comparison of platforms for testing antibodies to Chlamydia trachomatis antigens in the Democratic Republic of the Congo and Togo. 2021. Scientific Reports. , volume: 11, issue: 1, pages: . Doi: http://dx.doi.org/10.1038/s41598-021-86639-8. | Democratic Republic of the Congo, Togo | Cross-sectional | TF | no | no | no | yes | yes | Checklist for Prevalence Studies | Yes | Yes | Unclear | Yes | Yes | Yes | Yes | Yes | Yes | | | | | | | | | | | [8/9] (89%) | NA | 89% |
| Gwyn, S.; Nute, A. W.; Eshetu, Sata; Zerihun, Tadesse; Ambahun, Chernet; Mahteme, Haile; Taye, Zeru; Bethea, D.; Laurent, C.; Callahan, E. K.; Nash, S. D.; Martin, D. L.The performance of immunoassays to measure antibodies to the Chlamydia trachomatis antigen Pgp3 in different epidemiological settings for trachoma. 2021. American Journal of Tropical Medicine and Hygiene. , volume: 105, issue: 5, pages: 1362-1367. Doi: http://dx.doi.org/10.4269/ajtmh.21-0541. | Ethiopia | Cross-sectional | TF | TI | no | yes | yes | yes | Checklist for Prevalence Studies | Yes | Yes | Unclear | Yes | Unclear | Yes | Yes | Yes | Unclear | | | | | | | | | | | [6/9] (67%) | NA | 67% |
| Gyasi, M. E.; Noine, A.; Yayemain, D.; Debrah, O. A.; Asamani, D.; Gyapong, J.; Amoaku, W. M. K.Trachoma in northern Ghana: a need for further studies. 2010. Ophthalmic Epidemiology. , volume: 17, issue: 6, pages: 343-348. Doi: http://dx.doi.org/10.3109/09286586.2010.528132. | Ghana | Cross-sectional | TF | TI | no | no | no | no | Checklist for Prevalence Studies | Yes | Yes | Yes | No | Yes | Yes | Yes | Yes | Yes | | | | | | | | | | | [8/9] (89%) | NA | 89% |
| Hagan, M.; Yayemain, D.; Ahorsu, F.; Aboe, A.Prevalence of active trachoma two years after control activities. 2009. Ghana Medical Journal. , volume: 43, issue: 2, pages: 54-60. Doi: . | Ghana | Cross-sectional | 2 or more follicles | TF and TI | no | no | no | no | Checklist for Prevalence Studies | Yes | Yes | Unclear | No | Unclear | No, 2 follicles counted as TF | Yes | Yes | Unclear | | | | | | | | | | | [4/9] (44%) | NA | 44% |
| Tesfaye Haileselassie, Adera; Macleod, C.; Misganu, Endriyas; Dejene, M.; Willis, R.; Chu, B. K.; Yohannes, Letamo; Tebeje, Misganaw; Tamiru, Mesele; Emebet, Mekonnen; Alemayehu, Sisay; Yeneneh, Mulugeta; Wondu, Alemayehu; Khumbo, Kalua; Tezera Kifle, Destu; Adamu, Y.; Smith, J. L.; Abu, Beyene; Tadesse, A.; Solomon, A. W.Prevalence of and risk factors for trachoma in Southern Nations, Nationalities, and Peoples' Region, Ethiopia: results of 40 population-based prevalence surveys carried out with the Global Trachoma Mapping Project. 2016. Special Issue: Trachoma mapping. , volume: 23, issue: Suppl. 1, pages: 84-93. Doi: . | Ethiopia | Cross-sectional | TF | TI, TF and/or TI | no | no | no | no | Checklist for Prevalence Studies | Yes | Yes | Yes | Yes | Unclear | Yes | Yes | Yes | Unclear | | | | | | | | | | | [7/9] (78%) | NA | 78% |
| Hammou, J.; Guagliardo, S. A. J.; Obtel, M.; Razine, R.; Haroun, A. E.; Youbi, M.; Bellefquih, A. M.; White, M.; Gwyn, S.; Martin, D. L.Post-Validation Survey in Two Districts of Morocco after the Elimination of Trachoma as a Public Health Problem. 2022. American Journal of Tropical Medicine & Hygiene. , volume: 28, issue: , pages: 28. Doi: https://dx.doi.org/10.4269/ajtmh.21-1140. | Morocco | Cross-sectional | TF | TI, TF and/or TI | no | no | yes | yes | Checklist for Prevalence Studies | Yes | Yes | Yes | Yes | Yes | Yes | Yes | Yes | Yes | | | | | | | | | | | [9/9] (100%) | NA | 100% |
| Harding-Esch, E. M.; Edwards, T.; Sillah, A.; Sarr-Sissoho, I.; Aryee, E. A.; Snell, P.; Holland, M. J.; Mabey, D. C.; Bailey, R. L.Risk factors for active trachoma in The Gambia. 2008. Transactions of the Royal Society of Tropical Medicine and Hygiene. , volume: 102, issue: 12, pages: 1255-1262. Doi: http://dx.doi.org/10.1016/j.trstmh.2008.04.022. | Gambia | Cross-sectional | TF | TI, TF and/or TI | no | yes | no | no | Checklist for Prevalence Studies | Yes | Yes | Yes | Yes | Unclear | Yes | Yes | Yes | Unclear | | | | | | | | | | | [7/9] (78%) | NA | 78% |
| Harding-Esch, E. M.; Edwards, T.; Sillah, A.; Sarr, I.; Roberts, C. H.; Snell, P.; Aryee, E.; Molina, S.; Holland, M. J.; Mabey, D. C. W.; Bailey, R. L.Active trachoma and ocular Chlamydia trachomatis infection in two Gambian regions: on course for elimination by 2020?. 2009. PLoS Neglected Tropical Diseases. , volume: 3, issue: 12, pages: e573. Doi: http://dx.doi.org/10.1371/journal.pntd.0000573. | Gambia | Cross-sectional | TF | TI | no | no | no | no | Checklist for Prevalence Studies | Yes | Yes | Yes | Yes | Unclear | Yes | Yes | Yes | Unclear | | | | | | | | | | | [7/9] (78%) | NA | 78% |
| Harding-Esch, E. M.; Edwards, T.; Mkocha, H.; Munoz, B.; Holland, M. J.; Burr, S. E.; Sillah, A.; Gaydos, C. A.; Stare, D.; Mabey, D. C. W.; Bailey, R. L.; West, S. K.Trachoma prevalence and associated risk factors in The Gambia and Tanzania: baseline results of a cluster randomised controlled trial. 2010. PLoS Neglected Tropical Diseases. , volume: 4, issue: 11, pages: e861. Doi: http://dx.doi.org/10.1371/journal.pntd.0000861. | Gambia | Cross-sectional | TF | TI, TF and TI | no | yes | no | no | Both | Yes | Yes | Unclear | Yes | Unclear | Yes | Yes | Yes | Unclear | Random | Yes | Yes | Unclear | Yes | Yes | Yes | Yes | Yes | Yes | [6/9] (67%) | [8/10] (80%) | 80% |
| Harding-Esch, E. M.; Sillah, A.; Edwards, T.; Burr, S. E.; Hart, J. D.; Joof, H.; Laye, M.; Makalo, P.; Manjang, A.; Molina, S.; Sarr-Sissoho, I.; Quinn, T. C.; Lietman, T.; Holland, M. J.; Mabey, D.; West, S. K.; Bailey, R.Mass treatment with azithromycin for trachoma: when is one round enough? Results from the PRET Trial in The Gambia. 2013. PLoS Neglected Tropical Diseases. , volume: 7, issue: 6, pages: e2115. Doi: http://dx.doi.org/10.1371/journal.pntd.0002115. | Gambia | Cross-sectional within longitudinal | TF | no | no | yes | no | no | Checklist for Prevalence Studies | Yes | Yes | Yes | Yes | Yes | Yes | Yes | Yes | Yes | | | | | | | | | | | [9/9] (100%) | NA | 100% |
| Harding-Esch, E. M.; Kadimpeul, J.; Sarr, B.; Sane, A.; Badji, S.; Laye, M.; Sillah, A.; Burr, S. E.; MacLeod, D.; Last, A. R.; Holland, M. J.; Mabey, D. C.; Bailey, R. L.Population-based prevalence survey of follicular trachoma and trachomatous trichiasis in the Casamance region of Senegal. 2017. BMC Public Health. , volume: 18, issue: 1, pages: 62. Doi: https://dx.doi.org/10.1186/s12889-017-4605-0. | Senegal | Cross-sectional | TF | TI | no | no | no | no | Checklist for Prevalence Studies | Yes | Yes | Yes | Yes | Yes | Yes | Yes | Yes | Yes | | | | | | | | | | | [9/9] (100%) | NA | 100% |
| Harding-Esch, E. M.; Holland, M. J.; Schemann, J. F.; Sillah, A.; Sarr, B.; Christerson, L.; Pickering, H.; Molina-Gonzalez, S.; Sarr, I.; Andreasen, A. A.; Jeffries, D.; Grundy, C.; Mabey, D. C. W.; Herrmann, B.; Bailey, R. L.Impact of a single round of mass drug administration with azithromycin on active trachoma and ocular Chlamydia trachomatis prevalence and circulating strains in The Gambia and Senegal. 2019. Parasites and Vectors. , volume: 12, issue: 497, pages: . Doi: . | Senegal | Cross-sectional | TF | TI | no | yes | no | no | Checklist for Prevalence Studies | Yes | Yes | Yes | No | Yes | Yes | Yes | Yes | Yes | | | | | | | | | | | [8/9] (89%) | NA | 89% |
| Hudson, A. P.; McEntee, C. M.; Reacher, M.; Whittum-Hudson, J. A.; Taylor, H. R.Inapparent ocular infection by Chlamydia trachomatis in experimental and human trachoma. 1992. Current Eye Research. , volume: 11, issue: 3, pages: 279-83. Doi: . | unknown | Cross-sectional | TF | TI | no | yes | no | no | Checklist for DTA studies | | | | | | | | | | Unclear | Unclear | Unclear | Unclear | Yes | Yes | Unclear | Yes | Yes | Unclear | NA | [4/10] (40%) | 40% |
| Huguet, P.; Bella, L.; Einterz, E. M.; Goldschmidt, P.; Bensaid, P.Mass treatment of trachoma with azithromycin 1.5% eye drops in the Republic of Cameroon: feasibility, tolerance and effectiveness. 2010. British Journal of Ophthalmology. , volume: 94, issue: 2, pages: 157-160. Doi: http://dx.doi.org/10.1136/bjo.2009.161513. | Cameroon | Cross-sectional | TF | TF and/or TI, TF and TI | no | no | no | no | Checklist for Prevalence Studies | Yes | Yes | Yes | Yes | Yes | Yes | Yes | Yes | Yes | | | | | | | | | | | [9/9] (100%) | NA | 100% |
| Jenson, A.; Dize, L.; Mkocha, H.; Munoz, B.; Lee, J.; Gaydos, C.; Quinn, T.; West, S. K.Field evaluation of the cepheid GeneXpert Chlamydia trachomatis assay for detection of infection in a trachoma endemic community in Tanzania. 2013. PLoS Neglected Tropical Diseases. , volume: 7, issue: 7, pages: e2265. Doi: http://dx.doi.org/10.1371/journal.pntd.0002265. | Tanzania | Cross-sectional | TF | TI, TF and TI | no | yes | no | no | Checklist for DTA studies | | | | | | | | | | Unclear | Yes | Yes | Unclear | Yes | Yes | Yes | Yes | Yes | Yes | NA | [8/10] (80%) | 80% |

S6. Table of included studies

| reference | Country or countries of data collection | Type of study | TF or FPC | other clinical indicators? | photo-graded TF or FPC? | infection? | serology? | SCR? | Critical Appraisal Tool used** | Risk of Bias checklist items** Prevalence study: Was the sample frame appropriate to address the target pop? | Prevalence study: Were study participants sampled in an appropriate way? | Prevalence study: Was the sample size adequate? | Prevalence study: Were the study subjects and setting described in detail? | Prevalence study: Was the data analysis conducted with sufficient coverage of the identified sample? | Prevalence study: Were valid methods used for the identification of the condition(s)? | Prevalence study: Was the condition measured in a standard, reliable way for all participants? | Prevalence study: Was there appropriate statistical analysis? | Prevalence study: Was the response rate adequate, and if not, was the low response rate managed appropriately? | DTA study: Was a consecutive or random sample of patients enrolled? | DTA study: Was a case-control design avoided? | DTA study: Did the study avoid inappropriate exclusions? | DTA: Were the index test results interpreted without knowledge of the results of the reference standard? | DTA: If a threshold was used, was it pre-specified? | DTA: Is the reference standard likely to correctly classify the target condition? | DTA: Were the reference standard results interpreted without knowledge of the results of the index test? | DTA: Was there an appropriate interval between index test and reference standard? | DTA: Did all patients receive the same reference standard? | DTA: Were all patients included in the analysis? | Prevalence study score [meets criteria/total] (%) | DTA study score [meets criteria/total] (%) | Max score (if both checklists used, higher of 2 scores) |
|---|---|---|---|---|---|---|---|---|---|---|---|---|---|---|---|---|---|---|---|---|---|---|---|---|---|---|---|---|---|---|---|---|
| Jip, N. F.; King, J. D.; Diallo, M. O.; Miri, E. S.; Hamza, A. T.; Ngondi, J.; Emerson, P. M.Blinding trachoma in Katsina State, Nigeria: population-based prevalence survey in ten local government areas. 2008. Ophthalmic Epidemiology. , volume: 15, issue: 5, pages: 294-302. Doi: http://dx.doi.org/10.1080/09286580802256542. | Nigeria | Cross-sectional | TF | TI, TF and/or TI | no | no | no | no | Checklist for Prevalence Studies | Yes | Yes | Yes | Yes | Unclear | Yes | Yes | Yes | Unclear | | | | | | | | | | | [7/9] (78%) | NA | 78% |
| Kabona, G.; Ngondi, J.; Kirumbi, E.; Kamugisha, M.; Kabole, I.; Simon, A.; Massae, P.; Gower, L.; Nanai, A.; Deogratius, D.; Mangasini, E.; Mmbando, D.; Aboe, A.; Mwingira, U. J.Evaluation of four years implementation of the safe strategy (surgery, antibiotics, facial cleanliness and environmental improvement) for trachoma control in handeni district, tanzania. 2013. American Journal of Tropical Medicine and Hygiene. , volume: 1), issue: , pages: 45210. Doi: . | Tanzania | Cross-sectional | TF | TI | no | no | no | no | Checklist for Prevalence Studies | Yes | Yes | Unclear | No | Unclear | Unclear | Yes | Unclear | Unclear | | | | | | | | | | | [3/9] (33%) | NA | 33% |
| Kalua, K.; Chirwa, T.; Kalilani, L.; Abbenyi, S.; Mukaka, M.; Bailey, R.Prevalence and risk factors for trachoma in central and southern Malawi. 2010. PLoS ONE [Electronic Resource]. , volume: , issue: February, pages: e9067. Doi: http://dx.doi.org/10.1371/journal.pone.0009067. | Malawi | Cross-sectional | TF | TI | no | no | no | no | Checklist for Prevalence Studies | Yes | Yes | Yes | Yes | Yes | Yes | Yes | Yes | Yes | | | | | | | | | | | [9/9] (100%) | NA | 100% |
| Kalua, K.; Singini, I.; Mukaka, M.; Msyamboza, K.; Masika, M.; Bailey, R.Scaling up of trachoma mapping in Salima District, Central Malawi. 2014. Health. , volume: 6, issue: 1, pages: 57-63. Doi: http://dx.doi.org/10.4236/health.2014.61009. | Malawi | Cross-sectional | TF | TI | no | no | no | no | Checklist for Prevalence Studies | Yes | Yes | Yes | Yes | Yes | Yes | Yes | Yes | Yes | | | | | | | | | | | [9/9] (100%) | NA | 100% |
| Kashaf, M. S.; Munoz, B. E.; Mkocha, H.; Wolle, M. A.; Naufal, F.; West, S. K.Incidence and progression of trachomatous scarring in a cohort of children in a formerly hyper-endemic district of Tanzania. 2020. PLoS Neglected Tropical Diseases. , volume: 14, issue: 10, pages: . Doi: http://dx.doi.org/10.1371/journal.pntd.0008708. | Tanzania | Cross-sectional within longitudinal | TF | no | no | yes | no | no | Checklist for Prevalence Studies | Yes | Yes | Yes | No | Unclear | Yes | Yes | Yes | Unclear | | | | | | | | | | | [6/9] (67%) | NA | 67% |
| Kassim, K.; Kassim, J.; Aman, R.; Abduku, M.; Tegegne, M.; Sahiledengle, B.Prevalence of active trachoma and associated risk factors among children of the pastoralist population in Madda Walabu rural district, Southeast Ethiopia: A community-based cross-sectional study. 2019. BMC Infectious Diseases. , volume: 19(1) (no pagination), issue: , pages: . Doi: https://dx.doi.org/10.1186/s12879-019-3992-5. | Ethiopia | Cross-sectional | TF | TI, TF and/or TI, TF and TI | no | no | no | no | Checklist for Prevalence Studies | Yes | Yes | Yes | Yes | Unclear | Yes | Yes | Yes | Unclear | | | | | | | | | | | [7/9] (78%) | NA | 78% |
| Kasubi, M.; Kabona, G.; Simon, A.; Kalabashanga, J.; Harran, M.; French, M.; Mwingira, U.; West, S.; Martin, D.; Ngondi, J.Alternative indicators to monitor trachoma elimination; does longitudinal chlamydia infection and antibody data add evidence to the understanding of resurgence. 2020. American Journal of Tropical Medicine and Hygiene. , volume: 103(5 SUPPL), issue: , pages: 365. Doi: . | Tanzania | Cross-sectional | TF | no | no | yes | yes | no | Checklist for Prevalence Studies | Yes | Unclear | Unclear | Yes | Unclear | Yes | Yes | Unclear | Unclear | | | | | | | | | | | [4/9] (44%) | NA | 44% |
| Katz, J.; West Jr, K. P.; Khatry, S. K.; LeClerq, S. C.; Pradhan, E. K.; Thapa, M. D.; Shrestha, S. R.; Taylor, H. R.Prevalence and risk factors for trachoma in Sarlahi district, Nepal. 1996. British Journal of Ophthalmology. , volume: 80(12), issue: , pages: 1037-1041. Doi: http://dx.doi.org/10.1136/bjo.80.12.1037. | Nepal | Cross-sectional | TF | TI | no | no | no | no | Checklist for Prevalence Studies | Yes | Yes | Unclear | No | Yes | Yes | Yes | Yes | Yes | | | | | | | | | | | [7/9] (78%) | NA | 78% |
| Kedir, S.; Lemnuro, K.; Yesse, M.; Abdella, B.; Muze, M.; Mustefa, A.; Musema, M.; Hussen, L.Prevalence and factors associated with active trachoma among children 1-9 years of age in the catchment population of tora primary hospital, silte zone, southern ethiopia, 2020. 2021. Open Ophthalmology Journal. , volume: 15, issue: 1, pages: 108-116. Doi: 10.2174/1874364102115010108. | Ethiopia | Cross-sectional | TF | TI, TF and/or TI, TF and TI | no | no | no | no | Checklist for Prevalence Studies | Yes | Yes | Yes | Yes | Yes | Yes | Yes | Yes | Yes | | | | | | | | | | | [9/9] (100%) | NA | 100% |
| Keenan, J. D.; See, C. W.; Moncada, J.; Ayele, B.; Gebre, T.; Stoller, N. E.; McCulloch, C. E.; Porco, T. C.; Gaynor, B. D.; Emerson, P. M.; Schachter, J.; Lietman, T. M.Diagnostic characteristics of tests for ocular Chlamydia after mass azithromycin distributions. 2012. Investigative Ophthalmology & Visual Science. , volume: 53, issue: 1, pages: 235-40. Doi: https://dx.doi.org/10.1167/iovs.11-8493. | Ethiopia | Cross-sectional within RCT | TF | TI, TF and/or TI | no | yes | no | no | Both | Yes | Yes | Yes | No | Unclear | Yes | Yes | Yes | Unclear | Random | Yes | Yes | Unclear | Yes | Yes | Yes | Yes | Yes | Yes | [6/9] (67%) | [8/10] (80%) | 80% |
| Keenan, J. D.; Tadesse, Z.; Gebresillasie, S.; Shiferaw, A.; Zerihun, M.; Emerson, P. M.; Callahan, K.; Cotter, S. Y.; Stoller, N. E.; Porco, T. C.; Oldenburg, C. E.; Lietman, T. M.Mass azithromycin distribution for hyperendemic trachoma following a cluster-randomized trial: A continuation study of randomly reassigned subclusters (TANA II). 2018. PLoS Medicine / Public Library of Science. , volume: 15, issue: 8, pages: e1002633. Doi: https://dx.doi.org/10.1371/journal.pmed.1002633. | Ethiopia | Cross-sectional within RCT | TF | no | no | yes | no | no | Checklist for Prevalence Studies | Yes | Yes | Yes | Yes | Unclear | Yes | Yes | Yes | Unclear | | | | | | | | | | | [7/9] (78%) | NA | 78% |
| Ketema, K.; Tiruneh, M.; Woldeyohannes, D.; Muluye, D.Active trachoma and associated risk factors among children in Baso Liben District of East Gojjam, Ethiopia. 2012. BMC Public Health. , volume: 12, issue: , pages: 1105. Doi: . | Ethiopia | Cross-sectional | TF | TI, TF and/or TI | no | no | no | no | Checklist for Prevalence Studies | Yes | Yes | Unclear | Yes | Unclear | Yes | Yes | Yes | Unclear | | | | | | | | | | | [6/9] (67%) | NA | 67% |
| Khandekar, R.; Mohammed, A. J.Outcome of azithromycin treatment of active trachoma in Omani schoolchildren. 2003. Eastern Mediterranean Health Journal. , volume: 9, issue: 45052, pages: 1026-1033. Doi: . | Oman | Case-control | TF | TI | no | no | no | no | Checklist for Prevalence Studies | Yes | Yes | Yes | Yes | Unclear | Yes | Yes | Yes | Unclear | | | | | | | | | | | [7/9] (78%) | NA | 78% |
| Khandekar, R.; Mabry, R.; Al-Hadrami, K.; Sarvanan, N.Active trachoma, face washing (F) and environmental improvement (E) in a high-risk population in Oman. 2005. Eastern Mediterranean Health Journal. , volume: 11, issue: 3, pages: 402-409. Doi: . | Oman | Cross-sectional | TF | TI | no | no | no | no | Checklist for Prevalence Studies | No | No | Unclear | Yes | Yes | Yes | Yes | Yes | Yes | | | | | | | | | | | [6/9] (67%) | NA | 67% |
| Sumeet, Khanduja; Vishal, Jhanji; Namrata, Sharma; Praveen, Vashist; Murthy, G. V. S.; Sanjeev, Gupta; Gita, Satpathy; Radhika, Tandon; Titiyal, J. S.; Vajpayee, R. B.Rapid assessment of trachoma among children living in rural Northern India. 2009. Ophthalmic Epidemiology. , volume: 16, issue: 4, pages: 206-211. Doi: http://dx.doi.org/10.1080/09286580902999389. | India | Cross-sectional | TF | TI, TF and/or TI | no | yes | no | no | Checklist for Prevalence Studies | No, using TRA methods, sampled most-likely to be positive areas | Yes | Yes | Yes | Unclear | Yes | Yes | Yes | Unclear | | | | | | | | | | | [6/9] (67%) | NA | 67% |
| Kim, J. S.; Oldenburg, C. E.; Cooley, G.; Amza, A.; Kadri, B.; Nassirou, B.; Cotter, S. Y.; Stoller, N. E.; West, S. K.; Bailey, R. L.; Keenan, J. D.; Gaynor, B. D.; Porco, T. C.; Lietman, T. M.; Martin, D. L.Community-level chlamydial serology for assessing trachoma elimination in trachoma-endemic Niger. 2019. PLoS Neglected Tropical Diseases. , volume: 13, issue: 1, pages: e0007127. Doi: http://dx.doi.org/10.1371/journal.pntd.0007127. | Niger | Cross-sectional | TF | TI | no | no | yes | no | Both | Yes | Yes | Unclear | Yes | Unclear | Yes | Yes | Yes | Unclear | random | Yes | Yes | Unclear | Yes | Yes | Yes | Yes | Yes | Yes | [6/9] (67%) | [8/10] (80%) | 80% |
| King, J. D.; Ngondi, J.; Gatpan, G.; Lopidia, B.; Becknell, S.; Emerson, P. M.The burden of trachoma in Ayod County of Southern Sudan. 2008. PLoS Neglected Tropical Diseases. , volume: 2, issue: 9, pages: e299. Doi: http://dx.doi.org/10.1371/journal.pntd.0000299. | South Sudan | Cross-sectional | TF | TI, TF and/or TI | no | no | no | no | Checklist for Prevalence Studies | Yes | Yes | Yes | Yes | Yes | Yes | Yes | Yes | Yes | | | | | | | | | | | [9/9] (100%) | NA | 100% |
| King, J. D.; Jip, N.; Jugu, Y. S.; Othman, A.; Rodgers, A. F.; Dajom, D. Y.; Miri, E.; Emerson, P. M.Mapping trachoma in Nasarawa and Plateau States, central Nigeria. 2010. British Journal of Ophthalmology. , volume: 94, issue: 1, pages: 14-19. Doi: http://dx.doi.org/10.1136/bjo.2009.165282. | Nigeria | Cross-sectional | TF | TI, TF and/or TI | no | no | no | no | Checklist for Prevalence Studies | Yes | Yes | Yes | Yes | Yes | Yes | Yes | Yes | Yes | | | | | | | | | | | [9/9] (100%) | NA | 100% |
| King, J. D.; Odermatt, P.; Utzinger, J.; Ngondi, J.; Bamani, S.; Kamissoko, Y.; Boubicar, K.; Hassan, A. S.; Nwobi, B. C.; Jip, N.; Amnie, A.; Teferi, T.; Mosher, A. W.; Stewart, A. E. P.; Cromwell, E. A.; Emerson, P. M.Trachoma among children in community surveys from four African countries and implications of using school surveys for evaluating prevalence. 2013. International Health (RSTMH). , volume: 5, issue: 4, pages: 280-287. Doi: http://dx.doi.org/10.1093/inthealth/iht027. | Ethiopia, Mali, Niger, Nigeria | Cross-sectional | TF | TI | no | no | no | no | Checklist for Prevalence Studies | Yes | Yes | Unclear | Yes | Unclear | Yes | Yes | Yes | Unclear | | | | | | | | | | | [6/9] (67%) | NA | 67% |
| King, J. D.; Teferi, T.; Cromwell, E. A.; Zerihun, M.; Ngondi, J. M.; Damte, M.; Ayalew, F.; Tadesse, Z.; Gebre, T.; Mulualem, A.; Karie, A.; Melak, B.; Adugna, M.; Gessesse, D.; Worku, A.; Endeshaw, T.; Ayele, F. A.; Stoller, N. E.; King, M. R. A.; Mosher, A. W.; Gebregzabher, T.; Haileysus, G.; Odermatt, P.; Utzinger, J.; Emerson, P. M.Prevalence of trachoma at sub-district level in Ethiopia: determining when to stop mass azithromycin distribution. 2014. PLoS Neglected Tropical Diseases. , volume: 8, issue: 3, pages: e2732. Doi: . | Ethiopia | Cross-sectional | TF | TI | no | no | no | no | Checklist for Prevalence Studies | Yes | Yes | Yes | Yes | Yes | Yes | Yes | Yes | Yes | | | | | | | | | | | [9/9] (100%) | NA | 100% |
| Koroma, J. B.; Heck, E.; Vandy, M.; Sonnie, M.; Hodges, M.; MacArthur, C.; Sankara, D. P.The epidemiology of trachoma in the five northern districts of Sierra Leone. 2011. Ophthalmic Epidemiology. , volume: 18, issue: 4, pages: 150-157. Doi: http://dx.doi.org/10.3109/09286586.2011.594204. | Sierra Leone | Cross-sectional | TF | TF and/or TI | no | no | no | no | Checklist for Prevalence Studies | Yes | Yes | Yes | Yes | Yes | Yes | Yes | Yes | Yes | | | | | | | | | | | [9/9] (100%) | NA | 100% |
| Koukounari, A.; Moustaki, I.; Blake, I. M.; Donnelly, C. A.; Alexander, N. D. E.; Harding-Esch, E. M.; Holland, M. J.; Basanez, M. G.; Manoj, G.; Mabey, D. C.; Bailey, R.; Grassly, N. C.; Solomon, A. W.Evaluation of diagnostics of chlamydia trachomatis infection in Kahe Mpya subvillage, Rombo District, Tanzania: A latent variable modeling approach. 2011. American Journal of Tropical Medicine and Hygiene. , volume: 1), issue: , pages: 168. Doi: . | Tanzania | Cross-sectional | TF | TI | no | yes | no | no | Checklist for Prevalence Studies | Unclear | Unclear | Unclear | No | Unclear | Unclear | Unclear | Unclear | Unclear | | | | | | | | | | | [0/9] (0%) | NA | 0% |
| Kur, L. W.; Picon, D.; Adibo, O.; Robinson, E.; Sabasio, A.; Edwards, T.; Ndyaba, A.; Rumunu, J.; Lewis, K.; Lado, M.; Kolaczinski, J.Trachoma in Western Equatoria State, Southern Sudan: implications for national control. 2009. PLoS Neglected Tropical Diseases. , volume: 3, issue: 7, pages: e492. Doi: http://dx.doi.org/10.1371/journal.pntd.0000492. | South Sudan | Cross-sectional | TF | TI | no | no | no | no | Checklist for Prevalence Studies | Yes | Yes | Yes | Yes | Yes | Yes | Yes | Yes | Yes | | | | | | | | | | | [9/9] (100%) | NA | 100% |
| Laming, A. C.; Hallsworth, P. J.Detection of chlamydial nucleic acid in follicular trachoma. 1999. Medical Journal of Australia. , volume: 170, issue: 4, pages: 190. Doi: . | Australia | Cross-sectional | TF | no | no | yes | no | no | Checklist for DTA studies | | | | | | | | | | Unclear | Unclear | Unclear | Unclear | Unclear | Unclear | Yes | Unclear | Unclear | Unclear | NA | [1/10] (10%) | 10% |

S6. Table of included studies

| | | | | | | | | | | Risk of Bias checklist items** | | | | | | | | | | | | | | | | | | | | | |
|---|---|---|---|---|---|---|---|---|---|---|---|---|---|---|---|---|---|---|---|---|---|---|---|---|---|---|---|---|---|---|---|
| reference | Country or countries of data collection | Type of study | TF or FPC | other clinical indicators? | photo-graded TF or FPC? | infection? | serology? | SCR? | Critical Appraisal Tool used** | Prevalence study: Was the sample frame appropriate to address the target pop? | Prevalence study: Were study participants sampled in an appropriate way? | Prevalence study: Was the sample size adequate? | Prevalence study: Were the study subjects and setting described in detail? | Prevalence study: Was the data analysis conducted with sufficient coverage of the identified sample? | Prevalence study: Were valid methods used for the identification of the condition(s)? | Prevalence study: Was the condition measured in a standard, reliable way for all participants? | Prevalence study: Was there appropriate statistical analysis? | Prevalence study: Was the response rate adequate, and if not, was the low response rate managed appropriately? | DTA study: Was a consecutive or random sample of patients enrolled? | DTA study: Was a case-control design avoided? | DTA study: Did the study avoid inappropriate exclusions? | DTA: Were the index test results interpreted without knowledge of the results of the reference standard? | DTA: If a threshold was used, was it pre-specified? | DTA: Is the reference standard likely to correctly classify the target condition? | DTA: Were the reference standard results interpreted without knowledge of the results of the index test? | DTA: Was there an appropriate interval between index test and reference standard? | DTA: Did all patients receive the same reference standard? | DTA: Were all patients included in the analysis? | Prevalence study score [meets criteria/total] (%) | DTA study score [meets criteria/total] (%) | Max score (if both checklists used, higher of 2 scores) |
| Last, A. R.; Burr, S. E.; Weiss, H. A.; Harding-Esch, E. M.; Cassama, E.; Nabicassa, M.; Mabey, D. C.; Holland, M. J.; Bailey, R. L.Risk factors for active trachoma and ocular Chlamydia trachomatis infection in treatment-naive trachoma-hyperendemic communities of the Bijagos Archipelago, Guinea Bissau. 2014. PLoS Neglected Tropical Diseases. , volume: 8, issue: 6, pages: e2900. Doi: http://dx.doi.org/10.1371/journal.pntd.0002900. | Guinea Bissau | Cross-sectional | TF | TI, TF and/or TI | no | yes | no | no | Checklist for Prevalence Studies | Yes | Yes | Yes | Yes | Unclear | Yes | Yes | Yes | Unclear | | | | | | | | | | | [7/9] (78%) | NA | 78% |
| Last, A.; Cassama, E.; Bojang, E.; Nabicassa, M.; Burr, S.; Thompson, K.; Haddad, D.; Mabey, D.; Holland, M.; Bailey, R.Trachoma elimination in an endemic island setting in West Africa: Are two doses of oral azithromycin better than one?. 2015. American Journal of Tropical Medicine and Hygiene. , volume: 93(4 Supplement), issue: , pages: 403-404. Doi: . | Guinea Bissau | Cross-sectional within RCT | TF | no | no | yes | no | no | Checklist for Prevalence Studies | Unclear | Unclear | Unclear | No | Unclear | Unclear | Yes | Yes | Unclear | | | | | | | | | | | [2/9] (22%) | NA | 22% |
| Last, A. R.; Burr, S. E.; Harding-Esch, E.; Cassama, E.; Nabicassa, M.; Roberts, C. H.; Mabey, D. C. W.; Holland, M. J.; Bailey, R. L.The impact of a single round of community mass treatment with azithromycin on disease severity and ocular Chlamydia trachomatis load in treatment-naive trachoma-endemic island communities in West Africa. 2017. Parasites and Vectors. , volume: 10, issue: 624, pages: . Doi: http://dx.doi.org/10.1186/s13071-017-2566-x. | Guinea Bissau | Cross-sectional | TF | TI | no | yes | no | no | Checklist for Prevalence Studies | Yes | Yes | Yes | No | Yes | Yes | Yes | Yes | Yes | | | | | | | | | | | [8/9] (89%) | NA | 89% |
| Lee, J.; Munoz, B.; Mkocha, H.; Gaydos, C.; West, S.Decline of ocular Chlamydia trachomatis infection and follicular trachoma with annual rounds of mass treatment in mesoendemic communities in Tanzania. 2013. Investigative Ophthalmology and Visual Science. Conference. , volume: 54, issue: 15, pages: . Doi: . | Tanzania | Cross-sectional | TF | no | no | yes | no | no | Checklist for Prevalence Studies | Unclear | Unclear | Unclear | No | Unclear | Yes | Yes | Yes | Unclear | | | | | | | | | | | [3/9] (33%) | NA | 33% |
| Lee, J. S.; Munoz, B. E.; Mkocha, H.; Gaydos, C. A.; Quinn, T. C.; West, S. K.The effect of multiple rounds of mass drug administration on the association between ocular Chlamydia trachomatis infection and follicular trachoma in preschool-aged children. 2014. PLoS Neglected Tropical Diseases. , volume: 8, issue: 4, pages: e2761. Doi: http://dx.doi.org/10.1371/journal.pntd.0002761. | Tanzania | Cross-sectional | TF | TI | no | yes | no | no | Checklist for Prevalence Studies | Yes | Yes | Unclear | No | Unclear | Yes | Yes | Yes | Unclear | | | | | | | | | | | [5/9] (56%) | NA | 56% |
| Liang, QingFeng; Lu, XinXin; Wang, Mei; Tian, Lei; Labbe, A.; Hu, AiLianStudy of infectious conjunctivitis among children in rural areas of Qinghai province. 2016. Special Issue: Etiology and prevention of trachoma.. , volume: 59, issue: 6, pages: 548-554. Doi: http://dx.doi.org/10.1007/s11427-016-5058-x. | China | Cross-sectional | TF | TI | no | yes | no | no | Checklist for Prevalence Studies | Unclear | Yes | Yes | Yes | Yes | Yes | Yes | Yes | Yes | | | | | | | | | | | [8/9] (89%) | NA | 89% |
| Liu, F. C.; Porco, T. C.; Amza, A.; Kadri, B.; Nassirou, B.; West, S. K.; Bailey, R. L.; Keenan, J. D.; Lietman, T. M.Short-term forecasting of the prevalence of clinical trachoma: utility of including delayed recovery and tests for infection. 2015. Parasites and Vectors. , volume: 8, issue: 535, pages: . Doi: . | Niger | Cross-sectional within longitudinal | TF | TI | no | yes | no | no | Checklist for Prevalence Studies | Yes | Yes | Yes | Yes | Yes | Yes | Yes | Yes | Yes | | | | | | | | | | | [9/9] (100%) | NA | 100% |
| Luna, E. J. de A.; Lopes, M. de F. C.; Medina, N. H.; Favacho, J.; Cardoso, M. R. A.Prevalence of trachoma in schoolchildren in Brazil. 2016. Ophthalmic Epidemiology. , volume: 23, issue: 6, pages: 360-365. Doi: http://dx.doi.org/10.1080/09286586.2016.1244274. | Brazil | Cross-sectional | TF | TI | no | no | no | no | Checklist for Prevalence Studies | Unclear, school-based | Yes | Yes | Yes | Yes | Yes | Yes | Yes | Yes | | | | | | | | | | | [8/9] (89%) | NA | 89% |
| Lynch, K. D.; Morotti, W.; Brian, G.; Ketchup, L.; Kingston, K.; Starr, M.; Ware, R. S.; Everill, B.; Asgar, N.; O'Keefe, A.; Whop, L. J.; Kaldor, J. M.; Lambert, S. B.Clinical signs of trachoma and laboratory evidence of ocular Chlamydia trachomatis infection in a remote Queensland community: a serial cross-sectional study. 2022. Medical Journal of Australia.. , volume: , issue: , pages: . Doi: https://dx.doi.org/10.5694/mja2.51735. | Australia | Cross-sectional | TF | <5 follicles | no | yes | yes | no | Both | Yes | Unclear | Yes | Yes | Yes | Yes | Yes | Yes | No | Unclear | Yes | Yes | Unclear | Yes | Yes | Yes | Yes | Yes | Yes | [7/9] (78%) | [8/10] (80%) | 80% |
| Lynch, K. D.; Apadinuwe, S. C.; Lambert, S. B.; Hillgrove, T.; Starr, M.; Catlett, B.; Ware, R. S.; Cama, A.; Webster, S.; Harding-Esch, E. M.; Bakhtiari, A.; Butcher, R.; Cunningham, P.; Martin, D.; Gwyn, S.; Solomon, A. W.; Garabwan, C.; Kaldor, J. M.; Nery, S. V.A national survey integrating clinical, laboratory, and WASH data to determine the typology of trachoma in Nauru. 2022. PLoS Neglected Tropical Diseases. , volume: 16, issue: 4, pages: . Doi: http://dx.doi.org/10.1371/journal.pntd.0010275. | Nauru | Cross-sectional | TF | no | no | yes | yes | yes | Both | Yes | Yes | Yes | Yes | Yes | Yes | Yes | Yes | Yes | Random | Yes | Yes | Yes | Yes | Yes | Yes | Yes | Yes | Yes | [9/9] (100%) | [9/10] (90%) | 100% |
| Lynch, K. D.; Brian, G.; Ahwang, T.; Newie, T.; Newie, V.; Perrett, C.; Wharton, G.; Brown, A.; Tozer, S.; Kaldor, J. M.; Whop, L. J.; Andrews, R. M.; Lambert, S. B.Discord between presence of follicular conjunctivitis and Chlamydia trachomatis infection in a single Torres Strait Island community: a cross-sectional survey. 2022. Australian and New Zealand journal of public health. , volume: 46, issue: 2, pages: 155-160. Doi: http://dx.doi.org/10.1111/1753-6405.13179. | Australia | Cross-sectional | TF | <5 follicles | no | yes | no | no | Checklist for Prevalence Studies | Yes | Yes | Yes | Yes | Yes | Yes | Yes | Yes | Yes | | | | | | | | | | | [9/9] (100%) | NA | 100% |
| Mabey, D. C.; Bailey, R. L.; Dunn, D.; Jones, D.; Williams, J. H.; Whittle, H. C.; Ward, M. E.Expression of MHC class II antigens by conjunctival epithelial cells in trachoma: implications concerning the pathogenesis of blinding disease. 1991. Journal of Clinical Pathology. , volume: 44, issue: 4, pages: 285-9. Doi: . | Gambia | Case-control | F2/F3 | P3 | no | no | no | no | Checklist for Prevalence Studies | Unclear | Unclear | Unclear | Yes | Unclear | Yes | Yes | Yes | Unclear | | | | | | | | | | | [4/9] (44%) | NA | 44% |
| Macleod, C. K.; Binnawi, K. H.; Elshafie, B. E.; Sadig, H. E.; Hassan, A.; Cocks, N.; Willis, R.; Chu, B.; Solomon, A. W.Unimproved water sources and open defecation are associated with active trachoma in children in internally displaced persons camps in the Darfur States of Sudan. 2019. Transactions of the Royal Society of Tropical Medicine and Hygiene. , volume: 113, issue: 10, pages: 599-609. Doi: http://dx.doi.org/10.1093/trstmh/trz042. | Sudan | Cross-sectional | TF | TI | no | no | no | no | Checklist for Prevalence Studies | Yes | Yes | Yes | Yes | Yes | Yes | Yes | Yes | Yes | | | | | | | | | | | [9/9] (100%) | NA | 100% |
| Macleod, C. K.; Butcher, R.; Javati, S.; Gwyn, S.; Jonduo, M.; Abdad, M. Y.; Roberts, C. H.; Keys, D.; Koim, S. P.; Ko, R.; Garap, J.; Pahau, D.; Houinei, W.; Martin, D. L.; Pomat, W. S.; Solomon, A. W.Trachoma, anti-Pgp3 serology, and ocular Chlamydia trachomatis infection in Papua New Guinea. 2020. Clinical Infectious Diseases. , volume: 72, issue: 3, pages: 423-430. Doi: http://dx.doi.org/10.1093/cid/ciaa042. | Papua New Guinea | Cross-sectional | TF | no | no | yes | yes | yes | Checklist for Prevalence Studies | Yes | Yes | Yes | Yes | Unclear | Yes | Yes | Yes | Unclear | | | | | | | | | | | [7/9] (78%) | NA | 78% |
| Madani, M. O.; Huguet, P.; Mariotti, S. P.; Dezoumbe, D.; Tosi, C.; Djada, D.; Negrel, A. D.Trachoma in Northern Chad: Epidemiological survey. [French]. 2003. Cahiers Sante. , volume: 13(1), issue: , pages: 45184. Doi: . | Chad | Cross-sectional | TF | TI | no | no | no | no | Checklist for Prevalence Studies | Unclear | Unclear | Unclear | No | Yes | Yes | Yes | Unclear | Yes | | | | | | | | | | | [4/9] (44%) | NA | 44% |
| Sumit, Malhotra; Praveen, Vashist; Noopur, Gupta; Mani, Kalaivani; Gita, Satpathy; Anita, Shah; Sujaya, Krishnan; Rajvardhan, AzadPrevalence of trachoma in Car-Nicobar Island, India after three annual rounds of mass drug administration with azithromycin. 2016. PLoS ONE [Electronic Resource]. , volume: 11, issue: 7, pages: e0158625. Doi: http://dx.doi.org/10.1371/journal.pone.0158625. | India | Cross-sectional | TF | TI | no | no | no | no | Checklist for Prevalence Studies | Yes | Yes | Yes | Yes | Unclear | Yes | Yes | Yes | Yes | | | | | | | | | | | [8/9] (89%) | NA | 89% |
| Martin, D. L.; Wiegand, R.; Goodhew, B.; Lammie, P.; Black, C. M.; West, S.; Gaydos, C. A.; Dize, L.; Mkocha, H.; Kasubi, M.; Gambhir, M.Serological Measures of Trachoma Transmission Intensity. 2015. Scientific Reports. , volume: 5, issue: , pages: 18532. Doi: https://dx.doi.org/10.1038/srep18532. | Tanzania | Cross-sectional | TF | no | no | yes | yes | no | Checklist for Prevalence Studies | Unclear | Unclear | Unclear | No | Unclear | Yes | Yes | Yes | Unclear | | | | | | | | | | | [3/9] (33%) | NA | 33% |
| Martin, D. L.; Bid, R.; Sandi, F.; Goodhew, E. B.; Massae, P. A.; Lasway, A.; Philippin, H.; Makupa, W.; Molina, S.; Holland, M. J.; Mabey, D. C. W.; Drakeley, C.; Lammie, P. J.; Solomon, A. W.Serology for trachoma surveillance after cessation of mass drug administration. 2015. PLoS Neglected Tropical Diseases. , volume: 9, issue: 2, pages: e0003555. Doi: http://dx.doi.org/10.1371/journal.pntd.0003555. | Tanzania | Cross-sectional | TF | TI | no | yes | yes | yes | Checklist for Prevalence Studies | Yes | Yes | Yes | Yes | Low coverage of total pop (58%), coverage in 1-9 was higher (72%) | Yes | Yes | Yes | No | | | | | | | | | | | [7/9] (78%) | NA | 78% |
| Mathew, A. A.; Keeffe, J. E.; Mesurier, R. T. le; Taylor, H. R.Trachoma in the Pacific Islands: evidence from Trachoma Rapid Assessment. 2009. British Journal of Ophthalmology. , volume: 93, issue: 7, pages: 866-870. Doi: http://dx.doi.org/10.1136/bjo.2008.151720. | Fiji, Kiribati, Nauru, Solomon Islands, Vanuatu | Cross-sectional | TF | TI | no | no | no | no | Checklist for Prevalence Studies | Yes | No, TRA | No | Yes | Low coverage (<80%) | Yes | Yes | Yes | Unclear | | | | | | | | | | | [5/9] (56%) | NA | 56% |
| Medina, N. H.; Massaini, M. G.; Azevedo, C. L. B.; Harima, C.; Prado, M.; Maluf, S.; Marcucci, M.; Caligaris, L. S. A.; Morimoto, W. T. M.Epidemiological surveillance of trachoma in a school in the city of Sao Paulo, SP. 1998. Vigilancia epidemiologica do tracoma em instituicao de ensino na cidade de Sao Paulo, SP.. , volume: 32, issue: 1, pages: 59-63. Doi: http://dx.doi.org/10.1590/S0034-89101998000100008. | Brazil | Cross-sectional | TF | TF and/or TI | no | no | no | no | Checklist for Prevalence Studies | Yes | Yes | Yes | No | Yes | Yes | Yes | Yes | Yes | | | | | | | | | | | [8/9] (89%) | NA | 89% |
| Meng, N.; Seiha, D.; Thorn, P.; Willis, R.; Flueckiger, R. M.; Dejene, M.; Lewallen, S.; Courtright, P.; Solomon, A. W.Assessment of trachoma in Cambodia: trachoma is not a public health problem. 2016. Special Issue: Trachoma mapping.. , volume: 23, issue: Suppl. 1, pages: 44992. Doi: . | Cambodia | Cross-sectional | TF | TI | no | no | no | no | Checklist for Prevalence Studies | Yes | Yes | Yes | No | Yes | Yes | Yes | Yes | Yes | | | | | | | | | | | [8/9] (89%) | NA | 89% |
| Mesfin, M. M.; Camera, J. de la; Tareke, I. G.; Amanual, G.; Araya, T.; Kedir, A. M.A community-based trachoma survey: prevalence and risk factors in the Tigray region of Northern Ethiopia. 2006. Ophthalmic Epidemiology. , volume: 13, issue: 3, pages: 173-181. Doi: http://dx.doi.org/10.1080/09286580600611427. | Ethiopia | Cross-sectional | TF | TI | no | no | no | no | Checklist for Prevalence Studies | Yes | Yes | Unclear | Yes | Yes | Yes | Yes | Yes | Yes | | | | | | | | | | | [8/9] (89%) | NA | 89% |
| Michel, C. E. C.; Solomon, A. W.; Magbanua, J. P.; Massae, P. A.; Huang, L.; Mosha, J.; West, S. K.; Nadala, E. C.; Bailey, R.; Wisniewski, C.; Mabey, D. C.; Lee, H. H.Field evaluation of a rapid point-of-care assay for targeting antibiotic treatment for trachoma control: a comparative study. 2006. Lancet. , volume: 367(9522), issue: , pages: 1585-1590. Doi: https://dx.doi.org/10.1016/S0140-6736%2806%2968695-9. | Tanzania | Cross-sectional | TF | no | no | yes | no | no | Both | Yes | Unclear | Unclear | No | Yes | Yes | Yes | Yes | Yes | Unclear | Yes | Yes | Unclear | Yes | Yes | Yes | Yes | Yes | Yes | [6/9] (67%) | [8/10] (80%) | 80% |
| Michel, C. E. C.; Roper, K. G.; Divena, M. A.; Lee, H. H.; Taylor, H. R.Correlation of clinical trachoma and infection in aboriginal communities. 2011. PLoS Neglected Tropical Diseases. , volume: 5, issue: 3, pages: e986. Doi: http://dx.doi.org/10.1371/journal.pntd.0000986. | Australia | Cross-sectional | TF | no | no | yes | no | no | Checklist for Prevalence Studies | Yes | Yes | Yes | Yes | Yes | Yes | Yes | Yes | Yes | | | | | | | | | | | [9/9] (100%) | NA | 100% |

S6. Table of included studies

| | | | | | | | | | | Risk of Bias checklist items** | | | | | | | | | | | | | | | | | | | | | |
|---|---|---|---|---|---|---|---|---|---|---|---|---|---|---|---|---|---|---|---|---|---|---|---|---|---|---|---|---|---|---|---|
| reference | Country or countries of data collection | Type of study | TF or FPC | other clinical indicators? | photo-graded TF or FPC? | infection? | serology? | SCR? | Critical Appraisal Tool used** | Prevalence study: Was the sample frame appropriate to address the target pop? | Prevalence study: Were study participants sampled in an appropriate way? | Prevalence study: Was the sample size adequate? | Prevalence study: Were the study subjects and setting described in detail? | Prevalence study: Was the data analysis conducted with sufficient coverage of the identified sample? | Prevalence study: Were valid methods used for the identification of the condition(s)? | Prevalence study: Was the condition measured in a standard, reliable way for all participants? | Prevalence study: Was there appropriate statistical analysis? | Prevalence study: Was the response rate adequate, and if not, was the low response rate managed appropriately? | DTA study: Was a consecutive or random sample of patients enrolled? | DTA study: Was a case-control design avoided? | DTA study: Did the study avoid inappropriate exclusions? | DTA: Were the index test results interpreted without knowledge of the results of the reference standard? | DTA: If a threshold was used, was it pre-specified? | DTA: Is the reference standard likely to correctly classify the target condition? | DTA: Were the reference standard results interpreted without knowledge of the results of the index test? | DTA: Was there an appropriate interval between index test and reference standard? | DTA: Did all patients receive the same reference standard? | DTA: Were all patients included in the analysis? | Prevalence study score [meets criteria/total] (%) | DTA study score [meets criteria/total] (%) | Max score (if both checklists used, higher of 2 scores) |
| Migchelsen, S. J.; Martin, D. L.; Southisombath, K.; Turyaguma, P.; Heggen, A.; Rubangakene, P. P.; Joof, H.; Makalo, P.; Cooley, G.; Gwyn, S.; Solomon, A. W.; Holland, M. J.; Courtright, P.; Willis, R.; Alexander, N. D. E.; Mabey, D. C. W.; Roberts, C. H.Defining seropositivity thresholds for use in trachoma elimination studies. 2017. PLoS Neglected Tropical Diseases. , volume: 11, issue: 1, pages: e0005230. Doi: http://dx.doi.org/10.1371/journal.pntd.0005230. | Gambia, Laos, Uganda | Cross-sectional | TF | TI | no | no | yes | yes | Both | Yes | Yes | Unclear | Yes | Unclear | Yes | Yes | Yes | Unclear | Random | Yes | Yes | Yes | Yes | Yes | Yes | Yes | Yes | Yes | [6/9] (67%) | [9/10] (90%) | 90% |
| Alfonso Miller, H.; Lopez Mesa, C. B. de; Talero, S. L.; Meza Cardenas, M.; Ramirez, S. P.; Moreno-Montoya, J.; Porras, A.; Trujillo-Trujillo, J.Prevalence of trachoma and associated factors in the rural area of the department of Vaupes, Colombia. 2020. PLoS ONE [Electronic Resource]. , volume: 15, issue: 5, pages: . Doi: http://dx.doi.org/10.1371/journal.pone.0229297. | Colombia | Cross-sectional | TF | TI | no | no | no | no | Checklist for Prevalence Studies | Yes | Yes | Unclear | Yes | Yes | Yes | Yes | Yes | Yes | | | | | | | | | | | [8/9] (89%) | NA | 89% |
| Miller, K.; Schmidt, G.; Melese, M.; Alemayehu, W.; Yi, E.; Cevallos, V.; Donnellan, C.; Olinger, L.; Fantaye, D.; Gaynor, B.; Whitcher, J. P.; Lietman, T.How reliable is the clinical exam in detecting ocular chlamydial infection?. 2004. Ophthalmic Epidemiology. , volume: 11, issue: 3, pages: 255-62. Doi: . | Ethiopia | Cross-sectional | TF | TI, TF and/or TI, TF and TI | no | yes | no | no | Checklist for DTA studies | | | | | | | | | | Random | Yes | Yes | Yes | Yes | Yes | Yes | Yes | Yes | Yes | NA | [9/10] (90%) | 90% |
| Miller, H.; Gallego, G.; Rodriguez, G.Clinical evidence of trachoma in Colombian Amerindians of the Vaupes Province. [Spanish]. 2010. Biomedica. , volume: 30(3), issue: , pages: 432-439. Doi: . | Colombia | Cross-sectional | TF | TI | no | no | no | no | Checklist for Prevalence Studies | Yes | Yes | No | Yes | No | Yes | Yes | Yes | No | | | | | | | | | | | [6/9] (67%) | NA | 67% |
| Missamou, F.; Marlhand, H.; Dzabatou-Babeaux, A. S. P.; Sendzi, S.; Bernasconi, J.; D'Souza, S.; Bakhtiari, A.; Millar, T.; Willis, R.; Bengraine, K.; Resnikoff, S.; Solomon, A. W.A population-based trachoma prevalence survey covering seven districts of Sangha and Likouala departments, Republic of the Congo. 2018. Special Issue: 2017 Global Trachoma Mapping Project.. , volume: 25, issue: Suppl. 1, pages: 155-161. Doi: http://dx.doi.org/10.1080/09286586.2018.1546878. | Republic of the Congo | Cross-sectional | TF | TI | no | no | no | no | Checklist for Prevalence Studies | Yes | Yes | Yes | No | Yes | Yes | Yes | Yes | Yes | | | | | | | | | | | [8/9] (89%) | NA | 89% |
| Mkocha, H.; Munoz, B.; West, S.Trachoma and ocular Chlamydia trachomatis rates in children in trachoma-endemic communities enrolled for at least three years in the Tanzania National Trachoma Control Programme. 2009. Tanzania journal of health research. , volume: 11, issue: 3, pages: 103-110. Doi: . | Tanzania | Cross-sectional | TF | TI | no | yes | no | no | Checklist for Prevalence Studies | Yes | Yes | Yes | No | Unclear | Yes | Yes | Yes | Unclear | | | | | | | | | | | [6/9] (67%) | NA | 67% |
| Mohammadi, S. F.; Bazi-Shad, A.; Akbarizadeh, J.; Ashrafi, E.; Moinfar, Z.; Shirdel, E.; Nabavi, M.; Samiei, S.; Salehi-Rad, S.; Solomon, A. W.Follow-up and report on active trachoma in Zabol, Iran, prompted by Sharifi-Rad and Fallah's observations published in May 2016. 2017. New Microbes and New Infections. , volume: 20, issue: , pages: 14-15. Doi: https://dx.doi.org/10.1016/j.nmni.2017.08.001. | Iran | Cross-sectional | TF | no | no | yes | no | no | Checklist for Prevalence Studies | Yes | No, only children w/ red eye screened for trachoma | Yes | No | No | Unclear | Yes | Yes | Unclear | | | | | | | | | | | [4/9] (44%) | NA | 44% |
| Mpyet, C.; Ogoshi, C.; Goyol, M.Prevalence of trachoma in Yobe State, North-Eastern Nigeria. 2008. Ophthalmic Epidemiology. , volume: 15, issue: 5, pages: 303-307. Doi: http://dx.doi.org/10.1080/09286580802237633. | Nigeria | Cross-sectional | TF | TI, TF and/or TI | no | no | no | no | Checklist for Prevalence Studies | Yes | Yes | Unclear | Yes | Unclear | Yes | Yes | Yes | Unclear | | | | | | | | | | | [6/9] (67%) | NA | 67% |
| Mpyet, C.; Goyol, M.; Ogoshi, C.Personal and environmental risk factors for active trachoma in children in Yobe state, north-eastern Nigeria. 2010. Tropical Medicine and International Health. , volume: 15, issue: 2, pages: 168-172. Doi: http://dx.doi.org/10.1111/j.1365-3156.2009.02436.x. | Nigeria | Cross-sectional | TF | TI, TF and/or TI | no | no | no | no | Checklist for Prevalence Studies | Yes | Yes | Yes | Yes | Yes | Yes | Yes | Yes | Yes | | | | | | | | | | | [9/9] (100%) | NA | 100% |
| Mpyet, C.; Lass, B. D.; Yahaya, H. B.; Solomon, A. W.Prevalence of and risk factors for trachoma in Kano State, Nigeria. 2012. PLoS ONE [Electronic Resource]. , volume: 7, issue: 7, pages: e40421. Doi: http://dx.doi.org/10.1371/journal.pone.0040421. | Nigeria | Cross-sectional | TF | TI | no | no | no | no | Checklist for Prevalence Studies | Yes | Yes | Yes | Yes | Unclear | Yes | Yes | Yes | Unclear | | | | | | | | | | | [7/9] (78%) | NA | 78% |
| Muhammad, N.; Mpyet, C.; Adamu, M. D.; William, A.; Umar, M. M.; Muazu, H.; Onyebuchi, U.; Isiyaku, S.; Flueckiger, R. M.; Chu, B. K.; Willis, R.; Pavluck, A.; Dalhatu, A.; Ogoshi, C.; Olobio, N.; Gordon, B. A.; Solomon, A. W.Prevalence of trachoma in the Area Councils of the Federal Capital Territory, Nigeria: results of six population-based surveys. 2018. Special Issue: 2017 Global Trachoma Mapping Project.. , volume: 25, issue: Suppl. 1, pages: 45247. Doi: http://dx.doi.org/10.1080/09286586.2017.1367409. | Nigeria | Cross-sectional | TF | TI | no | no | no | no | Checklist for Prevalence Studies | Yes | Yes | Yes | No | Yes | Yes | Yes | Yes | Yes | | | | | | | | | | | [8/9] (89%) | NA | 89% |
| Munoz, B.; Stare, D.; Mkocha, H.; Gaydos, C.; Quinn, T.; West, S. K.Can clinical signs of trachoma be used after multiple rounds of mass antibiotic treatment to indicate infection?. 2011. Investigative Ophthalmology & Visual Science. , volume: 52, issue: 12, pages: 8806-10. Doi: https://dx.doi.org/10.1167/iovs.11-8074. | Tanzania | Cross-sectional | TF | no | yes | yes | no | no | Both | Yes | Yes | Yes | No | Unclear | Yes | Yes | Yes | Unclear | Yes | Yes | Yes | Unclear | Yes | Yes | Yes | Yes | Yes | Yes | [6/9] (67%) | [9/10] (90%) | 90% |
| Nash, S. D.; Sata, E.; Stewart, A. E. P.; Astale, T.; Zerihun, M.; Gessesse, D.; Ayenew, G.; Chanyalew, M.; Melak, B.; Tadesse, Z.; Callahan, E. K.Longitudinal trends in trachoma over eight years in a hyperendemic setting under the safe strategy: Results from serial impact surveys in west gojjam zone, Ethiopia. 2017. American Journal of Tropical Medicine and Hygiene. , volume: 97(5 Supplement 1), issue: , pages: 228. Doi: . | Ethiopia | Cross-sectional | TF | TI | no | yes | no | no | Checklist for Prevalence Studies | Yes | Unclear | Unclear | No | Unclear | Unclear | Yes | Unclear | Unclear | | | | | | | | | | | [2/9] (22%) | NA | 22% |
| Nash, S. D.; Stewart, A. E. P.; Astale, T.; Sata, E.; Zerihun, M.; Gessese, D.; Melak, B.; Ayenew, G.; Ayele, Z.; Bayissasse, B.; Chanyalew, M.; Tadesse, Z.; Kelly Callahan, E.Trachoma prevalence remains below threshold in five districts after stopping mass drug administration: Results of five surveillance surveys within a hyperendemic setting in Amhara, Ethiopia. 2018. Transactions of the Royal Society of Tropical Medicine and Hygiene. , volume: 112(12), issue: , pages: 538-545. Doi: https://dx.doi.org/10.1093/trstmh/try096. | Ethiopia | Cross-sectional | TF | TI | no | yes | no | no | Checklist for Prevalence Studies | Yes | Yes | Unclear | No | Unclear | Yes | Yes | Yes | Unclear | | | | | | | | | | | [5/9] (56%) | NA | 56% |
| Nash, S. D.; Stewart, A. E. P.; Zerihun, M.; Sata, E.; Gessese, D.; Melak, B.; Endeshaw, T.; Chanyalew, M.; Chernet, A.; Bayissasse, B.; Moncada, J.; Lietman, T. M.; Emerson, P. M.; King, J. D.; Tadesse, Z.; Callahan, E. K.Ocular Chlamydia trachomatis infection under the surgery, antibiotics, facial cleanliness, and environmental improvement strategy in Amhara, Ethiopia, 2011-2015. 2018. Clinical Infectious Diseases. , volume: 67, issue: 12, pages: 1840-1846. Doi: http://dx.doi.org/10.1093/cid/ciy377. | Ethiopia | Cross-sectional | TF | TI | no | yes | no | no | Checklist for Prevalence Studies | Yes | Yes | Unclear | Yes | Unclear | Yes | Yes | Yes | Unclear | | | | | | | | | | | [6/9] (67%) | NA | 67% |
| Nash, S. D.; Chernet, A.; Moncada, J.; Stewart, A. E. P.; Astale, T.; Sata, E.; Zerihun, M.; Gessese, D.; Melak, B.; Ayenew, G.; Ayele, Z.; Chanyalew, M.; Lietman, T. M.; Callahan, E. K.; Schachter, J.; Tadesse, Z.Ocular chlamydia trachomatis infection and infectious load among pre-school aged children within trachoma hyperendemic districts receiving the safe strategy, Amhara Region, Ethiopia. 2020. PLoS Neglected Tropical Diseases. , volume: 14(5), issue: , pages: 44942. Doi: https://dx.doi.org/10.1371/journal.pntd.0008226. | Ethiopia | Cross-sectional | TF | TI | no | yes | no | no | Checklist for Prevalence Studies | Yes | Yes | Unclear | Yes | Unclear | Yes | Yes | Yes | Unclear | | | | | | | | | | | [6/9] (67%) | NA | 67% |
| Nash, S. D.; Chernet, A.; Astale, T.; Sata, E.; Zerihun, M.; Nute, A. W.; Jensen, K. A.; Gessese, D.; Ayele, Z.; Melak, B.; Haile, M.; Zeru, T.; Tadesse, Z.; Callahan, E. K.Population-based prevalence of ocular Chlamydia trachomatis infection among infants in the trachoma endemic Amhara region, Ethiopia. 2022. American Journal of Tropical Medicine and Hygiene. , volume: 106, issue: 1, pages: 62-65. Doi: http://dx.doi.org/10.4269/ajtmh.21-0873. | Ethiopia | Cross-sectional | TF | TI | no | yes | no | no | Checklist for Prevalence Studies | Yes | Yes | Unclear | No | Unclear | Yes | Yes | Yes | Unclear | | | | | | | | | | | [5/9] (56%) | NA | 56% |
| Natividad, A.; Freeman, T. C.; Jeffries, D.; Burton, M. J.; Mabey, D. C. W.; Bailey, R. L.; Holland, M. J.Human conjunctival transcriptome analysis reveals the prominence of innate defense in Chlamydia trachomatis infection. 2010. Infection and Immunity. , volume: 78, issue: 11, pages: 4895-4911. Doi: http://dx.doi.org/10.1128/IAI.00844-10. | Gambia | Cross-sectional | F2/F3 | P3 | no | yes | no | no | Checklist for DTA studies | | | | | | | | | | Unclear | No | No, excluded on basis of ability to detect RNA | Unclear | Yes | Yes | Yes | Yes | Yes | No, omitted people without adequate quantity of RNA | NA | [5/10] (50%) | 50% |
| Naufal, F.; Brady, C. J.; Wolle, M. A.; Kashaf, M. S.; Mkocha, H.; Bradley, C.; Kabona, G.; Ngondi, J.; Massof, R. W.; West, S. K.Evaluation of photography using head-mounted display technology (ICAPS) for district trachoma surveys. 2021. PLoS Neglected Tropical Diseases. , volume: 15, issue: 11, pages: . Doi: http://dx.doi.org/10.1371/journal.pntd.0009928. | Tanzania | Cross-sectional | TF | no | yes | no | no | no | Both | Unclear | Unclear | Unclear | No | Unclear | Yes | Yes | Yes | Unclear | Random | Yes | Yes | Yes | Yes | Yes | Yes | Yes | Yes | Yes, although ungradeable images excluded for some analyses | [3/9] (33%) | [8/10] (80%) | 80% |
| Ndisabiye, D.; Gahungu, A.; Kayugi, D.; Waters, E. K.Association of environmental risk factors and trachoma in Gashoho Health district, Burundi. 2020. African Health Sciences. , volume: 20, issue: 1, pages: 182-189. Doi: http://dx.doi.org/10.4314/ahs.v20i1.23. | Burundi | Cross-sectional | TF | TI, TF and/or TI | no | no | no | no | Checklist for Prevalence Studies | Unclear, convenience sample from clinic | Unclear | Yes | No | Yes | Unclear | Yes | Yes | Yes | | | | | | | | | | | [5/9] (56%) | NA | 56% |
| Negrel, A. D.; Khazraji, Y. C.; Akalay, O.Trachoma in the province of Ouarzazate Morocco. [French]. 1992. Bulletin of the World Health Organization. , volume: 70(4), issue: , pages: 451-456. Doi: . | Morocco | Cross-sectional | TF | TF and/or TI | no | no | no | no | Checklist for Prevalence Studies | Yes | Yes | Yes | Yes | Yes | Yes | Yes | Yes | Yes | | | | | | | | | | | [9/9] (100%) | NA | 100% |
| Nesemann, J. M.; Seider, M. I.; Snyder, B. M.; Maamari, R. N.; Fletcher, D. A.; Haile, B. A.; Tadesse, Z.; Varnado, N. E.; Cotter, S. Y.; Callahan, E. K.; Emerson, P. M.; Margolis, T. P.; Lietman, T. M.; Keenan, J. D.Comparison of smartphone photography, single-lens reflex photography, and field-grading for trachoma. 2020. American Journal of Tropical Medicine and Hygiene. , volume: 103(6), issue: , pages: 2488-2491. Doi: https://dx.doi.org/10.4269/ajtmh.20-0386. | Ethiopia | Cross-sectional | TF | TI | yes | no | no | no | Both | Yes | Yes | No | No | Unclear | Yes | Yes | Yes | Unclear | Random | Yes | Yes | Yes | Yes | Yes | Yes | Yes | Yes | Yes | [5/9] (56%) | [9/10] (90%) | 90% |
| Nesemann, J.; Munoz, M. B.; Morocho-Alburqueque, N.; Quincho-Lopez, A.; Wittberg, D. M.; Honorio, H.; Durand, S.; Saboya-Diaz, M. I.; Harding-Esch, E. M.; Talero, S.; Solomon, A. W.; Lescano, A. G.; Keenan, J. D.Prevalence of trachoma in alto amazonas, loreto department, Peru. 2021. Investigative Ophthalmology and Visual Science. Conference: Annual Meeting Association for Research in Vision and Ophthalmology, ARVO. , volume: 62, issue: 8, pages: . Doi: . | Peru | Cross-sectional | TF | no | no | yes | no | no | Checklist for Prevalence Studies | Yes | Yes | Unclear | No | Yes | Yes | Yes | Yes | Yes | | | | | | | | | | | [7/9] (78%) | NA | 78% |

S6. Table of included studies

| reference | Country or countries of data collection | Type of study | TF or FPC | other clinical indicators? | photo-graded TF or FPC? | infection? | serology? | SCR? | Critical Appraisal Tool used** | Risk of Bias checklist items** | | | | | | | | | | | | | | | | | | | Prevalence study score [meets criteria/total] (%) | DTA study score [meets criteria/total] (%) | Max score (if both checklists used, higher of 2 scores) |
|---|---|---|---|---|---|---|---|---|---|---|---|---|---|---|---|---|---|---|---|---|---|---|---|---|---|---|---|---|---|---|---|
| | | | | | | | | | | Prevalence study: Was the sample frame appropriate to address the target pop? | Prevalence study: Were study participants sampled in an appropriate way? | Prevalence study: Was the sample size adequate? | Prevalence study: Were the study subjects and setting described in detail? | Prevalence study: Was the data analysis conducted with sufficient coverage of the identified sample? | Prevalence study: Were valid methods used for the identification of the condition(s)? | Prevalence study: Was the condition measured in a standard, reliable way for all participants? | Prevalence study: Was there appropriate statistical analysis? | Prevalence study: Was the response rate adequate, and if not, was the low response rate managed appropriately? | DTA study: Was a consecutive or random sample of patients enrolled? | DTA study: Was a case-control design avoided? | DTA study: Did the study avoid inappropriate exclusions? | DTA: Were the index test results interpreted without knowledge of the results of the reference standard? | DTA: If a threshold was used, was it pre-specified? | DTA: Is the reference standard likely to correctly classify the target condition? | DTA: Were the reference standard results interpreted without knowledge of the results of the index test? | DTA: Was there an appropriate interval between index test and reference standard? | DTA: Did all patients receive the same reference standard? | DTA: Were all patients included in the analysis? | | | |
| Ngondi, J.; Onsarigo, A.; Adamu, L.; Matende, I.; Baba, S.; Reacher, M.; Emerson, P.; Zingeser, J.The epidemiology of trachoma in Eastern Equatoria and Upper Nile States, southern Sudan. 2005. Bulletin of the World Health Organization. , volume: 83, issue: 12, pages: 904-912. Doi: . | South Sudan | Cross-sectional | TF | TI, TF and/or TI | no | no | no | no | Checklist for Prevalence Studies | Yes | Yes | Yes | Yes | Yes | Yes | Yes | Yes | Yes | | | | | | | | | | | [9/9] (100%) | NA | 100% |
| Ngondi, J.; Onsarigo, A.; Matthews, F.; Reacher, M.; Brayne, C.; Baba, S.; Solomon, A. W.; Zingeser, J.; Emerson, P. M.Effect of 3 years of SAFE (surgery, antibiotics, facial cleanliness, and environmental change) strategy for trachoma control in southern Sudan: a cross-sectional study. 2006. Lancet (British edition). , volume: 368, issue: 9535, pages: 589-595. Doi: http://dx.doi.org/10.1016/S0140-6736(06)69202-7. | South Sudan | Cross-sectional | TF | TI, TF and/or TI | no | no | no | no | Checklist for Prevalence Studies | Yes | Yes | Unclear | Yes | Unclear | Yes | Yes | Yes | Unclear | | | | | | | | | | | [6/9] (67%) | NA | 67% |
| Ngondi, J.; Ole-Sempele, F.; Onsarigo, A.; Matende, I.; Baba, S.; Reacher, M.; Matthews, F.; Brayne, C.; Emerson, P.Blinding trachoma in postconflict southern Sudan. 2006. PLoS Medicine. , volume: 3, issue: 12, pages: e478. Doi: http://dx.doi.org/10.1371/journal.pmed.0030478. | South Sudan | Cross-sectional | TF | TI, TF and/or TI | no | no | no | no | Checklist for Prevalence Studies | Yes | Yes | Yes | Yes | Yes | Yes | Yes | Yes | Yes | | | | | | | | | | | [9/9] (100%) | NA | 100% |
| Ngondi, J.; Teshome, Gebre; Shargie, E. B.; Graves, P. M.; Yeshewamebrat, Ejigsemahu; Tesfaye, Teferi; Asrat, Genet; Mosher, A. W.; Tekola, Endeshaw; Mulat, Zerihun; Ayenew, Messele; Richards, F. O., Jr.; Emerson, P. M.Risk factors for active trachoma in children and trichiasis in adults: a household survey in Amhara Regional State, Ethiopia. 2008. Transactions of the Royal Society of Tropical Medicine and Hygiene. , volume: 102, issue: 5, pages: 432-438. Doi: http://dx.doi.org/10.1016/j.trstmh.2008.02.014. | Ethiopia | Cross-sectional | TF | TI | no | no | no | no | Checklist for Prevalence Studies | Yes | Yes | Yes | Yes | Yes | Yes | Yes | Yes | Yes | | | | | | | | | | | [9/9] (100%) | NA | 100% |
| Ngondi, J.; Gebre, T.; Shargie, E. B.; Adamu, L.; Ejigsemahu, Y.; Teferi, T.; Zerihun, M.; Ayele, B.; Cevallos, V.; King, J.; Emerson, P. M.Evaluation of three years of the SAFE strategy (Surgery, Antibiotics, Facial cleanliness and Environmental improvement) for trachoma control in five districts of Ethiopia hyperendemic for trachoma. 2009. Transactions of the Royal Society of Tropical Medicine and Hygiene. , volume: 103(10), issue: , pages: 1001-1010. Doi: https://dx.doi.org/10.1016/j.trstmh.2008.11.023. | Ethiopia | Cross-sectional | TF | TI, TF and/or TI | no | yes | no | no | Checklist for Prevalence Studies | Yes | Yes | Unclear | Yes | Unclear | Yes | Yes | Yes | Unclear | | | | | | | | | | | [6/9] (67%) | NA | 67% |
| Ngondi, J.; Teshome, Gebre; Shargie, E. B.; Liknaw, Adamu; Tesfaye, Teferi; Mulat, Zerihun; Berhan, Ayele; King, J. D.; Cromwell, E. A.; Emerson, P. M.Estimation of effects of community intervention with Antibiotics, Facial cleanliness, and Environmental improvement (A,F,E) in five districts of Ethiopia hyperendemic for trachoma. 2010. British Journal of Ophthalmology. , volume: 94, issue: 3, pages: 278-281. Doi: http://dx.doi.org/10.1136/bjo.2009.168260. | Ethiopia | Cross-sectional | TF | TI | no | no | no | no | Checklist for Prevalence Studies | Yes | Yes | Unclear | Yes | Unclear | Yes | Yes | Yes | Unclear | | | | | | | | | | | [6/9] (67%) | NA | 67% |
| Nigusie, A.; Berhe, R.; Gedefaw, M.Prevalence and associated factors of active trachoma among childeren aged 1-9 years in rural communities of Gonji Kolella district, West Gojjam zone, North West Ethiopia. 2015. BMC Research Notes. , volume: 8, issue: , pages: 641. Doi: https://dx.doi.org/10.1186/s13104-015-1529-6. | Ethiopia | Cross-sectional | TF | TI, TF and/or TI | no | no | no | no | Checklist for Prevalence Studies | Yes | Yes | Yes | Yes | Yes | Yes | Yes | Yes | Yes | | | | | | | | | | | [9/9] (100%) | NA | 100% |
| Noatina, B. N.; Kagmeni, G.; Mengouo, M. N.; Moungui, H. C.; Tarini, A.; Zhang, YaoBi; Bella, A. L. F.Prevalence of trachoma in the Far North region of Cameroon: results of a survey in 27 health districts. 2013. PLoS Neglected Tropical Diseases. , volume: 7, issue: 5, pages: e2240. Doi: http://dx.doi.org/10.1371/journal.pntd.0002240. | Cameroon | Cross-sectional | TF | TF and/or TI | no | no | no | no | Checklist for Prevalence Studies | Yes | Yes | No | Yes | Yes | Yes | Yes | Yes | Yes | | | | | | | | | | | [8/9] (89%) | NA | 89% |
| Noatina, B. N.; Kagmeni, G.; Souleymanou, Y.; Moungui, H. C.; Hien, A. T.; Akame, J.; Zhang, YaoBi; Bella, A. L. F.Prevalence of trachoma in the north region of Cameroon: results of a survey in 15 health districts. 2014. PLoS Neglected Tropical Diseases. , volume: 8, issue: 6, pages: e2932. Doi: http://dx.doi.org/10.1371/journal.pntd.0002932. | Cameroon | Cross-sectional | TF | TF and/or TI | no | no | no | no | Checklist for Prevalence Studies | Unclear | Yes | Yes | Yes | Yes | Yes | Yes | Yes | Yes | | | | | | | | | | | [8/9] (89%) | NA | 89% |
| Odonkor, M.; Naufal, F.; Munoz, B.; Mkocha, H.; Kasubi, M.; Wolle, M.; West, S.Serology, infection, and clinical trachoma as tools in prevalence surveys for re-emergence of trachoma in a formerly hyperendemic district. 2021. PLoS Neglected Tropical Diseases. , volume: 15, issue: 4, pages: . Doi: http://dx.doi.org/10.1371/journal.pntd.0009343. | Tanzania | Cross-sectional | TF | no | yes | yes | yes | no | Both | Yes | Yes | Yes | Yes | No, 78% coverage | Yes | Yes | Yes | Yes | Random | Yes | Yes | Yes | Yes | Yes | Yes | Yes | Yes | Depends on measure | [8/9] (89%) | [8/10] (80%) | 89% |
| Odonkor, M.; Naufal, F.; Mkocha, H.; Funga, N.; Munoz, B.; West, S. K.The Impact of Image Quality and Trachomatous Inflammation on Using Photography for Trachoma Prevalence Surveys. 2022. Translational Vision Science & Technology. , volume: 11, issue: 3, pages: 11. Doi: https://dx.doi.org/10.1167/tvst.11.3.11. | Tanzania | Cross-sectional | TF | no | yes | no | no | no | Both | Unclear | Unclear | Unclear | No | Unclear | Yes | Yes | Yes | Unclear | Unclear | Yes | Yes | Yes | Yes | Yes | Yes | Yes | Yes | Yes | [3/9] (33%) | [9/10] (90%) | 90% |
| Oldenburg, C. E.; Amza, A.; Kadri, B.; Nassirou, B.; Cotter, S. Y.; Stoller, N. E.; West, S. K.; Bailey, R. L.; Porco, T. C.; Gaynor, B. D.; Keenan, J. D.; Lietman, T. M.Comparison of mass azithromycin coverage targets of children in Niger: a cluster-randomized trachoma trial. 2018. American Journal of Tropical Medicine and Hygiene. , volume: 98, issue: 2, pages: 389-395. Doi: http://dx.doi.org/10.4269/ajtmh.17-0501. | Niger | Cross-sectional within longitudinal | TF | no | no | yes | no | no | Checklist for Prevalence Studies | Yes | Yes | Unclear | Yes | Unclear | Yes | Yes | Yes | Unclear | | | | | | | | | | | [6/9] (67%) | NA | 67% |
| Oswald, W. E.; Stewart, A. E. P.; Kramer, M. R.; Endeshaw, T.; Zerihun, M.; Melak, B.; Sata, E.; Gessese, D.; Teferi, T.; Tadesse, Z.; Guadie, B.; King, J. D.; Emerson, P. M.; Callahan, E. K.; Flanders, D.; Moe, C. L.; Clasen, T. F.Active trachoma and community use of sanitation, Ethiopia. 2017. Bulletin of the World Health Organization. , volume: 95, issue: 4, pages: 250-260. Doi: http://dx.doi.org/10.2471/blt.16.177758. | Ethiopia | Cross-sectional | TF | TI, TF and/or TI | no | no | no | no | Checklist for Prevalence Studies | Yes | Yes | Unclear | No | Unclear | Yes | Yes | Yes | Unclear | | | | | | | | | | | [5/9] (56%) | NA | 56% |
| Phiri, I.; Manangazira, P.; Macleod, C. K.; Mduluza, T.; Dhobbie, T.; Chaora, S. G.; Chigwena, C.; Katiyo, J.; Willis, R.; Bakhtiari, A.; Bare, P.; Courtright, P.; Macheka, B.; Midzi, N.; Solomon, A. W.The burden of and risk factors for trachoma in selected districts of Zimbabwe: results of 16 population-based prevalence surveys. 2018. Special Issue: 2017 Global Trachoma Mapping Project. , volume: 25, issue: Suppl. 1, pages: 181-191. Doi: http://dx.doi.org/10.1080/09286586.2017.1298823. | Zimbabwe | Cross-sectional | TF | TI | no | no | no | no | Checklist for Prevalence Studies | Yes | Yes | Yes | Yes | Yes | Yes | Yes | Yes | Yes | | | | | | | | | | | [9/9] (100%) | NA | 100% |
| Quicke, E.; Sillah, A.; Harding-Esch, E. M.; Last, A.; Joof, H.; Makalo, P.; Bailey, R. L.; Burr, S. E.Follicular trachoma and trichiasis prevalence in an urban community in The Gambia, West Africa: is there a need to include urban areas in national trachoma surveillance?. 2013. Tropical Medicine and International Health. , volume: 18, issue: 11, pages: 1344-1352. Doi: http://dx.doi.org/10.1111/tmi.12182. | Gambia | Cross-sectional | TF | TI | no | no | no | no | Checklist for Prevalence Studies | Yes | Yes | Unclear | Yes | Yes | Yes | Yes | Yes | Yes | | | | | | | | | | | [8/9] (89%) | NA | 89% |
| Ramadhani, A. M.; Derrick, T.; Macleod, D.; Massae, P.; Malisa, A.; Mbuya, K.; Mtuy, T.; Makupa, W.; Roberts, C. H.; Bailey, R. L.; Mabey, D. C. W.; Holland, M. J.; Burton, M. J.Ocular immune responses, Chlamydia trachomatis infection and clinical signs of trachoma before and after azithromycin mass drug administration in a treatment naïve trachoma-endemic Tanzanian community. 2019. PLoS Neglected Tropical Diseases. , volume: 13, issue: 7, pages: e0007559. Doi: http://dx.doi.org/10.1371/journal.pntd.0007559. | Tanzania | Cross-sectional within longitudinal | TF, F2/F3 | TI, P2/P3 | yes | yes | no | no | Checklist for Prevalence Studies | Yes | Unclear | Unclear | Yes | Unclear | Yes | Yes | Yes | Unclear | | | | | | | | | | | [5/9] (56%) | NA | 56% |
| Ramyil, A.; Wade, P.; Ogoshi, C.; Goyol, M.; Adenuga, O.; Dami, N.; Mpyet, C.Prevalence of trachoma in Jigawa State, Northwestern Nigeria. 2015. Special Issue: Trachoma... , volume: 22, issue: 3, pages: 184-189. Doi: . | Nigeria | Cross-sectional | TF | TI | no | no | no | no | Checklist for Prevalence Studies | Yes | Yes | Yes | Yes | Yes | Yes | Yes | Yes | Yes | | | | | | | | | | | [9/9] (100%) | NA | 100% |
| Reda, G.; Yemane, D.; Gebreyesus, A.Prevalence and associated factors of active trachoma among 1-9years old children in Deguatemben, Tigray, Ethiopia, 2018: community cross-sectional study. 2020. BMC Ophthalmology. , volume: 20(1), issue: , pages: 144. Doi: https://dx.doi.org/10.1186/s12886-020-01394-0. | Ethiopia | Cross-sectional | TF | TI, TF and/or TI, TF and TI | no | no | no | no | Checklist for Prevalence Studies | Yes | Yes | Yes | Yes | Yes | Yes | Yes | Yes | Yes | | | | | | | | | | | [9/9] (100%) | NA | 100% |
| Reilly, L. A.; Favacho, J.; Garcez, L. M.; Courtenay, O.Preliminary evidence that synanthropic flies contribute to the transmission of trachoma-causing Chlamydia trachomatis in Latin America. 2007. Cadernos de Saude Publica. , volume: 23, issue: 7, pages: 1682-1688. Doi: http://dx.doi.org/10.1590/S0102-311X2007000700020. | Brazil | Cross-sectional | TF | TI, TF and/or TI | no | no | no | no | Checklist for Prevalence Studies | Yes | Unclear | Unclear | No | Yes | Yes | Yes | Yes | Yes | | | | | | | | | | | [6/9] (67%) | NA | 67% |
| Roba, A. A.; Patel, D.; Zondervan, M.Risk of trachoma in a SAFE intervention area. 2013. International Ophthalmology. , volume: 33, issue: 1, pages: 53-59. Doi: 10.1007/s10792-012-9632-3. | Ethiopia | Cross-sectional | TF | TI, TF and/or TI | no | no | no | no | Checklist for Prevalence Studies | No, excluded communities receiving more frequent MDAs as part of other intervention studies | Yes | Yes | Yes | Yes | Yes | Yes | Yes | Yes | | | | | | | | | | | [8/9] (89%) | NA | 89% |
| D.S, RosenTrachoma among children in western Yemen. 1995. , volume: 3, issue: , pages: 45245. Doi: . | Yemen | Cross-sectional | TF | TI | no | no | no | no | Checklist for Prevalence Studies | Unclear | Yes | Unclear | No | Unclear | Unclear | Unclear | Unclear | Unclear | | | | | | | | | | | [1/9] (11%) | NA | 11% |
| Ruberanziza, E.; Mupfasoni, D.; Nizeyimana, V.; Karibushi, B.; Kabera, M.; Kaberuka, T.; Kabanda, G.; Sebeza, J.; Kramer, M. H.; Ruxin, J.; Fenwick, A.; Kalua, K.Prevalence and risk factors for trachoma in Rwanda. 2009. East African Journal of Public Health. , volume: 6, issue: 3, pages: 287-91. Doi: . | Rwanda | Cross-sectional | TF | TI | no | no | no | no | Checklist for Prevalence Studies | Yes | Yes | Yes | Yes | Yes | Yes | Yes | Yes | Yes | | | | | | | | | | | [9/9] (100%) | NA | 100% |
| Saboya-Diaz, M. I.; Angeles, C. A. C.; Socorro Avellaneda Yajahuanca, R. del; Melendez Ruiz, S. K.; Cabrera, R.; Honorio Morales, H. A.; Pachas, P. E.; Guardo, M.; Renneker, K. K.; Munoz, B. E.; West, S. K.Associated factors of the co-occurrence of trachoma and soil-transmitted helminthiases in children 1 to 9 years old in rural communities of the Amazon basin in Loreto Department, Peru: results from a population-based survey. 2022. PLoS Neglected Tropical Diseases. , volume: 16, issue: 7, pages: . Doi: http://dx.doi.org/10.1371/journal.pntd.0010532. | Peru | Cross-sectional | TF | no | no | yes | no | no | Checklist for Prevalence Studies | Yes | Yes | Yes | Yes | Unclear | Yes | Yes | Yes | Unclear | | | | | | | | | | | [7/9] (78%) | NA | 78% |

S6. Table of included studies

| | | | | | | | | | | Risk of Bias checklist items** | | | | | | | | | | | | | | | | | | | | | |
|---|---|---|---|---|---|---|---|---|---|---|---|---|---|---|---|---|---|---|---|---|---|---|---|---|---|---|---|---|---|---|---|
| reference | Country or countries of data collection | Type of study | TF or FPC | other clinical indicators? | photo-graded TF or FPC? | infection? | serology? | SCR? | Critical Appraisal Tool used** | Prevalence study: Was the sample frame appropriate to address the target pop? | Prevalence study: Were study participants sampled in an appropriate way? | Prevalence study: Was the sample size adequate? | Prevalence study: Were the study subjects and setting described in detail? | Prevalence study: Was the data analysis conducted with sufficient coverage of the identified sample? | Prevalence study: Were valid methods used for the identification of the condition[s]? | Prevalence study: Was the condition measured in a standard, reliable way for all participants? | Prevalence study: Was there appropriate statistical analysis? | Prevalence study: Was the response rate adequate, and if not, was the low response rate managed appropriately? | DTA study: Was a consecutive or random sample of patients enrolled? | DTA study: Was a case-control design avoided? | DTA study: Did the study avoid inappropriate exclusions? | DTA: Were the index test results interpreted without knowledge of the results of the reference standard? | DTA: If a threshold was used, was it pre-specified? | DTA: Is the reference standard likely to correctly classify the target condition? | DTA: Were the reference standard results interpreted without knowledge of the results of the index test? | DTA: Was there an appropriate interval between index test and reference standard? | DTA: Did all patients receive the same reference standard? | DTA: Were all patients included in the analysis? | Prevalence study score [meets criteria/total] (%) | DTA study score [meets criteria/total] (%) | Max score (if both checklists used, higher of 2 scores) |
| Sanders, A. M.; Stewart, A. E. P.; Makoy, S.; Chebet, J. J.; Magok, P.; Kuol, A.; Blauvelt, C.; Lako, R.; Rumunu, J.; Callahan, E. K.; Nash, S. D.Burden of trachoma in five counties of Eastern Equatoria state, South Sudan: results from population-based surveys. 2017. PLoS Neglected Tropical Diseases. , volume: 11, issue: 6, pages: e0005658. Doi: . | South Sudan | Cross-sectional | TF | TI, TF and/or TI | no | no | no | no | Checklist for Prevalence Studies | Yes | Yes | Yes | Yes | Yes | Yes | Yes | Yes | Yes | | | | | | | | | | | [9/9] (100%) | NA | 100% |
| Sanders, A. M.; Abdalla, Z.; Elshafie, B. E.; Elsanosi, M.; Nute, A. W.; Aziz, N.; Callahan, E. K.; Nash, S. D.Progress toward elimination of trachoma as a public health problem in seven localities in the republic of Sudan: results from population-based surveys. 2019. American Journal of Tropical Medicine and Hygiene. , volume: 101, issue: 6, pages: 1296-1302. Doi: . | Sudan | Cross-sectional | TF | TI | no | no | no | no | Checklist for Prevalence Studies | Yes | Yes | Yes | Yes | No | Yes | Yes | Yes | No | | | | | | | | | | | [7/9] (78%) | NA | 78% |
| Sanders, A. M.; Abdalla, Z.; Elshafie, B. E.; Nute, A. W.; Long, E. F.; Aziz, N.; Weiss, P.; Callahan, E. K.; Nash, S. D.Prevalence of trachoma within refugee camps serving South Sudanese refugees in White Nile State, Sudan: results from population-based surveys. 2019. PLoS Neglected Tropical Diseases. , volume: 13, issue: 6, pages: e0007491. Doi: http://dx.doi.org/10.1371/journal.pntd.0007491. | Sudan | Cross-sectional | TF | TI | no | no | no | no | Checklist for Prevalence Studies | Yes | Yes | Yes | Yes | Yes | Yes | Yes | Yes | Yes | | | | | | | | | | | [9/9] (100%) | NA | 100% |
| Schellini, S. A.; Lavezzo, M. M.; Ferraz, L. B.; Olbrich Neto, J.; Medina, N. H.; Padovani, C. R.Prevalence and spatial distribution of trachoma among schoolchildren in Botucatu, Sao Paulo-Brazil. 2010. Prevalencia e localizacao espacial dos casos de tracoma detectados em escolares de Botucatu, Sao Paulo-Brasil.. , volume: 73, issue: 4, pages: 358-362. Doi: http://dx.doi.org/10.1590/S0004-27492010000400012. | Brazil | Cross-sectional | TF | TI | no | no | no | no | Checklist for Prevalence Studies | No, school-based | Yes | Yes | Yes | Unclear | Yes | Yes | Yes | Unclear | | | | | | | | | | | [6/9] (67%) | NA | 67% |
| Schemann, J. F.; Sacko, D.; Banou, A.; Bamani, S.; Bore, B.; Coulibaly, S.; Ag El Mouchtahide, M.Mapping trachoma in Mali: Results of a national survey. [French]. 1998. Bulletin of the World Health Organization. , volume: 76(6), issue: , pages: 599-606. Doi: . | Mali | Cross-sectional | TF | TI | no | no | no | no | Checklist for Prevalence Studies | Yes | Yes | Unclear | Yes | Unclear | Yes | Yes | Yes | Unclear | | | | | | | | | | | [6/9] (67%) | NA | 67% |
| See, C. W.; Alemayehu, W.; Melese, M.; Zhou, Z.; Porco, T. C.; Shiboski, S.; Gaynor, B. D.; Eng, J.; Keenan, J. D.; Lietman, T. M.How reliable are tests for trachoma?--a latent class approach. 2011. Investigative Ophthalmology & Visual Science. , volume: 52, issue: 9, pages: 6133-7. Doi: https://dx.doi.org/10.1167/iovs.11-7419. | Ethiopia | Cross-sectional | TF | TI | no | yes | no | no | Both | Yes | Yes | Unclear | No | Unclear | Yes | Yes | Yes | Unclear | Random | Yes | Yes | Yes | Yes | Yes | Yes | Yes | Yes | Yes | [5/9] (56%) | [9/10] (90%) | 90% |
| Senyonjo, L. G.; Debrah, O.; Martin, D. L.; Asante-Poku, A.; Migchelsen, S. J.; Gwyn, S.; deSouza, D. K.; Solomon, A. W.; Agyemang, D.; Biritwum-Kwadwo, N.; Marfo, B.; Bakajika, D.; Mensah, E. O.; Aboe, A.; Koroma, J.; Addy, J.; Bailey, R.Serological and PCR-based markers of ocular Chlamydia trachomatis transmission in northern Ghana after elimination of trachoma as a public health problem. 2018. PLoS Neglected Tropical Diseases. , volume: 12, issue: 12, pages: e0007027. Doi: . | Ghana | Cross-sectional | TF | no | no | yes | yes | yes | Both | Yes | Yes | Unclear | Yes | Yes | Yes | Yes | Yes | Yes | Random | Yes | Yes | Yes | Yes | Yes | Yes | Yes | Yes | Yes | [8/9] (89%) | [9/10] (90%) | 90% |
| Mohammed, Shaffi; Abebe, BejigaCommon eye diseases in children of rural community in Goro district, Central Ethiopia. 2005. Ethiopian Journal of Health Development. , volume: 19, issue: 2, pages: 148-152. Doi: . | Ethiopia | Cross-sectional | TF | TI, TF and/or TI | no | no | no | no | Checklist for Prevalence Studies | Yes | Yes | Yes | Yes | Unclear | Yes | Yes | Yes | Unclear | | | | | | | | | | | [7/9] (78%) | NA | 78% |
| Sheehan, J. P.; Gebresillasie, S.; Shiferaw, A.; Aragie, S.; Tadesse, Z.; Tadesse, D.; Somkijrungroj, T.; Stoller, N. E.; Callahan, E. K.; Emerson, P. M.; Lietman, T. M.; Keenan, J. D.School-Based versus Community-Based Sampling for Trachoma Surveillance. 2018. American Journal of Tropical Medicine and Hygiene. , volume: 99(1), issue: , pages: 150-154. Doi: https://dx.doi.org/10.4269/ajtmh.17-0904. | Ethiopia | Cross-sectional | TF | no | no | yes | no | no | Checklist for Prevalence Studies | Yes | Yes | Unclear | No, No number sampled | Unclear | Yes | Yes | Yes | Unclear | | | | | | | | | | | [5/9] (56%) | NA | 56% |
| Shekhawat, N.; Mkocha, H.; Munoz, B.; Gaydos, C.; Dize, L.; Quinn, T. C.; West, S. K.Cohort and age effects of mass drug administration on prevalence of trachoma: a longitudinal study in rural Tanzania. 2014. Investigative Ophthalmology & Visual Science. , volume: 55, issue: 4, pages: 2307-2314. Doi: http://dx.doi.org/10.1167/iovs.13-12701. | Tanzania | Cross-sectional within longitudinal | TF | no | no | yes | no | no | Checklist for Prevalence Studies | Yes | Yes | Unclear | No | Unclear | Yes | Yes | Yes | Unclear | | | | | | | | | | | [5/9] (56%) | NA | 56% |
| Shimelash, A.; Alemayehu, M.; Dagne, H.; Mihiretie, G.; Lamore, Y.; Tegegne, E.; Kumlachew, L.Prevalence of active trachoma and associated factors among school age children in Debre Tabor Town, Northwest Ethiopia, 2019: a community based cross-sectional study. 2022. Italian Journal of Pediatrics. , volume: 48, issue: 1, pages: 61. Doi: https://dx.doi.org/10.1186/s13052-022-01258-x. | Ethiopia | Cross-sectional | TF | TI, TF and/or TI | no | no | no | no | Checklist for Prevalence Studies | Yes | Yes | Yes | Yes | Yes | Yes | Yes | Yes | Yes | | | | | | | | | | | [9/9] (100%) | NA | 100% |
| Silva, Evanildo JosÃ© da; Oliveira, Layze Alves Vieira; Nasser, Luciano SÃ³lia; Leite, Luciana Fernandes Amaro; Caldeira, AntÃ´nio Prates; Oliveira, Leida CalegÃ¡rio dePrevalÃªncia de Tracoma em crianÃ§as em idade escolar no municÃpio de Turmalina, MG. 2016. Rev. bras. oftalmol. , volume: 75, issue: 3, pages: 181-184. Doi: 10.5935/0034-7280.20160038. | Brazil | Cross-sectional | TF | TI | no | no | no | no | Checklist for Prevalence Studies | No, school based | Unclear | Yes | Yes | Unclear | Unclear, TF definition Not given or cited | Yes | Yes | Unclear | | | | | | | | | | | [4/9] (44%) | NA | 44% |
| Smith, A. G.; Broman, A. T.; Alemayehu, W.; Munoz, B. E.; West, S. K.; Gower, E. W.Relationship between trachoma and chronic and acute malnutrition in children in rural Ethiopia. 2007. Journal of Tropical Pediatrics. , volume: 53(5), issue: , pages: 308-312. Doi: https://dx.doi.org/10.1093/tropej/fmm039. | Ethiopia | Cross-sectional | TF | TI, TF and/or TI | no | yes | no | no | Both | No, only includes children w/ a relative who had TT surgery | Not for a prevalence study | Unclear | Yes | Unclear | Yes | Yes | Yes | Unclear | Unclear | Yes | Yes | Unclear | Yes | Yes | Yes | Yes | Yes | Yes | [4/9] (44%) | [8/10] (80%) | 80% |
| Snyder, B. M.; Sie, A.; Tapsoba, C.; Dah, C.; Ouermi, L.; Zakane, S. A.; Keenan, J. D.; Oldenburg, C. E.Smartphone photography as a possible method of post-validation trachoma surveillance in resource-limited settings. 2019. International Health. , volume: 11(6), issue: , pages: 613-615. Doi: https://dx.doi.org/10.1093/inthealth/ihz035. | Burkina Faso | Cross-sectional | TF | TI | yes | no | no | no | Checklist for DTA studies | | | | | | | | | | Random | Yes | Yes | Unclear | Yes | Yes | Yes | Yes | Yes | Yes | NA | [8/10] (80%) | 80% |
| Solomon, A.Rational use of azithromycin in the control of trachoma : using quantitative PCR to assess distribution of infection and impact of treatment. 2003. . , volume: , issue: , pages: . Doi: . | Tanzania | Cross-sectional within longitudinal | TF | TI, TF and/or TI | no | yes | no | no | Both | Yes | Yes | Yes | Yes | Yes | Yes | Yes | Yes | Yes | NA, whole pop | Yes | Yes | Yes | Yes | Yes | Yes | Yes | Yes | Yes | [9/9] (100%) | [9/10] (90%) | 100% |
| Solomon, A. W.; Harding-Esch, E.; Alexander, N. D. E.; Aguirre, A.; Holland, M. J.; Bailey, R. L.; Foster, A.; Mabey, D. C. W.; Massae, P. A.; Courtright, P.; Shao, J. F.Two doses of azithromycin to eliminate trachoma in a Tanzanian community. 2008. New England Journal of Medicine. , volume: 358, issue: 17, pages: 1870-1871. Doi: http://dx.doi.org/10.1056/NEJMc0706263. | Tanzania | Cross-sectional within longitudinal | TF | no | no | yes | no | no | Checklist for Prevalence Studies | Yes | Yes | Yes | No | Yes | Yes | Yes | Yes | Yes | | | | | | | | | | | [8/9] (89%) | NA | 89% |
| Stare, D.; Harding-Esch, E.; Munoz, B.; Bailey, R.; Mabey, D.; Holland, M.; Gaydos, C.; West, S.Design and baseline data of a randomized trial to evaluate coverage and frequency of mass treatment with azithromycin: the Partnership for Rapid Elimination of Trachoma (PRET) in Tanzania and The Gambia. 2011. Ophthalmic Epidemiology. , volume: 18, issue: 1, pages: 20-29. Doi: http://dx.doi.org/10.3109/09286586.2010.545500. | Gambia, Tanzania | Cross-sectional within longitudinal | TF | no | no | yes | no | no | Checklist for Prevalence Studies | Yes | Yes | Unclear | No | Unclear | Yes | Yes | Yes | Unclear | | | | | | | | | | | [5/9] (56%) | NA | 56% |
| Stewart, A. E. P.; Mulat, Zerihun; Demelash, Gessese; Berhanu, Melak; Eshetu, Sata; Nute, A. W.; Tigist, Astale; Tekola, Endeshaw; Tesfaye, Teferi; Zerihun, Tadesse; Callahan, E. K.; Melsew, Chanyalew; Birhan, Gaudie; Emerson, P. M.; King, J. D.; Nash, S. D.Progress to eliminate trachoma as a public health problem in Amhara National Regional State, Ethiopia: results of 152 population-based surveys. 2019. American Journal of Tropical Medicine and Hygiene. , volume: 101, issue: 6, pages: 1286-1295. Doi: . | Ethiopia | Cross-sectional | TF | TI, TF and/or TI | no | no | no | no | Checklist for Prevalence Studies | Yes | Yes | Unclear | Yes | Unclear | Yes | Yes | Yes | No | | | | | | | | | | | [6/9] (67%) | NA | 67% |
| Tadesse, B.; Worku, A.; Kumie, A.; Yimer, S. A.Effect of water, sanitation and hygiene interventions on active trachoma in North and South Wollo zones of Amhara Region, Ethiopia: A Quasi-experimental study. 2017. PLoS Neglected Tropical Diseases. , volume: 11(11) (no pagination), issue: , pages: . Doi: https://dx.doi.org/10.1371/journal.pntd.0006080. | Ethiopia | Cross-sectional within longitudinal | TF | TI, TF and/or TI | no | no | no | no | Checklist for Prevalence Studies | No, purposively sampled based on water status | Yes | Yes | Yes | Unclear | Yes | Yes | Yes | Unclear | | | | | | | | | | | [6/9] (67%) | NA | 67% |
| Beselam, Tadesse; Alemayehu, Worku; Abera, Kumie; Yimer, S. A.The burden of and risk factors for active trachoma in the North and South Wollo Zones of Amhara Region, Ethiopia: a crosssectional study. 2017. Infectious Diseases of Poverty. , volume: 6, issue: 143, pages: . Doi: http://dx.doi.org/10.1186/s40249-017-0358-3. | Ethiopia | Cross-sectional | TF | TI | no | no | no | no | Checklist for Prevalence Studies | Yes | Yes | Yes | Yes | Unclear | Yes | Yes | Yes | Unclear | | | | | | | | | | | [7/9] (78%) | NA | 78% |
| Praveen, Vashist; Noopur, Gupta; Rathore, A. S.; Anita, Shah; Suman, SinghRapid assessment of trachoma in underserved population of Car-Nicobar Island, India. 2013. PLoS ONE [Electronic Resource]. , volume: 8, issue: 6, pages: e65918. Doi: http://dx.doi.org/10.1371/journal.pone.0065918. | India | Cross-sectional | TF | TI, TF and/or TI | no | no | no | no | Checklist for Prevalence Studies | No, purposive (TRA) | Yes | Unclear | Yes | Unclear | Yes | Yes | Yes | Unclear | | | | | | | | | | | [5/9] (56%) | NA | 56% |
| Wang, M.; Lu, X.; Hu, A.; Zhang, M.; Li, X.; Deng, S.; Wang, N.Etiological characteristics of chlamydia trachoma conjunctivitis of Primary Boarding School students in the Qinghai Tibetan area. 2016. Science China. , volume: Life sciences. 59(6), issue: , pages: 555-560. Doi: https://dx.doi.org/10.1007/s11427-016-5060-3. | China | Case-control | TF | no | no | yes | no | no | Checklist for Prevalence Studies | No, school based | Unclear | Unclear | Yes | Unclear | No, swabs included both upper tarsal and conj sac. In addition, cases were students with any number of follicles. | Yes | Yes | Unclear | | | | | | | | | | | [3/9] (33%) | NA | 33% |
| West, S.; Munoz, B.; Bobo, L.; Quinn, T. C.; Mkocha, H.; Lynch, M.; Mmbaga, B. B. O.; Viscidi, R.Nonocular chlamydia infection and risk of ocular reinfection after mass treatment in a trachoma hyperendemic area. 1993. Investigative Ophthalmology and Visual Science. , volume: 34(11), issue: , pages: 3194-3198. Doi: . | Tanzania | Cross-sectional | TF | TI | no | yes | no | no | Checklist for Prevalence Studies | Yes | Unclear | Unclear | No, No year | Unclear | Yes | Yes | Yes | Unclear | | | | | | | | | | | [4/9] (44%) | NA | 44% |
| West, S. K.; Munoz, B.; Mkocha, H.; Hsieh, Y. H.; Lynch, M. C.Progression of active trachoma to scarring in a cohort of Tanzanian children. 2001. Ophthalmic Epidemiology. , volume: 8, issue: 44960, pages: 137-44. Doi: . | Tanzania | Case-control | TF | TI | no | yes | no | no | Checklist for Prevalence Studies | No, case control | Unclear | Unclear | Yes | Unclear | Yes | Yes | Yes | Unclear | | | | | | | | | | | [4/9] (44%) | NA | 44% |
| West, S. K.; Munoz, B.; Mkocha, H.; Gaydos, C.; Quinn, T.Trachoma and ocular Chlamydia trachomatis were not eliminated three years after two rounds of mass treatment in a trachoma hyperendemic village. 2007. Investigative Ophthalmology & Visual Science. , volume: 48, issue: 4, pages: 1492-1497. Doi: . | Tanzania | Cross-sectional within longitudinal | TF | TI | no | yes | no | no | Checklist for Prevalence Studies | Yes | Yes | Yes | Yes | Yes | Yes | Yes | Yes | Yes | | | | | | | | | | | [9/9] (100%) | NA | 100% |

S6. Table of included studies

| reference | Country or countries of data collection | Type of study | TF or FPC | other clinical indicators? | photo-graded TF or FPC? | infection? | serology? | SCR? | Critical Appraisal Tool used** | Risk of Bias checklist items** Prevalence study: Was the sample frame appropriate to address the target pop? | Prevalence study: Were study participants sampled in an appropriate way? | Prevalence study: Was the sample size adequate? | Prevalence study: Were the study subjects and setting described in detail? | Prevalence study: Was the data analysis conducted with sufficient coverage of the identified sample? | Prevalence study: Were valid methods used for the identification of the condition(s)? | Prevalence study: Was the condition measured in a standard, reliable way for all participants? | Prevalence study: Was there appropriate statistical analysis? | Prevalence study: Was the response rate adequate, and if not, was the low response rate managed appropriately? | DTA study: Was a consecutive or random sample of patients enrolled? | DTA study: Was a case-control design avoided? | DTA study: Did the study avoid inappropriate exclusions? | DTA: Were the index test results interpreted without knowledge of the results of the reference standard? | DTA: If a threshold was used, was it pre-specified? | DTA: Is the reference standard likely to correctly classify the target condition? | DTA: Were the reference standard results interpreted without knowledge of the results of the index test? | DTA: Was there an appropriate interval between index test and reference standard? | DTA: Did all patients receive the same reference standard? | DTA: Were all patients included in the analysis? | Prevalence study score [meets criteria/total] (%) | DTA study score [meets criteria/total] (%) | Max score (if both checklists used, higher of 2 scores) |
|---|---|---|---|---|---|---|---|---|---|---|---|---|---|---|---|---|---|---|---|---|---|---|---|---|---|---|---|---|---|---|---|
| West, S. K.; Stare, D.; Mkocha, H.; Munoz, B.; Gaydos, C.; Quinn, T. C.Do infants increase the risk of re-emergent infection in households after mass drug administration for trachoma?. 2011. Investigative Ophthalmology & Visual Science. , volume: 52, issue: 8, pages: 6040-2. Doi: https://dx.doi.org/10.1167/iovs.11-7372. | Tanzania | Cross-sectional within longitudinal | TF | TI | no | yes | no | no | Checklist for Prevalence Studies | Yes | Yes | Yes | No | Yes | Yes | Yes | Yes | Yes | | | | | | | | | | | [8/9] (89%) | NA | 89% |
| West, S. K.; Bailey, R.; Munoz, B.; Edwards, T.; Mkocha, H.; Gaydos, C.; Lietman, T.; Porco, T.; Mabey, D.; Quinn, T. C.A randomized trial of two coverage targets for mass treatment with azithromycin for trachoma. 2013. PLoS Neglected Tropical Diseases. , volume: 7, issue: 8, pages: e2415. Doi: http://dx.doi.org/10.1371/journal.pntd.0002415. | Tanzania | Cross-sectional within longitudinal | TF | no | no | yes | no | no | Checklist for Prevalence Studies | Yes | Unclear | Yes | No | Unclear | Yes | Yes | Yes | Unclear | | | | | | | | | | | [5/9] (56%) | NA | 56% |
| West, S. K.; Munoz, B. E.; Mkocha, H.; Gaydos, C.; Quinn, T.Risk of infection with Chlamydia trachomatis from migrants to communities undergoing mass drug administration for trachoma control. 2015. Special Issue: Trachoma. , volume: 22, issue: 3, pages: 170-175. Doi: | Tanzania | Cross-sectional within longitudinal | TF | no | no | yes | no | no | Checklist for Prevalence Studies | Yes | Yes | Unclear | No | Unclear | Yes | Yes | Yes | Unclear | | | | | | | | | | | [5/9] (56%) | NA | 56% |
| West, S. K.; Munoz, B.; Weaver, J.; Mrango, Z.; Dize, L.; Gaydos, C.; Quinn, T. C.; Martin, D. L.Can we use antibodies to Chlamydia trachomatis as a surveillance tool for national trachoma control programs? Results from a district survey. 2016. PLoS Neglected Tropical Diseases. , volume: 10, issue: 1, pages: e0004352. Doi: http://dx.doi.org/10.1371/journal.pntd.0004352. | Tanzania | Cross-sectional | TF | no | no | yes | yes | no | Checklist for Prevalence Studies | Yes | Yes | Yes | No | Yes | Yes | Yes | Yes | Yes | | | | | | | | | | | [8/9] (89%) | NA | 89% |
| West, S. K.; Munoz, B.; Mkocha, H.; Dize, L.; Gaydos, C. A.; Swenor, B.; Ervin, A. M.; Quinn, T. C.Treating village newcomers and travelers for trachoma: results from ASANTE cluster randomized trial. 2017. PLoS ONE [Electronic Resource]. , volume: 12, issue: 6, pages: e0178595. Doi: http://dx.doi.org/10.1371/journal.pone.0178595. | Tanzania | Cross-sectional within longitudinal | TF | no | no | yes | no | no | Checklist for Prevalence Studies | Yes | Yes | Unclear | No | Unclear | Yes | Yes | Yes | Unclear | | | | | | | | | | | [5/9] (56%) | NA | 56% |
| West, S. K.; Zambrano, A. I.; Sharma, S.; Mishra, S. K.; Munoz, B. E.; Dize, L.; Crowley, K.; Gaydos, C. A.; Rotondo, L. A.Surveillance surveys for reemergent trachoma in formerly endemic districts in Nepal from 2 to 10 years after mass drug administration cessation. 2017. JAMA Ophthalmology. , volume: 135, issue: 11, pages: 1141-1146. Doi: http://dx.doi.org/10.1001/jamaophthalmol.2017.3062. | Nepal | Cross-sectional | TF | no | no | yes | yes | no | Checklist for Prevalence Studies | Yes | Yes | Yes | Yes | Unclear | Yes | Yes | Yes | Unclear | | | | | | | | | | | [7/9] (78%) | NA | 78% |
| West, S. K.; Munoz, B.; Kaur, H.; Dize, L.; Mkocha, H.; Gaydos, C. A.; Quinn, T. C.Longitudinal change in the serology of antibodies to Chlamydia trachomatis pgp3 in children residing in a trachoma area. 2018. Scientific Reports. , volume: 8, issue: 1, pages: 3520. Doi: http://dx.doi.org/10.1038/s41598-018-21127-0. | Tanzania | Cross-sectional | TF | no | no | yes | yes | yes | Checklist for Prevalence Studies | Yes | Yes | Yes | Yes | Unclear | Yes | Yes | Yes | Unclear | | | | | | | | | | | [7/9] (78%) | NA | 78% |
| West, S. K.; Nanji, A. A.; Mkocha, H.; Munoz, B.; Gaydos, C.; Quinn, T. C.Evidence for contamination with C. trachomatis in the household environment of children with active trachoma: a cross-sectional study in Kongwa, Tanzania. 2019. PLoS Neglected Tropical Diseases. , volume: 13, issue: 12, pages: . Doi: http://dx.doi.org/10.1371/journal.pntd.0007834. | Tanzania | Cross-sectional | TF | no | no | yes | no | no | Checklist for Prevalence Studies | Yes | Yes | Yes | No. No year | Unclear | Yes | Yes | Yes | Unclear | | | | | | | | | | | [6/9] (67%) | NA | 67% |
| West, S. K.; Munoz, B.; Mkocha, H.; Gaydos, C. A.; Quinn, T. C.The effect of mass drug administration for trachoma on antibodies to Chlamydia trachomatis pgp3 in children. 2020. Scientific Reports. , volume: 10, issue: 9, pages: . Doi: http://dx.doi.org/10.1038/s41598-020-71833-x. | Tanzania | Cross-sectional | TF | no | no | yes | yes | yes | Checklist for Prevalence Studies | Yes | Yes | Yes | Yes | Unclear | Yes | Yes | Yes | Unclear | | | | | | | | | | | [7/9] (78%) | NA | 78% |
| Wilson, N.; Goodhew, B.; Mkocha, H.; Joseph, K.; Bandea, C.; Black, C.; Igietseme, J.; Munoz, B.; West, S. K.; Lammie, P.; Kasubi, M.; Martin, D. L.Evaluation of a single dose of azithromycin for trachoma in low-prevalence communities. 2019. Ophthalmic Epidemiology. , volume: 26, issue: 1, pages: 44932. Doi: http://dx.doi.org/10.1080/09286586.2017.1293693. | Tanzania | Cross-sectional within longitudinal | TF | no | no | yes | yes | no | Checklist for Prevalence Studies | Yes | Yes | Yes | Yes | Unclear | Yes | Yes | Yes | Unclear | | | | | | | | | | | [7/9] (78%) | NA | 78% |
| Woldekidan, E.; Daka, D.; Legesse, D.; Laelago, T.; Betebo, B.Prevalence of active trachoma and associated factors among children aged 1 to 9 years in rural communities of Lemo district, southern Ethiopia: Community based cross sectional study. 2019. BMC Infectious Diseases. , volume: 19(1) (no pagination), issue: , pages: . Doi: https://dx.doi.org/10.1186/s12879-019-4495-0. | Ethiopia | Cross-sectional | TF | TI, TF and/or TI | no | no | no | no | Checklist for Prevalence Studies | Yes | Yes | Yes | Yes | Yes | Yes | Yes | Yes | Yes | | | | | | | | | | | [9/9] (100%) | NA | 100% |
| Yoseph, Worku; Samson, BayuScreening for ocular abnormalities and subnormal vision in school children of Butajira town, southern Ethiopia. 2002. Ethiopian Journal of Health Development. , volume: 16, issue: 2, pages: 165-171. Doi: . | Ethiopia | Cross-sectional | TF | TI | no | no | no | no | Checklist for Prevalence Studies | No, school based | Yes | Unclear | Yes | Unclear | Yes | Yes | Yes | Yes | | | | | | | | | | | [6/9] (67%) | NA | 67% |
| Xue, WenWen; Lu, LiNa; Zhu, JianFeng; He, XiangGui; He, JiangNan; Zhao, Rong; Zou, HaiDongA cross-sectional population-based survey of trachoma among migrant school aged children in Shanghai, China. 2016. BioMed Research International. , volume: 2016, issue: , pages: Article-8692685. Doi: . | China | Cross-sectional | TF | TI | no | no | no | no | Checklist for Prevalence Studies | Yes | Unclear | Unclear | Yes | Yes | Yes | Yes | Yes | Yes | | | | | | | | | | | [7/9] (78%) | NA | 78% |
| Yang, J. L.; Schachter, J.; Moncada, J.; Habte, D.; Zerihun, M.; House, J. I.; Zhou, Z.; Hong, K. C.; Maxey, K.; Gaynor, B. D.; Lietman, T. M.Comparison of an rRNA-based and DNA-based nucleic acid amplification test for the detection of Chlamydia trachomatis in trachoma. 2007. British Journal of Ophthalmology. , volume: 91(3), issue: , pages: 293-295. Doi: https://dx.doi.org/10.1136/bjo.2006.099150. | Ethiopia | Cross-sectional | TF | TI, TF and/or TI | no | yes | no | no | Both | Yes | Unclear | Unclear | No | Unclear | Yes | Yes | Yes | Unclear | Consecutive | Yes | Yes | Unclear | Yes | Yes | Yes | Yes | Yes | Yes | [4/9] (44%) | [8/10] (80%) | 80% |
| Yang, J. L.; Hong, K. C.; Schachter, J.; Moncada, J.; Lekew, T.; House, J. I.; Zhou, Z.; Neuwelt, M. D.; Rutar, T.; Halfpenny, C.; Shah, N.; Whitcher, J. P.; Lietman, T. M.Detection of Chlamydia trachomatis ocular infection in trachoma-endemic communities by rRNA amplification. 2009. Investigative Ophthalmology and Visual Science. , volume: 50(1), issue: , pages: 90-94. Doi: https://dx.doi.org/10.1167/iovs.08-2247. | Ethiopia | Cross-sectional | TF | TI, TF and/or TI | no | yes | no | no | Both | Yes | Yes | Unclear | Yes | Unclear | Yes | Yes | Yes | Unclear | Random | Yes | Yes | Unclear | Yes | Yes | Yes | Yes | Yes | Yes | [6/9] (67%) | [8/10] (80%) | 80% |
| Yaya, G.; Kemata, B.; Baanam, M. Y.; Bobossi-Serengbe, G.Blinding trachoma: results of a prevalence survey in 8 health districts in CAR. 2015. Le trachome evolutif: resultats d'une enquete de prevalence dans huit prefectures sanitaires en RCA.. , volume: 108, issue: 4, pages: 299-304. Doi: http://dx.doi.org/10.1007/s13149-015-0446-1. | Central African Republic | Cross-sectional | TF | TI | no | no | no | no | Checklist for Prevalence Studies | Yes | Yes | Yes | Yes | Yes | Yes | Yes | Yes | Yes | | | | | | | | | | | [9/9] (100%) | NA | 100% |
| Yayemain, D.; King, J. D.; Debrah, O.; Emerson, P. M.; Aboe, A.; Ahorsu, F.; Wanye, S.; Ansah, M. O.; Gyapong, J. O.; Hagan, M.Achieving trachoma control in Ghana after implementing the SAFE strategy. 2009. Transactions of the Royal Society of Tropical Medicine and Hygiene. , volume: 103, issue: 10, pages: 993-1000. Doi: http://dx.doi.org/10.1016/j.trstmh.2009.02.007. | Ghana | Cross-sectional | TF | TI, TF and/or TI | no | no | no | no | Checklist for Prevalence Studies | Unclear | Unclear | Unclear | No | Unclear | Yes | Unclear | Unclear | Unclear | | | | | | | | | | | [1/9] (11%) | NA | 11% |
| Yilkal, Alemu; Abebe, BejigaThe impact of water supply on trachoma prevalence. 2004. Ethiopian Medical Journal. , volume: 42, issue: 3, pages: 179-184. Doi: . | Ethiopia | Cross-sectional | TF | TI | no | no | no | no | Checklist for Prevalence Studies | Yes | Yes | Yes | Yes | Unclear | Yes | Yes | Yes | Unclear | | | | | | | | | | | [7/9] (78%) | NA | 78% |
| Yohannan, J.; Munoz, B.; Mkocha, H.; Gaydos, C. A.; Bailey, R.; Lietman, T. A.; Quinn, T.; West, S. K.Can we stop mass drug administration prior to 3 annual rounds in communities with low prevalence of trachoma?: PRET Ziada trial results. 2013. JAMA Ophthalmology. , volume: 131, issue: 4, pages: 431-6. Doi: https://dx.doi.org/10.1001/jamaophthalmol.2013.2356. | Tanzania | Cross-sectional within longitudinal | TF | no | no | yes | no | no | Checklist for Prevalence Studies | Yes | Yes | Yes | No | Unclear | Yes | Yes | Yes | Unclear | | | | | | | | | | | [6/9] (67%) | NA | 67% |
| Zambrano, A. I.; Sharma, S.; Crowley, K.; Dize, L.; Munoz, B. E.; Mishra, S. K.; Rotondo, L. A.; Gaydos, C. A.; West, S. K.The World Health Organization Recommendations for Trachoma Surveillance, Experience in Nepal and Added Benefit of Testing for Antibodies to Chlamydia trachomatis pgp3 Protein: NESTS Study. 2016. PLoS Neglected Tropical Diseases. , volume: 10(9) (no pagination), issue: , pages: . Doi: https://dx.doi.org/10.1371/journal.pntd.0005003. | Nepal | Cross-sectional | TF | no | no | yes | yes | no | Checklist for Prevalence Studies | Yes | Yes | Unclear | No | Unclear | Yes | Yes | Yes | Unclear | | | | | | | | | | | [5/9] (56%) | NA | 56% |
| Zambrano, A. I.; Munoz, B. E.; Mkocha, H.; Dize, L.; Gaydos, C. A.; Quinn, T.; West, S. K.Measuring trachomatous inflammation-intense (TI) when prevalence is low provides data on infection with Chlamydia trachomatis. 2017. Investigative Ophthalmology & Visual Science. , volume: 58, issue: 2, pages: 997-1000. Doi: . | Tanzania | Cross-sectional within longitudinal | TF | TI, TF and/or TI | no | yes | no | no | Both | Yes | Yes | Yes | Yes | Unclear | Yes | Yes | Yes | Yes | Random | Yes | Yes | Yes | Yes | Yes | Yes | Yes | Yes | Yes | [8/9] (89%) | [9/10] (90%) | 90% |
| Zerihun, N.Trachoma in Jimma zone, South Western Ethiopia. 1997. Tropical Medicine and International Health. , volume: 2, issue: 12, pages: 1115-1121. Doi: . | Ethiopia | Cross-sectional | TF | TI, TF and/or TI | no | no | no | no | Checklist for Prevalence Studies | Yes | Yes | Yes | Yes | Unclear | Yes | Yes | Yes | Yes | | | | | | | | | | | [8/9] (89%) | NA | 89% |

TF: trachomatous inflammation--follicular, as measured by the World Health Organization (WHO) Simplified Grading System
TI: trachomatous inflammation--intense, as measured by the WHO Simplified Grading System
RCT: randomised control trial
FPC: follicles, papillae, cicatrices system
SCR: seroconversion rate
DTA: Diagnostic Test Accuracy
*reference number refers to 'references' tab within this workbook, may not match reference number in manuscript
**from the Joanna Briggs Institute (https://jbi.global/critical-appraisal-tools)

0%
0%
0%
0%
0%
0%
0%
0%
