# Supplementary material for: Comparison of Methods for Assessing Chlamydia trachomatis Transmission Intensity: A Systematic Review
Source: Am J Trop Med Hyg. 2026 Sep 22;115(5):903–22. doi: 10.4269/ajtmh.25-0541 (PMC13615270; doi:10.4269/ajtmh.25-0541)
Supplement: Supplementary file 11 [file tpmd250541.SD9.pdf]

S9. TI vs. infection, seroprevalence, and SCR

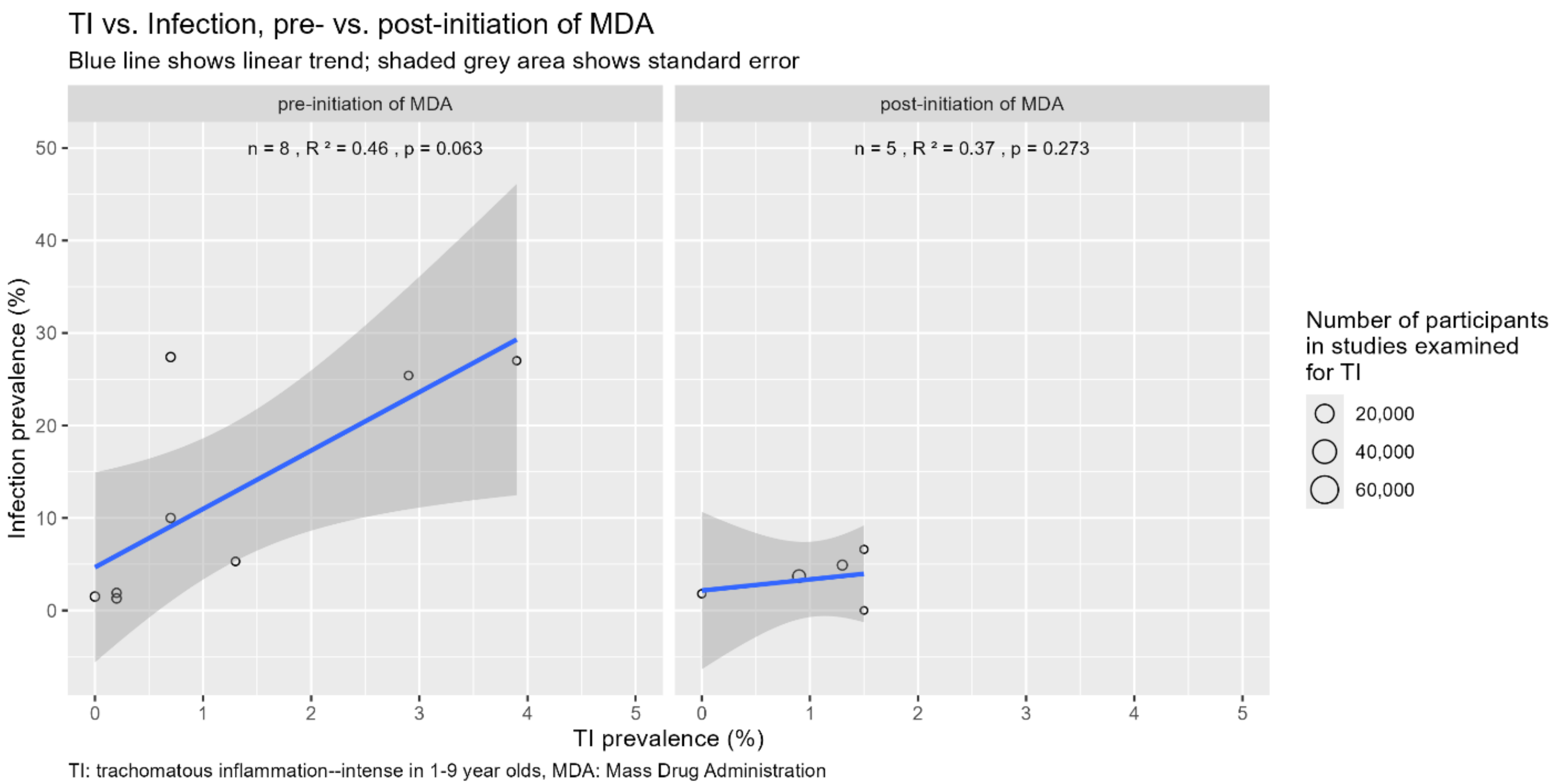

TI vs. Seroprevalence, pre- vs. post-initiation of MDA

Blue line shows linear trend; shaded grey area shows standard error

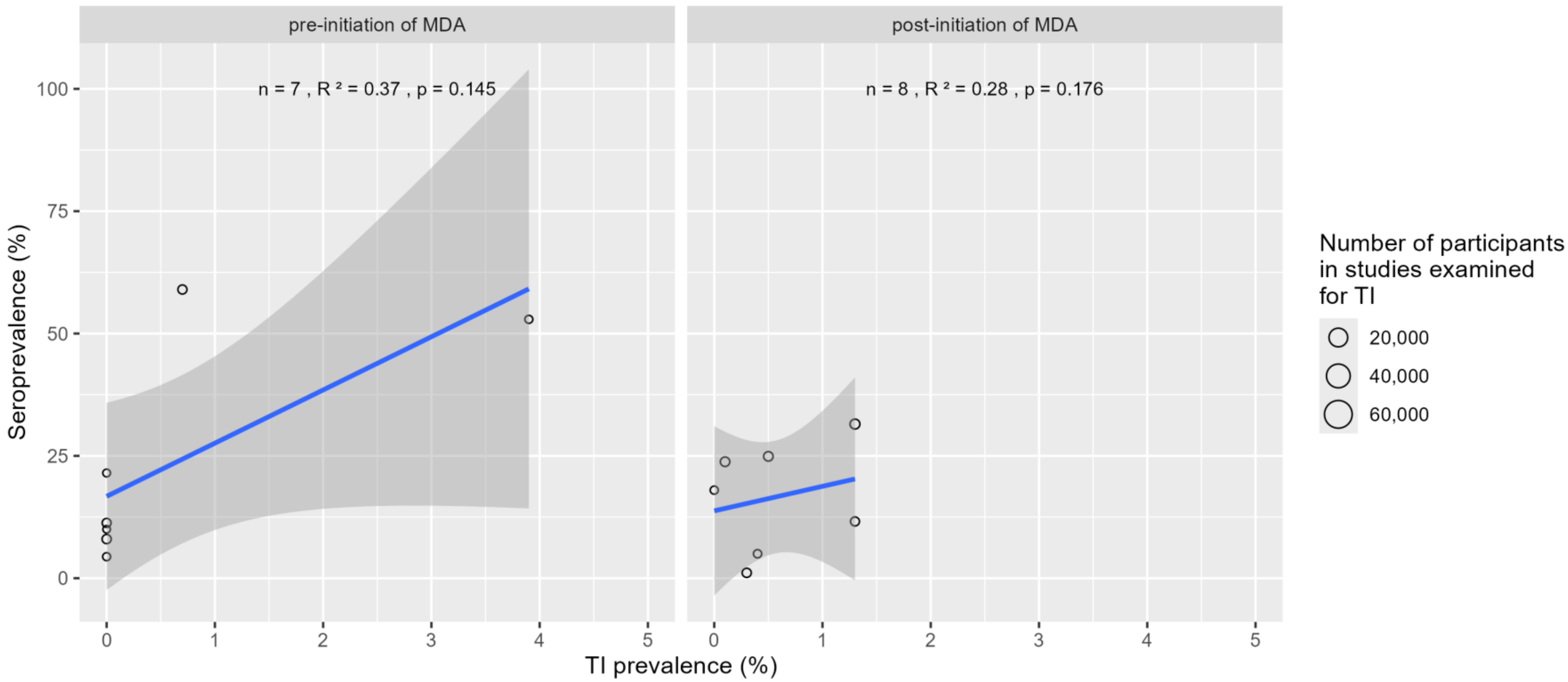

TI: trachomatous inflammation--intense in 1-9 year olds, MDA: Mass Drug Administration

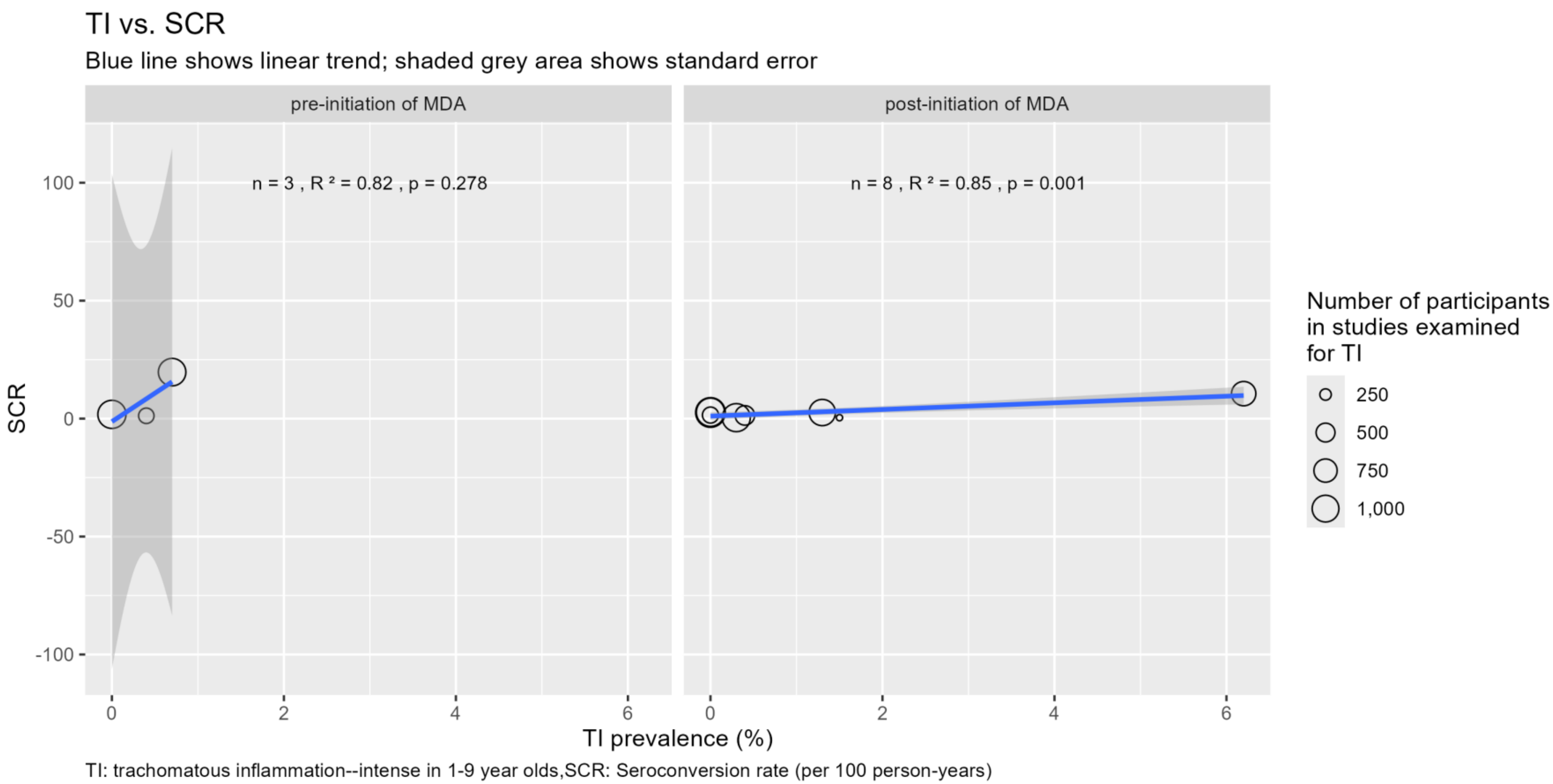
TI vs. SCR
Blue line shows linear trend; shaded grey area shows standard error
pre-initiation of MDA
post-initiation of MDA
n = 3 , R ² = 0.82 , p = 0.278
n = 8 , R ² = 0.85 , p = 0.001
100
50
0
-50
-100
SCR
0
2
4
6
TI prevalence (%)
Number of participants in studies examined for TI
250
500
750
1,000
TI: trachomatous inflammation--intense in 1-9 year olds,SCR: Seroconversion rate (per 100 person-years)
