# Supplementary material for: Comparison of Methods for Assessing Chlamydia trachomatis Transmission Intensity: A Systematic Review
Source: Am J Trop Med Hyg. 2026 Sep 22:tpmd250541. Online ahead of print. doi: 10.4269/ajtmh.25-0541 (PMC13615270; doi:10.4269/ajtmh.25-0541)
Supplement: Supplementary file 2 [file tpmd250541.SD10.pdf]

S10. Table of studies used for programmatic decision-making

Study ID (First author, year)	Country	Survey level	Area Surveyed	Year*	MDA status	Population tested (for TF)	TF prevalence (%)**	*Chlamydia trachomatis* infection prevalence (%)***	SCR (per 100 person-years)^	context	Typical programmatic action based on TF prevalence	Notes
Senyonjo 2018	Ghana	combination of several implementation units	Overall	2015	history of MDA	11,730	0.8 (0.6 - 1)	0.04 (0 - 0.1)	1.3	post-MDA (no unusual epi)	no MDA warranted	infection age range: 1-9 years, SCR context: post-MDA
Senyonjo 2018	Ghana	implementation unit	Wa	2015	history of MDA	1,357	1.1 (0.5 - 1.9)	0		post-MDA (no unusual epi)	no MDA warranted	infection age range: 1-9 years
Senyonjo 2018	Ghana	implementation unit	West Gonja	2015	history of MDA	1,148	1.1 (0.4 - 1.2)	0		post-MDA (no unusual epi)	no MDA warranted	infection age range: 1-9 years
Senyonjo 2018	Ghana	implementation unit	Bole/Sawla-Tuna-Kalpa	2015	history of MDA	1,307	0.5 (0.2 - 1)	0.07 (0 - 0.12)		post-MDA (no unusual epi)	no MDA warranted	infection age range: 1-9 years
Senyonjo 2018	Ghana	implementation unit	Zabzugu-Tatale	2015	history of MDA	1,345	1 (0.5 - 1.5)	0.2 (0 - 0.6)		post-MDA (no unusual epi)	no MDA warranted	infection age range: 1-9 years
Migchelsen 2017	Gambia	implementation unit	Lower River Region	2014	history of MDA	383	3.7		1.5	post-MDA (no unusual epi)	no MDA warranted	SCR context: post-MDA
Hammou 2022	Morocco	implementation unit	Agadez	2019	history of MDA	1,246	0.2 (0 - 0.5)		2.6	post-MDA (no unusual epi)	no MDA warranted	SCR context: post-validation surveillance
Hammou 2022	Morocco	implementation unit	Boumalene Dades	2019	history of MDA	1,121	0.1 (0 - 0.2)		2.7	post-MDA (no unusual epi)	no MDA warranted	SCR context: post-validation surveillance
Burr 2019	Malawi	implementation unit	Mchinji	2014	history of MDA	3,355	6.4 (5.6 - 7.3)	0.4 (0.2 - 0.7)	2.1	post-MDA (no unusual epi)	1 round MDA	infection age range: 1-9 years, SCR context: post-MDA
Burr 2019	Malawi	implementation unit	Chikwawa	2014	history of MDA	2,720	5.1 (4.3 - 6)	0.2 (0.1 - 0.4)	2.8	post-MDA (no unusual epi)	1 round MDA	infection age range: 1-9 years, SCR context: post-MDA
Ngondi 2009	Ethiopia	implementation unit	Dera	2007	history of MDA	398	15 (10 - 20)	1.1 (0.1 - 4)		post-MDA (no unusual epi)	3 rounds MDA	infection age range: 1-6 years
Ngondi 2009	Ethiopia	implementation unit	Estie	2007	history of MDA	382	46 (36 - 53)	1.7 (0.3 - 4.8)		post-MDA (no unusual epi)	3 rounds MDA	infection age range: 1-6 years
Ngondi 2009	Ethiopia	implementation unit	Huleteju Enese	2007	history of MDA	409	36 (29 - 43)	3.9 (1.6 - 7.8)		post-MDA (no unusual epi)	3 rounds MDA	infection age range: 1-6 years
Ngondi 2009	Ethiopia	implementation unit	Enebsie Sarmedir	2007	history of MDA	443	11 (8 - 15)	4.4 (1.9 - 8.6)		post-MDA (no unusual epi)	3 rounds MDA	infection age range: 1-6 years
Ngondi 2009	Ethiopia	implementation unit	Ebinat	2007	history of MDA	416	54 (48 - 61)	4.4 (1.9 - 8.6)		post-MDA (no unusual epi)	3 rounds MDA	infection age range: 1-6 years
Senyonjo 2018	Ghana	implementation unit	Gushiegu	2015	no history of MDA	1,367	0.7 (0.3 - 1.1)	0		pre-MDA	no MDA warranted	infection age range: 1-9 years
Senyonjo 2018	Ghana	implementation unit	Nadowli	2015	no history of MDA	1,363	0.8 (0.3 - 1.5)	0		pre-MDA	no MDA warranted	infection age range: 1-9 years
Gwyn 2021	Togo	implementation unit	Keran	2017	no history of MDA	1,511	0.4 (0.1 - 0.6)		0.7	pre-MDA	no MDA warranted	SCR context: no history of MDA
Gwyn 2021	Togo	implementation unit	Anie	2017	no history of MDA	1,414	0.3		1.0	pre-MDA	no MDA warranted	SCR context: no history of MDA
Gwyn 2021	Democratic Republic of the Congo	implementation unit	Nyemba	2018	no history of MDA	1,578	1.1 (0.5 - 1.8)		1.1	pre-MDA	no MDA warranted	SCR context: no history of MDA
Migchelsen 2017	Gambia	implementation unit	Upper River Region	2014	no history of MDA	359	3.1		1.2	pre-MDA	no MDA warranted	SCR context: no history of MDA
Gwyn 2021	DRC	implementation unit	Manono	2018	no history of MDA	1,591	7.3 (4.2 - 11.6)		10.4	pre-MDA	1 round MDA	SCR context: no history of MDA
Butcher 2020	Kiribati	implementation unit	Tarawa	2016	no history of MDA	1,059	38.2 (35.7 - 41.5)	27.4 (24.7 - 30.1)	19.7	pre-MDA	3 rounds MDA	infection age range: 1-9 years, SCR context: no history of MDA
Lynch 2022 2	Nauru	implementation unit	whole country	2019	no history of MDA	818	21.8 (15.2 - 26.2)	34.5 (30.6 - 38.9)	4.4	pre-MDA	3 rounds MDA	infection age range: 1-9 years, SCR context: no history of MDA
Cama 2017	Kiribati	implementation unit	Kiritimati	2015	no history of MDA	406	28 (24 - 35)	27		pre-MDA	3 rounds MDA	infection age range: 1-9 years
Gwyn 2021	Ethiopia	implementation unit	Alefa	2017	history of MDA	1,106	3.2 (1.4 - 5.7)	0	0.4	unusual epidemiology	no MDA warranted	infection age range: 1-5 years, SCR context: unusual epidemiology
Gwyn 2021	Ethiopia	implementation unit	Woreta Town	2017	history of MDA	532	2.7 (1.5 - 4.5)	0	1.4	unusual epidemiology	no MDA warranted	infection age range: 1-5 years, SCR context: unusual epidemiology
Nash 2018 1	Ethiopia	implementation unit	Injibara	2015	history of MDA	1,019	1.5 (0.4 - 4.9)	0		unusual epidemiology	no MDA warranted	infection age range: 1-5 years
Nash 2022	Ethiopia	implementation unit	Sedie	2018	history of MDA	863	4.1 (2 - 6.8)	0		unusual epidemiology	no MDA warranted	infection age range: 1-5 years
Butcher 2016	Solomon Islands	implementation unit	Temotu, Rennell, Bellona	2013	no history of MDA	1,135	0.26	1.3		unusual epidemiology	no MDA warranted	infection age range: 1-9 years
Cocks 2016/ Macleod 2016	Fiji	implementation unit	Western Division	2013	no history of MDA	1,009	2.8 (1.4 - 4.3)	1.9		unusual epidemiology	no MDA warranted	infection age range: 1-9 years
Kasubi 2020	Tanzania	implementation unit	Mpwapwa	2017	history of MDA	1,000	9.5 (9.6 - 12.2)	4.2		unusual epidemiology	1 round MDA	infection age range: 1-9 years
Gwyn 2021	Ethiopia	implementation unit	Dera	2017	history of MDA	967	14.7 (10 - 20.5)	0	2.6	unusual epidemiology	3 rounds MDA	infection age range: 1-5 years, SCR context: unusual epidemiology
Nash 2022	Ethiopia	implementation unit	Enebessie Sar Midir	2018	history of MDA	808	16.2 (11.7 - 22)	0.3 (0 - 1)		unusual epidemiology	3 rounds MDA	infection age range: 1-5 years
Butcher 2020	Vanuatu	combination of several implementation units	whole country	2016	no history of MDA	1,112	16.5 (14.3 - 18.7)	1.5 (0.8 - 2.2)	1.8	unusual epidemiology	3 rounds MDA	infection age range: 1-9 years, SCR context: no history of MDA
Nash 2022	Ethiopia	implementation unit	Enarge	2018	history of MDA	589	16.9 (12.5 - 23.3)	1.1 (0 - 2.5)		unusual epidemiology	3 rounds MDA	infection age range: 1-5 years

S10. Table of studies used for programmatic decision-making

Study ID (First author, year)	Country	Survey level	Area Surveyed	Year*	MDA status	Population tested (for TF)	TF prevalence (%)**	*Chlamydia trachomatis* infection prevalence (%)***	SCR (per 100 person-years)^	context	Typical programmatic action based on TF prevalence	Notes
Nash 2022	Ethiopia	implementation unit	Huletu Eju Enessie	2018	history of MDA	958	13.4 (10 - 17.7)	1.3 (0 - 3.2)		unusual epidemiology	3 rounds MDA	infection age range: 1-5 years
Gwyn 2021	Ethiopia	implementation unit	Andabet	2017	history of MDA	824	37 (31.1 - 43.3)	11.3	10.6	unusual epidemiology	3 rounds MDA	infection age range: 1-5 years, SCR context: unusual epidemiology
Nash 2017	Ethiopia	combination of several implementation units	16 districts in Amhara	2012	history of MDA	17,124	18.1 (15.8 - 20.7)	2.5 (0 - 5)		unusual epidemiology	3 rounds MDA	infection age range: 1-5 years
Nash 2018 2	Ethiopia	combination of several implementation units	10 zones in Amhara	2011	history of MDA	68,959	26.2 (25.2 - 27.2)	5.7 (4.2 - 7.3)		unusual epidemiology	3 rounds MDA	infection age range: 1-5 years
Nash 2022	Ethiopia	implementation unit	Enemay	2018	history of MDA	647	23.4 (17.8 - 29.7)	3.8 (1.3 - 6.9)		unusual epidemiology	3 rounds MDA	infection age range: 1-5 years
Nash 2022	Ethiopia	implementation unit	Shebel	2018	history of MDA	743	22.9 (16.8 - 27.7)	4 (1.4 - 7.3)		unusual epidemiology	3 rounds MDA	infection age range: 1-5 years
Macleod 2020	Papua New Guinea	implementation unit	West New Britain	2015	no history of MDA	1,000	11.4		0.4	unusual epidemiology	3 rounds MDA	SCR context: unusual epidemiology
Macleod 2020	Papua New Guinea	implementation unit	Western	2015	no history of MDA	790	11.2		1	unusual epidemiology	3 rounds MDA	SCR context: unusual epidemiology
Macleod 2020	Papua New Guinea	implementation unit	Southern Highlands	2015	no history of MDA	1,391	12.2		1.6	unusual epidemiology	3 rounds MDA	SCR context: unusual epidemiology

***Thresholds for infection: low: <1%, medium: 1-2%, high: ≥2%

**Thresholds for TF: low: <5%, medium: 5-9.9%, high: ≥10%

*Year of data collection, if known; otherwise, year of publication

^Thresholds for SCR depend on context: for areas that have no MDA history or have unusual epidemology, low: <=1.6, medium: 1.6-3.8, high: >=3.8; for areas post-MDA (including post-validation), low: <=2.2, medium: 2-2-4.5, high: >=4.5 seroconversions per 100 person-years

Cells appear as red, yellow, and green associated with thresholds pertaining to high, medium, and low categories (respectively), according to thresholds given above.

TF: trachomatous inflammation--follicuar, SCR: Seroconversion Rate, MDA: Mass Drug Administration
