# Supplementary material for: Comparison of Methods for Assessing Chlamydia trachomatis Transmission Intensity: A Systematic Review
Source: Am J Trop Med Hyg. 2026 Sep 22:tpmd250541. Online ahead of print. doi: 10.4269/ajtmh.25-0541 (PMC13615270; doi:10.4269/ajtmh.25-0541)
Supplement: Supplementary file 4 [file tpmd250541.SD2.pdf]

S2. Search terms

1. Search in OVID (for Medline, Embase, and Global Health databases)

1	exp Trachoma/
2	exp Chlamydia trachomatis/
3	trachoma.mp. [mp=title, book title, abstract, original title, name of substance word, subject heading word, floating sub-heading word, keyword heading word, organism supplementary concept word, protocol supplementary concept word, rare disease supplementary concept word, unique identifier, synonyms]
4	ocular trachoma.mp. [mp=title, book title, abstract, original title, name of substance word, subject heading word, floating sub-heading word, keyword heading word, organism supplementary concept word, protocol supplementary concept word, rare disease supplementary concept word, unique identifier, synonyms]
5	**1 or 2 or 3 or 4**
6	Polymerase Chain Reaction/
7	Nucleic Acid Amplification Techniques/
8	infect*.tw.
9	PCR.mp. [mp=title, book title, abstract, original title, name of substance word, subject heading word, floating sub-heading word, keyword heading word, organism supplementary concept word, protocol supplementary concept word, rare disease supplementary concept word, unique identifier, synonyms]
10	LCR.mp. [mp=title, book title, abstract, original title, name of substance word, subject heading word, floating sub-heading word, keyword heading word, organism supplementary concept word, protocol supplementary concept word, rare disease supplementary concept word, unique identifier, synonyms]
11	**6 or 7 or 8 or 9 or 10**
12	Enzyme-Linked Immunosorbent Assay/
13	Serology/
14	lateral flow assay.mp.
15	sero*.mp. [mp=title, book title, abstract, original title, name of substance word, subject heading word, floating sub-heading word, keyword heading word, organism supplementary concept word, protocol supplementary concept word, rare disease supplementary concept word, unique identifier, synonyms]
16	antibod*.mp. [mp=title, book title, abstract, original title, name of substance word, subject heading word, floating sub-heading word, keyword heading word, organism supplementary concept word, protocol supplementary concept word, rare disease supplementary concept word, unique identifier, synonyms]
17	**12 or 13 or 14 or 15 or 16**
18	Photography/
19	photo*.mp. [mp=title, book title, abstract, original title, name of substance word, subject heading word, floating sub-heading word, keyword heading word, organism supplementary concept word, protocol supplementary concept word, rare disease supplementary concept word, unique identifier, synonyms]
20	image.mp. [mp=title, book title, abstract, original title, name of substance word, subject heading word, floating sub-heading word, keyword heading word, organism supplementary concept word, protocol supplementary concept word, rare disease supplementary concept word, unique identifier, synonyms]

21	images.mp. [mp=title, book title, abstract, original title, name of substance word, subject heading word, floating sub-heading word, keyword heading word, organism supplementary concept word, protocol supplementary concept word, rare disease supplementary concept word, unique identifier, synonyms]
22	imaging.mp. [mp=title, book title, abstract, original title, name of substance word, subject heading word, floating sub-heading word, keyword heading word, organism supplementary concept word, protocol supplementary concept word, rare disease supplementary concept word, unique identifier, synonyms]
23	**18 or 19 or 20 or 21 or 22**
24	(TI or TS or pannus or herbert*).mp. [mp=title, book title, abstract, original title, name of substance word, subject heading word, floating sub-heading word, keyword heading word, organism supplementary concept word, protocol supplementary concept word, rare disease supplementary concept word, unique identifier, synonyms]
25	**11 or 17 or 23 or 24**
26	**5 and 25**

2. Scopus search: (TITLE-ABS-KEY (trachoma) AND (TITLE-ABS-KEY (infect* OR pcr OR lcr) OR TITLE-ABS-KEY (sero* OR "lateral flow assay" OR antibod*) OR TITLE-ABS-KEY (photo* OR image OR images OR imaging) OR TITLE-ABS-KEY (ti OR ts OR pannus OR herbert*)))

3. Global Index Medicus search: tw:((tw:(trachoma)) AND (tw:(infect* OR pcr OR lcr OR sero* OR "lateral flow assay" OR antibod* OR photo* OR image OR images OR imaging OR ti OR ts OR pannus OR herbert*)))

4. ETHoS Search: "trachoma"
