# Supplementary material for: Comparison of Methods for Assessing Chlamydia trachomatis Transmission Intensity: A Systematic Review
Source: Am J Trop Med Hyg. 2026 Sep 22:tpmd250541. Online ahead of print. doi: 10.4269/ajtmh.25-0541 (PMC13615270; doi:10.4269/ajtmh.25-0541)
Supplement: Supplementary file 8 [file tpmd250541.SD6.pdf]

S6. Table of included studies

										Risk of Bias checklist items**																					
reference	Country or countries of data collection	Type of study	TF or FPC	other clinical indicators?	photo-graded TF or FPC?	infection?	serology?	SCR?	Critical Appraisal Tool used**	Prevalence study: Was the sample frame appropriate to address the target pop?	Prevalence study: Were study participants sampled in an appropriate way?	Prevalence study: Was the sample size adequate?	Prevalence study: Were the study subjects and setting described in detail?	Prevalence study: Was the data analysis conducted with sufficient coverage of the identified sample?	Prevalence study: Were valid methods used for the identification of the condition(s)?	Prevalence study: Was the condition measured in a standard, reliable way for all participants?	Prevalence study: Was there appropriate statistical analysis?	Prevalence study: Was the response rate adequate, and if not, was the low response rate managed appropriately?	DTA study: Was a consecutive or random sample of patients enrolled?	DTA study: Was a case-control design avoided?	DTA study: Did the study avoid inappropriate exclusions?	DTA: Were the index test results interpreted without knowledge of the results of the reference standard?	DTA: If a threshold was used, was it pre-specified?	DTA: Is the reference standard likely to correctly classify the target condition?	DTA: Were the reference standard results interpreted without knowledge of the results of the index test?	DTA: Was there an appropriate interval between index test and reference standard?	DTA: Did all patients receive the same reference standard?	DTA: Were all patients included in the analysis?	Prevalence study score [meets criteria/total] (%)	DTA study score [meets criteria/total] (%)	Max score (if both checklists used, higher of 2 scores)
Abdilwohab, M. G.; Abebo, Z. H.High prevalence of clinically active trachoma and its associated risk factors among preschool-aged children in arba minch health and demographic surveillance site, southern ethiopia. 2020. Clinical Ophthalmology. , volume: 14, issue: , pages: 3709-3718. Doi: https://dx.doi.org/10.2147/OPTH.S282567.	Ethiopia	Cross-sectional	TF	TI	no	no	no	no	Checklist for Prevalence Studies	Yes	Yes	Yes	Yes	Yes	Yes	Yes	Yes	Yes											[9/9] (100%)	NA	100%
Abdou, A.; Nassirou, B.; Kadri, B.; Moussa, F.; Munoz, B. E.; Opong, E.; West, S. K.Prevalence and risk factors for trachoma and ocular Chlamydia trachomatis infection in Niger. 2007. British Journal of Ophthalmology. , volume: 91, issue: 1, pages: 13-17. Doi: http://dx.doi.org/10.1136/bjo.2006.099507.	Niger	Cross-sectional	TF	TI	no	yes	no	no	Checklist for Prevalence Studies	Yes	Yes	Yes	No	Yes	Yes	Yes	Yes	Yes											[8/9] (89%)	NA	89%
Abebo, T. A.; Tesfaye, D. J.Prevalence and distribution of active trachoma among children 1-9 years old at Leku town, Southern Ethiopia. 2017. Current Pediatric Research. , volume: 21(3), issue: , pages: 507-513. Doi: .	Ethiopia	Cross-sectional	TF	TI	no	no	no	no	Checklist for Prevalence Studies	Yes	Yes	Yes	Yes	Yes	Yes	Yes	Yes	Yes											[9/9] (100%)	NA	100%
Yilikal, Adamu; Semira, FerejiPrevalence of active trachoma among children between 1-9 years, in Woliso town, central Ethiopia. 2018. Ethiopian Journal of Health Development. , volume: 32, issue: 2, pages: unpaginated. Doi: .	Ethiopia	Cross-sectional	TF	TI, TF and/or TI	no	no	no	no	Checklist for Prevalence Studies	Yes	Yes	Yes	Yes	Unclear	Yes	Yes	Yes	Unclear											[7/9] (78%)	NA	78%
Tesfay, Haileselassie; Samson, BayuAltitude - a risk factor for active trachoma in southern Ethiopia. 2007. Ethiopian Medical Journal. , volume: 45, issue: 2, pages: 181-186. Doi: .	Ethiopia	Cross-sectional	TF	TI	no	no	no	no	Checklist for Prevalence Studies	Yes	Yes	No	Yes	Yes	Yes	Yes	Yes	Yes											[8/9] (89%)	NA	89%
Al-Arab, G. E.; Tawfik, N.; El-Gendy, R.; Anwar, W.; Courtright, P.The burden of trachoma in the rural Nile Delta of Egypt: a survey of Menofiya governorate. 2001. British Journal of Ophthalmology. , volume: 85, issue: 12, pages: 1406-1410. Doi: http://dx.doi.org/10.1136/bjo.85.12.1406.	Egypt	Cross-sectional	TF	TI	no	no	no	no	Checklist for Prevalence Studies	Yes	Yes	Yes	No	Yes	Yes	Yes	Yes	Yes											[8/9] (89%)	NA	89%
Wondu, Alemayehu; Muluken, Melese; Fredlander, E.; Alemayehu, Worku; Courtright, P.Active trachoma in children in central Ethiopia: association with altitude. 2005. Transactions of the Royal Society of Tropical Medicine and Hygiene. , volume: 99, issue: 11, pages: 840-843. Doi: http://dx.doi.org/10.1016/j.trstmh.2005.06.013.	Ethiopia	Cross-sectional	TF	TI, TF and TI, TF and/or TI	no	no	no	no	Checklist for Prevalence Studies	Yes	Yes	Yes	Yes	Yes	Yes	Yes	Yes	Yes											[9/9] (100%)	NA	100%
Amer, K.; Muller, A.; Abdelhafiz, H. M.; Al-Khatib, T.; Bakhtiari, A.; Boisson, S.; El-Arab, G. E.; Gad, H.; Gordon, B. A.; Madian, A.; Mahanna, A. T.; Mokhtar, S.; Safa, O. H.; Samy, M.; Shalaby, M.; Taha, Z. A.; Willis, R.; Yacoub, A.; Mamdouh, A. R.; Younis, A. K.; Zoheir, M. B. E.; Courtright, P.; Solomon, A. W.Prevalence of trachoma in four marakez of Elmenia and Bani Suef Governorates, Egypt. 2018. Special Issue: 2017 Global Trachoma Mapping Project.. , volume: 25, issue: Suppl. 1, pages: 70-78. Doi: http://dx.doi.org/10.1080/09286586.2018.1446536.	Egypt	Cross-sectional	TF	TI	no	no	no	no	Checklist for Prevalence Studies	Yes	Yes	Yes	No	Yes	Yes	Yes	Yes	Yes											[8/9] (89%)	NA	89%
Amza, A.; Goldschmidt, P.; Einterz, E.; Huguet, P.; Olmiere, C.; Bensaid, P.; Bella-Assumpta, L.Elimination of active trachoma after two topical mass treatments with azithromycin 1.5% eye drops. 2010. PLoS Neglected Tropical Diseases. , volume: 4, issue: 11, pages: e895. Doi: http://dx.doi.org/10.1371/journal.pntd.0000895.	Cameroon	Cross-sectional	TF	TF and/or TI	no	no	no	no	Checklist for Prevalence Studies	Yes	Yes	Yes	Yes	Yes	Yes	Yes	Yes	Yes											[9/9] (100%)	NA	100%
Amza, A.; Kadri, B.; Nassirou, B.; Stoller, N. E.; Yu, S. N.; Zhou, Z.; Chin, S.; West, S. K.; Bailey, R. L.; Mabey, D. C. W.; Keenan, J. D.; Porco, T. C.; Lietman, T. M.; Gaynor, B. D.Community risk factors for ocular chlamydia infection in Niger: Pre-treatment results from a cluster-randomized trachoma trial. 2012. PLoS Neglected Tropical Diseases. , volume: 6(4) (no pagination), issue: , pages: . Doi: https://dx.doi.org/10.1371/journal.pntd.0001586.	Niger	Cross-sectional	TF	TF and/or TI	no	yes	no	no	Checklist for Prevalence Studies	Yes	Yes	Yes	Yes	Yes	Yes	Yes	Yes	Yes											[9/9] (100%)	NA	100%
Amza, A.; Kadri, B.; Nassirou, B.; Yu, S. N.; Stoller, N. E.; Bhosai, S. J.; Zhou, Z.; McCulloch, C. E.; West, S. K.; Bailey, R. L.; Keenan, J. D.; Lietman, T. M.; Gaynor, B. D.The Easiest Children to Reach Are Most Likely to Be Infected with Ocular Chlamydia trachomatis in Trachoma Endemic Areas of Niger. 2013. PLoS Neglected Tropical Diseases. , volume: 7(1) (no pagination), issue: , pages: . Doi: https://dx.doi.org/10.1371/journal.pntd.0001983.	Niger	Cross-sectional	TF	no	no	yes	no	no	Checklist for Prevalence Studies	Yes	Yes	Unclear	No	Unclear	Yes	Yes	Yes	Unclear											[5/9] (56%)	NA	56%
Amza, A.; Kadri, B.; Nassirou, B.; Cotter, S. Y.; Stoller, N. E.; Zhou, ZhaoXia; Bailey, R. L.; Mabey, D. C.; Porco, T. C.; Keenan, J. D.; Gaynor, B. D.; West, S. K.; Lietman, T. M.A cluster-randomized trial to assess the efficacy of targeting trachoma treatment to children. 2017. Clinical Infectious Diseases. , volume: 64, issue: 6, pages: 743-750. Doi: .	Niger	Cross-sectional within RCT	TF	TI	no	yes	no	no	Checklist for Prevalence Studies	Yes	Yes	Unclear	Yes	Unclear	Yes	Yes	Yes	Unclear											[6/9] (67%)	NA	67%
Amza, A.; Kadri, B.; Nassirou, B.; Cotter, S. Y.; Stoller, N. E.; West, S. K.; Bailey, R. L.; Porco, T. C.; Gaynor, B. D.; Keenan, J. D.; Lietman, T. M.; Oldenburg, C. E.Effectiveness of expanding annual mass azithromycin distribution treatment coverage for trachoma in Niger: a cluster randomised trial. 2018. British Journal of Ophthalmology. , volume: 102, issue: 5, pages: 680-686. Doi: http://dx.doi.org/10.1136/bjophthalmol-2017-310916.	Niger	Cross-sectional within RCT	TF	no	no	yes	no	no	Checklist for Prevalence Studies	Yes	Yes	Unclear	Yes	Unclear	Yes	Yes	Yes	Unclear											[6/9] (67%)	NA	67%
Amza, A.; Kadri, B.; Nassirou, B.; Cotter, S. Y.; Stoller, N. E.; West, S. K.; Bailey, R. L.; Porco, T. C.; Keenan, J. D.; Lietman, T. M.; Oldenburg, C. E.Community-level association between clinical trachoma and ocular chlamydia infection after mass azithromycin distribution in a mesoendemic region of Niger. 2019. Ophthalmic Epidemiology. , volume: 26, issue: 4, pages: 231-237. Doi: http://dx.doi.org/10.1080/09286586.2019.1597129.	Niger	Cross-sectional within RCT	TF	TI	no	yes	no	no	Checklist for Prevalence Studies	Yes	Yes	Unclear	Yes	Unclear	Yes	Yes	Yes	Unclear											[6/9] (67%)	NA	67%
Astle, W. F.; Wiafe, B.; Ingram, A. D.; Mwanga, M.; Glassco, C. B.Trachoma control in Southern Zambia - an international team project employing the SAFE strategy. 2006. Ophthalmic Epidemiology. , volume: 13, issue: 4, pages: 227-236. Doi: http://dx.doi.org/10.1080/09286580600718974.	Zambia	Cross-sectional	TF	TI	no	no	no	no	Checklist for Prevalence Studies	Yes	Unclear, Not sure how villages were chosen for inclusion	Unclear	Yes	Unclear	Yes	Yes	Yes	Unclear											[5/9] (56%)	NA	56%
Ayele, B.; Gebre, T.; Moncada, J.; House, J. I.; Stoller, N. E.; Zhou, Z.; Porco, T. C.; Gaynor, B. D.; Emerson, P. M.; Schachter, J.; Keenan, J. D.Risk factors for ocular chlamydia after three mass azithromycin distributions. 2011. PLoS Neglected Tropical Diseases. , volume: 5(12) (no pagination), issue: , pages: . Doi: https://dx.doi.org/10.1371/journal.pntd.0001441.	Ethiopia	Cross-sectional	TF	TI, TF and/or TI	no	yes	no	no	Checklist for Prevalence Studies	Yes	Yes	Yes	Yes	Yes	Yes	Yes	Yes	Yes											[9/9] (100%)	NA	100%
Ayelgn, K.; Guadu, T.; Getachew, A.Low prevalence of active trachoma and associated factors among children aged 1-9 years in rural communities of Metema District, Northwest Ethiopia: a community based cross-sectional study. 2021. Italian Journal of Pediatrics. , volume: 47(1) (no pagination), issue: , pages: . Doi: https://dx.doi.org/10.1186/s13052-021-01064-x.	Ethiopia	Cross-sectional	TF	TI	no	no	no	no	Checklist for Prevalence Studies	Yes	Yes	Yes	No	Yes	Yes	Yes	Yes	Yes											[8/9] (89%)	NA	89%
Ayena, K. D.; Amza, A.; Agbo, Y. M.; Dorkenoo, M.; Azoumah, K. D.; Dogbe, K. S.; Sognikin, K. S.; Komlagan, S. A.; Mathieu, E.; Bronzan, R.; Anthony, K. K.; Sodahlon, Y. K.; Banla, M. J.; Balo, K. P.[What do we know about trachoma in the economically weakest infantile population of Kara in Togo?]. [French]. 2010. Bulletin de la Societe belge d'ophtalmologie. , volume: , issue: 316, pages: 37-42. Doi: .	Togo	Cross-sectional	TF	TI	no	no	no	no	Checklist for Prevalence Studies	Yes	Unclear how villages/children chosen and if this was random.	Unclear	Yes	Unclear	Yes	Yes	Yes	Unclear											[5/9] (56%)	NA	56%
Ayena, K. D.; Dzidzinyo, K.; Koffi, K. S.; Salou, M.; Amza, A.; Amedome, K. M.; Balo, K. P.[Trachoma rapid assessment in the infantile population of Togo]. [French]. 2011. Medecine tropicale : revue du Corps de sante colonial. , volume: 71(5), issue: , pages: 515-516. Doi: .	Togo	Cross-sectional	TF	TI	no	no	no	no	Checklist for Prevalence Studies	Yes	Unclear how villages/children chosen and if this was random.	Unclear	No	Unclear	Yes	Yes	Yes	Unclear											[4/9] (44%)	NA	44%
Babalola, O. E.; Abiose, A.; Barrie, J. R.The problem of trachoma within an onchocerciasis-endemic zone in Kaduna: need for a multilateral approach?. 2005. Ophthalmic Epidemiology. , volume: 12, issue: 5, pages: 311-9. Doi: .	Nigeria	Cross-sectional within RCT	TF	TI	no	no	no	no	Checklist for Prevalence Studies	Yes	Unclear, villages chosen on basis of proximity to black fly breeding sites.	Unclear	No	Yes	Yes	Yes	Yes	Yes											[6/9] (67%)	NA	67%
Bailey, R. L.; Hampton, T. J.; Hayes, L. J.; Ward, M. E.; Whittle, H. C.; Mabey, D. C. W.Polymerase chain reaction for the detection of ocular chlamydial infection in trachoma-endemic communities. 1994. Journal of Infectious Diseases. , volume: 170, issue: 3, pages: 709-712. Doi: http://dx.doi.org/10.1093/infdis/170.3.709.	Gambia	Cross-sectional	any follicles	no	no	yes	no	no	Checklist for Prevalence Studies	Yes	Yes	Yes	No	Yes	Yes	Yes	Yes	Yes											[8/9] (89%)	NA	89%
Bamani, S.; Dembele, M.; Sankara, D.; Coulibaly, F.; Kamissoko, Y.; Ting, J.; Rotondo, L. A.; Emerson, P. M.; King, J. D.Evaluation of the prevalence of trachoma 12 years after baseline surveys in Kidal Region, Mali. 2010. Tropical Medicine and International Health. , volume: 15, issue: 3, pages: 306-311. Doi: http://dx.doi.org/10.1111/j.1365-3156.2009.02459.x.	Mali	Cross-sectional	TF	TI	no	no	no	no	Checklist for Prevalence Studies	Yes	Yes	Yes	Yes	Yes	Yes	Yes	Yes	Yes											[9/9] (100%)	NA	100%
Bamani, S.; King, J. D.; Dembele, M.; Coulibaly, F.; Sankara, D.; Kamissoko, Y.; Ting, J.; Rotondo, L. A.; Emerson, P. M.Where do we go from here? Prevalence of trachoma three years after stopping mass distribution of antibiotics in the regions of Kayes and Koulikoro, Mali. 2010. PLoS Neglected Tropical Diseases. , volume: 4, issue: 7, pages: e734. Doi: http://dx.doi.org/10.1371/journal.pntd.0000734.	Mali	Cross-sectional	TF	TI	no	no	no	no	Checklist for Prevalence Studies	Yes	Yes	Yes	Yes	Unclear	Yes	Yes	Yes	Unclear											[7/9] (78%)	NA	78%
Bejiga, A.; Alemayehu, W.Prevalence of trachoma and its determinants in Dalocha District, Central Ethiopia. 2001. Ophthalmic Epidemiology. , volume: 8, issue: 44960, pages: 119-25. Doi: .	Ethiopia	Cross-sectional	TF	TI	no	no	no	no	Checklist for Prevalence Studies	Yes	Yes	Yes	Yes	Yes	Yes	Yes	Yes	Yes											[9/9] (100%)	NA	100%
Bella, A. L.; Einterz, E.; Huguet, P.; Bensaid, P.; Amza, A.; Renault, D.Effectiveness and safety of azithromycin 1.5% eye drops for mass treatment of active trachoma in a highly endemic district in Cameroon. 2020. BMJ Open Ophthalmology. , volume: 5, issue: 1, pages: . Doi: 10.1136/bmjophth-2020-000531.	Cameroon	Cross-sectional	TF	TI	no	no	no	no	Checklist for Prevalence Studies	Yes	Yes	Yes	Yes	Yes	Yes	Yes	Yes	Yes											[9/9] (100%)	NA	100%

reference	Country or countries of data collection	Type of study	TF or FPC	other clinical indicators?	photo-graded TF or FPC?	infection?	serology?	SCR?	Critical Appraisal Tool used**	Risk of Bias checklist items** Prevalence study: Was the sample frame appropriate to address the target pop?	Prevalence study: Were study participants sampled in an appropriate way?	Prevalence study: Was the sample size adequate?	Prevalence study: Were the study subjects and setting described in detail?	Prevalence study: Was the data analysis conducted with sufficient coverage of the identified sample?	Prevalence study: Were valid methods used for the identification of the condition[s]?	Prevalence study: Was the condition measured in a standard, reliable way for all participants?	Prevalence study: Was there appropriate statistical analysis?	Prevalence study: Was the response rate adequate, and if not, was the low response rate managed appropriately?	DTA study: Was a consecutive or random sample of patients enrolled?	DTA study: Was a case-control design avoided?	DTA study: Did the study avoid inappropriate exclusions?	DTA study: Were the index test results interpreted without knowledge of the results of the reference standard?	DTA: If a threshold was used, was it pre-specified?	DTA: Is the reference standard likely to correctly classify the target condition?	DTA: Were the reference standard results interpreted without knowledge of the results of the index test?	DTA: Was there an appropriate interval between index test and reference standard?	DTA: Did all patients receive the same reference standard?	DTA: Were all patients included in the analysis?	Prevalence study score [meets criteria/total] (%)	DTA study score [meets criteria/total] (%)	Max score (if both checklists used, higher of 2 scores)
Yemane, Berhane; Alemayehu, Worku; Abebe, Bejiga; Liknaw, Adamu; Wondu, Alemayehu; Amir, Bedri; Zegeye, Haile; Allehone, Ayalew; Yilikal, Adamu; Teshome, Gebre; Kebede, T. D.; West, E.; West, S.Prevalence of trachoma in Ethiopia. 2007. Ethiopian Journal of Health Development. , volume: 21, issue: 3, pages: 211-215. Doi: .	Ethiopia	Cross-sectional	TF	TI, TF and/or TI	no	no	no	no	Checklist for Prevalence Studies	Yes	Yes	Unclear	No	Unclear	Yes	Yes	Yes	Unclear											[5/9] (56%)	NA	56%
Bhosai, S. J.; Amza, A.; Beido, N.; Bailey, R. L.; Keenan, J. D.; Gaynor, B. D.; Lietman, T. M.Application of smartphone cameras for detecting clinically active trachoma. 2012. British Journal of Ophthalmology. , volume: 96, issue: 10, pages: 1350-1351. Doi: 10.1136/bjophthalmol-2012-302050.	Niger	Cross-sectional	TF	TI	yes	no	no	no	Both	Yes	Yes	Yes	Yes	Yes	Yes	Yes	Unclear	Unclear	Random	Yes	Yes	Yes	Yes	Unclear - LCA used	Yes	Yes	Yes	Yes	[7/9] (78%)	[8/10] (80%)	80%
Bid, R.; Sandi, F.; Goodhew, B.; Massae, P.; Alexander, N.; Laswai, A.; Makupa, W.; Molina, S.; Holland, M.; Mabey, D.; Lammie, P. J.; Solomon, A.; Martin, D.Piloting serological tools for trachoma postelimination surveillance. 2013. American Journal of Tropical Medicine and Hygiene. , volume: 1), issue: , pages: 12. Doi: .	Tanzania	Cross-sectional	TF	TI	no	no	no	no	Checklist for Prevalence Studies	Unclear	Unclear	Unclear	No	Unclear	Unclear	Unclear	Unclear	Unclear											[0/9] (0%)	NA	0%
Bio, A. A.; Boko, P. M.; Dossou, Y. A.; Tougoue, J. J.; Kabore, A.; Sounouvou, I.; Biaou, J. E.; Nizigama, L.; Courtright, P.; Solomon, A. W.; Rotondo, L.; Batcho, W.; Kinde-Gazard, D.Prevalence of trachoma in northern Benin: results from 11 population-based prevalence surveys covering 26 districts. 2017. Ophthalmic Epidemiology. , volume: 24, issue: 4, pages: 265-273. Doi: http://dx.doi.org/10.1080/09286586.2017.1279337.	Benin	Cross-sectional	TF	TI	no	no	no	no	Checklist for Prevalence Studies	Yes	Yes	Yes	No	Yes	Yes	Yes	Yes	Yes											[8/9] (89%)	NA	89%
Bobo, L.; Munoz, B.; Viscidi, R.; Quinn, T.; Mkocha, H.; West, S.Diagnosis of Chlamydia trachomatis eye infection in Tanzania by polymerase chain reaction/enzyme immunoassay. 1991. Lancet. , volume: 338, issue: Oct. 5, pages: 847-850. Doi: .	Tanzania	Cross-sectional	TF	TI	no	yes	no	no	Checklist for Prevalence Studies	Yes	Yes	Unclear	No	Unclear	Yes	Yes	Yes	Unclear											[5/9] (56%)	NA	56%
Brady, C. J.; Wolle, M. A.; Naufal, F.; Mkocha, H.; West, S. K.Crowdsourcing can match field grading validity for follicular trachoma. 2021. Investigative Ophthalmology and Visual Science Conference: Annual Meeting Association for Research in Vision and Ophthalmology, ARVO. , volume: 62, issue: 8, pages: . Doi: .	Tanzania	Cross-sectional	TF	no	yes	no	no	no	Both	Unclear	Unclear	Unclear	Unclear	Unclear	Unclear	Unclear	Unclear	Unclear	Unclear	Unclear	Unclear	Unclear	Unclear	Unclear	Unclear	Unclear	Unclear	Unclear	[0/9] (0%)	[0/10] (0%)	0%
Burr, S. E.; Hart, J. D.; Edwards, T.; Baldeh, I.; Bojang, E.; Harding-Esch, E. M.; Holland, M. J.; Lietman, T. M.; West, S. K.; Mabey, D. C. W.; Sillah, A.; Bailey, R. L.Association between ocular bacterial carriage and follicular trachoma following mass azithromycin distribution in The Gambia. 2013. PLoS Neglected Tropical Diseases. , volume: 7, issue: 7, pages: e2347. Doi: http://dx.doi.org/10.1371/journal.pntd.0002347.	Gambia	Cross-sectional	TF	no	no	yes	no	no	Both	Yes	Yes	Yes	Yes	Yes	Yes	Yes	Yes	Yes	Random	Yes	Yes	Yes	Yes	Yes	Yes	Yes	Yes	Yes	[9/9] (100%)	[9/10] (90%)	100%
Burr, S. E.; Hart, J.; Samikwa, L.; Chaima, D.; Cooley, G.; Martin, D.; Masika, M.; Solomon, A. W.; Bailey, R. L.; Kalua, K.Pgp3 seroprevalence and associations with active trachoma and ocular Chlamydia trachomatis infection in Malawi: cross-sectional surveys in six evaluation units. 2019. PLoS Neglected Tropical Diseases. , volume: 13, issue: 10, pages: e0007749. Doi: http://dx.doi.org/10.1371/journal.pntd.0007749.	Malawi	Cross-sectional	TF	no	no	yes	yes	yes	Both	Yes	Yes	Yes	No	Unclear	Yes	Yes	Yes	Unclear	Random	Yes	Yes	Yes	Yes	Yes	Yes	Yes	Yes	Yes	[6/9] (67%)	[9/10] (90%)	90%
Burton, M. J.; Holland, M. J.; Makalo, P.; Aryee, E. A. N.; Sillah, A.; Cohuet, S.; Natividad, A.; Alexander, N. D. E.; Mabey, D. C. W.; Bailey, R. L.Profound and sustained reduction in Chlamydia trachomatis in The Gambia: a five-year longitudinal study of trachoma endemic communities. 2010. PLoS Neglected Tropical Diseases. , volume: 4, issue: 10, pages: e835. Doi: http://dx.doi.org/10.1371/journal.pntd.0000835.	Gambia	Cross-sectional within longitudinal	TF	no	no	yes	no	no	Checklist for Prevalence Studies	Yes	Yes	Unclear	No	Yes	Yes	Yes	Yes	Yes											[7/9] (78%)	NA	78%
Burton, M. J.; Ramadhani, A.; Weiss, H. A.; Hu, V.; Massae, P.; Burr, S. E.; Shangali, W.; Holland, M. J.; Mabey, D. C. W.; Bailey, R. L.Active trachoma is associated with increased conjunctival expression of IL17A and profibrotic cytokines. 2011. Infection and Immunity. , volume: 79, issue: 12, pages: 4977-4983. Doi: http://dx.doi.org/10.1128/IAI.05718-11.	Tanzania	Cross-sectional	TF	TI	no	yes	no	no	Both	Yes	Yes	Unclear	No	Yes	Yes	Yes	Yes	Yes	Unclear how village chosen	Yes	Yes	Unclear	Yes	Yes	Yes	Yes	Yes	Yes	[7/9] (78%)	[8/10] (80%)	80%
Butcher, R. M. R.; Sokana, O.; Jack, K.; Macleod, C. K.; Marks, M. E.; Kalae, E.; Sui, L.; Russell, C.; Tutill, H. J.; Williams, R. J.; Breuer, J.; Willis, R.; Mesurier, R. T. le; Mabey, D. C. W.; Solomon, A. W.; Roberts, C. H.Low prevalence of conjunctival infection with Chlamydia trachomatis in a treatment-naive trachoma-endemic region of the Solomon Islands. 2016. PLoS Neglected Tropical Diseases. , volume: 10, issue: 9, pages: e0004863. Doi: .	Solomon Islands	Cross-sectional	TF	TI	no	yes	no	no	Checklist for Prevalence Studies	Yes	Yes	Yes	Yes	Yes	Yes	Yes	Yes	Yes											[9/9] (100%)	NA	100%
Butcher, R.Using alternate indicators to define need for public health intervention for trachoma: Evidence from the Pacific Islands. 2017. . , volume: , issue: , pages: . Doi: .	Fiji	Cross-sectional	TF	TI	no	no	no	no	Checklist for Prevalence Studies	Yes	Yes	Yes	Yes	Unclear	Yes	Yes	Yes	Unclear											[7/9] (78%)	NA	78%
Butcher, R.; Sokana, O.; Jack, K.; Sui, L.; Russell, C.; Last, A.; Martin, D. L.; Burton, M. J.; Solomon, A. W.; Mabey, D. C. W.; Roberts, C. H.Clinical signs of trachoma are prevalent among Solomon Islanders who have no persistent markers of prior infection with Chlamydia trachomatis [version 1; referees: 2 approved]. 2018. Wellcome Open Research. , volume: 3, issue: , pages: . Doi: 10.12688/wellcomeopenres.13423.1.	Solomon Islands	Cross-sectional	TF	TI	no	yes	yes	no	Both	Unclear; only villages with previous TF rates >10% were chosen	Yes	Yes	Yes	Yes	Yes	Yes	Yes	Yes	Random	Yes	Yes	Yes	Yes	Yes	Yes	Yes	Yes	Yes	[8/9] (89%)	[9/10] (90%)	90%
Butcher, R.; Handley, B.; Garae, M.; Taoaba, R.; Pickering, H.; Bong, A.; Sokana, O.; Burton, M. J.; Sepulveda, N.; Cama, A.; Mesurier, R. le; Solomon, A. W.; Mabey, D.; Taleo, F.; Tekeraoi, R.; Roberts, C. H.Ocular Chlamydia trachomatis infection, anti-Pgp3 antibodies and conjunctival scarring in Vanuatu and Tarawa, Kiribati before antibiotic treatment for trachoma. 2020. Journal of Infection. , volume: 80, issue: 4, pages: 454-461. Doi: http://dx.doi.org/10.1016/j.jinf.2020.01.015.	Kiribati, Vanuatu	Cross-sectional	TF	TI	no	yes	yes	yes	Both	Yes	Yes	Yes	Yes	Yes	Yes	Yes	Yes	Yes	Random	Yes	Yes	Yes	Yes	Yes	Yes	Yes	Yes	Yes	[9/9] (100%)	[9/10] (90%)	100%
Abreu Caligaris, L. S.; Miyake Morimoto, W. T.; Medina, N. H.; Waldman, E. A.Trachoma prevalence and risk factors among preschool children in a central area of the city of Sao Paulo, Brazil. 2006. Ophthalmic Epidemiology. , volume: 13(6), issue: , pages: 365-370. Doi: https://dx.doi.org/10.1080/09286580601013078.	Brazil	Cross-sectional	TF	TI	no	no	no	no	Checklist for Prevalence Studies	Yes	Yes	Yes	No	Yes	Yes	Yes	Yes	Yes											[8/9] (89%)	NA	89%
Cama, A.; Muller, A.; Taoaba, R.; Butcher, R. M. R.; Itibita, I.; Migchelsen, S. J.; Kiauea, T.; Pickering, H.; Willis, R.; Roberts, C. H.; Bakhtiari, A.; Mesurier, R. T. le; Alexander, N. D. E.; Martin, D. L.; Tekeraoi, R.; Solomon, A. W.Prevalence of signs of trachoma, ocular Chlamydia trachomatis infection and antibodies to Pgp3 in residents of Kiritimati Island, Kiribati. 2017. PLoS Neglected Tropical Diseases. , volume: 11, issue: 9, pages: e0005863. Doi: http://dx.doi.org/10.1371/journal.pntd.0005863.	Kiribati	Cross-sectional	TF	TI	no	yes	yes	no	Checklist for Prevalence Studies	Yes	Yes	Yes	No	Yes	Yes	Yes	Yes	Yes											[8/9] (89%)	NA	89%
Chen, X.; Munoz, B.; Mkocha, H.; Gaydos, C. A.; Dize, L.; Quinn, T. C.; West, S. K.Risk of seroconversion and seroreversion of antibodies to Chlamydia trachomatis pgp3 in a longitudinal cohort of children in a low trachoma prevalence district in Tanzania. 2022. PLoS Neglected Tropical Diseases. , volume: 16(7) (no pagination), issue: , pages: . Doi: https://dx.doi.org/10.1371/journal.pntd.0010629.	Tanzania	Cross-sectional within longitudinal	TF	TI	no	yes	yes	yes	Checklist for Prevalence Studies	Yes	Yes	Unclear	Yes	Yes	Yes	Yes	Yes	Yes											[8/9] (89%)	NA	89%
Cochereau, I.; Goldschmidt, P.; Goepogui, A.; Afghani, T.; Delval, L.; Pouliquen, P.; Bourcier, T.; Robert, P. Y.Efficacy and safety of short duration azithromycin eye drops versus azithromycin single oral dose for the treatment of trachoma in children: a randomised, controlled, double-masked clinical trial. 2007. British Journal of Ophthalmology. , volume: 91, issue: 5, pages: 667-672. Doi: http://dx.doi.org/10.1136/bjo.2006.099275.	Guinea and Pakistan	Cross-sectional within RCT	TF	TF and/or TI	no	no	no	no	Checklist for Prevalence Studies	No	Unclear	Unclear	Yes	Unclear	Yes	Yes	Yes	Unclear											[4/9] (44%)	NA	44%
Cocks, N.; Rainima-Qaniuci, M.; Yalen, C.; Macleod, C.; Nakolinivalu, A.; Migchelsen, S.; Roberts, C. H.; Butcher, R.; Kama, M.; Mabey, D.; Marks, M.Community seroprevalence survey for yaws and trachoma in the Western Division of Fiji. 2016. Transactions of the Royal Society of Tropical Medicine and Hygiene. , volume: 110, issue: 10, pages: 582-587. Doi: http://dx.doi.org/10.1093/trstmh/trw069.	Fiji	Cross-sectional	TF	TI	no	yes	yes	no	Checklist for Prevalence Studies	Yes	Yes	Yes	Yes	Yes	Yes	Yes	Yes	Yes											[9/9] (100%)	NA	100%
Cox, J. T.; Mkocha, H.; Munoz, B.; West, S.Prevalence of trachomatous scarring in children in a formerly endemic district of Tanzania. 2017. Investigative Ophthalmology and Visual Science. Conference. , volume: 58, issue: 8, pages: . Doi: .	Tanzania	Cross-sectional	TF	no	no	yes	no	no	Checklist for Prevalence Studies	Yes	Unclear	Unclear	No	Unclear	Unclear	Unclear	Unclear	Unclear											[1/9] (11%)	NA	11%
Cromwell, E. A.; Amza, A.; Kadri, B.; Beidou, N.; King, J. D.; Sankara, D.; Mosher, A. W.; Hassan, S.; Kane, S.; Emerson, P. M.Trachoma prevalence in Niger: results of 31 district-level surveys. 2014. Transactions of the Royal Society of Tropical Medicine and Hygiene. , volume: 108, issue: 1, pages: 42-48. Doi: http://dx.doi.org/10.1093/trstmh/trt101.	Niger	Cross-sectional	TF	TI	no	no	no	no	Checklist for Prevalence Studies	Yes	Yes	Yes	No	Yes	Yes	Yes	Yes	Yes											[8/9] (89%)	NA	89%
Cumberland, P.; Girum, Hailu; Todd, J.Active trachoma in children aged three to nine years in rural communities in Ethiopia: prevalence, indicators and risk factors. 2005. Transactions of the Royal Society of Tropical Medicine and Hygiene. , volume: 99, issue: 2, pages: 120-127. Doi: http://dx.doi.org/10.1016/j.trstmh.2004.03.011.	Ethiopia	Cross-sectional	TF	TI	no	no	no	no	Checklist for Prevalence Studies	Yes	Yes	Unclear	No	Unclear	Yes	Yes	Yes	Unclear											[5/9] (56%)	NA	56%
Cumberland, P.; Edwards, T.; Hailu, G.; Harding-Esch, E.; Andreasen, A.; Mabey, D.; Todd, J.The impact of community level treatment and preventative interventions on trachoma prevalence in rural Ethiopia. 2008. International Journal of Epidemiology. , volume: 37, issue: 3, pages: 549-558. Doi: http://dx.doi.org/10.1093/ije/dyn045.	Ethiopia	Cross-sectional	TF	no	no	yes	no	no	Checklist for Prevalence Studies	Yes	Yes	Unclear	Yes	Unclear	Yes	Yes	Yes	Unclear											[6/9] (67%)	NA	67%

S6. Table of included studies

										Risk of Bias checklist items**																					
reference	Country or countries of data collection	Type of study	TF or FPC	other clinical indicators?	photo-graded TF or FPC?	infection?	serology?	SCR?	Critical Appraisal Tool used**	Prevalence study: Was the sample frame appropriate to address the target pop?	Prevalence study: Were study participants sampled in an appropriate way?	Prevalence study: Was the sample size adequate?	Prevalence study: Were the study subjects and setting described in detail?	Prevalence study: Was the data analysis conducted with sufficient coverage of the identified sample?	Prevalence study: Were valid methods used for the identification of the condition(s)?	Prevalence study: Was the condition measured in a standard, reliable way for all participants?	Prevalence study: Was there appropriate statistical analysis?	Prevalence study: Was the response rate adequate, and if not, was the low response rate managed appropriately?	DTA study: Was a consecutive or random sample of patients enrolled?	DTA study: Was a case-control design avoided?	DTA study: Did the study avoid inappropriate exclusions?	DTA: Were the index test results interpreted without knowledge of the results of the reference standard?	DTA: If a threshold was used, was it pre-specified?	DTA: Is the reference standard likely to correctly classify the target condition?	DTA: Were the reference standard results interpreted without knowledge of the results of the index test?	DTA: Was there an appropriate interval between index test and reference standard?	DTA: Did all patients receive the same reference standard?	DTA: Were all patients included in the analysis?	Prevalence study score [meets criteria/total] (%)	DTA study score [meets criteria/total] (%)	Max score (if both checklists used, higher of 2 scores)
Damasceno, R. W. F.; Santos, R. R.; Cavalcanti, T. R. T.; Hida, R. Y.; Santos, M. J.; Santos, A. M. C.; Dantas, P. E. C.Trachoma: epidemiologic study of scholars from Alagoas state - Brazil. 2009. Arquivos Brasileiros de Oftalmologia. , volume: 72, issue: 3, pages: 355-359. Doi: http://dx.doi.org/10.1590/S0004-27492009000300014.	Brazil	Cross-sectional	TF	TI	no	no	no	no	Checklist for Prevalence Studies	Yes	Yes	Yes	Yes	Unclear	Yes	Yes	Yes	Unclear											[7/9] (78%)	NA	78%
Datta, P.; Frost, E.; Peeling, R.; Masinde, S.; Deslandes, S.; Echelu, C.; Wamola, I.; Brunham, R. C.Ophthalmia neonatorum in a trachoma endemic area. 1994. Sexually Transmitted Diseases. , volume: 21, issue: 1, pages: 44930. Doi: .	Kenya	Cross-sectional	TF	TI	no	yes	no	no	Checklist for DTA studies										Consecutive	Yes	No	Unclear	NA	Yes	Yes	Yes	Yes	No	NA	[5/10] (50%)	50%
Debrah, O.; Mensah, E. O.; Senyonjo, L.; Souza, D. K. de; Hervie, T. E.; Agyemang, D.; Bakajika, D.; Marfo, B.; Ahorsu, F.; Wanye, S.; Bailey, R.; Koroma, J. B.; Aboe, A.; Biritwum, N. K.Elimination of trachoma as a public health problem in Ghana: providing evidence through a pre-validation survey. 2017. PLoS Neglected Tropical Diseases. , volume: 11, issue: 12, pages: e0006099. Doi: http://dx.doi.org/10.1371/journal.pntd.0006099.	Ghana	Cross-sectional	TF	TI	no	no	no	no	Checklist for Prevalence Studies	Yes	Yes	Yes	Yes	Yes	Yes	Yes	Yes	Yes											[9/9] (100%)	NA	100%
Derrick, T.; Holland, M. J.; Cassama, E.; Markham-David, R.; Nabicassa, M.; Marks, M.; Bailey, R. L.; Last, A. R.Can corneal pannus with trachomatous inflammation - follicular be used in combination as an improved specific clinical sign for current ocular Chlamydia trachomatis infection?. 2016. Parasites and Vectors. , volume: 9, issue: 30, pages: . Doi: .	Guinea Bissau	Case-control	TF	no	no	yes	no	no	Checklist for DTA studies										Unclear	No	Yes	Unclear	Yes	Yes	Unclear	Yes	Yes	Yes	NA	[6/10] (60%)	60%
Derrick, T.; Last, A. R.; Burr, S. E.; Roberts, C. H.; Nabicassa, M.; Cassama, E.; Bailey, R. L.; Mabey, D. C. W.; Burton, M. J.; Holland, M. J.Inverse relationship between microRNA-155 and -184 expression with increasing conjunctival inflammation during ocular Chlamydia trachomatis infection. 2016. BMC Infectious Diseases. , volume: 16, issue: 60, pages: . Doi: .	Guinea Bissau	Case-control	TF	P3 (FPC)	no	yes	no	no	Checklist for DTA studies										Unclear	No	Yes	Yes	Yes	Yes	Unclear	Yes	Yes	Yes	NA	[7/10] (70%)	70%
Derrick, T. R.; Sandetskaya, N.; Pickering, H.; Kolsch, A.; Ramadhani, A.; Mafuru, E.; Massae, P.; Malisa, A.; Mtuy, T.; Burton, M. J.; Holland, M. J.; Kuhlmeier, D.DjinniChip: evaluation of a novel molecular rapid diagnostic device for the detection of Chlamydia trachomatis in trachoma-endemic areas. 2020. Parasites and Vectors. , volume: 13, issue: 533, pages: . Doi: http://dx.doi.org/10.1186/s13071-020-04414-6.	Tanzania	Cross-sectional	TF	P3 (FPC)	no	yes	no	no	Checklist for DTA studies										All children from selected villages	Yes	Yes	Yes	Yes	Yes	Unclear	Yes	Yes	Yes	NA	[8/10] (80%)	80%
de Sousa Meneghim, R. L. F.; Viveiros, M. M. H.; Galindo-Ferreiro, A.; Padovani, C. R.; Schellini, S. A.Sequelae from Epidemic Viral Conjunctivitis Can Be Associated with Inflammatory Trachoma in Schoolchildren?. 2018. Seminars in Ophthalmology. , volume: 33, issue: 2, pages: 219-222. Doi: 10.1080/08820538.2016.1208762.	Brazil	Cross-sectional	TF	TI	no	no	no	no	Checklist for Prevalence Studies	No (what about private schools?)	Yes	Yes	No	Yes	Yes	Yes	Yes	Yes											[7/9] (78%)	NA	78%
Dorkenoo, A. M.; Bronzan, R. N.; Ayena, K. D.; Anthony, G.; Agbo, Y. M.; Sognikin, K. S. E.; Dogbe, K. S.; Amza, A.; Sodahlon, Y.; Mathieu, E.Nationwide integrated mapping of three neglected tropical diseases in Togo: countrywide implementation of a novel approach. 2012. Tropical Medicine and International Health. , volume: 17, issue: 7, pages: 896-903. Doi: http://dx.doi.org/10.1111/j.1365-3156.2012.03004.x.	Togo	Cross-sectional	TF	TI	no	no	no	no	Checklist for Prevalence Studies	Yes	Unclear	Unclear	No	Unclear	Yes	Yes	Yes	Unclear											[4/9] (44%)	NA	44%
Ahmed Badei, Duale; Nebiyu Negussu, Ayele; Macleod, C. K.; Amir Bedri, Kello; Zelalem Eshetu, Gezachew; Amsalu, Binegdie; Michael, Dejene; Wondu, Alemayehu; Flueckiger, R. M.; Massae, P. A.; Willis, R.; Biruck Kebede, Negash; Solomon, A. W.Epidemiology of trachoma and its implications for implementing the "SAFE" strategy in Somali Region, Ethiopia: results of 14 population-based prevalence surveys. 2018. Special Issue: 2017 Global Trachoma Mapping Project.. , volume: 25, issue: Suppl. 1, pages: 25-32. Doi: http://dx.doi.org/10.1080/09286586.2017.1409358.	Ethiopia	Cross-sectional	TF	TI	no	no	no	no	Checklist for Prevalence Studies	Yes	Yes	Unclear	Yes	Yes	Yes	Yes	Yes	Yes											[8/9] (89%)	NA	89%
Edwards, T.; Smith, J.; Sturrock, H. J. W.; Kur, L. W.; Sabasio, A.; Finn, T. P.; Lado, M.; Haddad, D.; Kolaczinski, J. H.Prevalence of trachoma in Unity State, South Sudan: results from a large-scale population-based survey and potential implications for further surveys. 2012. PLoS Neglected Tropical Diseases. , volume: 6, issue: 4, pages: e1585. Doi: http://dx.doi.org/10.1371/journal.pntd.0001585.	South Sudan	Cross-sectional	TF	TI	no	no	no	no	Checklist for Prevalence Studies	Yes	Yes	Unclear	Yes	Yes	Yes	Yes	Yes	Yes											[8/9] (89%)	NA	89%
Ejigu, M.; Kariuki, M. M.; Ilako, D. R.; Gelaw, Y.Rapid trachoma assessment in Kersa District, Southwest Ethiopia. 2013. Ethiopian Journal of Health Sciences. , volume: 23, issue: 1, pages: 44935. Doi: .	Ethiopia	Cross-sectional	TF	TI	no	no	no	no	Checklist for Prevalence Studies	Yes	Unclear - convenience sampling of sub-districts as per TRA methodology	Yes	Yes	Unclear	Yes	Yes	Yes	Unclear											[6/9] (67%)	NA	67%
Ervin, A. M.; Mkocha, H.; Munoz, B.; Dreger, K.; Dize, L.; Gaydos, C.; Quinn, T. C.; West, S. K.Surveillance and azithromycin treatment for newcomers and travelers evaluation (ASANTE) trial: design and baseline characteristics. 2016. Ophthalmic Epidemiology. , volume: 23, issue: 6, pages: 347-353. Doi: http://dx.doi.org/10.1080/09286586.2016.1238947.	Tanzania	Cross-sectional	TF	TI, TF and TI	no	yes	no	no	Checklist for Prevalence Studies	Yes	Yes	Unclear	Yes	Unclear	Yes	Yes	Yes	Unclear											[6/9] (67%)	NA	67%
Faal, N.; Bailey, R. L.; Sarr, I.; Joof, H.; Mabey, D. C. W.; Holland, M. J.Temporal cytokine gene expression patterns in subjects with trachoma identify distinct conjunctival responses associated with infection. 2005. Clinical and Experimental Immunology. , volume: 142, issue: 2, pages: 347-353. Doi: http://dx.doi.org/10.1111/j.1365-2249.2005.02917.x.	Gambia	Cross-sectional	TF	TF and TI	no	yes	no	no	Checklist for DTA studies										Unclear	Yes	Yes	Unclear	Yes	Yes	Yes	Yes	Yes	Yes	NA	[8/10] (80%)	80%
Faal, N.; Bailey, R. L.; Jeffries, D.; Joof, H.; Sarr, I.; Laye, M.; Mabey, D. C.; Holland, M. J.Conjunctival FOXP3 expression in trachoma: do regulatory T cells have a role in human ocular Chlamydia trachomatis infection?. 2006. PLoS Medicine / Public Library of Science. , volume: 3, issue: 8, pages: e266. Doi: .	Gambia	Cross-sectional	TF	TI	no	yes	no	no	Checklist for DTA studies										Unclear	Yes	Yes	Unclear	Yes	Yes	Yes	Yes	Yes	Unclear	NA	[7/10] (70%)	70%
Favacho, J.; Alves da Cunha, A. J. L.; Gomes, S. T. M.; Freitas, F. B.; Queiroz, M. A. F.; Vallinoto, A. C. R.; Ishak, R.; Ishak, M. D. O. G.Prevalence of trachoma in school children in the Marajo Archipelago, Brazilian Amazon, and the impact of the introduction of educational and preventive measures on the disease over eight years. 2018. PLoS Neglected Tropical Diseases. , volume: 12(2) (no pagination), issue: , pages: . Doi: https://dx.doi.org/10.1371/journal.pntd.0006282.	Brazil	Cross-sectional	TF	TI	no	no	no	no	Checklist for Prevalence Studies	Yes	Yes	Unclear	Yes	Unclear	Yes	Yes	Yes	Unclear											[6/9] (67%)	NA	67%
Faye, M.; Kuper, H.; Dineen, B.; Bailey, R.Rapid assessment for prioritisation of trachoma control at community level in one district of the Kaolack Region, Senegal. 2006. Transactions of the Royal Society of Tropical Medicine and Hygiene. , volume: 100, issue: 2, pages: 149-157. Doi: http://dx.doi.org/10.1016/j.trstmh.2005.06.029.	Senegal	Cross-sectional	TF	TI, TF and TI	no	no	no	no	Checklist for Prevalence Studies	Yes	Yes	Yes	Unclear	Unclear	Yes	Yes	Yes	Unclear											[6/9] (67%)	NA	67%
Felton, JMStudies of human immune responses to various antigenic proteins of Chlamydia trachomatis.. 2003. . , volume: , issue: , pages: . Doi: .	Gambia	Case-control	TF	TI	no	no	no	no	Checklist for DTA studies										Unclear	No	Yes	No	Yes	Yes	No	Yes	Yes	Yes	NA	[6/10] (60%)	60%
Feng, L.; Lu, X.; Yu, Y.; Wang, T.; Luo, S.; Sun, Z.; Duan, Q.; Wang, N.; Song, L.Survey, culture, and genome analysis of ocular Chlamydia trachomatis in Tibetan Boarding Primary Schools in Qinghai Province, China. 2017. Frontiers in Cellular and Infection Microbiology. , volume: 6, issue: JAN, pages: . Doi: 10.3389/fcimb.2016.00207.	China	Cross-sectional	TF	TI, <5 follicles	no	no	no	no	Checklist for Prevalence Studies	Yes	Unclear	Unclear	No	Unclear	Yes	Yes	Yes	Unclear											[4/9] (44%)	NA	44%
Ferede, A. T.; Dadi, A. F.; Tariku, A.; Adane, A. A.Prevalence and determinants of active trachoma among preschool-aged children in Dembia District, Northwest Ethiopia. 2017. Infectious Diseases of Poverty. , volume: 6(1) (no pagination), issue: , pages: . Doi: https://dx.doi.org/10.1186/s40249-017-0345-8.	Ethiopia	Cross-sectional	TF	TI	no	no	no	no	Checklist for Prevalence Studies	Yes	Yes	Yes	No	Yes	Yes	Yes	Yes	Yes											[8/9] (89%)	NA	89%
Freitas, H. S. A.; Medina, N. H.; Lopes, M. F. C.; Soares, O. E.; Teodoro, M. T. C.; Ramalho, K. R. B.; Caligaris, L. S. A.; Morschbacher, R.; de Menezes, M. N. C.; Luna, E. J. A.Trachoma in Indigenous Settlements in Brazil, 2000-2008. 2016. Ophthalmic Epidemiology. , volume: 23(6), issue: , pages: 354-359. Doi: https://dx.doi.org/10.3109/09286586.2015.1131305.	Brazil	Cross-sectional	TF	TI	no	no	no	no	Checklist for Prevalence Studies	Yes	Unclear	Unclear	Yes	Unclear	Yes	Unclear	Unclear	Unclear											[3/9] (33%)	NA	33%
Gelaye, B.; Kumie, A.; Aboset, N.; Berhane, Y.; Williams, M. A.School-based intervention: evaluating the role of water, latrines and hygiene education on trachoma and intestinal parasitic infections in Ethiopia. 2014. Journal of Water, Sanitation and Hygiene for Development. , volume: 4, issue: 1, pages: 120-130. Doi: http://dx.doi.org/10.2166/washdev.2013.060.	Ethiopia	Cross-sectional within longitudinal	TF	TI	no	no	no	no	Checklist for Prevalence Studies	Yes	Yes	Unclear	Yes	Unclear	Yes	Yes	Yes	Unclear											[6/9] (67%)	NA	67%
Genet, A.; Dagnew, Z.; Melkie, G.; Keleb, A.; Motbainor, A.; Mebrat, A.; Leshargie, C. T.Prevalence of active trachoma and its associated factors among 1-9 years of age children from model and non-model kebeles in Dangila district, northwest Ethiopia. 2022. PLoS ONE [Electronic Resource]. , volume: 17(6 June) (no pagination), issue: , pages: . Doi: https://dx.doi.org/10.1371/journal.pone.0268441.	Ethiopia	Cross-sectional	TF	TI	no	no	no	no	Checklist for Prevalence Studies	Yes	Yes	Yes	Yes	Yes	Yes	Yes	Yes	Yes											[9/9] (100%)	NA	100%
Ghasemian, E.; Inic-Kanada, A.; Collingro, A.; Tagini, F.; Stein, E.; Alchalabi, H.; Schuerer, N.; Kese, D.; Babiker, B. E.; Borel, N.; Greub, G.; Barisani-Asenbauer, T.Detection of Chlamydiaceae and Chlamydia-like organisms on the ocular surface of children and adults from a trachoma-endemic region. 2018. Scientific Reports. , volume: 8, issue: 1, pages: 7432. Doi: http://dx.doi.org/10.1038/s41598-018-23887-1.	Sudan	Case-control	TF	no	no	yes	no	no	Checklist for DTA studies										Unclear	No	Yes	Unclear	Yes	Yes	Yes	Unclear	Yes	Yes	NA	[6/10] (60%)	60%

S6. Table of included studies

										Risk of Bias checklist items**																					
reference	Country or countries of data collection	Type of study	TF or FPC	other clinical indicators?	photo-graded TF or FPC?	infection?	serology?	SCR?	Critical Appraisal Tool used**	Prevalence study: Was the sample frame appropriate to address the target pop?	Prevalence study: Were study participants sampled in an appropriate way?	Prevalence study: Was the sample size adequate?	Prevalence study: Were the study subjects and setting described in detail?	Prevalence study: Was the data analysis conducted with sufficient coverage of the identified sample?	Prevalence study: Were valid methods used for the identification of the condition(s)?	Prevalence study: Was the condition measured in a standard, reliable way for all participants?	Prevalence study: Was there appropriate statistical analysis?	Prevalence study: Was the response rate adequate, and if not, was the low response rate managed appropriately?	DTA study: Was a consecutive or random sample of patients enrolled?	DTA study: Was a case-control design avoided?	DTA study: Did the study avoid inappropriate exclusions?	DTA: Were the index test results interpreted without knowledge of the results of the reference standard?	DTA: If a threshold was used, was it pre-specified?	DTA: Is the reference standard likely to correctly classify the target condition?	DTA: Were the reference standard results interpreted without knowledge of the results of the index test?	DTA: Was there an appropriate interval between index test and reference standard?	DTA: Did all patients receive the same reference standard?	DTA: Were all patients included in the analysis?	Prevalence study score [meets criteria/total] (%)	DTA study score [meets criteria/total] (%)	Max score (if both checklists used, higher of 2 scores)
Ghasemian, E.; Inic-Kanada, A.; Collingro, A.; Mejdoubi, L.; Alchalabi, H.; Kese, D.; Elshafie, B. E.; Hammou, J.; Barisani-Asenbauer, T.Comparison of genovars and Chlamydia trachomatis infection loads in ocular samples from children in two distinct cohorts in Sudan and Morocco. 2021. PLoS Neglected Tropical Diseases. , volume: 15, issue: 8, pages: . Doi: http://dx.doi.org/10.1371/journal.pntd.0009655.	Morocco	Case-control	TF	no	no	yes	no	no	Checklist for DTA studies										Unclear	No	Yes	Unclear	Yes	Yes	Yes	Unclear	Yes	Yes	NA	[6/10] (60%)	60%
Goldschmidt, P.; Afghani, T.; Nadeem, M.; Ali-Khan, W.; Chaumeil, C.; De Barbeyrac, B.Clinical and microbiological diagnosis of trachoma in children living in rural areas in the District of Attock, Punjab, Pakistan. 2006. Ophthalmic Epidemiology. , volume: 13(5), issue: , pages: 335-342. Doi: https://dx.doi.org/10.1080/09286580600943796.	Pakistan	Cross-sectional	TF	TI	no	yes	no	no	Checklist for Prevalence Studies	Yes	Unclear	Unclear	Yes	Unclear	Yes	Yes	Yes	Unclear											[5/9] (56%)	NA	56%
Goldschmidt, P.; Benallaoua, D.; Amza, A.; Einterz, E.; Huguet, P.; Poisson, F.; Bilinkai, A. B.; Ismaila, M.; Bensaid, P.; Bella, L.; Chaumeil, C.Clinical and microbiological assessment of trachoma in the Kolofata Health District, Far North Region, Cameroon. 2012. Tropical Medicine and Health. , volume: 40, issue: 1, pages: 45121. Doi: http://dx.doi.org/10.2149/tmh.2011-26.	Cameroon	Cross-sectional	TF	TI	no	yes	no	no	Checklist for Prevalence Studies	Yes	Yes	Yes	Yes	Yes	Yes	Yes	Yes	Yes											[9/9] (100%)	NA	100%
Goldschmidt, P.; Einterz, E.The limits of medical interventions for the elimination of preventable blindness. 2014. Tropical Medicine and Health. , volume: 42, issue: 1, pages: 43-52. Doi: http://dx.doi.org/10.2149/tmh.2013-26.	Cameroon	Cross-sectional	TF	TF and TI	no	no	no	no	Checklist for Prevalence Studies	Yes	Yes	Yes	Yes	Unclear	Yes	Yes	Yes	Unclear											[7/9] (78%)	NA	78%
Golovaty, I.; Jones, L.; Gelaye, B.; Melkie, Tilahun; Habtamu, Belete; Abera, Kumie; Yemane, Berhane; Williams, M. A.Access to water source, latrine facilities and other risk factors of active trachoma in Ankober, Ethiopia. 2009. PLoS ONE [Electronic Resource]. , volume: , issue: August, pages: e6702. Doi: http://dx.doi.org/10.1371/journal.pone.0006702.	Ethiopia	Cross-sectional	TF	TI, TF and/or TI	no	no	no	no	Checklist for Prevalence Studies	Yes	Yes	Yes	Yes	Unclear	Yes	Yes	Yes	Unclear											[7/9] (78%)	NA	78%
Gower, E. W.; Solomon, A. W.; Burton, M. J.; Aguirre, A.; Munoz, B.; Bailey, R.; Holland, M.; Makalo, P.; Massae, P.; Mkocha, H.; Mabey, D. C.; West, S. K.Chlamydial positivity of nasal discharge at baseline is associated with ocular chlamydial positivity 2 months following azithromycin treatment. 2006. Investigative Ophthalmology & Visual Science. , volume: 47, issue: 11, pages: 4767-4771. Doi: .	Gambia, Tanzania	Cross-sectional	TF	TI	no	yes	no	no	Both	Yes	Yes	Yes	No	Yes	Yes	Yes	Yes	Yes	Unclear	Yes	Yes	Unclear	Yes	Yes	Yes	Yes	Yes	Yes	[8/9] (89%)	[8/10] (80%)	89%
Gwyn, S. E.; Xiang, L.; Kandel, R. P.; Dean, D.; Gambhir, M.; Martin, D. L.Prevalence of Chlamydia trachomatis-Specific Antibodies before and after Mass Drug Administration for Trachoma in Community-Wide Surveys of Four Communities in Nepal. 2018. American Journal of Tropical Medicine & Hygiene. , volume: 98, issue: 1, pages: 216-220. Doi: https://dx.doi.org/10.4269/ajtmh.17-0102.	Nepal	Cross-sectional	TF	no	no	no	yes	no	Checklist for Prevalence Studies	Yes	Yes	Yes	Yes	Yes	Yes	Yes	Yes	Unclear											[8/9] (89%)	NA	89%
Gwyn, S.; Mkocha, H.; Randall, J. M.; Kasubi, M.; Martin, D. L.Optimization of a rapid test for antibodies to the Chlamydia trachomatis antigen Pgp3. 2019. Diagnostic Microbiology and Infectious Disease. , volume: 93, issue: 4, pages: 293-298. Doi: http://dx.doi.org/10.1016/j.diagmicrobio.2018.11.001.	Tanzania	Cross-sectional	TF	no	no	yes	yes	no	Checklist for Prevalence Studies	Yes	Yes	Unclear	Yes	Unclear	Yes	Yes	Yes	Unclear											[6/9] (67%)	NA	67%
Gwyn, S.; Awoussi, M. S.; Bakhtiari, A.; Bronzan, R. N.; Crowley, K.; Harding-Esch, E. M.; Kassankogno, Y.; Kilangalanga, J. N.; Makangila, F.; Mupoyi, S.; Ngondi, J.; Ngoyi, B.; Palmer, S.; Randall, J. M.; Seim, A.; Solomon, A. W.; Stewart, R.; Togbey, K.; Uvon, P. A.; Martin, D. L.Comparison of platforms for testing antibodies to Chlamydia trachomatis antigens in the Democratic Republic of the Congo and Togo. 2021. Scientific Reports. , volume: 11, issue: 3, pages: . Doi: http://dx.doi.org/10.1038/s41598-021-86639-8.	Democratic Republic of the Congo, Togo	Cross-sectional	TF	no	no	no	yes	yes	Checklist for Prevalence Studies	Yes	Yes	Unclear	Yes	Yes	Yes	Yes	Yes	Yes											[8/9] (89%)	NA	89%
Gwyn, S.; Nute, A. W.; Eshetu, Sata; Zerihun, Tadesse; Ambahun, Chernet; Mahteme, Haile; Taye, Zeru; Bethea, D.; Laurent, C.; Callahan, E. K.; Nash, S. D.; Martin, D. L.The performance of immunoassays to measure antibodies to the Chlamydia trachomatis antigen Pgp3 in different epidemiological settings for trachoma. 2021. American Journal of Tropical Medicine and Hygiene. , volume: 105, issue: 5, pages: 1362-1367. Doi: http://dx.doi.org/10.4269/ajtmh.21-0541.	Ethiopia	Cross-sectional	TF	TI	no	yes	yes	yes	Checklist for Prevalence Studies	Yes	Yes	Unclear	Yes	Unclear	Yes	Yes	Yes	Unclear											[6/9] (67%)	NA	67%
Gyasi, M. E.; Nsiire, A.; Yayemain, D.; Debrah, O. A.; Asamani, D.; Gyapong, J.; Amoaku, W. M. K.Trachoma in northern Ghana: a need for further studies. 2010. Ophthalmic Epidemiology. , volume: 17, issue: 6, pages: 343-348. Doi: http://dx.doi.org/10.3109/09286586.2010.528132.	Ghana	Cross-sectional	TF	TI	no	no	no	no	Checklist for Prevalence Studies	Yes	Yes	Yes	No	Yes	Yes	Yes	Yes	Yes											[8/9] (89%)	NA	89%
Hagan, M.; Yayemain, D.; Ahorsu, F.; Aboe, A.Prevalence of active trachoma two years after control activities. 2009. Ghana Medical Journal. , volume: 43, issue: 2, pages: 54-60. Doi: .	Ghana	Cross-sectional	2 or more follicles	TF and TI	no	no	no	no	Checklist for Prevalence Studies	Yes	Yes	Unclear	No	Unclear	No, 2 follicles counted as TF	Yes	Yes	Unclear											[4/9] (44%)	NA	44%
Tesfaye Haileselassie, Adera; Macleod, C.; Misganu, Endriyas; Dejene, M.; Willis, R.; Chu, B. K.; Yohannes, Letamo; Tebeje, Misganaw; Tamiru, Mesele; Emebet, Mekonnen; Alemayehu, Sisay; Yeneneh, Mulugeta; Wondu, Alemayehu; Khumbo, Kalua; Tezera Kifle, Destu; Adamu, Y.; Smith, J. L.; Abu, Beyene; Tadesse, A.; Solomon, A. W. Prevalence of and risk factors for trachoma in Southern Nations, Nationalities, and Peoples' Region, Ethiopia: results of 40 population-based prevalence surveys carried out with the Global Trachoma Mapping Project. 2016. Special Issue: Trachoma mapping. , volume: 23, issue: Suppl. 1, pages: 84-93. Doi: .	Ethiopia	Cross-sectional	TF	TI, TF and/or TI	no	no	no	no	Checklist for Prevalence Studies	Yes	Yes	Yes	Yes	Unclear	Yes	Yes	Yes	Unclear											[7/9] (78%)	NA	78%
Hammou, J.; Guagliardo, S. A. J.; Obtel, M.; Razine, R.; Haroun, A. E.; Youbi, M.; Belfequih, A. M.; White, M.; Gwyn, S.; Martin, D. L.Post-Validation Survey in Two Districts of Morocco after the Elimination of Trachoma as a Public Health Problem. 2022. American Journal of Tropical Medicine & Hygiene. , volume: 28, issue: , pages: 28. Doi: https://dx.doi.org/10.4269/ajtmh.21-1140.	Morocco	Cross-sectional	TF	TI, TF and/or TI	no	no	yes	yes	Checklist for Prevalence Studies	Yes	Yes	Yes	Yes	Yes	Yes	Yes	Yes	Yes											[9/9] (100%)	NA	100%
Harding-Esch, E. M.; Edwards, T.; Sillah, A.; Sarr-Sissoho, I.; Aryee, E. A.; Snell, P.; Holland, M. J.; Mabey, D. C.; Bailey, R. L.Risk factors for active trachoma in The Gambia. 2008. Transactions of the Royal Society of Tropical Medicine and Hygiene. , volume: 102, issue: 12, pages: 1255-1262. Doi: http://dx.doi.org/10.1016/j.trstmh.2008.04.022.	Gambia	Cross-sectional	TF	TI, TF and/or TI	no	yes	no	no	Checklist for Prevalence Studies	Yes	Yes	Yes	Yes	Unclear	Yes	Yes	Yes	Unclear											[7/9] (78%)	NA	78%
Harding-Esch, E. M.; Edwards, T.; Sillah, A.; Sarr, I.; Roberts, C. H.; Snell, P.; Aryee, E.; Molina, S.; Holland, M. J.; Mabey, D. C. W.; Bailey, R. L.Active trachoma and ocular Chlamydia trachomatis infection in two Gambian regions: on course for elimination by 2020?. 2009. PLoS Neglected Tropical Diseases. , volume: 3, issue: 12, pages: e573. Doi: http://dx.doi.org/10.1371/journal.pntd.0000573.	Gambia	Cross-sectional	TF	TI	no	no	no	no	Checklist for Prevalence Studies	Yes	Yes	Yes	Yes	Unclear	Yes	Yes	Yes	Unclear											[7/9] (78%)	NA	78%
Harding-Esch, E. M.; Edwards, T.; Mkocha, H.; Munoz, B.; Holland, M. J.; Burr, S. E.; Sillah, A.; Gaydos, C. A.; Stare, D.; Mabey, D. C. W.; Bailey, R. L.; West, S. K.Trachoma prevalence and associated risk factors in The Gambia and Tanzania: baseline results of a cluster randomised controlled trial. 2010. PLoS Neglected Tropical Diseases. , volume: 4, issue: 11, pages: e861. Doi: http://dx.doi.org/10.1371/journal.pntd.0000861.	Gambia	Cross-sectional	TF	TI, TF and TI	no	yes	no	no	Both	Yes	Yes	Unclear	Yes	Unclear	Yes	Yes	Yes	Unclear	Random	Yes	Yes	Unclear	Yes	Yes	Yes	Yes	Yes	Yes	[6/9] (67%)	[8/10] (80%)	80%
Harding-Esch, E. M.; Sillah, A.; Edwards, T.; Burr, S. E.; Hart, J. D.; Joof, H.; Laye, M.; Makalo, P.; Manjang, A.; Molina, S.; Sarr-Sissoho, I.; Quinn, T. C.; Lietman, T.; Holland, M. J.; Mabey, D.; West, S. K.; Bailey, R.Mass treatment with azithromycin for trachoma: when is one round enough? Results from the PRET Trial in The Gambia. 2013. PLoS Neglected Tropical Diseases. , volume: 7, issue: 6, pages: e2115. Doi: http://dx.doi.org/10.1371/journal.pntd.0002115.	Gambia	Cross-sectional within longitudinal	TF	no	no	yes	no	no	Checklist for Prevalence Studies	Yes	Yes	Yes	Yes	Yes	Yes	Yes	Yes	Yes											[9/9] (100%)	NA	100%
Harding-Esch, E. M.; Kadimpeul, J.; Sarr, B.; Sane, A.; Badji, S.; Laye, M.; Sillah, A.; Burr, S. E.; MacLeod, D.; Last, A. R.; Holland, M. J.; Mabey, D. C.; Bailey, R. L.Population-based prevalence survey of follicular trachoma and trachomatous trichiasis in the Casamance region of Senegal. 2017. BMC Public Health. , volume: 18, issue: 1, pages: 62. Doi: https://dx.doi.org/10.1186/s12889-017-4605-0.	Senegal	Cross-sectional	TF	TI	no	no	no	no	Checklist for Prevalence Studies	Yes	Yes	Yes	Yes	Yes	Yes	Yes	Yes	Yes											[9/9] (100%)	NA	100%
Harding-Esch, E. M.; Holland, M. J.; Schemann, J. F.; Sillah, A.; Sarr, B.; Christerson, L.; Pickering, H.; Molina-Gonzalez, S.; Sarr, I.; Andreasen, A. A.; Jeffries, D.; Grundy, C.; Mabey, D. C. W.; Herrmann, B.; Bailey, R. L.Impact of a single round of mass drug administration with azithromycin on active trachoma and ocular Chlamydia trachomatis prevalence and circulating strains in The Gambia and Senegal. 2019. Parasites and Vectors. , volume: 12, issue: 497, pages: . Doi: .	Senegal	Cross-sectional	TF	TI	no	yes	no	no	Checklist for Prevalence Studies	Yes	Yes	Yes	No	Yes	Yes	Yes	Yes	Yes											[8/9] (89%)	NA	89%
Hudson, A. P.; McEntee, C. M.; Reacher, M.; Whittum-Hudson, J. A.; Taylor, H. R.Inapparent ocular infection by Chlamydia trachomatis in experimental and human trachoma. 1992. Current Eye Research. , volume: 11, issue: 3, pages: 279-83. Doi: .	unknown	Cross-sectional	TF	TI	no	yes	no	no	Checklist for DTA studies										Unclear	Unclear	Unclear	Unclear	Yes	Yes	Unclear	Yes	Yes	Unclear	NA	[4/10] (40%)	40%
Huguet, P.; Bella, L.; Einterz, E. M.; Goldschmidt, P.; Bensaid, P.Mass treatment of trachoma with azithromycin 1.5% eye drops in the Republic of Cameroon: feasibility, tolerance and effectiveness. 2010. British Journal of Ophthalmology. , volume: 94, issue: 2, pages: 157-160. Doi: http://dx.doi.org/10.1136/bjo.2009.161513.	Cameroon	Cross-sectional	TF	TF and/or TI, TF and TI	no	no	no	no	Checklist for Prevalence Studies	Yes	Yes	Yes	Yes	Yes	Yes	Yes	Yes	Yes											[9/9] (100%)	NA	100%
Jenson, A.; Dize, L.; Mkocha, H.; Munoz, B.; Lee, J.; Gaydos, C.; Quinn, T.; West, S. K.Field evaluation of the cepheid GeneXpert Chlamydia trachomatis assay for detection of infection in a trachoma endemic community in Tanzania. 2013. PLoS Neglected Tropical Diseases. , volume: 7, issue: 7, pages: e2265. Doi: http://dx.doi.org/10.1371/journal.pntd.0002265.	Tanzania	Cross-sectional	TF	TI, TF and TI	no	yes	no	no	Checklist for DTA studies										Unclear	Yes	Yes	Unclear	Yes	Yes	Yes	Yes	Yes	Yes	NA	[8/10] (80%)	80%

S6. Table of included studies

										Risk of Bias checklist items**																					
reference	Country or countries of data collection	Type of study	TF or FPC	other clinical indicators?	photo-graded TF or FPC?	infection?	serology?	SCR?	Critical Appraisal Tool used**	Prevalence study: Was the sample frame appropriate to address the target pop?	Prevalence study: Were study participants sampled in an appropriate way?	Prevalence study: Was the sample size adequate?	Prevalence study: Were the study subjects and setting described in detail?	Prevalence study: Was the data analysis conducted with sufficient coverage of the identified sample?	Prevalence study: Were valid methods used for the identification of the condition(s)?	Prevalence study: Was the condition measured in a standard, reliable way for all participants?	Prevalence study: Was there appropriate statistical analysis?	Prevalence study: Was the response rate adequate, and if not, was the low response rate managed appropriately?	DTA study: Was a consecutive or random sample of patients enrolled?	DTA study: Was a case-control design avoided?	DTA study: Did the study avoid inappropriate exclusions?	DTA: Were the index test results interpreted without knowledge of the results of the reference standard?	DTA: If a threshold was used, was it pre-specified?	DTA: Is the reference standard likely to correctly classify the target condition?	DTA: Were the reference standard results interpreted without knowledge of the results of the index test?	DTA: Was there an appropriate interval between index test and reference standard?	DTA: Did all patients receive the same reference standard?	DTA: Were all patients included in the analysis?	Prevalence study score [meets criteria/total] (%)	DTA study score [meets criteria/total] (%)	Max score (if both checklists used, higher of 2 scores)
Jip, N. F.; King, J. D.; Diallo, M. O.; Miri, E. S.; Hamza, A. T.; Ngondi, J.; Emerson, P. M.Blinding trachoma in Katsina State, Nigeria: population-based prevalence survey in ten local government areas. 2008. Ophthalmic Epidemiology. , volume: 15, issue: 5, pages: 294-302. Doi: http://dx.doi.org/10.1080/09286580802256542.	Nigeria	Cross-sectional	TF	TI, TF and/or TI	no	no	no	no	Checklist for Prevalence Studies	Yes	Yes	Yes	Yes	Unclear	Yes	Yes	Yes	Unclear											[7/9] (78%)	NA	78%
Kabona, G.; Ngondi, J.; Kirumbi, E.; Kamugisha, M.; Kabole, I.; Simon, A.; Massae, P.; Gower, L.; Nanai, A.; Deogratius, D.; Mangasini, E.; Mmbando, D.; Aboe, A.; Mwingira, U. J.Evaluation of four years implementation of the safe strategy (surgery, antibiotics, facial cleanliness and environmental improvement) for trachoma control in handeni district, tanzania. 2013. American Journal of Tropical Medicine and Hygiene. , volume: 1), issue: , pages: 45210. Doi: .	Tanzania	Cross-sectional	TF	TI	no	no	no	no	Checklist for Prevalence Studies	Yes	Yes	Unclear	No	Unclear	Unclear	Yes	Unclear	Unclear											[3/9] (33%)	NA	33%
Kalua, K.; Chirwa, T.; Kalilani, L.; Abbenyi, S.; Mukaka, M.; Bailey, R.Prevalence and risk factors for trachoma in central and southern Malawi. 2010. PLoS ONE [Electronic Resource]. , volume: , issue: February, pages: e9067. Doi: http://dx.doi.org/10.1371/journal.pone.0009067.	Malawi	Cross-sectional	TF	TI	no	no	no	no	Checklist for Prevalence Studies	Yes	Yes	Yes	Yes	Yes	Yes	Yes	Yes	Yes											[9/9] (100%)	NA	100%
Kalua, K.; Singini, I.; Mukaka, M.; Msyamboza, K.; Masika, M.; Bailey, R.Scaling up of trachoma mapping in Salima District, Central Malawi. 2014. Health. , volume: 6, issue: 1, pages: 57-63. Doi: http://dx.doi.org/10.4236/health.2014.61009.	Malawi	Cross-sectional	TF	TI	no	no	no	no	Checklist for Prevalence Studies	Yes	Yes	Yes	Yes	Yes	Yes	Yes	Yes	Yes											[9/9] (100%)	NA	100%
Kashaf, M. S.; Munoz, B. E.; Mkocha, H.; Wolle, M. A.; Naufal, F.; West, S. K.Incidence and progression of trachomatous scarring in a cohort of children in a formerly hyper-endemic district of Tanzania. 2020. PLoS Neglected Tropical Diseases. , volume: 14, issue: 10, pages: . Doi: http://dx.doi.org/10.1371/journal.pntd.0008708.	Tanzania	Cross-sectional within longitudinal	TF	no	no	yes	no	no	Checklist for Prevalence Studies	Yes	Yes	Yes	No	Unclear	Yes	Yes	Yes	Unclear											[6/9] (67%)	NA	67%
Kassim, K.; Kassim, J.; Aman, R.; Abduku, M.; Tegegne, M.; Sahiledengle, B.Prevalence of active trachoma and associated risk factors among children of the pastoralist population in Madda Walabu rural district, Southeast Ethiopia: A community-based cross-sectional study. 2019. BMC Infectious Diseases. , volume: 19(1) (no pagination), issue: , pages: . Doi: https://dx.doi.org/10.1186/s12879-019-3992-5.	Ethiopia	Cross-sectional	TF	TI, TF and/or TI, TF and TI	no	no	no	no	Checklist for Prevalence Studies	Yes	Yes	Yes	Yes	Unclear	Yes	Yes	Yes	Unclear											[7/9] (78%)	NA	78%
Kasubi, M.; Kabona, G.; Simon, A.; Kalabashanga, J.; Harran, M.; French, M.; Mwingira, U.; West, S.; Martin, D.; Ngondi, J.Alternative indicators to monitor trachoma elimination; does longitudinal chlamydia infection and antibody data add evidence to the understanding of resurgence. 2020. American Journal of Tropical Medicine and Hygiene. , volume: 103(5 SUPPL), issue: , pages: 365. Doi: .	Tanzania	Cross-sectional	TF	no	no	yes	yes	no	Checklist for Prevalence Studies	Yes	Unclear	Unclear	Yes	Unclear	Yes	Yes	Unclear	Unclear											[4/9] (44%)	NA	44%
Katz, J.; West Jr, K. P.; Khatry, S. K.; LeClerq, S. C.; Pradhan, E. K.; Thapa, M. D.; Shrestha, S. R.; Taylor, H. R.Prevalence and risk factors for trachoma in Sarlahi district, Nepal. 1996. British Journal of Ophthalmology. , volume: 80(12), issue: , pages: 1037-1041. Doi: http://dx.doi.org/10.1136/bjo.80.12.1037.	Nepal	Cross-sectional	TF	TI	no	no	no	no	Checklist for Prevalence Studies	Yes	Yes	Unclear	No	Yes	Yes	Yes	Yes	Yes											[7/9] (78%)	NA	78%
Kedir, S.; Lemnuro, K.; Yesse, M.; Abdella, B.; Muze, M.; Mustefa, A.; Musema, M.; Hussen, L.Prevalence and factors associated with active trachoma among children 1-9 years of age in the catchment population of tora primary hospital, silte zone, southern ethiopia, 2020. 2021. Open Ophthalmology Journal. , volume: 15, issue: 1, pages: 108-116. Doi: 10.2174/1874364102115010108.	Ethiopia	Cross-sectional	TF	TI, TF and/or TI, TF and TI	no	no	no	no	Checklist for Prevalence Studies	Yes	Yes	Yes	Yes	Yes	Yes	Yes	Yes	Yes											[9/9] (100%)	NA	100%
Keenan, J. D.; See, C. W.; Moncada, J.; Ayele, B.; Gebre, T.; Stoller, N. E.; McCulloch, C. E.; Porco, T. C.; Gaynor, B. D.; Emerson, P. M.; Schachter, J.; Lietman, T. M.Diagnostic characteristics of tests for ocular Chlamydia after mass azithromycin distributions. 2012. Investigative Ophthalmology & Visual Science. , volume: 53, issue: 1, pages: 235-40. Doi: https://dx.doi.org/10.1167/iovs.11-8493.	Ethiopia	Cross-sectional within RCT	TF	TI, TF and/or TI	no	yes	no	no	Both	Yes	Yes	Yes	No	Unclear	Yes	Yes	Yes	Unclear	Random	Yes	Yes	Unclear	Yes	Yes	Yes	Yes	Yes	Yes	[6/9] (67%)	[8/10] (80%)	80%
Keenan, J. D.; Tadesse, Z.; Gebresillasie, S.; Shiferaw, A.; Zerihun, M.; Emerson, P. M.; Callahan, K.; Cotter, S. Y.; Stoller, N. E.; Porco, T. C.; Oldenburg, C. E.; Lietman, T. M.Mass azithromycin distribution for hyperendemic trachoma following a cluster-randomized trial: A continuation study of randomly reassigned subclusters (TANA II). 2018. PLoS Medicine / Public Library of Science. , volume: 15, issue: 8, pages: e1002633. Doi: https://dx.doi.org/10.1371/journal.pmed.1002633.	Ethiopia	Cross-sectional within RCT	TF	no	no	yes	no	no	Checklist for Prevalence Studies	Yes	Yes	Yes	Yes	Unclear	Yes	Yes	Yes	Unclear											[7/9] (78%)	NA	78%
Ketema, K.; Tiruneh, M.; Woldeyohannes, D.; Muluye, D.Active trachoma and associated risk factors among children in Baso Liben District of East Gojjam, Ethiopia. 2012. BMC Public Health. , volume: 12, issue: , pages: 1105. Doi: .	Ethiopia	Cross-sectional	TF	TI, TF and/or TI	no	no	no	no	Checklist for Prevalence Studies	Yes	Yes	Unclear	Yes	Unclear	Yes	Yes	Yes	Unclear											[6/9] (67%)	NA	67%
Khandekar, R.; Mohammed, A. J.Outcome of azithromycin treatment of active trachoma in Omani schoolchildren. 2003. Eastern Mediterranean Health Journal. , volume: 9, issue: 45052, pages: 1026-1033. Doi: .	Oman	Case-control	TF	TI	no	no	no	no	Checklist for Prevalence Studies	Yes	Yes	Yes	Yes	Unclear	Yes	Yes	Yes	Unclear											[7/9] (78%)	NA	78%
Khandekar, R.; Mabry, R.; Al-Hadrami, K.; Sarvanan, N.Active trachoma, face washing (F) and environmental improvement (E) in a high-risk population in Oman. 2005. Eastern Mediterranean Health Journal. , volume: 11, issue: 3, pages: 402-409. Doi: .	Oman	Cross-sectional	TF	TI	no	no	no	no	Checklist for Prevalence Studies	No	No	Unclear	Yes	Yes	Yes	Yes	Yes	Yes											[6/9] (67%)	NA	67%
Sumeet, Khanduja; Vishal, Jhanji; Namrata, Sharma; Praveen, Vashist; Murthy, G. V. S.; Sanjeev, Gupta; Gita, Satpathy; Radhika, Tandon; Titiyal, J. S.; Vajpayee, R. B.Rapid assessment of trachoma among children living in rural Northern India. 2009. Ophthalmic Epidemiology. , volume: 16, issue: 4, pages: 206-211. Doi: http://dx.doi.org/10.1080/09286580902999389.	India	Cross-sectional	TF	TI, TF and/or TI	no	yes	no	no	Checklist for Prevalence Studies	No, using TRA methods, sampled most-likely to be positive areas	Yes	Yes	Yes	Unclear	Yes	Yes	Yes	Unclear											[6/9] (67%)	NA	67%
Kim, J. S.; Oldenburg, C. E.; Cooley, G.; Amza, A.; Kadri, B.; Nassirou, B.; Cotter, S. Y.; Stoller, N. E.; West, S. K.; Bailey, R. L.; Keenan, J. D.; Gaynor, B. D.; Porco, T. C.; Lietman, T. M.; Martin, D. L.Community-level chlamydial serology for assessing trachoma elimination in trachoma-endemic Niger. 2019. PLoS Neglected Tropical Diseases. , volume: 13, issue: 1, pages: e0007127. Doi: http://dx.doi.org/10.1371/journal.pntd.0007127.	Niger	Cross-sectional	TF	TI	no	no	yes	no	Both	Yes	Yes	Unclear	Yes	Unclear	Yes	Yes	Yes	Unclear	random	Yes	Yes	Unclear	Yes	Yes	Yes	Yes	Yes	Yes	[6/9] (67%)	[8/10] (80%)	80%
King, J. D.; Ngondi, J.; Gatpan, G.; Lopidia, B.; Becknell, S.; Emerson, P. M.The burden of trachoma in Ayod County of Southern Sudan. 2008. PLoS Neglected Tropical Diseases. , volume: 2, issue: 9, pages: e299. Doi: http://dx.doi.org/10.1371/journal.pntd.0000299.	South Sudan	Cross-sectional	TF	TI, TF and/or TI	no	no	no	no	Checklist for Prevalence Studies	Yes	Yes	Yes	Yes	Yes	Yes	Yes	Yes	Yes											[9/9] (100%)	NA	100%
King, J. D.; Jip, N.; Jugu, Y. S.; Othman, A.; Rodgers, A. F.; Dajom, D. Y.; Miri, E.; Emerson, P. M.Mapping trachoma in Nasarawa and Plateau States, central Nigeria. 2010. British Journal of Ophthalmology. , volume: 94, issue: 1, pages: 14-19. Doi: http://dx.doi.org/10.1136/bjo.2009.165282.	Nigeria	Cross-sectional	TF	TI, TF and/or TI	no	no	no	no	Checklist for Prevalence Studies	Yes	Yes	Yes	Yes	Yes	Yes	Yes	Yes	Yes											[9/9] (100%)	NA	100%
King, J. D.; Odermatt, P.; Utzinger, J.; Ngondi, J.; Bamani, S.; Kamissoko, Y.; Boubicar, K.; Hassan, A. S.; Nwobi, B. C.; Jip, N.; Amnie, A.; Teferi, T.; Mosher, A. W.; Stewart, A. E. P.; Cromwell, E. A.; Emerson, P. M.Trachoma among children in community surveys from four African countries and implications of using school surveys for evaluating prevalence. 2013. International Health (RSTMH). , volume: 5, issue: 4, pages: 280-287. Doi: http://dx.doi.org/10.1093/inthealth/iht027.	Ethiopia, Mali, Niger, Nigeria	Cross-sectional	TF	TI	no	no	no	no	Checklist for Prevalence Studies	Yes	Yes	Unclear	Yes	Unclear	Yes	Yes	Yes	Unclear											[6/9] (67%)	NA	67%
King, J. D.; Teferi, T.; Cromwell, E. A.; Zerihun, M.; Ngondi, J. M.; Damte, M.; Ayalew, F.; Tadesse, Z.; Gebre, T.; Mulualem, A.; Karie, A.; Melak, B.; Adugna, M.; Gessesse, D.; Worku, A.; Endashaw, T.; Ayele, F. A.; Stoller, N. E.; King, M. R. A.; Mosher, A. W.; Gebregzabher, T.; Haileysus, G.; Odermatt, P.; Utzinger, J.; Emerson, P. M.Prevalence of trachoma at sub-district level in Ethiopia: determining when to stop mass azithromycin distribution. 2014. PLoS Neglected Tropical Diseases. , volume: 8, issue: 3, pages: e2732. Doi: .	Ethiopia	Cross-sectional	TF	TI	no	no	no	no	Checklist for Prevalence Studies	Yes	Yes	Yes	Yes	Yes	Yes	Yes	Yes	Yes											[9/9] (100%)	NA	100%
Koroma, J. B.; Heck, E.; Vandy, M.; Sonnie, M.; Hodges, M.; MacArthur, C.; Sankara, D. P.The epidemiology of trachoma in the five northern districts of Sierra Leone. 2011. Ophthalmic Epidemiology. , volume: 18, issue: 4, pages: 150-157. Doi: http://dx.doi.org/10.3109/09286586.2011.594204.	Sierra Leone	Cross-sectional	TF	TF and/or TI	no	no	no	no	Checklist for Prevalence Studies	Yes	Yes	Yes	Yes	Yes	Yes	Yes	Yes	Yes											[9/9] (100%)	NA	100%
Koukounari, A.; Moustaki, I.; Blake, I. M.; Donnelly, C. A.; Alexander, N. D. E.; Harding-Esch, E. M.; Holland, M. J.; Basanez, M. G.; Manoj, G.; Mabey, D. C.; Bailey, R.; Grassly, N. C.; Solomon, A. W.Evaluation of diagnostics of chlamydia trachomatis infection in Kahe Mpya subvillage, Rombo District, Tanzania: A latent variable modeling approach. 2011. American Journal of Tropical Medicine and Hygiene. , volume: 1), issue: , pages: 168. Doi: .	Tanzania	Cross-sectional	TF	TI	no	yes	no	no	Checklist for Prevalence Studies	Unclear	Unclear	Unclear	No	Unclear	Unclear	Unclear	Unclear	Unclear											[0/9] (0%)	NA	0%
Kur, L. W.; Picon, D.; Adibo, O.; Robinson, E.; Sabasio, A.; Edwards, T.; Ndyaba, A.; Rumunu, J.; Lewis, K.; Lado, M.; Kolaczinski, J.Trachoma in Western Equatoria State, Southern Sudan: implications for national control. 2009. PLoS Neglected Tropical Diseases. , volume: 3, issue: 7, pages: e492. Doi: http://dx.doi.org/10.1371/journal.pntd.0000492.	South Sudan	Cross-sectional	TF	TI	no	no	no	no	Checklist for Prevalence Studies	Yes	Yes	Yes	Yes	Yes	Yes	Yes	Yes	Yes											[9/9] (100%)	NA	100%
Laming, A. C.; Hallsworth, P. J.Detection of chlamydial nucleic acid in follicular trachoma. 1999. Medical Journal of Australia. , volume: 170, issue: 4, pages: 190. Doi: .	Australia	Cross-sectional	TF	no	no	yes	no	no	Checklist for DTA studies										Unclear	Unclear	Unclear	Unclear	Unclear	Unclear	Yes	Unclear	Unclear	Unclear	NA	[1/10] (10%)	10%

S6. Table of included studies

										Risk of Bias checklist items**																					
reference	Country or countries of data collection	Type of study	TF or FPC	other clinical indicators?	photo-graded TF or FPC?	infection?	serology?	SCR?	Critical Appraisal Tool used**	Prevalence study: Was the sample frame appropriate to address the target pop?	Prevalence study: Were study participants sampled in an appropriate way?	Prevalence study: Was the sample size adequate?	Prevalence study: Were the study subjects and setting described in detail?	Prevalence study: Was the data analysis conducted with sufficient coverage of the identified sample?	Prevalence study: Were valid methods used for the identification of the condition(s)?	Prevalence study: Was the condition measured in a standard, reliable way for all participants?	Prevalence study: Was there appropriate statistical analysis?	Prevalence study: Was the response rate adequate, and if not, was the low response rate managed appropriately?	DTA study: Was a consecutive or random sample of patients enrolled?	DTA study: Was a case-control design avoided?	DTA study: Did the study avoid inappropriate exclusions?	DTA: Were the index test results interpreted without knowledge of the results of the reference standard?	DTA: If a threshold was used, was it pre-specified?	DTA: Is the reference standard likely to correctly classify the target condition?	DTA: Were the reference standard results interpreted without knowledge of the results of the index test?	DTA: Was there an appropriate interval between index test and reference standard?	DTA: Did all patients receive the same reference standard?	DTA: Were all patients included in the analysis?	Prevalence study score [meets criteria/total] (%)	DTA study score [meets criteria/total] (%)	Max score (if both checklists used, higher of 2 scores)
Last, A. R.; Burr, S. E.; Weiss, H. A.; Harding-Esch, E. M.; Cassama, E.; Nabicassa, M.; Mabey, D. C.; Holland, M. J.; Bailey, R. L.Risk factors for active trachoma and ocular Chlamydia trachomatis infection in treatment-naive trachoma-hyperendemic communities of the Bijagos Archipelago, Guinea Bissau. 2014. PLoS Neglected Tropical Diseases. , volume: 8, issue: 6, pages: e2900. Doi: http://dx.doi.org/10.1371/journal.pntd.0002900.	Guinea Bissau	Cross-sectional	TF	TI, TF and/or TI	no	yes	no	no	Checklist for Prevalence Studies	Yes	Yes	Yes	Yes	Unclear	Yes	Yes	Yes	Unclear											[7/9] (78%)	NA	78%
Last, A.; Cassama, E.; Bojang, E.; Nabicassa, M.; Burr, S.; Thompson, K.; Haddad, D.; Mabey, D.; Holland, M.; Bailey, R.Trachoma elimination in an endemic island setting in West Africa: Are two doses of oral azithromycin better than one?. 2015. American Journal of Tropical Medicine and Hygiene. , volume: 93(4 Supplement), issue: , pages: 403-404. Doi: .	Guinea Bissau	Cross-sectional within RCT	TF	no	no	yes	no	no	Checklist for Prevalence Studies	Unclear	Unclear	Unclear	No	Unclear	Unclear	Yes	Yes	Unclear											[2/9] (22%)	NA	22%
Last, A. R.; Burr, S. E.; Harding-Esch, E.; Cassama, E.; Nabicassa, M.; Roberts, C. H.; Mabey, D. C. W.; Holland, M. J.; Bailey, R. L.The impact of a single round of community mass treatment with azithromycin on disease severity and ocular Chlamydia trachomatis load in treatment-naive trachoma-endemic island communities in West Africa. 2017. Parasites and Vectors. , volume: 10, issue: 624, pages: . Doi: http://dx.doi.org/10.1186/s13071-017-2566-x.	Guinea Bissau	Cross-sectional	TF	TI	no	yes	no	no	Checklist for Prevalence Studies	Yes	Yes	Yes	No	Yes	Yes	Yes	Yes	Yes											[8/9] (89%)	NA	89%
Lee, J.; Munoz, B.; Mkocha, H.; Gaydos, C.; West, S.Decline of ocular Chlamydia trachomatis infection and follicular trachoma with annual rounds of mass treatment in mesoendemic communities in Tanzania. 2013. Investigative Ophthalmology and Visual Science. Conference. , volume: 54, issue: 15, pages: . Doi: .	Tanzania	Cross-sectional	TF	no	no	yes	no	no	Checklist for Prevalence Studies	Unclear	Unclear	Unclear	No	Unclear	Yes	Yes	Yes	Unclear											[3/9] (33%)	NA	33%
Lee, J. S.; Munoz, B. E.; Mkocha, H.; Gaydos, C. A.; Quinn, T. C.; West, S. K.The effect of multiple rounds of mass drug administration on the association between ocular Chlamydia trachomatis infection and follicular trachoma in preschool-aged children. 2014. PLoS Neglected Tropical Diseases. , volume: 8, issue: 4, pages: e2761. Doi: http://dx.doi.org/10.1371/journal.pntd.0002761.	Tanzania	Cross-sectional	TF	TI	no	yes	no	no	Checklist for Prevalence Studies	Yes	Yes	Unclear	No	Unclear	Yes	Yes	Yes	Unclear											[5/9] (56%)	NA	56%
Liang, QingFeng; Lu, XinXin; Wang, Mei; Tian, Lei; Labbe, A.; Hu, AiLianStudy of infectious conjunctivitis among children in rural areas of Qinghai province. 2016. Special Issue: Etiology and prevention of trachoma.. , volume: 59, issue: 6, pages: 548-554. Doi: http://dx.doi.org/10.1007/s11427-016-5058-x.	China	Cross-sectional	TF	TI	no	yes	no	no	Checklist for Prevalence Studies	Unclear	Yes	Yes	Yes	Yes	Yes	Yes	Yes	Yes											[8/9] (89%)	NA	89%
Liu, F. C.; Porco, T. C.; Amza, A.; Kadri, B.; Nassirou, B.; West, S. K.; Bailey, R. L.; Keenan, J. D.; Lietman, T. M.Short-term forecasting of the prevalence of clinical trachoma: utility of including delayed recovery and tests for infection. 2015. Parasites and Vectors. , volume: 8, issue: 535, pages: . Doi: .	Niger	Cross-sectional within longitudinal	TF	TI	no	yes	no	no	Checklist for Prevalence Studies	Yes	Yes	Yes	Yes	Yes	Yes	Yes	Yes	Yes											[9/9] (100%)	NA	100%
Luna, E. J. de A.; Lopes, M. de F. C.; Medina, N. H.; Favacho, J.; Cardoso, M. R. A.Prevalence of trachoma in schoolchildren in Brazil. 2016. Ophthalmic Epidemiology. , volume: 23, issue: 6, pages: 360-365. Doi: http://dx.doi.org/10.1080/09286586.2016.1244274.	Brazil	Cross-sectional	TF	TI	no	no	no	no	Checklist for Prevalence Studies	Unclear, school-based	Yes	Yes	Yes	Yes	Yes	Yes	Yes	Yes											[8/9] (89%)	NA	89%
Lynch, K. D.; Morotti, W.; Brian, G.; Ketchup, L.; Kingston, K.; Starr, M.; Ware, R. S.; Everill, B.; Asgar, N.; O'Keefe, A.; Whop, L. J.; Kaldor, J. M.; Lambert, S. B.Clinical signs of trachoma and laboratory evidence of ocular Chlamydia trachomatis infection in a remote Queensland community: a serial cross-sectional study. 2022. Medical Journal of Australia.. , volume: , issue: , pages: . Doi: https://dx.doi.org/10.5694/mja2.51735.	Australia	Cross-sectional	TF	<5 follicles	no	yes	yes	no	Both	Yes	Unclear	Yes	Yes	Yes	Yes	Yes	Yes	No	Unclear	Yes	Yes	Unclear	Yes	Yes	Yes	Yes	Yes	Yes	[7/9] (78%)	[8/10] (80%)	80%
Lynch, K. D.; Apadinuwe, S. C.; Lambert, S. B.; Hillgrove, T.; Starr, M.; Catlett, B.; Ware, R. S.; Cama, A.; Webster, S.; Harding-Esch, E. M.; Bakhtiari, A.; Butcher, R.; Cunningham, P.; Martin, D.; Gwyn, S.; Solomon, A. W.; Garabwan, C.; Kaldor, J. M.; Nery, S. V.A national survey integrating clinical, laboratory, and WASH data to determine the typology of trachoma in Nauru. 2022. PLoS Neglected Tropical Diseases. , volume: 16, issue: 4, pages: . Doi: http://dx.doi.org/10.1371/journal.pntd.0010275.	Nauru	Cross-sectional	TF	no	no	yes	yes	yes	Both	Yes	Yes	Yes	Yes	Yes	Yes	Yes	Yes	Yes	Random	Yes	Yes	Yes	Yes	Yes	Yes	Yes	Yes	Yes	[9/9] (100%)	[9/10] (90%)	100%
Lynch, K. D.; Brian, G.; Ahwang, T.; Newie, T.; Newie, V.; Perrett, C.; Wharton, G.; Brown, A.; Tozer, S.; Kaldor, J. M.; Whop, L. J.; Andrews, R. M.; Lambert, S. B.Discord between presence of follicular conjunctivitis and Chlamydia trachomatis infection in a single Torres Strait Island community: a cross-sectional survey. 2022. Australian and New Zealand journal of public health. , volume: 46, issue: 2, pages: 155-160. Doi: http://dx.doi.org/10.1111/1753-6405.13179.	Australia	Cross-sectional	TF	<5 follicles	no	yes	no	no	Checklist for Prevalence Studies	Yes	Yes	Yes	Yes	Yes	Yes	Yes	Yes	Yes											[9/9] (100%)	NA	100%
Mabey, D. C.; Bailey, R. L.; Dunn, D.; Jones, D.; Williams, J. H.; Whittle, H. C.; Ward, M. E.Expression of MHC class II antigens by conjunctival epithelial cells in trachoma: implications concerning the pathogenesis of blinding disease. 1991. Journal of Clinical Pathology. , volume: 44, issue: 4, pages: 285-9. Doi: .	Gambia	Case-control	F2/F3	P3	no	no	no	no	Checklist for Prevalence Studies	Unclear	Unclear	Unclear	Yes	Unclear	Yes	Yes	Yes	Unclear											[4/9] (44%)	NA	44%
Macleod, C. K.; Binnawi, K. H.; Elshafie, B. E.; Sadig, H. E.; Hassan, A.; Cocks, N.; Willis, R.; Chu, B.; Solomon, A. W.Unimproved water sources and open defecation are associated with active trachoma in children in internally displaced persons camps in the Darfur States of Sudan. 2019. Transactions of the Royal Society of Tropical Medicine and Hygiene. , volume: 113, issue: 10, pages: 599-609. Doi: http://dx.doi.org/10.1093/trstmh/trz042.	Sudan	Cross-sectional	TF	TI	no	no	no	no	Checklist for Prevalence Studies	Yes	Yes	Yes	Yes	Yes	Yes	Yes	Yes	Yes											[9/9] (100%)	NA	100%
Macleod, C. K.; Butcher, R.; Javati, S.; Gwyn, S.; Jonduo, M.; Abdad, M. Y.; Roberts, C. H.; Keys, D.; Koim, S. P.; Ko, R.; Garap, J.; Pahau, D.; Houinei, W.; Martin, D. L.; Pomat, W. S.; Solomon, A. W.Trachoma, anti-Pgp3 serology, and ocular Chlamydia trachomatis infection in Papua New Guinea. 2020. Clinical Infectious Diseases. , volume: 72, issue: 3, pages: 423-430. Doi: http://dx.doi.org/10.1093/cid/ciaa042.	Papua New Guinea	Cross-sectional	TF	no	no	yes	yes	yes	Checklist for Prevalence Studies	Yes	Yes	Yes	Yes	Unclear	Yes	Yes	Yes	Unclear											[7/9] (78%)	NA	78%
Madani, M. O.; Huguet, P.; Mariotti, S. P.; Dezoumbe, D.; Tosi, C.; Djada, D.; Negrel, A. D.Trachoma in Northern Chad: Epidemiological survey. [French]. 2003. Cahiers Sante. , volume: 13(1), issue: , pages: 45184. Doi: .	Chad	Cross-sectional	TF	TI	no	no	no	no	Checklist for Prevalence Studies	Unclear	Unclear	Unclear	No	Yes	Yes	Yes	Unclear	Yes											[4/9] (44%)	NA	44%
Sumit, Malhotra; Praveen, Vashist; Noopur, Gupta; Mani, Kalaivani; Gita, Satpathy; Anita, Shah; Sujaya, Krishnan; Rajvardhan, AzadPrevalence of trachoma in Car-Nicobar Island, India after three annual rounds of mass drug administration with azithromycin. 2016. PLoS ONE [Electronic Resource]. , volume: 11, issue: 7, pages: e0158625. Doi: http://dx.doi.org/10.1371/journal.pone.0158625.	India	Cross-sectional	TF	TI	no	no	no	no	Checklist for Prevalence Studies	Yes	Yes	Yes	Yes	Unclear	Yes	Yes	Yes	Yes											[8/9] (89%)	NA	89%
Martin, D. L.; Wiegand, R.; Goodhew, B.; Lammie, P.; Black, C. M.; West, S.; Gaydos, C. A.; Dize, L.; Mkocha, H.; Kasubi, M.; Gambhir, M.Serological Measures of Trachoma Transmission Intensity. 2015. Scientific Reports. , volume: 5, issue: , pages: 18532. Doi: https://dx.doi.org/10.1038/srep18532.	Tanzania	Cross-sectional	TF	no	no	yes	yes	no	Checklist for Prevalence Studies	Unclear	Unclear	Unclear	No	Unclear	Yes	Yes	Yes	Unclear											[3/9] (33%)	NA	33%
Martin, D. L.; Bid, R.; Sandi, F.; Goodhew, E. B.; Massae, P. A.; Lasway, A.; Philippin, H.; Makupa, W.; Molina, S.; Holland, M. J.; Mabey, D. C. W.; Drakeley, C.; Lammie, P. J.; Solomon, A. W.Serology for trachoma surveillance after cessation of mass drug administration. 2015. PLoS Neglected Tropical Diseases. , volume: 9, issue: 2, pages: e0003555. Doi: http://dx.doi.org/10.1371/journal.pntd.0003555.	Tanzania	Cross-sectional	TF	TI	no	yes	yes	yes	Checklist for Prevalence Studies	Yes	Yes	Yes	Yes	Low coverage of total pop (58%), coverage in 1-9 was higher (72%)	Yes	Yes	Yes	No											[7/9] (78%)	NA	78%
Mathew, A. A.; Keeffe, J. E.; Mesurier, R. T. le; Taylor, H. R.Trachoma in the Pacific Islands: evidence from Trachoma Rapid Assessment. 2009. British Journal of Ophthalmology. , volume: 93, issue: 7, pages: 866-870. Doi: http://dx.doi.org/10.1136/bjo.2008.151720.	Fiji, Kiribati, Nauru, Solomon Islands, Vanuatu	Cross-sectional	TF	TI	no	no	no	no	Checklist for Prevalence Studies	Yes	No, TRA	No	Yes	Low coverage (<80%)	Yes	Yes	Yes	Unclear											[5/9] (56%)	NA	56%
Medina, N. H.; Massaini, M. G.; Azevedo, C. L. B.; Harima, C.; Prado, M.; Maluf, S.; Marcucci, M.; Caligaris, L. S. A.; Morimoto, W. T. M.Epidemiological surveillance of trachoma in a school in the city of Sao Paulo, SP. 1998. Vigilancia epidemiologica do tracoma em instituicao de ensino na cidade de Sao Paulo, SP.. , volume: 32, issue: 1, pages: 59-63. Doi: http://dx.doi.org/10.1590/S0034-89101998000100008.	Brazil	Cross-sectional	TF	TF and/or TI	no	no	no	no	Checklist for Prevalence Studies	Yes	Yes	Yes	No	Yes	Yes	Yes	Yes	Yes											[8/9] (89%)	NA	89%
Meng, N.; Seiha, D.; Thorn, P.; Willis, R.; Flueckiger, R. M.; Dejene, M.; Lewallen, S.; Courtright, P.; Solomon, A. W.Assessment of trachoma in Cambodia: trachoma is not a public health problem. 2016. Special Issue: Trachoma mapping.. , volume: 23, issue: Suppl. 1, pages: 44992. Doi: .	Cambodia	Cross-sectional	TF	TI	no	no	no	no	Checklist for Prevalence Studies	Yes	Yes	Yes	No	Yes	Yes	Yes	Yes	Yes											[8/9] (89%)	NA	89%
Mesfin, M. M.; Camera, J. de la; Tareke, I. G.; Amanual, G.; Araya, T.; Kedir, A. M.A community-based trachoma survey: prevalence and risk factors in the Tigray region of Northern Ethiopia. 2006. Ophthalmic Epidemiology. , volume: 13, issue: 3, pages: 173-181. Doi: http://dx.doi.org/10.1080/09286580600611427.	Ethiopia	Cross-sectional	TF	TI	no	no	no	no	Checklist for Prevalence Studies	Yes	Yes	Unclear	Yes	Yes	Yes	Yes	Yes	Yes											[8/9] (89%)	NA	89%
Michel, C. E. C.; Solomon, A. W.; Magbanua, J. P.; Massae, P. A.; Huang, L.; Mosha, J.; West, S. K.; Nadala, E. C.; Bailey, R.; Wisniewski, C.; Mabey, D. C.; Lee, H. H.Field evaluation of a rapid point-of-care assay for targeting antibiotic treatment for trachoma control: a comparative study. 2006. Lancet. , volume: 367(9522), issue: , pages: 1585-1590. Doi: https://dx.doi.org/10.1016/S0140-6736%2806%2968695-9.	Tanzania	Cross-sectional	TF	no	no	yes	no	no	Both	Yes	Unclear	Unclear	No	Yes	Yes	Yes	Yes	Yes	Unclear	Yes	Yes	Unclear	Yes	Yes	Yes	Yes	Yes	Yes	[6/9] (67%)	[8/10] (80%)	80%
Michel, C. E. C.; Roper, K. G.; Divena, M. A.; Lee, H. H.; Taylor, H. R.Correlation of clinical trachoma and infection in aboriginal communities. 2011. PLoS Neglected Tropical Diseases. , volume: 5, issue: 3, pages: e986. Doi: http://dx.doi.org/10.1371/journal.pntd.0000986.	Australia	Cross-sectional	TF	no	no	yes	no	no	Checklist for Prevalence Studies	Yes	Yes	Yes	Yes	Yes	Yes	Yes	Yes	Yes											[9/9] (100%)	NA	100%

S6. Table of included studies

										Risk of Bias checklist items**																					
reference	Country or countries of data collection	Type of study	TF or FPC	other clinical indicators?	photo-graded TF or FPC?	infection?	serology?	SCR?	Critical Appraisal Tool used**	Prevalence study: Was the sample frame appropriate to address the target pop?	Prevalence study: Were study participants sampled in an appropriate way?	Prevalence study: Was the sample size adequate?	Prevalence study: Were the study subjects and setting described in detail?	Prevalence study: Was the data analysis conducted with sufficient coverage of the identified sample?	Prevalence study: Were valid methods used for the identification of the condition(s)?	Prevalence study: Was the condition measured in a standard, reliable way for all participants?	Prevalence study: Was there appropriate statistical analysis?	Prevalence study: Was the response rate adequate, and if not, was the low response rate managed appropriately?	DTA study: Was a consecutive or random sample of patients enrolled?	DTA study: Was a case-control design avoided?	DTA study: Did the study avoid inappropriate exclusions?	DTA: Were the index test results interpreted without knowledge of the results of the reference standard?	DTA: If a threshold was used, was it pre-specified?	DTA: Is the reference standard likely to correctly classify the target condition?	DTA: Were the reference standard results interpreted without knowledge of the results of the index test?	DTA: Was there an appropriate interval between index test and reference standard?	DTA: Did all patients receive the same reference standard?	DTA: Were all patients included in the analysis?	Prevalence study score [meets criteria/total] (%)	DTA study score [meets criteria/total] (%)	Max score (if both checklists used, higher of 2 scores)
Migchelsen, S. J.; Martin, D. L.; Southisombath, K.; Turyaguma, P.; Heggen, A.; Rubangakene, P. P.; Joof, H.; Makalo, P.; Cooley, G.; Gwyn, S.; Solomon, A. W.; Holland, M. J.; Courtright, P.; Willis, R.; Alexander, N. D. E.; Mabey, D. C. W.; Roberts, C. H.Defining seropositivity thresholds for use in trachoma elimination studies. 2017. PLoS Neglected Tropical Diseases. , volume: 11, issue: 1, pages: e0005230. Doi: http://dx.doi.org/10.1371/journal.pntd.0005230.	Gambia, Laos, Uganda	Cross-sectional	TF	TI	no	no	yes	yes	Both	Yes	Yes	Unclear	Yes	Unclear	Yes	Yes	Yes	Unclear	Random	Yes	Yes	Yes	Yes	Yes	Yes	Yes	Yes	Yes	[6/9] (67%)	[9/10] (90%)	90%
Alfonso Miller, H.; Lopez Mesa, C. B. de; Talero, S. L.; Meza Cardenas, M.; Ramirez, S. P.; Moreno-Montoya, J.; Porras, A.; Trujillo-Trujillo, J.Prevalence of trachoma and associated factors in the rural area of the department of Vaupes, Colombia. 2020. PLoS ONE [Electronic Resource]. , volume: 15, issue: 5, pages: . Doi: http://dx.doi.org/10.1371/journal.pone.0229297.	Colombia	Cross-sectional	TF	TI	no	no	no	no	Checklist for Prevalence Studies	Yes	Yes	Unclear	Yes	Yes	Yes	Yes	Yes	Yes											[8/9] (89%)	NA	89%
Miller, K.; Schmidt, G.; Melese, M.; Alemayehu, W.; Yi, E.; Cevallos, V.; Donnellan, C.; Olinger, L.; Fantaye, D.; Gaynor, B.; Whitcher, J. P.; Lietman, T.How reliable is the clinical exam in detecting ocular chlamydial infection?. 2004. Ophthalmic Epidemiology. , volume: 11, issue: 3, pages: 255-62. Doi: .	Ethiopia	Cross-sectional	TF	TI, TF and/or TI, TF and TI	no	yes	no	no	Checklist for DTA studies										Random	Yes	Yes	Yes	Yes	Yes	Yes	Yes	Yes	Yes	NA	[9/10] (90%)	90%
Miller, H.; Gallego, G.; Rodriguez, G.Clinical evidence of trachoma in Colombian Amerindians of the Vaupes Province. [Spanish]. 2010. Biomedica. , volume: 30(3), issue: , pages: 432-439. Doi: .	Colombia	Cross-sectional	TF	TI	no	no	no	no	Checklist for Prevalence Studies	Yes	Yes	No	Yes	No	Yes	Yes	Yes	No											[6/9] (67%)	NA	67%
Missamou, F.; Marlhand, H.; Dzabatou-Babeaux, A. S. P.; Sendzi, S.; Bernasconi, J.; D'Souza, S.; Bakhtiari, A.; Millar, T.; Willis, R.; Bengraine, K.; Resnikoff, S.; Solomon, A. W.A population-based trachoma prevalence survey covering seven districts of Sangha and Likouala departments, Republic of the Congo. 2018. Special Issue: 2017 Global Trachoma Mapping Project.. , volume: 25, issue: Suppl. 1, pages: 155-161. Doi: http://dx.doi.org/10.1080/09286586.2018.1546878.	Republic of the Congo	Cross-sectional	TF	TI	no	no	no	no	Checklist for Prevalence Studies	Yes	Yes	Yes	No	Yes	Yes	Yes	Yes	Yes											[8/9] (89%)	NA	89%
Mkocha, H.; Munoz, B.; West, S.Trachoma and ocular Chlamydia trachomatis rates in children in trachoma-endemic communities enrolled for at least three years in the Tanzania National Trachoma Control Programme. 2009. Tanzania journal of health research. , volume: 11, issue: 3, pages: 103-110. Doi: .	Tanzania	Cross-sectional	TF	TI	no	yes	no	no	Checklist for Prevalence Studies	Yes	Yes	Yes	No	Unclear	Yes	Yes	Yes	Unclear											[6/9] (67%)	NA	67%
Mohammadi, S. F.; Bazi-Shad, A.; Akbarizadeh, J.; Ashrafi, E.; Moinfar, Z.; Shirdel, E.; Nabavi, M.; Samiei, S.; Salehi-Rad, S.; Solomon, A. W.Follow-up and report on active trachoma in Zabol, Iran, prompted by Sharifi-Rad and Fallah's observations published in May 2016. 2017. New Microbes and New Infections. , volume: 20, issue: , pages: 14-15. Doi: https://dx.doi.org/10.1016/j.nmni.2017.08.001.	Iran	Cross-sectional	TF	no	no	yes	no	no	Checklist for Prevalence Studies	Yes	No, only children w/ red eye screened for trachoma	Yes	No	No	Unclear	Yes	Yes	Unclear											[4/9] (44%)	NA	44%
Mpyet, C.; Ogoshi, C.; Goyol, M.Prevalence of trachoma in Yobe State, North-Eastern Nigeria. 2008. Ophthalmic Epidemiology. , volume: 15, issue: 5, pages: 303-307. Doi: http://dx.doi.org/10.1080/09286580802237633.	Nigeria	Cross-sectional	TF	TI, TF and/or TI	no	no	no	no	Checklist for Prevalence Studies	Yes	Yes	Unclear	Yes	Unclear	Yes	Yes	Yes	Unclear											[6/9] (67%)	NA	67%
Mpyet, C.; Goyol, M.; Ogoshi, C.Personal and environmental risk factors for active trachoma in children in Yobe state, north-eastern Nigeria. 2010. Tropical Medicine and International Health. , volume: 15, issue: 2, pages: 168-172. Doi: http://dx.doi.org/10.1111/j.1365-3156.2009.02436.x.	Nigeria	Cross-sectional	TF	TI, TF and/or TI	no	no	no	no	Checklist for Prevalence Studies	Yes	Yes	Yes	Yes	Yes	Yes	Yes	Yes	Yes											[9/9] (100%)	NA	100%
Mpyet, C.; Lass, B. D.; Yahaya, H. B.; Solomon, A. W.Prevalence of and risk factors for trachoma in Kano State, Nigeria. 2012. PLoS ONE [Electronic Resource]. , volume: 7, issue: 7, pages: e40421. Doi: http://dx.doi.org/10.1371/journal.pone.0040421.	Nigeria	Cross-sectional	TF	TI	no	no	no	no	Checklist for Prevalence Studies	Yes	Yes	Yes	Yes	Unclear	Yes	Yes	Yes	Unclear											[7/9] (78%)	NA	78%
Muhammad, N.; Mpyet, C.; Adamu, M. D.; William, A.; Umar, M. M.; Muazu, H.; Onyebuchi, U.; Isiyaku, S.; Flueckiger, R. M.; Chu, B. K.; Willis, R.; Pavluck, A.; Dalhatu, A.; Ogoshi, C.; Olobio, N.; Gordon, B. A.; Solomon, A. W.Prevalence of trachoma in the Area Councils of the Federal Capital Territory, Nigeria: results of six population-based surveys. 2018. Special Issue: 2017 Global Trachoma Mapping Project.. , volume: 25, issue: Suppl. 1, pages: 45247. Doi: http://dx.doi.org/10.1080/09286586.2017.1367409.	Nigeria	Cross-sectional	TF	TI	no	no	no	no	Checklist for Prevalence Studies	Yes	Yes	Yes	No	Yes	Yes	Yes	Yes	Yes											[8/9] (89%)	NA	89%
Munoz, B.; Stare, D.; Mkocha, H.; Gaydos, C.; Quinn, T.; West, S. K.Can clinical signs of trachoma be used after multiple rounds of mass antibiotic treatment to indicate infection?. 2011. Investigative Ophthalmology & Visual Science. , volume: 52, issue: 12, pages: 8806-10. Doi: https://dx.doi.org/10.1167/iovs.11-8074.	Tanzania	Cross-sectional	TF	no	yes	yes	no	no	Both	Yes	Yes	Yes	No	Unclear	Yes	Yes	Yes	Unclear	Yes	Yes	Yes	Unclear	Yes	Yes	Yes	Yes	Yes	Yes	[6/9] (67%)	[9/10] (90%)	90%
Nash, S. D.; Sata, E.; Stewart, A. E. P.; Astale, T.; Zerihun, M.; Gessese, D.; Ayenew, G.; Chanyalew, M.; Melak, B.; Tadesse, Z.; Callahan, E. K.Longitudinal trends in trachoma over eight years in a hyperendemic setting under the safe strategy: Results from serial impact surveys in west gojjam zone, Ethiopia. 2017. American Journal of Tropical Medicine and Hygiene. , volume: 97(5 Supplement 1), issue: , pages: 228. Doi: .	Ethiopia	Cross-sectional	TF	TI	no	yes	no	no	Checklist for Prevalence Studies	Yes	Unclear	Unclear	No	Unclear	Unclear	Yes	Unclear	Unclear											[2/9] (22%)	NA	22%
Nash, S. D.; Stewart, A. E. P.; Astale, T.; Sata, E.; Zerihun, M.; Gessese, D.; Melak, B.; Ayenew, G.; Ayele, Z.; Bayissasse, B.; Chanyalew, M.; Tadesse, Z.; Kelly Callahan, E.Trachoma prevalence remains below threshold in five districts after stopping mass drug administration: Results of five surveillance surveys within a hyperendemic setting in Amhara, Ethiopia. 2018. Transactions of the Royal Society of Tropical Medicine and Hygiene. , volume: 112(12), issue: , pages: 538-545. Doi: https://dx.doi.org/10.1093/trstmh/try096.	Ethiopia	Cross-sectional	TF	TI	no	yes	no	no	Checklist for Prevalence Studies	Yes	Yes	Unclear	No	Unclear	Yes	Yes	Yes	Unclear											[5/9] (56%)	NA	56%
Nash, S. D.; Stewart, A. E. P.; Zerihun, M.; Sata, E.; Gessese, D.; Melak, B.; Endeshaw, T.; Chanyalew, M.; Chernet, A.; Bayissasse, B.; Moncada, J.; Lietman, T. M.; Emerson, P. M.; King, J. D.; Tadesse, Z.; Callahan, E. K.Ocular Chlamydia trachomatis infection under the surgery, antibiotics, facial cleanliness, and environmental improvement strategy in Amhara, Ethiopia, 2011-2015. 2018. Clinical Infectious Diseases. , volume: 67, issue: 12, pages: 1840-1846. Doi: http://dx.doi.org/10.1093/cid/ciy377.	Ethiopia	Cross-sectional	TF	TI	no	yes	no	no	Checklist for Prevalence Studies	Yes	Yes	Unclear	Yes	Unclear	Yes	Yes	Yes	Unclear											[6/9] (67%)	NA	67%
Nash, S. D.; Chernet, A.; Moncada, J.; Stewart, A. E. P.; Astale, T.; Sata, E.; Zerihun, M.; Gessese, D.; Melak, B.; Ayenew, G.; Ayele, Z.; Chanyalew, M.; Lietman, T. M.; Callahan, E. K.; Schachter, J.; Tadesse, Z.Ocular chlamydia trachomatis infection and infectious load among pre-school aged children within trachoma hyperendemic districts receiving the safe strategy, Amhara Region, Ethiopia. 2020. PLoS Neglected Tropical Diseases. , volume: 14(5), issue: , pages: 44942. Doi: https://dx.doi.org/10.1371/journal.pntd.0008226.	Ethiopia	Cross-sectional	TF	TI	no	yes	no	no	Checklist for Prevalence Studies	Yes	Yes	Unclear	Yes	Unclear	Yes	Yes	Yes	Unclear											[6/9] (67%)	NA	67%
Nash, S. D.; Chernet, A.; Astale, T.; Sata, E.; Zerihun, M.; Nute, A. W.; Jensen, K. A.; Gessese, D.; Ayele, Z.; Melak, B.; Haile, M.; Zeru, T.; Tadesse, Z.; Callahan, E. K.Population-based prevalence of ocular Chlamydia trachomatis infection among infants in the trachoma endemic Amhara region, Ethiopia. 2022. American Journal of Tropical Medicine and Hygiene. , volume: 106, issue: 1, pages: 62-65. Doi: http://dx.doi.org/10.4269/ajtmh.21-0873.	Ethiopia	Cross-sectional	TF	TI	no	yes	no	no	Checklist for Prevalence Studies	Yes	Yes	Unclear	No	Unclear	Yes	Yes	Yes	Unclear											[5/9] (56%)	NA	56%
Natividad, A.; Freeman, T. C.; Jeffries, D.; Burton, M. J.; Mabey, D. C. W.; Bailey, R. L.; Holland, M. J.Human conjunctival transcriptome analysis reveals the prominence of innate defense in Chlamydia trachomatis infection. 2010. Infection and Immunity. , volume: 78, issue: 11, pages: 4895-4911. Doi: http://dx.doi.org/10.1128/IAI.00844-10.	Gambia	Cross-sectional	F2/F3	P3	no	yes	no	no	Checklist for DTA studies										Unclear	No	No, excluded on basis of ability to detect RNA	Unclear	Yes	Yes	Yes	Yes	Yes	No, omitted people without adequate quantity of RNA	NA	[5/10] (50%)	50%
Naufal, F.; Brady, C. J.; Wolle, M. A.; Kashaf, M. S.; Mkocha, H.; Bradley, C.; Kabona, G.; Ngondi, J.; Massof, R. W.; West, S. K.Evaluation of photography using head-mounted display technology (ICAPS) for district trachoma surveys. 2021. PLoS Neglected Tropical Diseases. , volume: 15, issue: 11, pages: . Doi: http://dx.doi.org/10.1371/journal.pntd.0009928.	Tanzania	Cross-sectional	TF	no	yes	no	no	no	Both	Unclear	Unclear	Unclear	No	Unclear	Yes	Yes	Yes	Unclear	Random	Yes	Yes	Yes	Yes	Yes	Yes	Yes	Yes	Yes, although ungradeable images excluded for some analyses	[3/9] (33%)	[8/10] (80%)	80%
Ndisabiye, D.; Gahungu, A.; Kayugi, D.; Waters, E. K.Association of environmental risk factors and trachoma in Gashoho Health district, Burundi. 2020. African Health Sciences. , volume: 20, issue: 1, pages: 182-189. Doi: http://dx.doi.org/10.4314/ahs.v20i1.23.	Burundi	Cross-sectional	TF	TI, TF and/or TI	no	no	no	no	Checklist for Prevalence Studies	Unclear, convenience sample from clinic	Unclear	Yes	No	Yes	Unclear	Yes	Yes	Yes											[5/9] (56%)	NA	56%
Negrel, A. D.; Khazraji, Y. C.; Akalay, O.Trachoma in the province of Ouarzazate Morocco. [French]. 1992. Bulletin of the World Health Organization. , volume: 70(4), issue: , pages: 451-456. Doi: .	Morocco	Cross-sectional	TF	TF and/or TI	no	no	no	no	Checklist for Prevalence Studies	Yes	Yes	Yes	Yes	Yes	Yes	Yes	Yes	Yes											[9/9] (100%)	NA	100%
Nesemann, J. M.; Seider, M. I.; Snyder, B. M.; Maamari, R. N.; Fletcher, D. A.; Haile, B. A.; Tadesse, Z.; Varnado, N. E.; Cotter, S. Y.; Callahan, E. K.; Emerson, P. M.; Margolis, T. P.; Lietman, T. M.; Keenan, J. D.Comparison of smartphone photography, single-lens reflex photography, and field-grading for trachoma. 2020. American Journal of Tropical Medicine and Hygiene. , volume: 103(6), issue: , pages: 2488-2491. Doi: https://dx.doi.org/10.4269/ajtmh.20-0386.	Ethiopia	Cross-sectional	TF	TI	yes	no	no	no	Both	Yes	Yes	No	No	Unclear	Yes	Yes	Yes	Unclear	Random	Yes	Yes	Yes	Yes	Yes	Yes	Yes	Yes	Yes	[5/9] (56%)	[9/10] (90%)	90%
Nesemann, J.; Munoz, M. B.; Morocho-Alburqueque, N.; Quincho-Lopez, A.; Wittberg, D. M.; Honorio, H.; Durand, S.; Saboya-Diaz, M. I.; Harding-Esch, E. M.; Talero, S.; Solomon, A. W.; Lescano, A. G.; Keenan, J. D.Prevalence of trachoma in alto amazonas, loreto department, Peru. 2021. Investigative Ophthalmology and Visual Science. Conference: Annual Meeting Association for Research in Vision and Ophthalmology, ARVO. , volume: 62, issue: 8, pages: . Doi: .	Peru	Cross-sectional	TF	no	no	yes	no	no	Checklist for Prevalence Studies	Yes	Yes	Unclear	No	Yes	Yes	Yes	Yes	Yes											[7/9] (78%)	NA	78%

										Risk of Bias checklist items**																					
reference	Country or countries of data collection	Type of study	TF or FPC	other clinical indicators?	photo-graded TF or FPC?	infection?	serology?	SCR?	Critical Appraisal Tool used**	Prevalence study: Was the sample frame appropriate to address the target pop?	Prevalence study: Were study participants sampled in an appropriate way?	Prevalence study: Was the sample size adequate?	Prevalence study: Were the study subjects and setting described in detail?	Prevalence study: Was the data analysis conducted with sufficient coverage of the identified sample?	Prevalence study: Were valid methods used for the identification of the condition(s)?	Prevalence study: Was the condition measured in a standard, reliable way for all participants?	Prevalence study: Was there appropriate statistical analysis?	Prevalence study: Was the response rate adequate, and if not, was the low response rate managed appropriately?	DTA study: Was a consecutive or random sample of patients enrolled?	DTA study: Was a case-control design avoided?	DTA study: Did the study avoid inappropriate exclusions?	DTA: Were the index test results interpreted without knowledge of the results of the reference standard?	DTA: If a threshold was used, was it pre-specified?	DTA: Is the reference standard likely to correctly classify the target condition?	DTA: Were the reference standard results interpreted without knowledge of the results of the index test?	DTA: Was there an appropriate interval between index test and reference standard?	DTA: Did all patients receive the same reference standard?	DTA: Were all patients included in the analysis?	Prevalence study score [meets criteria/total] (%)	DTA study score [meets criteria/total] (%)	Max score (if both checklists used, higher of 2 scores)
Ngondi, J.; Onsarigo, A.; Adamu, L.; Matende, I.; Baba, S.; Reacher, M.; Emerson, P.; Zingeser, J.The epidemiology of trachoma in Eastern Equatoria and Upper Nile States, southern Sudan. 2005. Bulletin of the World Health Organization. , volume: 83, issue: 12, pages: 904-912. Doi: .	South Sudan	Cross-sectional	TF	TI, TF and/or TI	no	no	no	no	Checklist for Prevalence Studies	Yes	Yes	Yes	Yes	Yes	Yes	Yes	Yes	Yes											[9/9] (100%)	NA	100%
Ngondi, J.; Onsarigo, A.; Matthews, F.; Reacher, M.; Brayne, C.; Baba, S.; Solomon, A. W.; Zingeser, J.; Emerson, P. M.Effect of 3 years of SAFE (surgery, antibiotics, facial cleanliness, and environmental change) strategy for trachoma control in southern Sudan: a cross-sectional study. 2006. Lancet (British edition). , volume: 368, issue: 9535, pages: 589-595. Doi: http://dx.doi.org/10.1016/S0140-6736(06)69202-7.	South Sudan	Cross-sectional	TF	TI, TF and/or TI	no	no	no	no	Checklist for Prevalence Studies	Yes	Yes	Unclear	Yes	Unclear	Yes	Yes	Yes	Unclear											[6/9] (67%)	NA	67%
Ngondi, J.; Ole-Sempele, F.; Onsarigo, A.; Matende, I.; Baba, S.; Reacher, M.; Matthews, F.; Brayne, C.; Emerson, P.Blinding trachoma in postconflict southern Sudan. 2006. PLoS Medicine. , volume: 3, issue: 12, pages: e478. Doi: http://dx.doi.org/10.1371/journal.pmed.0030478.	South Sudan	Cross-sectional	TF	TI, TF and/or TI	no	no	no	no	Checklist for Prevalence Studies	Yes	Yes	Yes	Yes	Yes	Yes	Yes	Yes	Yes											[9/9] (100%)	NA	100%
Ngondi, J.; Teshome, Gebre; Shargie, E. B.; Graves, P. M.; Yeshewamebrat, Ejigsemahu; Tesfaye, Teferi; Asrat, Genet; Mosher, A. W.; Tekola, Endeshaw; Mulat, Zerihun; Ayenew, Messele; Richards, F. O., Jr.; Emerson, P. M.Risk factors for active trachoma in children and trichiasis in adults: a household survey in Amhara Regional State, Ethiopia. 2008. Transactions of the Royal Society of Tropical Medicine and Hygiene. , volume: 102, issue: 5, pages: 432-438. Doi: http://dx.doi.org/10.1016/j.trstmh.2008.02.014.	Ethiopia	Cross-sectional	TF	TI	no	no	no	no	Checklist for Prevalence Studies	Yes	Yes	Yes	Yes	Yes	Yes	Yes	Yes	Yes											[9/9] (100%)	NA	100%
Ngondi, J.; Gebre, T.; Shargie, E. B.; Adamu, L.; Ejigsemahu, Y.; Teferi, T.; Zerihun, M.; Ayele, B.; Cevallos, V.; King, J.; Emerson, P. M.Evaluation of three years of the SAFE strategy (Surgery, Antibiotics, Facial cleanliness and Environmental improvement) for trachoma control in five districts of Ethiopia hyperendemic for trachoma. 2009. Transactions of the Royal Society of Tropical Medicine and Hygiene. , volume: 103(10), issue: , pages: 1001-1010. Doi: https://dx.doi.org/10.1016/j.trstmh.2008.11.023.	Ethiopia	Cross-sectional	TF	TI, TF and/or TI	no	yes	no	no	Checklist for Prevalence Studies	Yes	Yes	Unclear	Yes	Unclear	Yes	Yes	Yes	Unclear											[6/9] (67%)	NA	67%
Ngondi, J.; Teshome, Gebre; Shargie, E. B.; Liknaw, Adamu; Tesfaye, Teferi; Mulat, Zerihun; Berhan, Ayele; King, J. D.; Cromwell, E. A.; Emerson, P. M.Estimation of effects of community intervention with Antibiotics, Facial cleanliness, and Environmental improvement (A,F,E) in five districts of Ethiopia hyperendemic for trachoma. 2010. British Journal of Ophthalmology. , volume: 94, issue: 3, pages: 278-281. Doi: http://dx.doi.org/10.1136/bjo.2009.168260.	Ethiopia	Cross-sectional	TF	TI	no	no	no	no	Checklist for Prevalence Studies	Yes	Yes	Unclear	Yes	Unclear	Yes	Yes	Yes	Unclear											[6/9] (67%)	NA	67%
Nigusie, A.; Berhe, R.; Gedefaw, M.Prevalence and associated factors of active trachoma among childeren aged 1-9 years in rural communities of Gonji Kolella district, West Gojjam zone, North West Ethiopia. 2015. BMC Research Notes. , volume: 8, issue: , pages: 641. Doi: https://dx.doi.org/10.1186/s13104-015-1529-6.	Ethiopia	Cross-sectional	TF	TI, TF and/or TI	no	no	no	no	Checklist for Prevalence Studies	Yes	Yes	Yes	Yes	Yes	Yes	Yes	Yes	Yes											[9/9] (100%)	NA	100%
Noatina, B. N.; Kagmeni, G.; Mengouo, M. N.; Moungui, H. C.; Tarini, A.; Zhang, YaoBi; Bella, A. L. F.Prevalence of trachoma in the Far North region of Cameroon: results of a survey in 27 health districts. 2013. PLoS Neglected Tropical Diseases. , volume: 7, issue: 5, pages: e2240. Doi: http://dx.doi.org/10.1371/journal.pntd.0002240.	Cameroon	Cross-sectional	TF	TF and/or TI	no	no	no	no	Checklist for Prevalence Studies	Yes	Yes	No	Yes	Yes	Yes	Yes	Yes	Yes											[8/9] (89%)	NA	89%
Noatina, B. N.; Kagmeni, G.; Souleymanou, Y.; Moungui, H. C.; Hien, A. T.; Akame, J.; Zhang, YaoBi; Bella, A. L. F.Prevalence of trachoma in the north region of Cameroon: results of a survey in 15 health districts. 2014. PLoS Neglected Tropical Diseases. , volume: 8, issue: 6, pages: e2932. Doi: http://dx.doi.org/10.1371/journal.pntd.0002932.	Cameroon	Cross-sectional	TF	TF and/or TI	no	no	no	no	Checklist for Prevalence Studies	Unclear	Yes	Yes	Yes	Yes	Yes	Yes	Yes	Yes											[8/9] (89%)	NA	89%
Odonkor, M.; Naufal, F.; Munoz, B.; Mkocha, H.; Kasubi, M.; Wolle, M.; West, S.Serology, infection, and clinical trachoma as tools in prevalence surveys for re-emergence of trachoma in a formerly hyperendemic district. 2021. PLoS Neglected Tropical Diseases. , volume: 15, issue: 4, pages: . Doi: http://dx.doi.org/10.1371/journal.pntd.0009343.	Tanzania	Cross-sectional	TF	no	yes	yes	yes	no	Both	Yes	Yes	Yes	Yes	No, 78% coverage	Yes	Yes	Yes	Yes	Random	Yes	Yes	Yes	Yes	Yes	Yes	Yes	Yes	Depends on measure	[8/9] (89%)	[8/10] (80%)	89%
Odonkor, M.; Naufal, F.; Mkocha, H.; Funga, N.; Munoz, B.; West, S. K.The Impact of Image Quality and Trachomatous Inflammation on Using Photography for Trachoma Prevalence Surveys. 2022. Translational Vision Science & Technology. , volume: 11, issue: 3, pages: 11. Doi: https://dx.doi.org/10.1167/tvst.11.3.11.	Tanzania	Cross-sectional	TF	no	yes	no	no	no	Both	Unclear	Unclear	Unclear	No	Unclear	Yes	Yes	Yes	Unclear	Unclear	Yes	Yes	Yes	Yes	Yes	Yes	Yes	Yes	Yes	[3/9] (33%)	[9/10] (90%)	90%
Oldenburg, C. E.; Amza, A.; Kadri, B.; Nassirou, B.; Cotter, S. Y.; Stoller, N. E.; West, S. K.; Bailey, R. L.; Porco, T. C.; Gaynor, B. D.; Keenan, J. D.; Lietman, T. M.Comparison of mass azithromycin coverage targets of children in Niger: a cluster-randomized trachoma trial. 2018. American Journal of Tropical Medicine and Hygiene. , volume: 98, issue: 2, pages: 389-395. Doi: http://dx.doi.org/10.4269/ajtmh.17-0501.	Niger	Cross-sectional within longitudinal	TF	no	no	yes	no	no	Checklist for Prevalence Studies	Yes	Yes	Unclear	Yes	Unclear	Yes	Yes	Yes	Unclear											[6/9] (67%)	NA	67%
Oswald, W. E.; Stewart, A. E. P.; Kramer, M. R.; Endeshaw, T.; Zerihun, M.; Melak, B.; Sata, E.; Gessese, D.; Teferi, T.; Tadesse, Z.; Guadie, B.; King, J. D.; Emerson, P. M.; Callahan, E. K.; Flanders, D.; Moe, C. L.; Clasen, T. F.Active trachoma and community use of sanitation, Ethiopia. 2017. Bulletin of the World Health Organization. , volume: 95, issue: 4, pages: 250-260. Doi: http://dx.doi.org/10.2471/blt.16.177758.	Ethiopia	Cross-sectional	TF	TI, TF and/or TI	no	no	no	no	Checklist for Prevalence Studies	Yes	Yes	Unclear	No	Unclear	Yes	Yes	Yes	Unclear											[5/9] (56%)	NA	56%
Phiri, I.; Manangazira, P.; Macleod, C. K.; Mduluza, T.; Dhobbie, T.; Chaora, S. G.; Chigwena, C.; Katiyo, J.; Willis, R.; Bakhtiari, A.; Bare, P.; Courtright, P.; Macheka, B.; Midzi, N.; Solomon, A. W.The burden of and risk factors for trachoma in selected districts of Zimbabwe: results of 16 population-based prevalence surveys. 2018. Special Issue: 2017 Global Trachoma Mapping Project.. , volume: 25, issue: Suppl. 1, pages: 181-191. Doi: http://dx.doi.org/10.1080/09286586.2017.1298823.	Zimbabwe	Cross-sectional	TF	TI	no	no	no	no	Checklist for Prevalence Studies	Yes	Yes	Yes	Yes	Yes	Yes	Yes	Yes	Yes											[9/9] (100%)	NA	100%
Quicke, E.; Sillah, A.; Harding-Esch, E. M.; Last, A.; Joof, H.; Makalo, P.; Bailey, R. L.; Burr, S. E.Follicular trachoma and trichiasis prevalence in an urban community in The Gambia, West Africa: is there a need to include urban areas in national trachoma surveillance?. 2013. Tropical Medicine and International Health. , volume: 18, issue: 11, pages: 1344-1352. Doi: http://dx.doi.org/10.1111/tmi.12182.	Gambia	Cross-sectional	TF	TI	no	no	no	no	Checklist for Prevalence Studies	Yes	Yes	Unclear	Yes	Yes	Yes	Yes	Yes	Yes											[8/9] (89%)	NA	89%
Ramadhani, A. M.; Derrick, T.; Macleod, D.; Massae, P.; Malisa, A.; Mbuya, K.; Mtuy, T.; Makupa, W.; Roberts, C. H.; Bailey, R. L.; Mabey, D. C. W.; Holland, M. J.; Burton, M. J.Ocular immune responses, Chlamydia trachomatis infection and clinical signs of trachoma before and after azithromycin mass drug administration in a treatment naive trachoma-endemic Tanzanian community. 2019. PLoS Neglected Tropical Diseases. , volume: 13, issue: 7, pages: e0007559. Doi: http://dx.doi.org/10.1371/journal.pntd.0007559.	Tanzania	Cross-sectional within longitudinal	TF, F2/F3	TI, P2/P3	yes	yes	no	no	Checklist for Prevalence Studies	Yes	Unclear	Unclear	Yes	Unclear	Yes	Yes	Yes	Unclear											[5/9] (56%)	NA	56%
Ramyil, A.; Wade, P.; Ogoshi, C.; Goyol, M.; Adenuga, O.; Dami, N.; Mpyet, C.Prevalence of trachoma in Jigawa State, Northwestern Nigeria. 2015. Special Issue: Trachoma.. , volume: 22, issue: 3, pages: 184-189. Doi: .	Nigeria	Cross-sectional	TF	TI	no	no	no	no	Checklist for Prevalence Studies	Yes	Yes	Yes	Yes	Yes	Yes	Yes	Yes	Yes											[9/9] (100%)	NA	100%
Reda, G.; Yemane, D.; Gebreyesus, A.Prevalence and associated factors of active trachoma among 1-9years old children in Deguatemben, Tigray, Ethiopia, 2018: community cross-sectional study. 2020. BMC Ophthalmology. , volume: 20(1), issue: , pages: 144. Doi: https://dx.doi.org/10.1186/s12886-020-01394-0.	Ethiopia	Cross-sectional	TF	TI, TF and/or TI, TF and TI	no	no	no	no	Checklist for Prevalence Studies	Yes	Yes	Yes	Yes	Yes	Yes	Yes	Yes	Yes											[9/9] (100%)	NA	100%
Reilly, L. A.; Favacho, J.; Garcez, L. M.; Courtenay, O.Preliminary evidence that synanthropic flies contribute to the transmission of trachoma-causing Chlamydia trachomatis in Latin America. 2007. Cadernos de Saude Publica. , volume: 23, issue: 7, pages: 1682-1688. Doi: http://dx.doi.org/10.1590/S0102-311X2007000700020.	Brazil	Cross-sectional	TF	TI, TF and/or TI	no	no	no	no	Checklist for Prevalence Studies	Yes	Unclear	Unclear	No	Yes	Yes	Yes	Yes	Yes											[6/9] (67%)	NA	67%
Roba, A. A.; Patel, D.; Zondervan, M.Risk of trachoma in a SAFE intervention area. 2013. International Ophthalmology. , volume: 33, issue: 1, pages: 53-59. Doi: 10.1007/s10792-012-9632-3.	Ethiopia	Cross-sectional	TF	TI, TF and/or TI	no	no	no	no	Checklist for Prevalence Studies	No, excluded communities receiving more frequent MDAs as part of other intervention studies	Yes	Yes	Yes	Yes	Yes	Yes	Yes	Yes											[8/9] (89%)	NA	89%
D.S, RosenTrachoma among children in western Yemen. 1995. . , volume: 3, issue: , pages: 45245. Doi: .	Yemen	Cross-sectional	TF	TI	no	no	no	no	Checklist for Prevalence Studies	Unclear	Yes	Unclear	No	Unclear	Unclear	Unclear	Unclear	Unclear											[1/9] (11%)	NA	11%
Ruberanziza, E.; Mupfasoni, D.; Nizeyimana, V.; Karibushi, B.; Kabera, M.; Kaberuka, T.; Kabanda, G.; Sebeza, J.; Kramer, M. H.; Ruxin, J.; Fenwick, A.; Kalua, K.Prevalence and risk factors for trachoma in Rwanda. 2009. East African Journal of Public Health. , volume: 6, issue: 3, pages: 287-91. Doi: .	Rwanda	Cross-sectional	TF	TI	no	no	no	no	Checklist for Prevalence Studies	Yes	Yes	Yes	Yes	Yes	Yes	Yes	Yes	Yes											[9/9] (100%)	NA	100%
Saboya-Diaz, M. I.; Angeles, C. A. C.; Socorro Avellaneda Yajahuanca, R. del; Melendez Ruiz, S. K.; Cabrera, R.; Honorio Morales, H. A.; Pachas, P. E.; Guardo, M.; Renneker, K. K.; Munoz, B. E.; West, S. K.Associated factors of the co-occurrence of trachoma and soil-transmitted helminthiases in children 1 to 9 years old in rural communities of the Amazon basin in Loreto Department, Peru: results from a population-based survey. 2022. PLoS Neglected Tropical Diseases. , volume: 16, issue: 7, pages: . Doi: http://dx.doi.org/10.1371/journal.pntd.0010532.	Peru	Cross-sectional	TF	no	no	yes	no	no	Checklist for Prevalence Studies	Yes	Yes	Yes	Yes	Unclear	Yes	Yes	Yes	Unclear											[7/9] (78%)	NA	78%

S6. Table of included studies

										Risk of Bias checklist items**																						
reference	Country or countries of data collection	Type of study	TF or FPC	other clinical indicators?	photo-graded TF or FPC?	infection?	serology?	SCR?	Critical Appraisal Tool used**	Prevalence study: Was the sample frame appropriate to address the target pop?	Prevalence study: Were study participants sampled in an appropriate way?	Prevalence study: Was the sample size adequate?	Prevalence study: Were the study subjects and setting described in detail?	Prevalence study: Was the data analysis conducted with sufficient coverage of the identified sample?	Prevalence study: Were valid methods used for the identification of the condition(s)?	Prevalence study: Was the condition measured in a standard, reliable way for all participants?	Prevalence study: Was there appropriate statistical analysis?	Prevalence study: Was the response rate adequate, and if not, was the low response rate managed appropriately?	DTA study: Was a consecutive or random sample of patients enrolled?	DTA study: Was a case-control design avoided?	DTA study: Did the study avoid inappropriate exclusions?	DTA: Were the index test results interpreted without knowledge of the results of the reference standard?	DTA: If a threshold was used, was it pre-specified?	DTA: Is the reference standard likely to correctly classify the target condition?	DTA: Were the reference standard results interpreted without knowledge of the results of the index test?	DTA: Was there an appropriate interval between index test and reference standard?	DTA: Did all patients receive the same reference standard?	DTA: Were all patients included in the analysis?	Prevalence study score [meets criteria/total] (%)	DTA study score [meets criteria/total] (%)	Max score (if both checklists used, higher of 2 scores)
Sanders, A. M.; Stewart, A. E. P.; Makoy, S.; Chebet, J. J.; Magok, P.; Kuol, A.; Blauvelt, C.; Lako, R.; Rumunu, J.; Callahan, E. K.; Nash, S. D.Burden of trachoma in five counties of Eastern Equatoria state, South Sudan: results from population-based surveys. 2017. PLoS Neglected Tropical Diseases. , volume: 11, issue: 6, pages: e0005658. Doi: .	South Sudan	Cross-sectional	TF	TI, TF and/or TI	no	no	no	no	Checklist for Prevalence Studies	Yes	Yes	Yes	Yes	Yes	Yes	Yes	Yes	Yes											[9/9] (100%)	NA	100%
Sanders, A. M.; Abdalla, Z.; Elshafie, B. E.; Elsanosi, M.; Nute, A. W.; Aziz, N.; Callahan, E. K.; Nash, S. D.Progress toward elimination of trachoma as a public health problem in seven localities in the republic of Sudan: results from population-based surveys. 2019. American Journal of Tropical Medicine and Hygiene. , volume: 101, issue: 6, pages: 1296-1302. Doi: .	Sudan	Cross-sectional	TF	TI	no	no	no	no	Checklist for Prevalence Studies	Yes	Yes	Yes	Yes	No	Yes	Yes	Yes	No											[7/9] (78%)	NA	78%
Sanders, A. M.; Abdalla, Z.; Elshafie, B. E.; Nute, A. W.; Long, E. F.; Aziz, N.; Weiss, P.; Callahan, E. K.; Nash, S. D.Prevalence of trachoma within refugee camps serving South Sudanese refugees in White Nile State, Sudan: results from population-based surveys. 2019. PLoS Neglected Tropical Diseases. , volume: 13, issue: 6, pages: e0007491. Doi: http://dx.doi.org/10.1371/journal.pntd.0007491.	Sudan	Cross-sectional	TF	TI	no	no	no	no	Checklist for Prevalence Studies	Yes	Yes	Yes	Yes	Yes	Yes	Yes	Yes	Yes											[9/9] (100%)	NA	100%
Schellini, S. A.; Lavezzo, M. M.; Ferraz, L. B.; Olbrich Neto, J.; Medina, N. H.; Padovani, C. R.Prevalence and spatial distribution of trachoma among schoolchildren in Botucatu, Sao Paulo-Brazil. 2010. Prevalencia e localizacao espacial dos casos de tracoma detectados em escolares de Botucatu, Sao Paulo-Brasil.. , volume: 73, issue: 4, pages: 358-362. Doi: http://dx.doi.org/10.1590/S0004-27492010000400012.	Brazil	Cross-sectional	TF	TI	no	no	no	no	Checklist for Prevalence Studies	No, school-based	Yes	Yes	Yes	Unclear	Yes	Yes	Yes	Unclear											[6/9] (67%)	NA	67%
Schemann, J. F.; Sacko, D.; Banou, A.; Bamani, S.; Bore, B.; Coulibaly, S.; Ag El Mouchtahide, M.Mapping trachoma in Mali: Results of a national survey. [French]. 1998. Bulletin of the World Health Organization. , volume: 76(6), issue: , pages: 599-606. Doi: .	Mali	Cross-sectional	TF	TI	no	no	no	no	Checklist for Prevalence Studies	Yes	Yes	Unclear	Yes	Unclear	Yes	Yes	Yes	Unclear											[6/9] (67%)	NA	67%
See, C. W.; Alemayehu, W.; Melese, M.; Zhou, Z.; Porco, T. C.; Shiboski, S.; Gaynor, B. D.; Eng, J.; Keenan, J. D.; Lietman, T. M.How reliable are tests for trachoma?--a latent class approach. 2011. Investigative Ophthalmology & Visual Science. , volume: 52, issue: 9, pages: 6133-7. Doi: https://dx.doi.org/10.1167/iovs.11-7419.	Ethiopia	Cross-sectional	TF	TI	no	yes	no	no	Both	Yes	Yes	Unclear	No	Unclear	Yes	Yes	Yes	Unclear	Random	Yes	Yes	Yes	Yes	Yes	Yes	Yes	Yes	Yes	[5/9] (56%)	[9/10] (90%)	90%
Senyonjo, L. G.; Debrah, O.; Martin, D. L.; Asante-Poku, A.; Migchelsen, S. J.; Gwyn, S.; deSouza, D. K.; Solomon, A. W.; Agyemang, D.; Biritwum-Kwadwo, N.; Marfo, B.; Bakajika, D.; Mensah, E. O.; Aboe, A.; Koroma, J.; Addy, J.; Bailey, R.Serological and PCR-based markers of ocular Chlamydia trachomatis transmission in northern Ghana after elimination of trachoma as a public health problem. 2018. PLoS Neglected Tropical Diseases. , volume: 12, issue: 12, pages: e0007027. Doi: .	Ghana	Cross-sectional	TF	no	no	yes	yes	yes	Both	Yes	Yes	Unclear	Yes	Yes	Yes	Yes	Yes	Yes	Random	Yes	Yes	Yes	Yes	Yes	Yes	Yes	Yes	Yes	[8/9] (89%)	[9/10] (90%)	90%
Mohammed, Shaffi; Abebe, BejigaCommon eye diseases in children of rural community in Goro district, Central Ethiopia. 2005. Ethiopian Journal of Health Development. , volume: 19, issue: 2, pages: 148-152. Doi: .	Ethiopia	Cross-sectional	TF	TI, TF and/or TI	no	no	no	no	Checklist for Prevalence Studies	Yes	Yes	Yes	Yes	Unclear	Yes	Yes	Yes	Unclear											[7/9] (78%)	NA	78%
Sheehan, J. P.; Gebresillasie, S.; Shiferaw, A.; Aragie, S.; Tadesse, Z.; Tadesse, D.; Somkijrungroj, T.; Stoller, N. E.; Callahan, E. K.; Emerson, P. M.; Lietman, T. M.; Keenan, J. D.School-Based versus Community-Based Sampling for Trachoma Surveillance. 2018. American Journal of Tropical Medicine and Hygiene. , volume: 99(1), issue: , pages: 150-154. Doi: https://dx.doi.org/10.4269/ajtmh.17-0904.	Ethiopia	Cross-sectional	TF	no	no	yes	no	no	Checklist for Prevalence Studies	Yes	Yes	Unclear	No, No number sampled	Unclear	Yes	Yes	Yes	Unclear											[5/9] (56%)	NA	56%
Shekhawat, N.; Mkocha, H.; Munoz, B.; Gaydos, C.; Dize, L.; Quinn, T. C.; West, S. K.Cohort and age effects of mass drug administration on prevalence of trachoma: a longitudinal study in rural Tanzania. 2014. Investigative Ophthalmology & Visual Science. , volume: 55, issue: 4, pages: 2307-2314. Doi: http://dx.doi.org/10.1167/iovs.13-12701.	Tanzania	Cross-sectional within longitudinal	TF	no	no	yes	no	no	Checklist for Prevalence Studies	Yes	Yes	Unclear	No	Unclear	Yes	Yes	Yes	Unclear											[5/9] (56%)	NA	56%
Shimelash, A.; Alemayehu, M.; Dagne, H.; Mihiretie, G.; Lamore, Y.; Tegegne, E.; Kumlachew, L.Prevalence of active trachoma and associated factors among school age children in Debre Tabor Town, Northwest Ethiopia, 2019: a community based cross-sectional study. 2022. Italian Journal of Pediatrics. , volume: 48, issue: 1, pages: 61. Doi: https://dx.doi.org/10.1186/s13052-022-01258-x.	Ethiopia	Cross-sectional	TF	TI, TF and/or TI	no	no	no	no	Checklist for Prevalence Studies	Yes	Yes	Yes	Yes	Yes	Yes	Yes	Yes	Yes											[9/9] (100%)	NA	100%
Silva, Evanildo JosÃ© da; Oliveira, Layze Alves Vieira; Nasser, Luciano SÃ³lia; Leite, Luciana Fernandes Amaro; Caldeira, AntÃ´nio Prates; Oliveira, Leida CalegÃ¡rio dePrevalÃªncia de Tracoma em crianÃ§as em idade escolar no municÃpio de Turmalina, MG. 2016. Rev. bras. oftalmol. , volume: 75, issue: 3, pages: 181-184. Doi: 10.5935/0034-7280.20160038.	Brazil	Cross-sectional	TF	TI	no	no	no	no	Checklist for Prevalence Studies	No, school based	Unclear	Yes	Yes	Unclear	Unclear, TF definition Not given or cited	Yes	Yes	Unclear											[4/9] (44%)	NA	44%
Smith, A. G.; Broman, A. T.; Alemayehu, W.; Munoz, B. E.; West, S. K.; Gower, E. W.Relationship between trachoma and chronic and acute malnutrition in children in rural Ethiopia. 2007. Journal of Tropical Pediatrics. , volume: 53(5), issue: , pages: 308-312. Doi: https://dx.doi.org/10.1093/tropej/fmm039.	Ethiopia	Cross-sectional	TF	TI, TF and/or TI	no	yes	no	no	Both	No, only includes children w/ a relative who had TT surgery	Not for a prevalence study	Unclear	Yes	Unclear	Yes	Yes	Yes	Unclear	Unclear	Yes	Yes	Unclear	Yes	Yes	Yes	Yes	Yes	Yes	[4/9] (44%)	[8/10] (80%)	80%
Snyder, B. M.; Sie, A.; Tapsoba, C.; Dah, C.; Ouermi, L.; Zakane, S. A.; Keenan, J. D.; Oldenburg, C. E.Smartphone photography as a possible method of post-validation trachoma surveillance in resource-limited settings. 2019. International Health. , volume: 11(6), issue: , pages: 613-615. Doi: https://dx.doi.org/10.1093/inthealth/ihz035.	Burkina Faso	Cross-sectional	TF	TI	yes	no	no	no	Checklist for DTA studies										Random	Yes	Yes	Unclear	Yes	Yes	Yes	Yes	Yes	Yes	NA	[8/10] (80%)	80%
Solomon, A.Rational use of azithromycin in the control of trachoma : using quantitative PCR to assess distribution of infection and impact of treatment. 2003. . , volume: , issue: , pages: . Doi: .	Tanzania	Cross-sectional within longitudinal	TF	TI, TF and/or TI	no	yes	no	no	Both	Yes	Yes	Yes	Yes	Yes	Yes	Yes	Yes	Yes	NA, whole pop	Yes	Yes	Yes	Yes	Yes	Yes	Yes	Yes	Yes	[9/9] (100%)	[9/10] (90%)	100%
Solomon, A. W.; Harding-Esch, E.; Alexander, N. D. E.; Aguirre, A.; Holland, M. J.; Bailey, R. L.; Foster, A.; Mabey, D. C. W.; Massae, P. A.; Courtright, P.; Shao, J. F.Two doses of azithromycin to eliminate trachoma in a Tanzanian community. 2008. New England Journal of Medicine. , volume: 358, issue: 17, pages: 1870-1871. Doi: http://dx.doi.org/10.1056/NEJMc0706263.	Tanzania	Cross-sectional within longitudinal	TF	no	no	yes	no	no	Checklist for Prevalence Studies	Yes	Yes	Yes	No	Yes	Yes	Yes	Yes	Yes											[8/9] (89%)	NA	89%
Stare, D.; Harding-Esch, E.; Munoz, B.; Bailey, R.; Mabey, D.; Holland, M.; Gaydos, C.; West, S.Design and baseline data of a randomized trial to evaluate coverage and frequency of mass treatment with azithromycin: the Partnership for Rapid Elimination of Trachoma (PRET) in Tanzania and The Gambia. 2011. Ophthalmic Epidemiology. , volume: 18, issue: 1, pages: 20-29. Doi: http://dx.doi.org/10.3109/09286586.2010.545500.	Gambia, Tanzania	Cross-sectional within longitudinal	TF	no	no	yes	no	no	Checklist for Prevalence Studies	Yes	Yes	Unclear	No	Unclear	Yes	Yes	Yes	Unclear											[5/9] (56%)	NA	56%
Stewart, A. E. P.; Mulat, Zerihun; Demelash, Gessese; Berhanu, Melak; Eshetu, Sata; Nute, A. W.; Tigist, Astale; Tekola, Endeshaw; Tesfaye, Teferi; Zerihun, Tadesse; Callahan, E. K.; Melsew, Chanyalew; Birhan, Gaudie; Emerson, P. M.; King, J. D.; Nash, S. D.Progress to eliminate trachoma as a public health problem in Amhara National Regional State, Ethiopia: results of 152 population-based surveys. 2019. American Journal of Tropical Medicine and Hygiene. , volume: 101, issue: 6, pages: 1286-1295. Doi: .	Ethiopia	Cross-sectional	TF	TI, TF and/or TI	no	no	no	no	Checklist for Prevalence Studies	Yes	Yes	Unclear	Yes	Unclear	Yes	Yes	Yes	No											[6/9] (67%)	NA	67%
Tadesse, B.; Worku, A.; Kumie, A.; Yimer, S. A.Effect of water, sanitation and hygiene interventions on active trachoma in North and South Wollo zones of Amhara Region, Ethiopia: A Quasi-experimental study. 2017. PLoS Neglected Tropical Diseases. , volume: 11(11) (no pagination), issue: , pages: . Doi: https://dx.doi.org/10.1371/journal.pntd.0006080.	Ethiopia	Cross-sectional within longitudinal	TF	TI, TF and/or TI	no	no	no	no	Checklist for Prevalence Studies	No, purposively sampled based on water status	Yes	Yes	Yes	Unclear	Yes	Yes	Yes	Unclear											[6/9] (67%)	NA	67%
Beselam, Tadesse; Alemayehu, Worku; Abera, Kumie; Yimer, S. A.The burden of and risk factors for active trachoma in the North and South Wollo Zones of Amhara Region, Ethiopia: a crosssectional study. 2017. Infectious Diseases of Poverty. , volume: 6, issue: 143, pages: . Doi: http://dx.doi.org/10.1186/s40249-017-0358-3.	Ethiopia	Cross-sectional	TF	TI	no	no	no	no	Checklist for Prevalence Studies	Yes	Yes	Yes	Yes	Unclear	Yes	Yes	Yes	Unclear											[7/9] (78%)	NA	78%
Praveen, Vashist; Noopur, Gupta; Rathore, A. S.; Anita, Shah; Suman, SinghRapid assessment of trachoma in underserved population of Car-Nicobar Island, India. 2013. PLoS ONE [Electronic Resource]. , volume: 8, issue: 6, pages: e65918. Doi: http://dx.doi.org/10.1371/journal.pone.0065918.	India	Cross-sectional	TF	TI, TF and/or TI	no	no	no	no	Checklist for Prevalence Studies	No, purposive (TRA)	Yes	Unclear	Yes	Unclear	Yes	Yes	Yes	Unclear											[5/9] (56%)	NA	56%
Wang, M.; Lu, X.; Hu, A.; Zhang, M.; Li, X.; Deng, S.; Wang, N.Etiological characteristics of chlamydia trachoma conjunctivitis of Primary Boarding School students in the Qinghai Tibetan area. 2016. Science China. , volume: Life sciences. 59(6), issue: , pages: 555-560. Doi: https://dx.doi.org/10.1007/s11427-016-5060-3.	China	Case-control	TF	no	no	yes	no	no	Checklist for Prevalence Studies	No, school based	Unclear	Unclear	Yes	Unclear	No, swabs included both upper tarsal and conj sac. In addition, cases were students with any number of follicles.	Yes	Yes	Unclear											[3/9] (33%)	NA	33%
West, S.; Munoz, B.; Bobo, L.; Quinn, T. C.; Mkocha, H.; Lynch, M.; Mmbaga, B. B. O.; Viscidi, R.Nonocular chlamydia infection and risk of ocular reinfection after mass treatment in a trachoma hyperendemic area. 1993. Investigative Ophthalmology and Visual Science. , volume: 34(11), issue: , pages: 3194-3198. Doi: .	Tanzania	Cross-sectional	TF	TI	no	yes	no	no	Checklist for Prevalence Studies	Yes	Unclear	Unclear	No, No year	Unclear	Yes	Yes	Yes	Unclear											[4/9] (44%)	NA	44%
West, S. K.; Munoz, B.; Mkocha, H.; Hsieh, Y. H.; Lynch, M. C.Progression of active trachoma to scarring in a cohort of Tanzanian children. 2001. Ophthalmic Epidemiology. , volume: 8, issue: 44960, pages: 137-44. Doi: .	Tanzania	Case-control	TF	TI	no	yes	no	no	Checklist for Prevalence Studies	No, case control	Unclear	Unclear	Yes	Unclear	Yes	Yes	Yes	Unclear											[4/9] (44%)	NA	44%
West, S. K.; Munoz, B.; Mkocha, H.; Gaydos, C.; Quinn, T.Trachoma and ocular Chlamydia trachomatis were not eliminated three years after two rounds of mass treatment in a trachoma hyperendemic village. 2007. Investigative Ophthalmology & Visual Science. , volume: 48, issue: 4, pages: 1492-1497. Doi: .	Tanzania	Cross-sectional within longitudinal	TF	TI	no	yes	no	no	Checklist for Prevalence Studies	Yes	Yes	Yes	Yes	Yes	Yes	Yes	Yes	Yes											[9/9] (100%)	NA	100%

S6. Table of included studies

										Risk of Bias checklist items**																					
reference	Country or countries of data collection	Type of study	TF or FPC	other clinical indicators?	photo-graded TF or FPC?	infection?	serology?	SCR?	Critical Appraisal Tool used**	Prevalence study: Was the sample frame appropriate to address the target pop?	Prevalence study: Were study participants sampled in an appropriate way?	Prevalence study: Was the sample size adequate?	Prevalence study: Were the study subjects and setting described in detail?	Prevalence study: Was the data analysis conducted with sufficient coverage of the identified sample?	Prevalence study: Were valid methods used for the identification of the condition(s)?	Prevalence study: Was the condition measured in a standard, reliable way for all participants?	Prevalence study: Was there appropriate statistical analysis?	Prevalence study: Was the response rate adequate, and if not, was the low response rate managed appropriately?	DTA study: Was a consecutive or random sample of patients enrolled?	DTA study: Was a case-control design avoided?	DTA study: Did the study avoid inappropriate exclusions?	DTA: Were the index test results interpreted without knowledge of the results of the reference standard?	DTA: If a threshold was used, was it pre-specified?	DTA: Is the reference standard likely to correctly classify the target condition?	DTA: Were the reference standard results interpreted without knowledge of the results of the index test?	DTA: Was there an appropriate interval between index test and reference standard?	DTA: Did all patients receive the same reference standard?	DTA: Were all patients included in the analysis?	Prevalence study score [meets criteria/total] (%)	DTA study score [meets criteria/total] (%)	Max score (if both checklists used, higher of 2 scores)
West, S. K.; Stare, D.; Mkocha, H.; Munoz, B.; Gaydos, C.; Quinn, T. C.Do infants increase the risk of re-emergent infection in households after mass drug administration for trachoma?. 2011. Investigative Ophthalmology & Visual Science. , volume: 52, issue: 8, pages: 6040-2. Doi: https://dx.doi.org/10.1167/iovs.11-7372.	Tanzania	Cross-sectional within longitudinal	TF	TI	no	yes	no	no	Checklist for Prevalence Studies	Yes	Yes	Yes	No	Yes	Yes	Yes	Yes	Yes											[8/9] (89%)	NA	89%
West, S. K.; Bailey, R.; Munoz, B.; Edwards, T.; Mkocha, H.; Gaydos, C.; Lietman, T.; Porco, T.; Mabey, D.; Quinn, T. C.A randomized trial of two coverage targets for mass treatment with azithromycin for trachoma. 2013. PLoS Neglected Tropical Diseases. , volume: 7, issue: 8, pages: e2415. Doi: http://dx.doi.org/10.1371/journal.pntd.0002415.	Tanzania	Cross-sectional within longitudinal	TF	no	no	yes	no	no	Checklist for Prevalence Studies	Yes	Unclear	Yes	No	Unclear	Yes	Yes	Yes	Unclear											[5/9] (56%)	NA	56%
West, S. K.; Munoz, B. E.; Mkocha, H.; Gaydos, C.; Quinn, T.Risk of infection with Chlamydia trachomatis from migrants to communities undergoing mass drug administration for trachoma control. 2015. Special Issue: Trachoma.. , volume: 22, issue: 3, pages: 170-175. Doi: .	Tanzania	Cross-sectional within longitudinal	TF	no	no	yes	no	no	Checklist for Prevalence Studies	Yes	Yes	Unclear	No	Unclear	Yes	Yes	Yes	Unclear											[5/9] (56%)	NA	56%
West, S. K.; Munoz, B.; Weaver, J.; Mrango, Z.; Dize, L.; Gaydos, C.; Quinn, T. C.; Martin, D. L.Can we use antibodies to Chlamydia trachomatis as a surveillance tool for national trachoma control programs? Results from a district survey. 2016. PLoS Neglected Tropical Diseases. , volume: 10, issue: 1, pages: e0004352. Doi: http://dx.doi.org/10.1371/journal.pntd.0004352.	Tanzania	Cross-sectional	TF	no	no	yes	yes	no	Checklist for Prevalence Studies	Yes	Yes	Yes	No	Yes	Yes	Yes	Yes	Yes											[8/9] (89%)	NA	89%
West, S. K.; Munoz, B.; Mkocha, H.; Dize, L.; Gaydos, C. A.; Swenor, B.; Ervin, A. M.; Quinn, T. C.Treating village newcomers and travelers for trachoma: results from ASANTE cluster randomized trial. 2017. PLoS ONE [Electronic Resource]. , volume: 12, issue: 6, pages: e0178595. Doi: http://dx.doi.org/10.1371/journal.pone.0178595.	Tanzania	Cross-sectional within longitudinal	TF	no	no	yes	no	no	Checklist for Prevalence Studies	Yes	Yes	Unclear	No	Unclear	Yes	Yes	Yes	Unclear											[5/9] (56%)	NA	56%
West, S. K.; Zambrano, A. I.; Sharma, S.; Mishra, S. K.; Munoz, B. E.; Dize, L.; Crowley, K.; Gaydos, C. A.; Rotondo, L. A.Surveillance surveys for reemergent trachoma in formerly endemic districts in Nepal from 2 to 10 years after mass drug administration cessation. 2017. JAMA Ophthalmology. , volume: 135, issue: 11, pages: 1141-1146. Doi: http://dx.doi.org/10.1001/jamaophthalmol.2017.3062.	Nepal	Cross-sectional	TF	no	no	yes	yes	no	Checklist for Prevalence Studies	Yes	Yes	Yes	Yes	Unclear	Yes	Yes	Yes	Unclear											[7/9] (78%)	NA	78%
West, S. K.; Munoz, B.; Kaur, H.; Dize, L.; Mkocha, H.; Gaydos, C. A.; Quinn, T. C.Longitudinal change in the serology of antibodies to Chlamydia trachomatis pgp3 in children residing in a trachoma area. 2018. Scientific Reports. , volume: 8, issue: 1, pages: 3520. Doi: http://dx.doi.org/10.1038/s41598-018-21127-0.	Tanzania	Cross-sectional	TF	no	no	yes	yes	yes	Checklist for Prevalence Studies	Yes	Yes	Yes	Yes	Unclear	Yes	Yes	Yes	Unclear											[7/9] (78%)	NA	78%
West, S. K.; Nanji, A. A.; Mkocha, H.; Munoz, B.; Gaydos, C.; Quinn, T. C.Evidence for contamination with C. trachomatis in the household environment of children with active trachoma: a cross-sectional study in Kongwa, Tanzania. 2019. PLoS Neglected Tropical Diseases. , volume: 13, issue: 12, pages: . Doi: http://dx.doi.org/10.1371/journal.pntd.0007834.	Tanzania	Cross-sectional	TF	no	no	yes	no	no	Checklist for Prevalence Studies	Yes	Yes	Yes	No, No year	Unclear	Yes	Yes	Yes	Unclear											[6/9] (67%)	NA	67%
West, S. K.; Munoz, B.; Mkocha, H.; Gaydos, C. A.; Quinn, T. C.The effect of mass drug administration for trachoma on antibodies to Chlamydia trachomatis pgp3 in children. 2020. Scientific Reports. , volume: 10, issue: 9, pages: . Doi: http://dx.doi.org/10.1038/s41598-020-71833-x.	Tanzania	Cross-sectional	TF	no	no	yes	yes	yes	Checklist for Prevalence Studies	Yes	Yes	Yes	Yes	Unclear	Yes	Yes	Yes	Unclear											[7/9] (78%)	NA	78%
Wilson, N.; Goodhew, B.; Mkocha, H.; Joseph, K.; Bandea, C.; Black, C.; Igietseme, J.; Munoz, B.; West, S. K.; Lammie, P.; Kasubi, M.; Martin, D. L.Evaluation of a single dose of azithromycin for trachoma in low-prevalence communities. 2019. Ophthalmic Epidemiology. , volume: 26, issue: 1, pages: 44932. Doi: http://dx.doi.org/10.1080/09286586.2017.1293693.	Tanzania	Cross-sectional within longitudinal	TF	no	no	yes	yes	no	Checklist for Prevalence Studies	Yes	Yes	Yes	Yes	Unclear	Yes	Yes	Yes	Unclear											[7/9] (78%)	NA	78%
Woldekidan, E.; Daka, D.; Legesse, D.; Laelago, T.; Betebo, B.Prevalence of active trachoma and associated factors among children aged 1 to 9 years in rural communities of Lemo district, southern Ethiopia: Community based cross sectional study. 2019. BMC Infectious Diseases. , volume: 19(1) (no pagination), issue: , pages: . Doi: https://dx.doi.org/10.1186/s12879-019-4495-0.	Ethiopia	Cross-sectional	TF	TI, TF and/or TI	no	no	no	no	Checklist for Prevalence Studies	Yes	Yes	Yes	Yes	Yes	Yes	Yes	Yes	Yes											[9/9] (100%)	NA	100%
Yoseph, Worku; Samson, BayuScreening for ocular abnormalities and subnormal vision in school children of Butajira town, southern Ethiopia. 2002. Ethiopian Journal of Health Development. , volume: 16, issue: 2, pages: 165-171. Doi: .	Ethiopia	Cross-sectional	TF	TI	no	no	no	no	Checklist for Prevalence Studies	No, school based	Yes	Unclear	Yes	Unclear	Yes	Yes	Yes	Yes											[6/9] (67%)	NA	67%
Xue, WenWen; Lu, LiNa; Zhu, JianFeng; He, XianGui; He, JiangNan; Zhao, Rong; Zou, HaiDongA cross-sectional population-based survey of trachoma among migrant school aged children in Shanghai, China. 2016. BioMed Research International. , volume: 2016, issue: , pages: Article-8692685. Doi: .	China	Cross-sectional	TF	TI	no	no	no	no	Checklist for Prevalence Studies	Yes	Unclear	Unclear	Yes	Yes	Yes	Yes	Yes	Yes											[7/9] (78%)	NA	78%
Yang, J. L.; Schachter, J.; Moncada, J.; Habte, D.; Zerihun, M.; House, J. I.; Zhou, Z.; Hong, K. C.; Maxey, K.; Gaynor, B. D.; Lietman, T. M.Comparison of an rRNA-based and DNA-based nucleic acid amplification test for the detection of Chlamydia trachomatis in trachoma. 2007. British Journal of Ophthalmology. , volume: 91(3), issue: , pages: 293-295. Doi: https://dx.doi.org/10.1136/bjo.2006.099150.	Ethiopia	Cross-sectional	TF	TI, TF and/or TI	no	yes	no	no	Both	Yes	Unclear	Unclear	No	Unclear	Yes	Yes	Yes	Unclear	Consecutive	Yes	Yes	Unclear	Yes	Yes	Yes	Yes	Yes	Yes	[4/9] (44%)	[8/10] (80%)	80%
Yang, J. L.; Hong, K. C.; Schachter, J.; Moncada, J.; Lekew, T.; House, J. I.; Zhou, Z.; Neuwelt, M. D.; Rutar, T.; Halfpenny, C.; Shah, N.; Whitcher, J. P.; Lietman, T. M.Detection of Chlamydia trachomatis ocular infection in trachoma-endemic communities by rRNA amplification. 2009. Investigative Ophthalmology and Visual Science. , volume: 50(1), issue: , pages: 90-94. Doi: https://dx.doi.org/10.1167/iovs.08-2247.	Ethiopia	Cross-sectional	TF	TI, TF and/or TI	no	yes	no	no	Both	Yes	Yes	Unclear	Yes	Unclear	Yes	Yes	Yes	Unclear	Random	Yes	Yes	Unclear	Yes	Yes	Yes	Yes	Yes	Yes	[6/9] (67%)	[8/10] (80%)	80%
Yaya, G.; Kemata, B.; Baanam, M. Y.; Bobossi-Serengbe, G.Blinding trachoma: results of a prevalence survey in 8 health districts in CAR. 2015. Le trachome evolutif: resultats d'une enquete de prevalence dans huit prefectures sanitaires en RCA.. , volume: 108, issue: 4, pages: 299-304. Doi: http://dx.doi.org/10.1007/s13149-015-0446-1.	Central African Republic	Cross-sectional	TF	TI	no	no	no	no	Checklist for Prevalence Studies	Yes	Yes	Yes	Yes	Yes	Yes	Yes	Yes	Yes											[9/9] (100%)	NA	100%
Yayemain, D.; King, J. D.; Debrah, O.; Emerson, P. M.; Aboe, A.; Ahorsu, F.; Wanye, S.; Ansah, M. O.; Gyapong, J. O.; Hagan, M.Achieving trachoma control in Ghana after implementing the SAFE strategy. 2009. Transactions of the Royal Society of Tropical Medicine and Hygiene. , volume: 103, issue: 10, pages: 993-1000. Doi: http://dx.doi.org/10.1016/j.trstmh.2009.02.007.	Ghana	Cross-sectional	TF	TI, TF and/or TI	no	no	no	no	Checklist for Prevalence Studies	Unclear	Unclear	Unclear	No	Unclear	Yes	Unclear	Unclear	Unclear											[1/9] (11%)	NA	11%
Yilkal, Alemu; Abebe, BejigaThe impact of water supply on trachoma prevalence. 2004. Ethiopian Medical Journal. , volume: 42, issue: 3, pages: 179-184. Doi: .	Ethiopia	Cross-sectional	TF	TI	no	no	no	no	Checklist for Prevalence Studies	Yes	Yes	Yes	Yes	Unclear	Yes	Yes	Yes	Unclear											[7/9] (78%)	NA	78%
Yohannan, J.; Munoz, B.; Mkocha, H.; Gaydos, C. A.; Bailey, R.; Lietman, T. A.; Quinn, T.; West, S. K.Can we stop mass drug administration prior to 3 annual rounds in communities with low prevalence of trachoma?: PRET Ziada trial results. 2013. JAMA Ophthalmology. , volume: 131, issue: 4, pages: 431-6. Doi: https://dx.doi.org/10.1001/jamaophthalmol.2013.2356.	Tanzania	Cross-sectional within longitudinal	TF	no	no	yes	no	no	Checklist for Prevalence Studies	Yes	Yes	Yes	No	Unclear	Yes	Yes	Yes	Unclear											[6/9] (67%)	NA	67%
Zambrano, A. I.; Sharma, S.; Crowley, K.; Dize, L.; Munoz, B. E.; Mishra, S. K.; Rotondo, L. A.; Gaydos, C. A.; West, S. K.The World Health Organization Recommendations for Trachoma Surveillance, Experience in Nepal and Added Benefit of Testing for Antibodies to Chlamydia trachomatis pgp3 Protein: NESTS Study. 2016. PLoS Neglected Tropical Diseases. , volume: 10(9) (no pagination), issue: , pages: . Doi: https://dx.doi.org/10.1371/journal.pntd.0005003.	Nepal	Cross-sectional	TF	no	no	yes	yes	no	Checklist for Prevalence Studies	Yes	Yes	Unclear	No	Unclear	Yes	Yes	Yes	Unclear											[5/9] (56%)	NA	56%
Zambrano, A. I.; Munoz, B. E.; Mkocha, H.; Dize, L.; Gaydos, C. A.; Quinn, T.; West, S. K.Measuring trachomatous inflammation-intense (TI) when prevalence is low provides data on infection with Chlamydia trachomatis. 2017. Investigative Ophthalmology & Visual Science. , volume: 58, issue: 2, pages: 997-1000. Doi: .	Tanzania	Cross-sectional within longitudinal	TF	TI, TF and/or TI	no	yes	no	no	Both	Yes	Yes	Yes	Yes	Unclear	Yes	Yes	Yes	Yes	Random	Yes	Yes	Yes	Yes	Yes	Yes	Yes	Yes	Yes	[8/9] (89%)	[9/10] (90%)	90%
Zerihun, N.Trachoma in Jimma zone, South Western Ethiopia. 1997. Tropical Medicine and International Health. , volume: 2, issue: 12, pages: 1115-1121. Doi: .	Ethiopia	Cross-sectional	TF	TI, TF and/or TI	no	no	no	no	Checklist for Prevalence Studies	Yes	Yes	Yes	Yes	Unclear	Yes	Yes	Yes	Yes											[8/9] (89%)	NA	89%
TF: trachomatous inflammation--follicular, as measured by the World Health Organization (WHO) Simplified Grading System																															0%
TI: trachomatous inflammation--intense, as measured by the WHO Simplified Grading System																															0%
RCT: randomised control trial																															0%
FPC: follicles, papillae, cicatrices system																															0%
SCR: seroconversion rate																															0%
DTA: Diagnostic Test Accuracy																															0%
*reference number refers to 'references' tab within this workbook, may not match reference number in manuscript																															0%
**from the Joanna Briggs Institute (https://jbi.global/critical-appraisal-tools)																															0%
