# Supplementary material for: Comparison of Methods for Assessing Chlamydia trachomatis Transmission Intensity: A Systematic Review
Source: Am J Trop Med Hyg. 2026 Sep 22:tpmd250541. Online ahead of print. doi: 10.4269/ajtmh.25-0541 (PMC13615270; doi:10.4269/ajtmh.25-0541)
Supplement: Supplementary file 9 [file tpmd250541.SD7.pdf]

S7. Analysis by region, publication year

Studies by region

Region (World Health Organization)	Number of studies included in review	Percent of total number of studies*
African Region	177	75%
Region of the Americas	14	6%
Eastern Mediterranean Region	14	6%
South-East Asia Region	7	3%
Western Pacific Region	19	8%
Country not specified	2	1%

* Total number of studies is 235. Studies may have included data from one or more regions.

Studies by publication year

Publication Year	Number of studies	Running total	Running percent
1991	2	2	1%
1992	2	4	2%
1993	1	5	2%
1994	2	7	3%
1995	1	8	3%
1996	1	9	4%
1997	1	10	4%
1998	2	12	5%
1999	1	13	6%
2001	3	16	7%
2002	1	17	7%
2003	4	21	9%
2004	2	23	10%
2005	7	30	13%
2006	10	40	17%
2007	8	48	20%
2008	7	55	23%
2009	12	67	29%
2010	15	82	35%
2011	10	92	39%
2012	8	100	43%
2013	15	115	49%
2014	9	124	53%
2015	8	132	56%
2016	16	148	63%
2017	21	169	72%
2018	19	188	80%
2019	15	203	86%
2020	13	216	92%
2021	9	225	96%
2022	10	235	100%
