# Supplementary material for: Comparison of Methods for Assessing Chlamydia trachomatis Transmission Intensity: A Systematic Review
Source: Am J Trop Med Hyg. 2026 Sep 22:tpmd250541. Online ahead of print. doi: 10.4269/ajtmh.25-0541 (PMC13615270; doi:10.4269/ajtmh.25-0541)
Supplement: Supplementary file 10 [file tpmd250541.SD8.pdf]

S8. Bland-Altman plot

Field grade vs. photo grade, follicular trachoma*

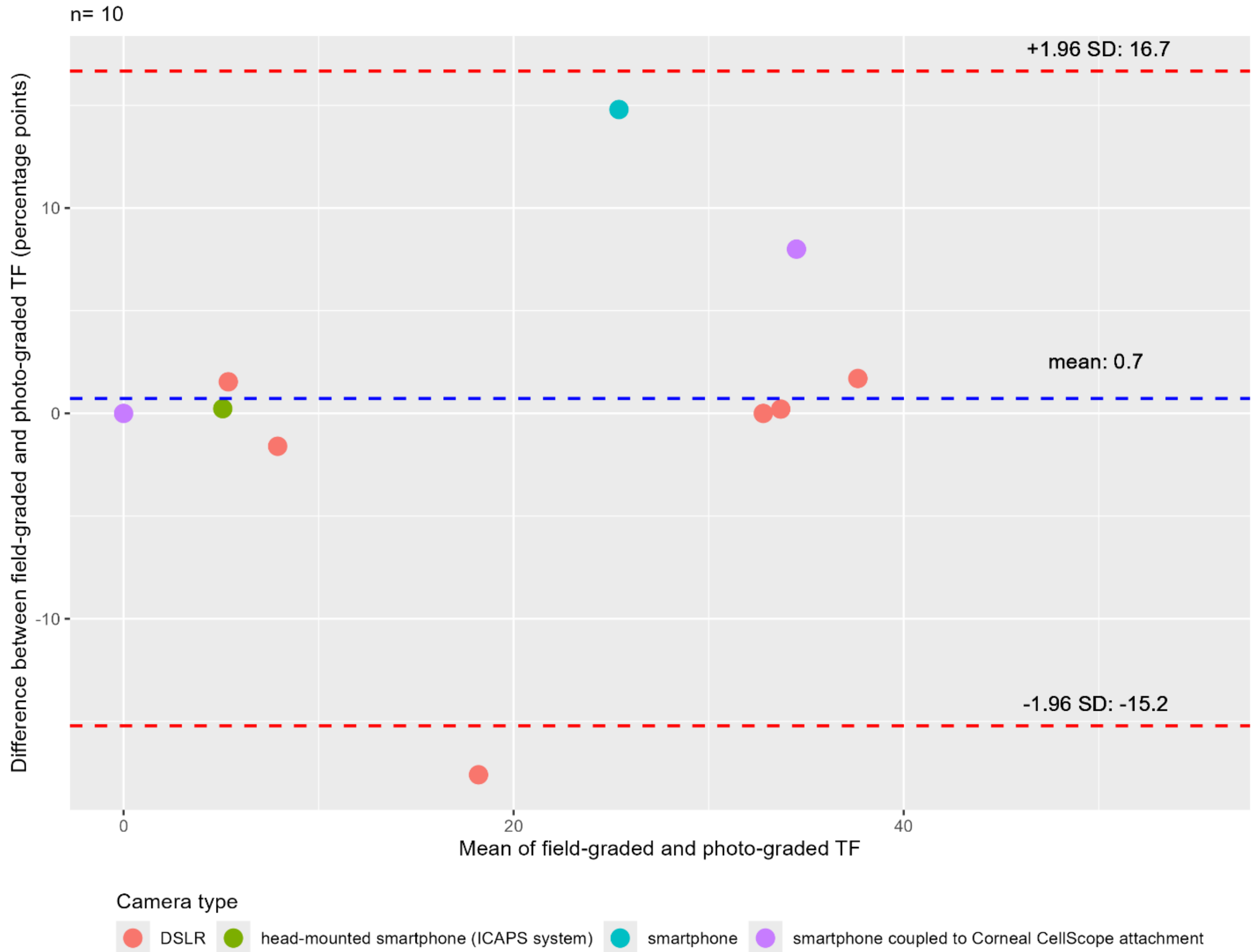

*measured as trachomatous inflammation—follicular in the simplified World Health Organization system or F2/F3 in the Follicles, Papillae, Cicatrices system

TF = follicular trachoma, SD = standard deviation, DSLR = digital single-lens reflex
