# Supplementary material for: Comparison of Methods for Assessing Chlamydia trachomatis Transmission Intensity: A Systematic Review
Source: Am J Trop Med Hyg. 2026 Sep 22:tpmd250541. Online ahead of print. doi: 10.4269/ajtmh.25-0541 (PMC13615270; doi:10.4269/ajtmh.25-0541)
Supplement: Supplementary file 11 [file tpmd250541.SD9.pdf]

S9. TI vs. infection, seroprevalence, and SCR

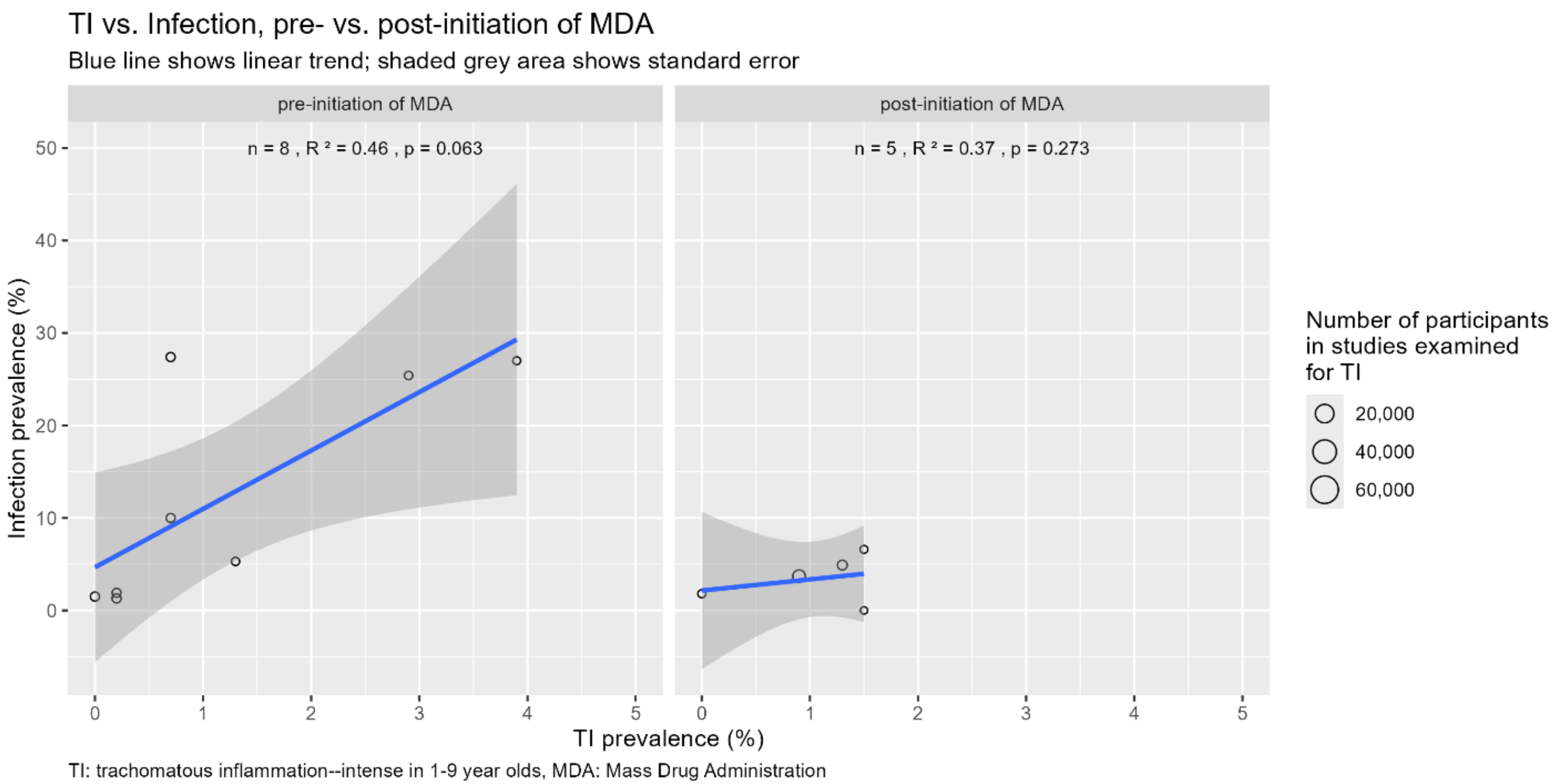

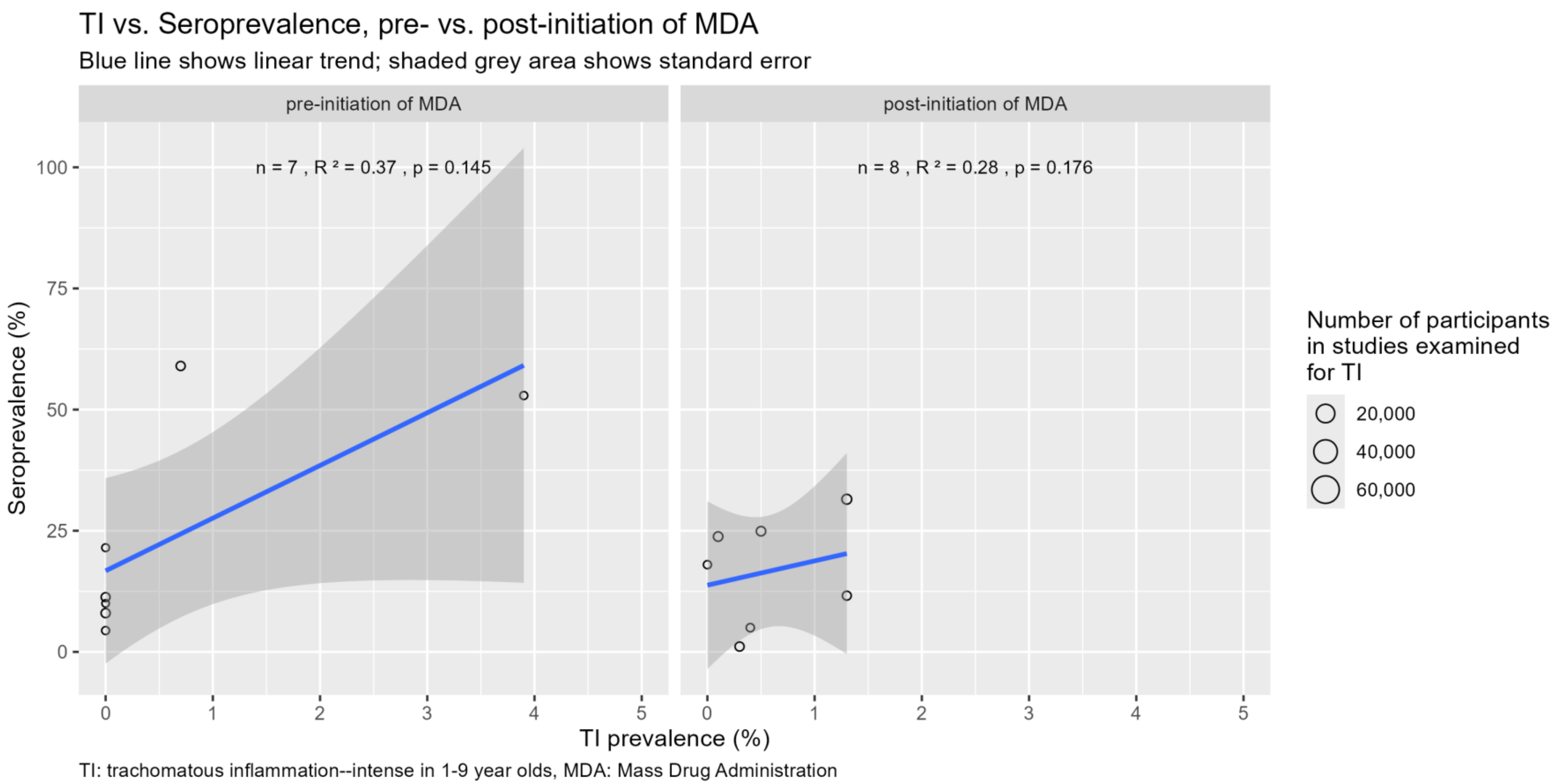
TI vs. Seroprevalence, pre- vs. post-initiation of MDA
Blue line shows linear trend; shaded grey area shows standard error
pre-initiation of MDA
post-initiation of MDA
n = 7 , R ² = 0.37 , p = 0.145
n = 8 , R ² = 0.28 , p = 0.176
Seroprevalence (%)
100
75
50
25
0
0
1
2
3
4
5
TI prevalence (%)
Number of participants in studies examined for TI
20,000
40,000
60,000
TI: trachomatous inflammation--intense in 1-9 year olds, MDA: Mass Drug Administration

TI vs. SCR

Blue line shows linear trend; shaded grey area shows standard error

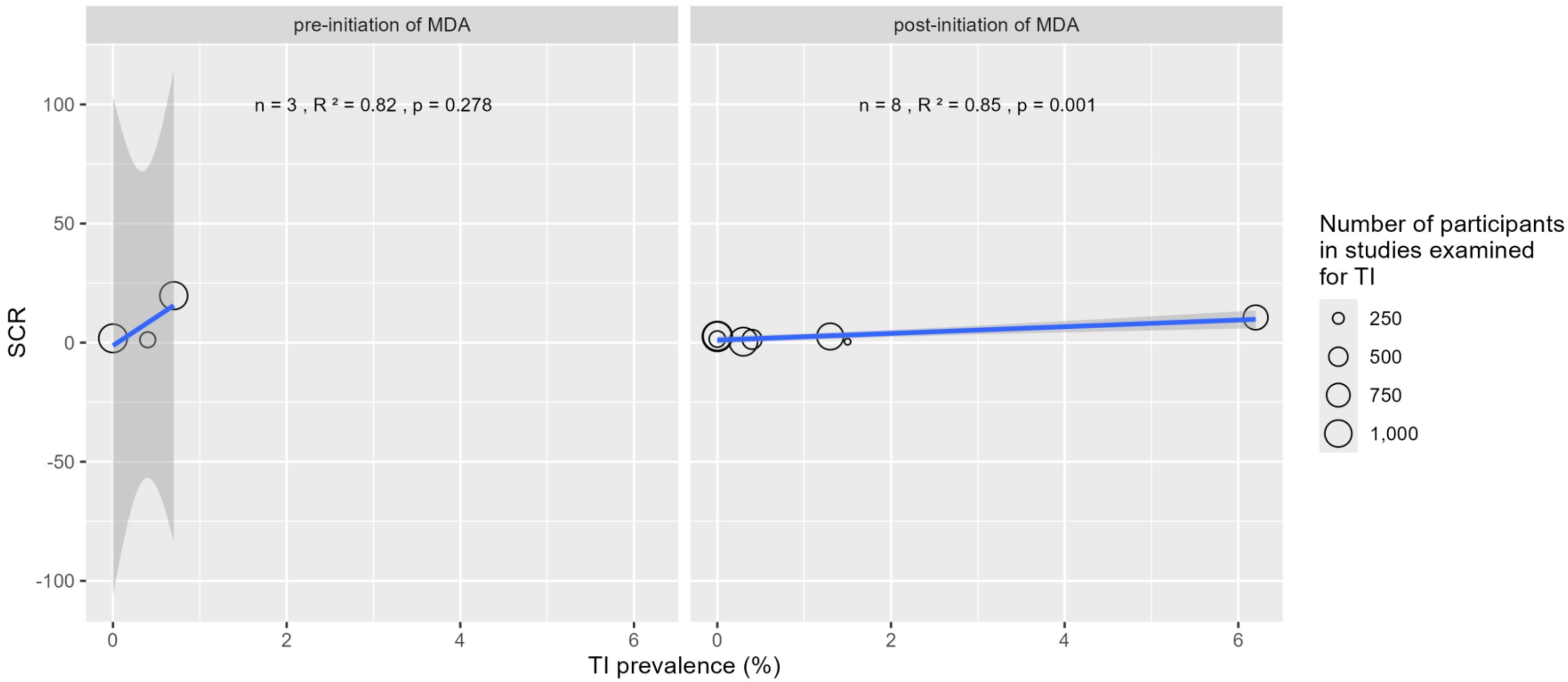

TI: trachomatous inflammation--intense in 1-9 year olds,SCR: Seroconversion rate (per 100 person-years)
